# Effect of traditional Chinese medicine monomers interfering with quorum-sensing on virulence factors of extensively drug-resistant *Acinetobacter baumannii*

**DOI:** 10.3389/fphar.2023.1135180

**Published:** 2023-03-30

**Authors:** Li Zeng, Fei Lin, Baodong Ling

**Affiliations:** ^1^ School of Pharmacy, Chengdu Medical College, Chengdu, China; ^2^ Department of Pharmacy, The Third People’s Hospital of Yibin, Yibin, China; ^3^ Department of Pharmacy, The First Affiliated Hospital of Chengdu Medical College, Chengdu Medical College, Chengdu, China

**Keywords:** traditional Chinese medicine monomer, extensively drug-resistant *A. baumannii*, quorum sensing inhibitor, virulence factor, quorum sensing

## Abstract

The antimicrobial resistance of *Acinetobacter baumannii* (*A. baumannii*) clinical isolates has emerged as a great threat to public health. Quorum sensing (QS) is one of the resistance mechanisms for drug-resistant *A. baumannii*. Interfering with QS is a promising strategy to combat infections caused by drug-resistant bacteria. This study explored the QS inhibition ability of thirty-four traditional Chinese medicine monomers (TCMMs) and assessed the effect of QS inhibitors (QSIs) on the virulence factors of twelve extensively drug-resistant *A. baumannii* (XDRAB) strains. Nine traditional Chinese medicine monomers, such as caffeic acid, cinnamic acid, and myricetin, were found to be able to inhibit the bacterial QS. Then, at 1/8 of the minimal inhibitory concentration, we found that these QSIs inhibited extensively drug-resistant *A. baumannii* adhesion and biofilm formation and downregulated the expression levels of virulence-associated genes (*abaI, abaR, csuE, pgaA*, and *bap*). In conclusion, nine traditional Chinese medicine monomers have QS inhibitory activity and may downregulate QS genes to interfere with the QS system, which could inhibit the expression of extensively drug-resistant *A. baumannii* virulence factors. These results suggest that traditional Chinese medicine monomers could develop as novel anti-virulence compounds to control extensively drug-resistant *A. baumannii* infections.

## 1 Introduction

Extensively drug-resistant *A. baumannii* (XDRAB) is a superbug-bacteria responsible for nosocomial infections in hospitals through strong intrinsic and acquired resistance mechanisms (Lee et al., 2017), which has been listed as one of the highest-grade resistant pathogens of the ESKAPE family by the World Health Organization (Mulani et al., 2019). In addition, the ability of XDRAB to form biofilms increased colonization and persistence in environments, leading to aggravating drug resistance and bacterial pathogenicity (Yan and Bassler, 2019; Mohammed et al., 2020). Therefore, it is of great necessity to search for novel therapeutic strategies to combat XDRAB infections. Recent studies have revealed the key roles of drug resistance and biofilm formation in *A. baumannii*, which are regulated by the quorum sensing (QS) system (Shaaban et al., 2019; PauL et al., 2020).

QS system is a process of communication between microbial cells. Bacteria coordinate their behaviors in a bacterial cell density-dependent manner through this process (Abisado et al., 2018). In *A. baumannii*, QS is regulated by the two-component system abaI/abaR, which is the luxI and luxR homologs of QS in Gram-negative bacteria (Oh and Han, 2022). The *abaI* gene is responsible for the synthesis of N-acyl-homoserine lactone (AHL) signaling molecules (Kalia, V.C., 2013), and then the QS system senses changes in bacterial cell density by signaling molecules. When the density reaches the threshold, the synthesized signaling molecules interact with the receptor abaR, resulting in a coordinated change in gene expression, which plays an important role in the production of virulence factors and antibiotic resistance (Wu et al., 2020). Thus, interfering with the QS system is an attractive treatment against XDRAB infections by inhibiting bacterial virulence hence effectively reducing bacteria pathogenicity without inducing selective pressure resistance.

It has been reported that some synthetic chemical quorum sensing inhibitors (QSIs) like furanones and pyrrolidones, could successfully inhibit the virulence of pathogens (de Nys et al., 2006). However, the majority of these inhibitors are not only toxic to humans but also expensive. Hence, there is an urgent need to find economic, safe, and effective QSIs. Traditional Chinese medicine (TCM) has been used to treat infectious diseases since ancient times, and due to their low toxicity, many monomers isolated from TCM are now used as anti-virulence drugs or antibiotic adjuvants to improve therapeutic effects and safety (Guzzo et al., 2020; Gao et al., 2021). Therefore, it may be a better choice to search for effective QSIs from traditional Chinese medicine monomers (TCMMs)*.*

In this study, we sought to investigate the QS inhibition ability of thirty-four TCMMs, as well as the effect of TCMMs on virulence factors of XDRAB measured by adhesion ability, biofilm formation ability and the expression of virulence factor-related genes.

## 2 Materials and methods

### 2.1 Bacterial strains

Twelve isolates of XDRAB were derived from the bacterial specimens of patients from the critical care units, internal medicine, and emergency departments at the First Affiliated Hospital of Chengdu Medical College from 2018 to 2020 (Chengdu, China). Standard laboratory methods and ATB New (BioMérieux, France) were used to identify these isolates, which were also confirmed by PCR of the genes *16S rRNA* and *blaOXA-51* (Chang et al., 2015); *Chromobacterium violaceum* 026 (CV026) was purchased from Biobw (Beijing, China); *A. baumannii* ATCC 17978 and *Escherichia coli* DH5a were preserved in our laboratory.

### 2.2 Drugs and reagents

Berberine, ferulic acid, baicalin, baicalein, ursolic acid, naringenin, hesperidin, gallic acid, malic acid, resveratrol, myricetin, vanillin, phloretin, capsaicin, osthole, andrographolide, parthenolide, magnolol, and ellagic acid were purchased from Meilun Biological (Dalian, China). Meanwhile, ampicillin, piperacillin, ampicillin sulbactam (2:1), cefoperazone sulbactam (2:1), efotaxime, ceftazidime, tetracycline, doxycycline, amikacin, gentamicin, ciprofloxacin, levofloxacin, imipenem, meropenem, tigecycline and polymyxin B were purchased from Meilun Biological (Dalian, China); Linoleic acid, kaempferol, 4-terpineol, coumarin, linalool, naringin, matrine, and cinnamaldehyde were purchased from Macklin Biochemical (Shanghai, China); Eugenol, citral, vanillin, cinnamic acid, hordenine, and nootkatone were purchased from Yuanye Biochemical (Shanghai, China); Furanone C30 and C6-HSL were purchased from Sigma-Aldrich (Taufkirchen, Germany); RNA isolation kit, cDNA synthesis kit, and fluorescent dye SYBR Color qPCR Master Mix were purchased from Vazyme Biological (Nanjing, China). Cation-adjusted Muller-Hinton broth (CAMHB) (Comprised of beef extract, caseins hydrolyzates, starch and calcium chloride), Luria Bertani (LB) broth medium (Comprised of tryptone, yeast extract, sodium chloride and glucose), LB agar medium (Comprised of tryptone, yeast extract, sodium chloride and agar) and tryptone soy broth (TSB) medium (Comprised of tryptone, soya peptone, sodium chloride, glucose and dipotassium hydrogen phosphate) were purchased from Haibo Biotechnology (Qingdao, China). Dimethyl sulfoxide (DMSO) and phosphate buffer saline (PBS) were purchased from Kelong Chemical (Chengdu, China).

### 2.3 Minimum inhibitory concentration test

The minimal inhibitory concentration (MIC) of thirty-four TCMMs for CV026 and XDRAB were determined by using the broth microdilution method. Briefly, CV026 and XDRAB were inoculated in LB agar medium at 30°C and 37°C for 16–20 h, respectively. And then resuspended in saline (0.9% sodium chloride) to produce a 0.5 McFarland turbidity standard, followed by a 20-fold dilution. TCMMs were initially dissolved in dimethyl sulfoxide (DMSO) and diluted with PBS into a series of solutions at different concentrations. CAMHB medium, bacterial suspension and TCMMs at different concentrations were added into 96-well cell culture plates in groups. The CV026 and XDRAB were incubated at 30°C and 37°C for 16–20 h, respectively. Less than OD600 of 0.1 were regarded as no bacterial growth, and colored drugs were observed macroscopically and data were recorded (Peng et al., 2020; Humphries et al., 2021).

### 2.4 Quorum sensing inhibition activity assay

CV026 is used as a biosensor strain to detect anti-QS activity. A culture-activated CV026 strain (5 mL) was added to 50 mL of LB nutrient agar medium containing kanamycin and C6-HSL (final concentration of 20 μg/mL), mixed and poured well, then punched and subinhibitory TCMM concentrations were added into the wells. After 24 h of incubation at 30°C, plates were observed for the inhibition of violacein. At the same time, the culture-activated CV026 bacterial solution was added to a 6-well cell culture plate, and then LB broth medium containing kanamycin, C6-HSL, and TCMM solutions with subinhibitory concentrations were added. 0.9% normal saline was used as the blank control, and furanone C30 was used as the positive control. After 24 h of incubation at 30°C, 1 mL of the bacterial solution from each well was centrifuged (13,000 g, 5 min) and DMSO was added to the cell pellets to dissolve violacein pigment. Supernatants (containing dissolved violacein) were transferred to a 96-well cell culture plate, and the absorbances were measured at 585 nm (Lou et al., 2019).

### 2.5 Growth curve test

Taking the 96-well cell culture plate, 170 μL TSB medium was added into each well, and then diluted bacterial solution and TCMMs solution with the different concentrations (1 MIC, 1/2 MIC, 1/4 MIC, and 1/8 MIC) were added. The value of OD_570_ was measured after incubation at 37°C every 6 h. The growth curves of XDRAB within 24 h were plotted, respectively. Concentrations that did not affect bacterial growth were finally selected as subsequent working concentrations (Aranda et al., 2011).

### 2.6 Effect on adhesion

Twelve activated XDRAB strains were diluted 1:1,000 and added into confocal dishes, then 1 mL of working concentration TCMMs were added to each well and incubated at 37°C for 4 h. After rinsing twice with PBS and sonicating for 10 min, PBS was added again to detach the bacteria adhering to the culture dish. 100 μL of detached bacteria were diluted 10-fold and spread on LB plates and cultured at 37°C for 24 h before counting. The relative value method (lg10 (N×10 × 1,000)) was used to assess the effect of TCMMs on XDRAB adhesion (Fan et al., 2018).

### 2.7 Effect on biofilm formation

We performed biofilm formation assays using crystal violet. Briefly, using the 96-well cell culture plate, 170 μL of TSB medium and 10 μL of PBS (control group) or TCMM solution at the working concentration were added into each well, followed by the inoculation of 20 μL of bacterial suspension (OD_600_ of 0.12). *Escherichia coli* DH5a was used as a negative control and six replicate wells were set for each strain. The plates were incubated at 37°C for 24 h, then the wells were voided and washed three times with PBS. After complete dryness, 0.1% crystal violet was added for staining for 20 min, then PBS was washed three times, dried in air for 30 min, dissolved in 95% ethanol for 15 min, and finally, the absorbance at 570 nm was measured (Farshadzadeh et al., 2018).

### 2.8 Effect on the expression of genes related to QS and virulence factors

The effect of TCMMs on XDRAB QS-regulated genes and virulence factor-related genes *abaR, abaI, csuE, bap,* and *pgaA* was investigated by quantitative reverse transcription-polymerase chain reaction (qRT-PCR), the gene primer sequences are presented in Table 1. The *16S rRNA* gene was used for the internal standard of mRNA quantification, and the expression levels of virulence factor genes were detected using the fluorescent dye SYBR Color qPCR Master Mix. According to the kit instructions, RNA was extracted and reverse transcribed into cDNA, followed by a 20 μL system used for the reaction. The cycling conditions of qRT-PCR were as follows: pre-denaturation at 95°C for 3 min, followed by 40 cycles of 95°C for 15 s and 60°C for 5 s. The expression level of each gene was normalized and the relative expression was calculated as 2^−ΔΔCT^. Fold changes in gene expression from TCMM-treated cells were compared to untreated cells that were propagated under the same conditions (Luo et al., 2015; Navidifar et al., 2022).

**TABLE 1:** Primer sequence.

Gene primers		Primer (5'→3′)
*16S rRNA*	F	CAG​CTC​GTG​TCG​TGA​GAT​GT
	R	CGT​AAG​GGC​CAT​GAT​GAC​TT
*abaI*	F	GAC​TGC​TAG​AGG​AAG​GCG​GAT​TTG
	R	AGA​CTA​CTA​CCC​ACC​ACA​CAA​CCC
*abaR*	F	TAA​ATG​TCG​GTT​GGG​CTC​AGT​CAA​G
	R	GCT​GGA​ATG​CAC​TGT​TTG​AGT​CAA​C
*csuE*	F	TCA​GAC​CGG​AGA​AAA​ACT​TAA​CG
	R	GCC​GGA​AGC​CGT​ATG​TAG​AA
*bap*	F	AAT​GCA​CCG​GTA​CTT​GAT​CC
	R	TAT​TGC​CTG​CAG​GGT​CAG​TT
*pgaA*	F	GCCGACGGTCGCGATAC
	R	ATG​CAC​ATC​ACC​AAA​ACG​GTA​CT

Genes *abaI*, *abaR*, and *16S rRNA* were designed and synthesized by Sangon Biotech (Shanghai) Co., Ltd. Genes *csuE*, *bap*, and *pgaA* were referred to the literature (Navidifar et al., 2022).

### 2.9 Statistical analysis

The mean and standard deviation of the mean were calculated using SPSS 26.0. GraphPad Prism 8.0 was used for mapping. The statistical analysis of the data was performed using the Student’s *t*-test to compare the treated group with the control group. Data was considered significant when *p* < 0.05.

## 3 Results

### 3.1 Anti-bacterial activity of TCMMs

The MIC of thirty-four TCMMs against strain CV026 ranged from 16 to 1,024 μg/mL (Table 2). Cinnamic aldehyde, carvacrol, and resveratrol showed obvious antibacterial activity, and the MICs were 32 μg/mL, 32 μg/mL and 16 μg/mL, respectively. The MIC of furanone C30 was 64 μg/mL. Twelve isolates of *A. baumannii* were susceptible to polymyxin B or tigecycline, but resistant to other antimicrobial agents, including aminoglycosides, carbapenems, cephalosporins, fluoroquinolones, and tetracyclines (Table 3). Such resistance profiles classified these isolates as XDRAB.

**TABLE 2:** MICs of 34 TCMMs against CV026.

TCMMs	MIC (μg/mL)	TCMMs	MIC (μg/mL)	TCMMs	MIC (μg/mL)
Caffeic acid	1,024	Naringenin	128	Myricetin	256
Berberine	256	Naringin	>1,024	Vanillin	1,024
Ferulic acid	1,024	Cinnamic acid	1,024	Hordenine	1,024
Baicalin	256	Matrine	1,024	Phloretin	128
Baicalein	128	Hesperidin	1,024	Capsaicin	512
Ursolic acid	128	Citral	256	Osthole	128
Linoleic acid	512	Cinnamic aldehyde	32	Nootkatone	128
Kaempferol	512	Carvacrol	32	Andrographolide	>1,024
4-Terpineol	512	Gallic acid	128	Parthenolide	512
Coumarin	512	Malic acid	1,024	Magnolol	64
Linalool	256	Resveratrol	16	Ellagic acid	256
Eugenol	64	Furanone C30	64		

TCMMs, traditional Chinese medicine monomers; CV026, *Chromobacterium violaceum* 026; MIC, minimal inhibitory concentration; Furanone C30, drug control.

**TABLE 3:** MICs of 16 antibacterial agents against 12 clinical isolates of *A. baumannii*.

Antibacterial agents	MIC (μg/mL)													
	AB1		AB2	AB3	AB4	AB5	AB6	AB7	AB8	AB9	AB10	AB11	AB12	
Ampicillin	>1,024		>1,024	>1,024	>1,024	>1,024	>1,024	>1,024	>1,024	>1,024	>1,024	>1,024	>1,024	
Piperacillin	>512		>512	>1,024	1,024	>512	512	256	>1,024	>1,024	512	1,024	>512	
Ceftazidime	64		64	64	64	128	>256	>256	>256	128	>256	>256	256	
Cefotaxime	128		128	128	128	256	>256	256	>256	128	>256	256	256	
Ampicillin/sulbactam	256		64	128	256	64	64	64	256	128	64	64	64	
Cefoperazone/sulbactam	128		64	128	64	64	64	64	256	64	64	64	64	
Amikacin	>256		>256	>256	>512	>256	>256	>256	>512	>256	>256	>256	>256	
Gentamicin	>128		>128	>256	>256	>128	>128	>256	>256	>256	>128	>128	>512	
Ciprofloxacin	>32		32	64	32	>32	16	128	64	256	32	>32	16	
Levofloxacin	8		8	8	8	16	8	16	8	16	8	8	16	
Tetracycline	>128		>128	128	256	>128	>128	128	>256	64	>128	>128	>128	
Doxycycline	64		64	64	64	32	32	32	32	32	32	64	32	
Meropenem	32		8	16	32	16	16	16	32	16	16	32	8	
Imipenem	32		16	16	32	32	16	16	32	16	32	16	16	
Tigecycline	2		2	1	2	1	1	1	1	1	1	2	1	
Polymyxin B	1		1	2	1	2	2	2	2	2	2	2	2	

### 3.2 Quorum sensing inhibition activity of TCMMs

Thirty-four TCMMs showed different effects on the QS activity of CV026 (Table 4). Twenty-one TCMMs, including coumarin, 4-terpineol, and hordenine, formed turbid inhibition circles on purple plates and showed different degrees of QS inhibition effects, which belonged to QS activity inhibitors. In contrast, the remaining thirteen TCMMs and blank controls did not form turbid or only transparent diaphragms, indicating no QS inhibitory activity. Thus, twenty-one of thirty-four TCMMs showed different degrees of QS inhibitory activity.

**TABLE 4:** Effect of 34 TCMMs on QS activity of CV026.

TCMMs	QS activity	TCMMs	QS activity	TCMMs	QS activity
4-Terpineol	+	Hordenine	+	Caffeic acid	+
Magnolol	+	Matrine	+	Ellagic acid	+
Cinnamic acid	+	Kaempferol	+	Coumarin	+
Carvacrol	+	Vanillin	+	Parthenolide	+
Myricetin	+	Naringenin	+	Baicalein	+
Nootkatone	+	Resveratrol	+	Eugenol	+
Phloretin	+	Osthole	+	Berberine	+
Hesperidin	—	Andrographolide	—	Malic acid	—
Linoleic Acid	—	Ferulic acid	—	Linalool	—
Baicalin	—	Capsaicin	—	Gallic acid	—
Citral	—	Cinnamic aldehyde	—	Naringin	—
Ursolic acid	—				

QS, Quorum sensing; +, QS inhibitory activity; —, No QS inhibitory activity.

**FIGURE 1:**
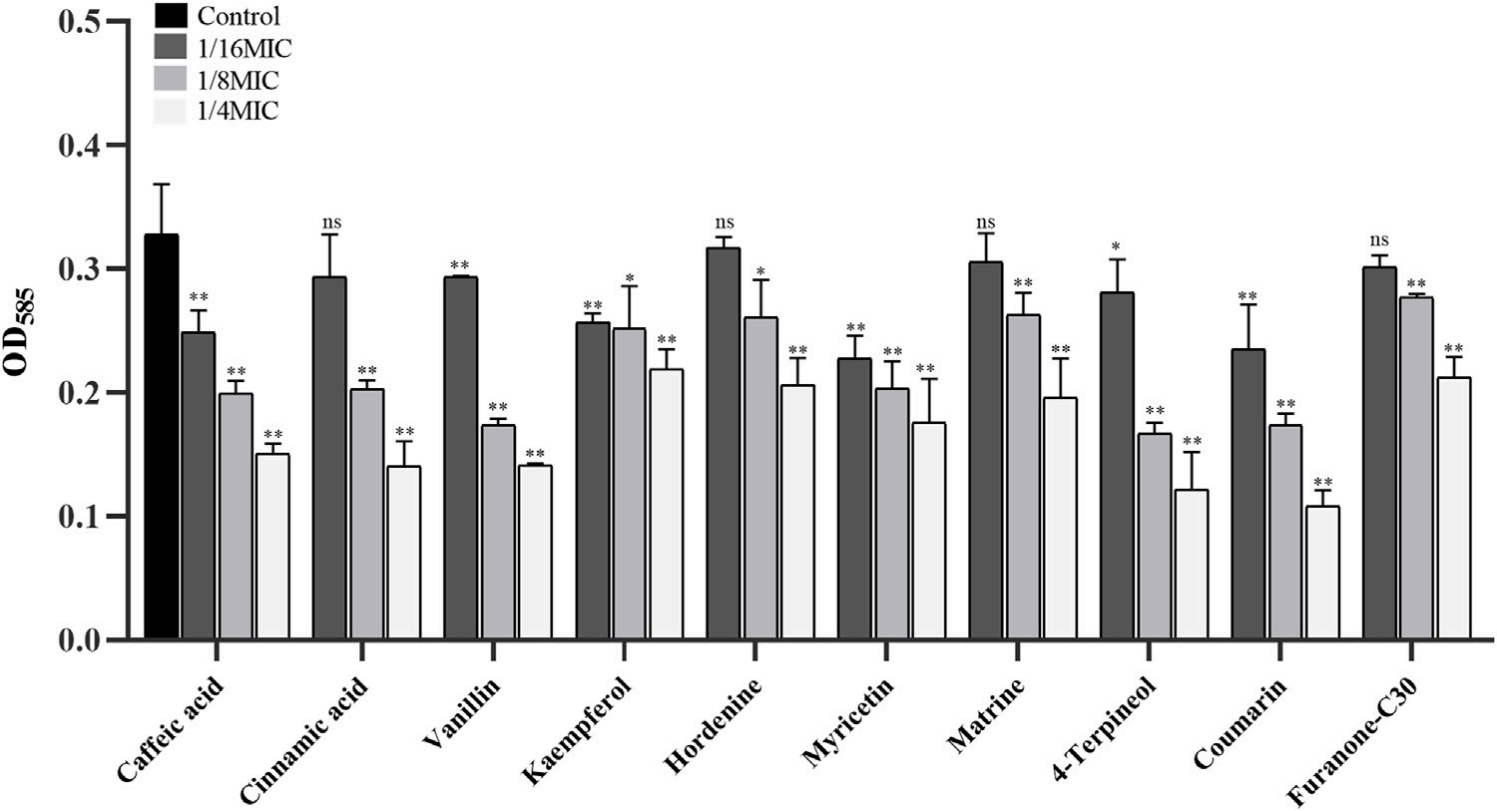
Effect of 9 TCMMs on the violacein production of CV026 at 1/4, 1/8 and 1/16MIC. TCMMs, traditional Chinese medicine monomers; MIC, minimal inhibitory concentration; CV026, *Chromobacterium violaceum* 026; Furanone C30, drug control; **, *p <* 0.01; *, *p* < 0.05*;* ns, *p* > 0.05.

Nine of twenty-one TCMMs could inhibit violacein production to varying degrees at sub-inhibitory concentrations and decrease in a concentration-dependent manner (Figure 1). Caffeic acid, vanillin, cinnamic acid, matrine, hordenine, kaempferol, coumarin, 4-terpineol, and myricetin showed relatively obvious QS inhibitory activity, with significant differences at the concentrations of both 1/4 MIC and 1/8 MIC (*p* < 0.05), and the inhibitory effects were better than furanone C30. In addition, caffeic acid, vanillin, kaempferol, myricetin, and coumarin also showed inhibitory effects at a concentration of 1/16 MIC (*p* < 0.05). Thus, nine TCMMs, including caffeic acid, vanillin, and cinnamic acid, have inhibitory effects on the QS system without affecting the normal growth of the strains, which can be regarded as potential QSIs.

### 3.3 Effect of TCMMs on the growth of XDRAB

**FIGURE 2:**
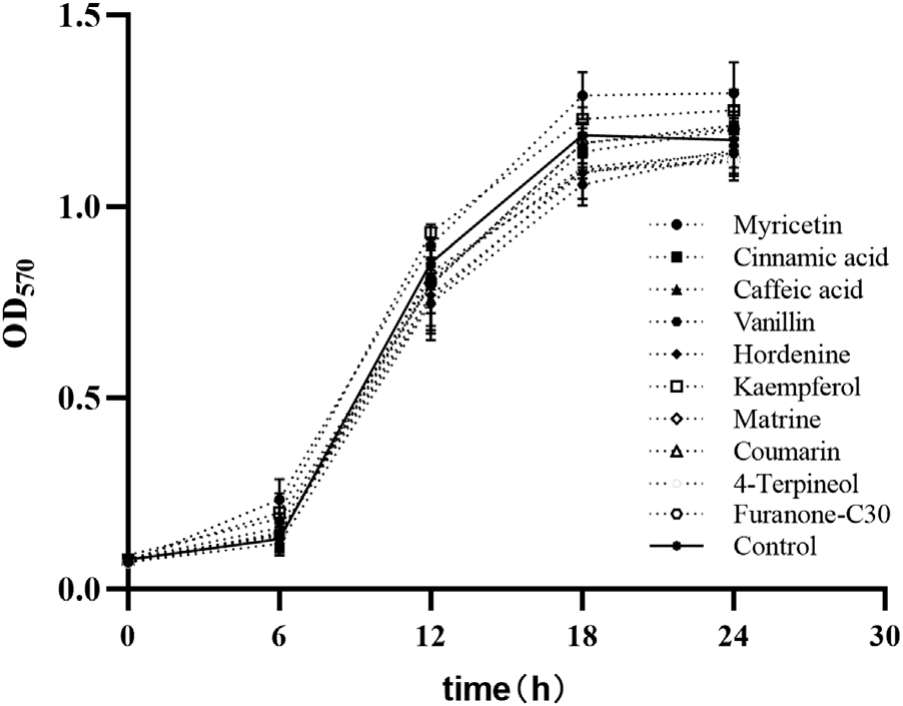
Bacterial growth curves of 12 XDRAB in the presence of 9 TCMMs at sub-MIC. Control, no TCMM; Myricetin (1/8 MIC = 32 μg/mL), cinnamic acid (1/8 MIC = 256 μg/mL), caffeic acid (1/8 MIC = 256 μg/mL), vanillin (1/8 MIC = 256 μg/mL), hordenine (1/8 MIC = 128 μg/mL), kaempferol (1/8 MIC = 64 μg/mL), matrine (1/8 MIC = 256 μg/mL), coumarin (1/8 MIC = 32 μg/mL), 4-terpineol (1/8 MIC = 64 μg/mL) and furanone C30 (1/8 MIC = 4 μg/mL). TCMMs, traditional Chinese medicine monomers; XDRAB, extensively drug-resistant *A. baumannii;* MIC, minimal inhibitory concentration.

The MICs of nine TCMMs against twelve XDRAB strains ranged from 256 to 2048 μg/mL. We found that myricetin, cinnamic acid, caffeic acid, vanillin, hordenine, kaempferol, matrine, coumarin, 4-terpineol, and furanone C30 did not affect the growth of bacterial cells at the 1/8 MIC concentration of 32, 256, 256, 256, 128, 64, 256, 32, 64, and 4 μg/mL, respectively. The growth curve of XDRAB by TCMMs is shown in Figure 2.

### 3.4 Effect of TCMMs on the adhesion of XDRAB

**FIGURE 3:**
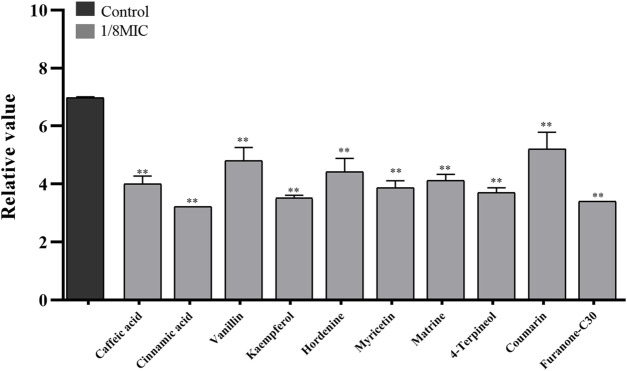
Effect of 9 TCMMs on the adhesion ability of XDRAB strains at 1/8 MIC (x ± s, *n* = 12). TCMMs, traditional Chinese medicine monomers; MIC, minimal inhibitory concentration; XDRAB, extensively drug-resistant *A. baumannii;* **, *p <* 0.01.

Nine TCMMs could inhibit the adhesion of twelve XDRAB strains to confocal dishes at a concentration of 1/8 MIC and significantly reduce the number of colonies adhering to the culture dishes (*p* < 0.01). After 1/8 MIC of kaempferol and 4-terpineol, the relative numbers of bacteria adhering to the culture dishes were 3.52 ± 0.09 and 3.70 ± 0.17, respectively, compared with 6.99 ± 0.02 in the blank control group (Figure 3).

### 3.5 Effect of TCMMs on the biofilm formation of XDRAB

Nine TCMMs could inhibit the biofilm formation of XDRAB to different extents. At a concentration of 1/8 MIC, 4-terpineol, coumarin, hordenine, caffeic acid, matrine, kaempferol, and vanillin could significantly inhibit the biofilm formation of XDRAB (*p* < 0.05). Myricetin, cinnamic acid, and furanone C30 had relatively weak inhibitory effects (*p* > 0.05). Overall, TCMMs showed stronger anti-biofilm effects compared with furanone C30 (Table 5).

**TABLE 5:** Effect of 9 TCMMs on the biofilm formation ability of XDRAB at 1/8 MIC (x ± s, *n* = 12).

TCMMs (1/8 MIC)	BF ability of XDRAB (n = 12)			Inhibition rate of BF (%)		*p*-value	
Control	0.58 ± 0.21						
Caffeic acid	0.39 ± 0.12			32.41		<0.01	
Cinnamic acid	0.51 ± 0.11			13.09		>0.05	
Vanillin	0.42 ± 0.10			27.60		<0.05	
Kaempferol	0.33 ± 0.11			42.68		<0.01	
Hordenine	0.36 ± 0.08			38.11		<0.01	
Myricetin	0.51 ± 0.14			12.40		>0.05	
Matrine	0.38 ± 0.10			35.04		<0.01	
4-Terpineol	0.31 ± 0.10			47.37		<0.01	
Coumarin	0.38 ± 0.07			34.15		<0.01	
Furanone C30	0.46 ± 0.13			21.69		>0.05	

BF, biofilm formation; XDRAB, extensively drug-resistant *A. baumannii;* Furanone C30 = drug control; *p*-value = TCMMs was compared with control.

### 3.6 Effect of TCMMs on the expression of XDRAB virulence factor-related genes

The expression levels of QS-regulated genes *abaI, abaR* and virulence factor-related genes *csuE, bap*, and *pgaA* in biofilm strongly positive strains were analyzed by qRT-PCR at a concentration of 1/8 MIC (Table 6). Compared with the control group, the expression levels of QS genes *abaI* and *abaR*, pilus regulatory gene *csuE*, and biofilm-related genes *bap* and *pgaA* were significantly decreased by the eight TCMMs at the concentration of 1/8 MIC, except coumarin, which showed no effect on the biofilm-related gene *bap*. Therefore, nine TCMMs downregulated the QS-related genes *abaI, abaR* and the virulence factor-associated genes *csuE, bap*, and *pgaA,* except coumarin, which upregulated the gene *bap*.

**TABLE 6:** Effect of 9 TCMMs on the virulence factor-related gene expression of XDRAB at 1/8 MIC (x ± s, *n* = 3).

Relative expression	*abaI*	*abaR*	*csuE*	*bap*	*pgaA*
(2^-△△CT^)					
Control	1.00 ± 0.04	1.00 ± 0.02	1.00 ± 0.04	1.00 ± 0.02	1.00 ± 0.01
Caffeic acid	0.19 ± 0.07	0.20 ± 0.09	0.10 ± 0.02	0.37 ± 0.04	0.10 ± 0.08
Vanillin	0.27 ± 0.07	0.23 ± 0.02	0.10 ± 0.03	0.47 ± 0.06	0.07 ± 0.02
Cinnamic acid	0.17 ± 0.04	0.19 ± 0.03	0.13 ± 0.02	0.48 ± 0.08	0.06 ± 0.02
Matrine	0.34 ± 0.04	0.31 ± 0.09	0.11 ± 0.03	0.63 ± 0.09	0.13 ± 0.03
Hordenine	0.28 ± 0.09	0.41 ± 0.19	0.10 ± 0.03	0.35 ± 0.12	0.11 ± 0.12
Myricetin	0.25 ± 0.04	0.30 ± 0.04	0.17 ± 0.07	0.31 ± 0.01	0.10 ± 0.08
Coumarin	0.50 ± 0.14	0.39 ± 0.07	0.19 ± 0.02	1.20 ± 0.10	0.12 ± 0.01
Kaempferol	0.14 ± 0.03	0.29 ± 0.11	0.21 ± 0.09	0.55 ± 0.09	0.09 ± 0.02
4-Terpineol	0.39 ± 0.14	0.32 ± 0.01	0.20 ± 0.08	0.61 ± 0.01	0.15 ± 0.06

## 4 Discussion

Biofilm formation in XDRAB is closely associated with bacterial virulence and resistance (Ayoub and Hammoudi, 2020). It has been found that biofilm formation is a dynamic process regulated by various factors, in which QS plays a crucial role (Carradori et al., 2020). QSI makes bacteria more susceptible to clearance by reducing biofilm formation, thus decreasing the virulence and drug resistance of pathogens (Warrier et al., 2021). Hence, QS is a critical target for the design of novel antimicrobial therapeutics. Some TCMMs have been shown to inhibit QS in addition to their antimicrobial activity (Lee et al., 2014; Chen et al., 2016; Zhao et al., 2020). In this study, the QS inhibitory activity of thirty-four TCMMs was investigated by biosensor CV026. The results revealed that twenty-one TCMMs showed different degrees of QS inhibitory activity, among which nine TCMMs, such as caffeic acid, cinnamic acid, and vanillin, had relatively obvious QS inhibitory activity. Similar to our findings, coumarin and caffeic acid have been found to interfere with the violacein production of CV026 (D’almeida et al., 2017; Pejin et al., 2015).

The effect of nine TCMMs on XDRAB adhesion and biofilm formation was investigated in this study. At the concentration of 1/8 MIC, all the nine TCMMs reduced the adhesion and biofilm formation of XDRAB: adhesion ability was inhibited in a range of 25.46%–53.79%; biofilm formation ability was inhibited in a range of 12.40%–47.37%. The QS system is an important way for information exchange between bacteria and involves different stages of bacterial biofilm formation (Ahmed et al., 2020). It has been reported that coumarin affects the production of violacein from CV026 at a concentration of 100 μg/mL, followed by inhibiting the formation of biofilm in *Pseudomonas aeruginosa* (D’almeida et al., 2017). Similarly, kaempferol, 4-terpineol and hordenine, also showed QS and biofilm inhibition activity against other common clinical pathogenic bacteria (Rekha et al., 2017; Zhou et al., 2018; Bose et al., 2020). The results depicted that the nine TCMMs represented by 4- terpineol could inhibit the adhesion and biofilm formation of XDRAB, and these drugs might have broad anti-virulence activity. Biofilm is a component of the XDRAB resistance mechanisms (Mirghani et al., 2022). So, the susceptibility of antimicrobial agents to bacteria can be increased by reducing biofilm formation. These results suggested that the nine TCMMs could be used as antibacterial adjuvants to increase the bactericidal effect of antibacterial drugs. Alves also found linalool to be a QSI that reduced the expression of *A. baumannii* virulence factors (Alves et al., 2016). Therefore, QS is closely related to bacterial virulence, which is consistent with our research results.

We also used qRT-PCR to investigate the effect of nine TCMMs on virulence factor-related gene expression of XDRAB with strong biofilm formation ability. It has been shown that QS genes are critical regulators of biofilm formation, and various virulence factors (Elnegery et al., 2021). QSI inhibits the expression of virulence factor-related genes in pathogens by down-regulating QS key genes (Wang et al., 2021). The results indicated that the nine TCMMs downregulated QS-related genes *abaI, abaR* and virulence factor-related genes *csuE, bap*, and *pgaA* except coumarin, which upregulated the *bap* gene. The *abaI* expression was reduced by 80.97%, 83.18%, 73.30%, and 86.22% in cultures treated with 1/8 MICs of caffeic acid, cinnamic acid, vanillin, and kaempferol, respectively. In addition, treatment with myricetin and 4-terpineol revealed a significant decrease in the expression of *pgaA* and *bap* by 90.10%, 84.94%, and 68.77%, 39.07%, respectively. However, coumarin upregulated the expression of *bap* by 19.80%. Therefore, by down-regulating QS genes *abaI* and *abaR*, the nine TCMMs affected the expression of *csuE*, which prevented bacteria from transforming from plankton to biofilm (de Breij et al., 2009), and inhibited the expression of *bap* and *pgaA*, which reduced bacterial adhesion and destroyed biofilm formation, followed by reducing bacterial virulence (Choi et al., 2009; Farshadzadeh et al., 2018).

In conclusion, nine TCMMs like caffeic acid, cinnamic acid, and vanillin showed QS inhibitory activity. They might interfere with the QS system and inhibit the expression of XDRAB virulence factors by inhibiting QS and virulence factor-related genes, thus reversing bacterial drug resistance and reducing pathogenicity. This could develop as novel anti-virulence compounds to control XDRAB infections.

## Data Availability

The original contributions presented in the study are included in the article/Supplementary Materials, further inquiries can be directed to the corresponding author.
