# Supplementary figures and images for: Effect of traditional Chinese medicine monomers interfering with quorum-sensing on virulence factors of extensively drug-resistant Acinetobacter baumannii (part 2 of 2)
Source: Front Pharmacol. 2023 Mar 30;14:1135180. doi: 10.3389/fphar.2023.1135180 (PMC10097947; doi:10.3389/fphar.2023.1135180)

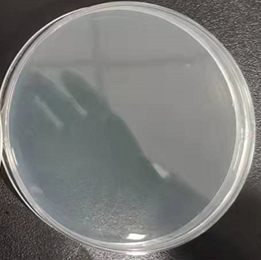

Supplement: Supplementary file 1 [file DataSheet1.zip › Data Sheet 1/Effect of 9 TCMMs on the adhesion ability of XDRAB/adhesion data/Kaempferol/0 (3).jpg]

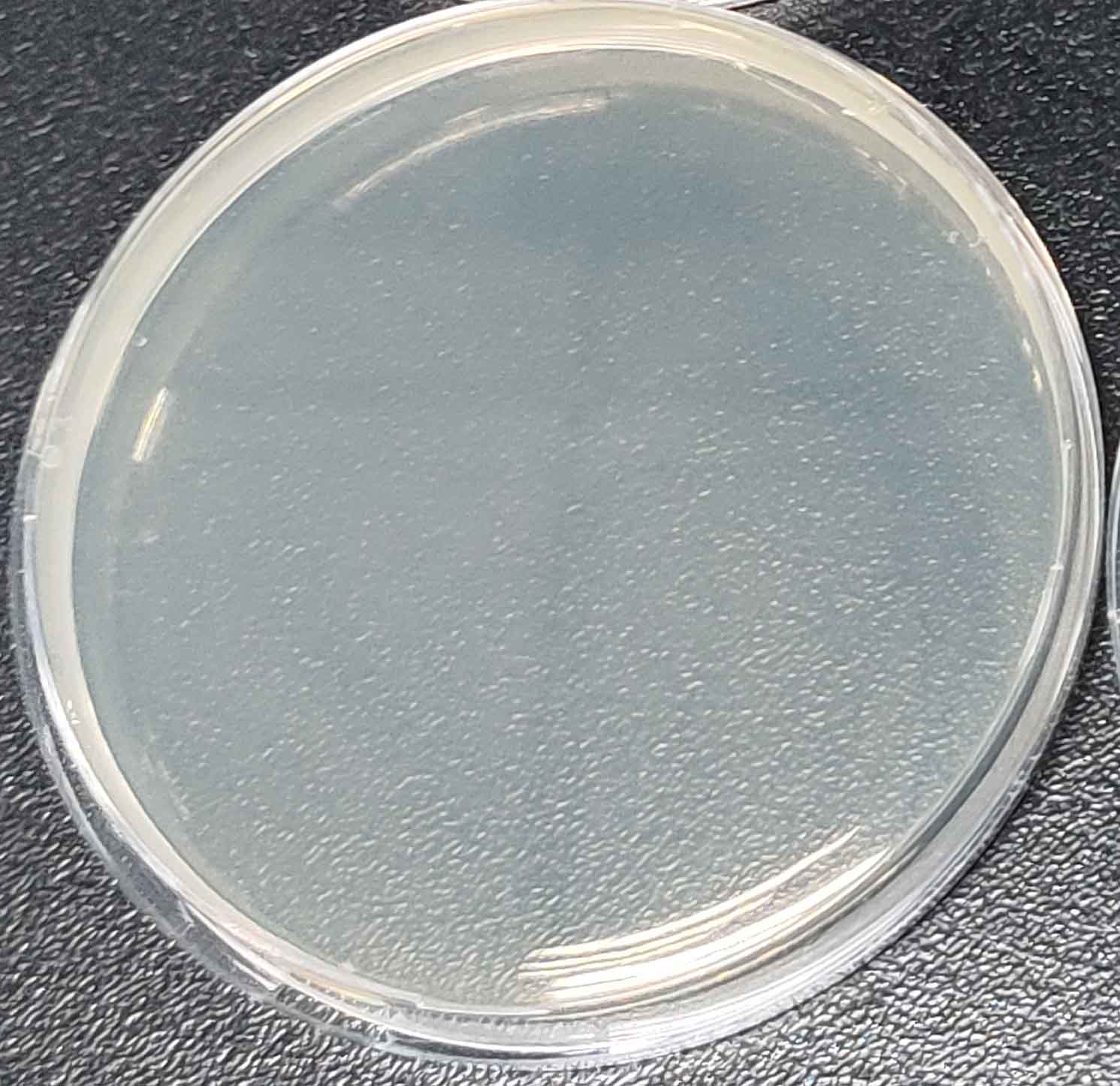

Supplement: Supplementary file 1 [file DataSheet1.zip › Data Sheet 1/Effect of 9 TCMMs on the adhesion ability of XDRAB/adhesion data/Kaempferol/0 (4).jpg]

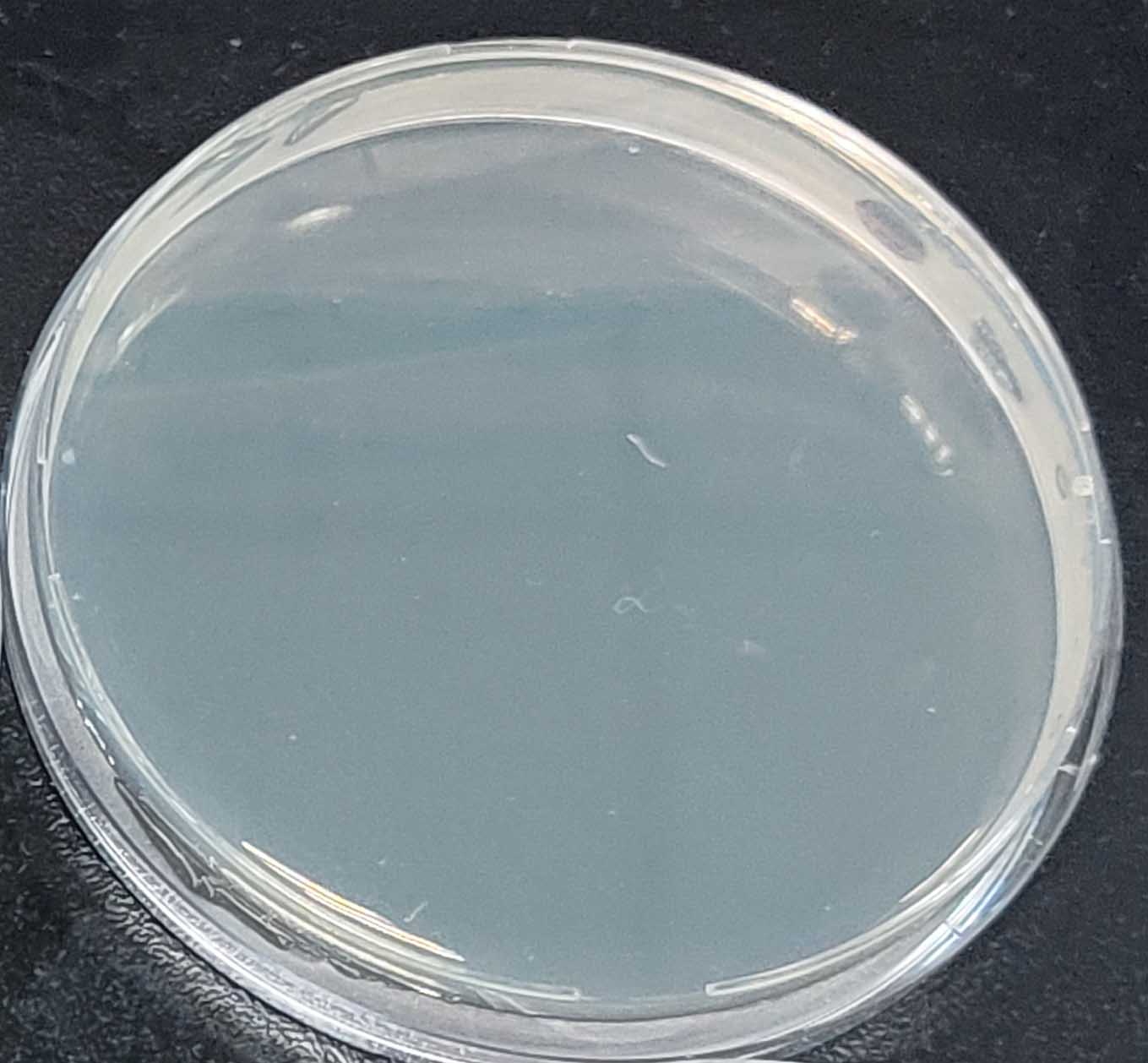

Supplement: Supplementary file 1 [file DataSheet1.zip › Data Sheet 1/Effect of 9 TCMMs on the adhesion ability of XDRAB/adhesion data/Kaempferol/0 (5).jpg]

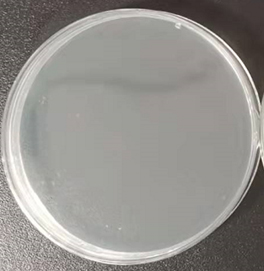

Supplement: Supplementary file 1 [file DataSheet1.zip › Data Sheet 1/Effect of 9 TCMMs on the adhesion ability of XDRAB/adhesion data/Kaempferol/0 (6).jpg]

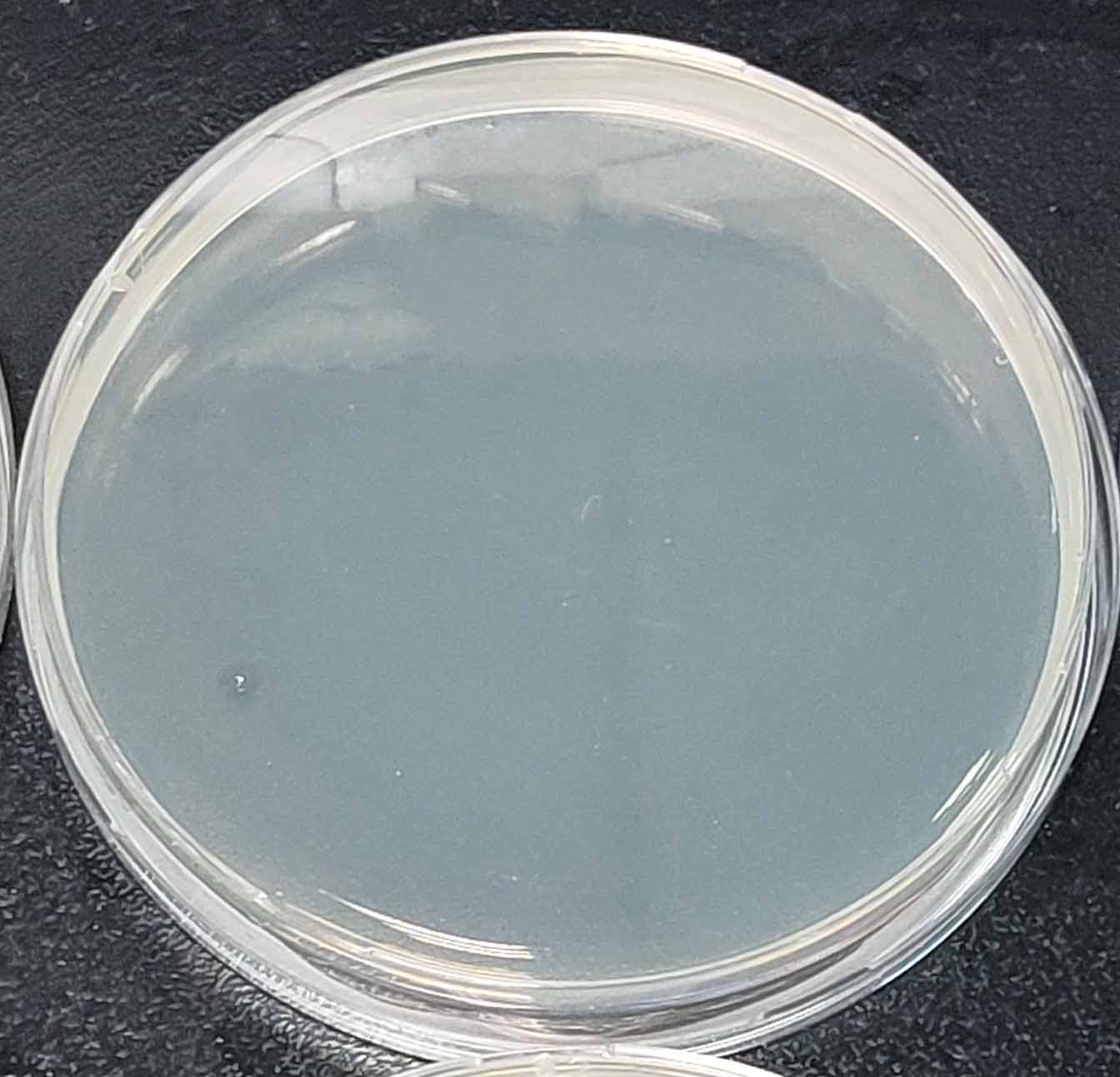

Supplement: Supplementary file 1 [file DataSheet1.zip › Data Sheet 1/Effect of 9 TCMMs on the adhesion ability of XDRAB/adhesion data/Kaempferol/0 (7).jpg]

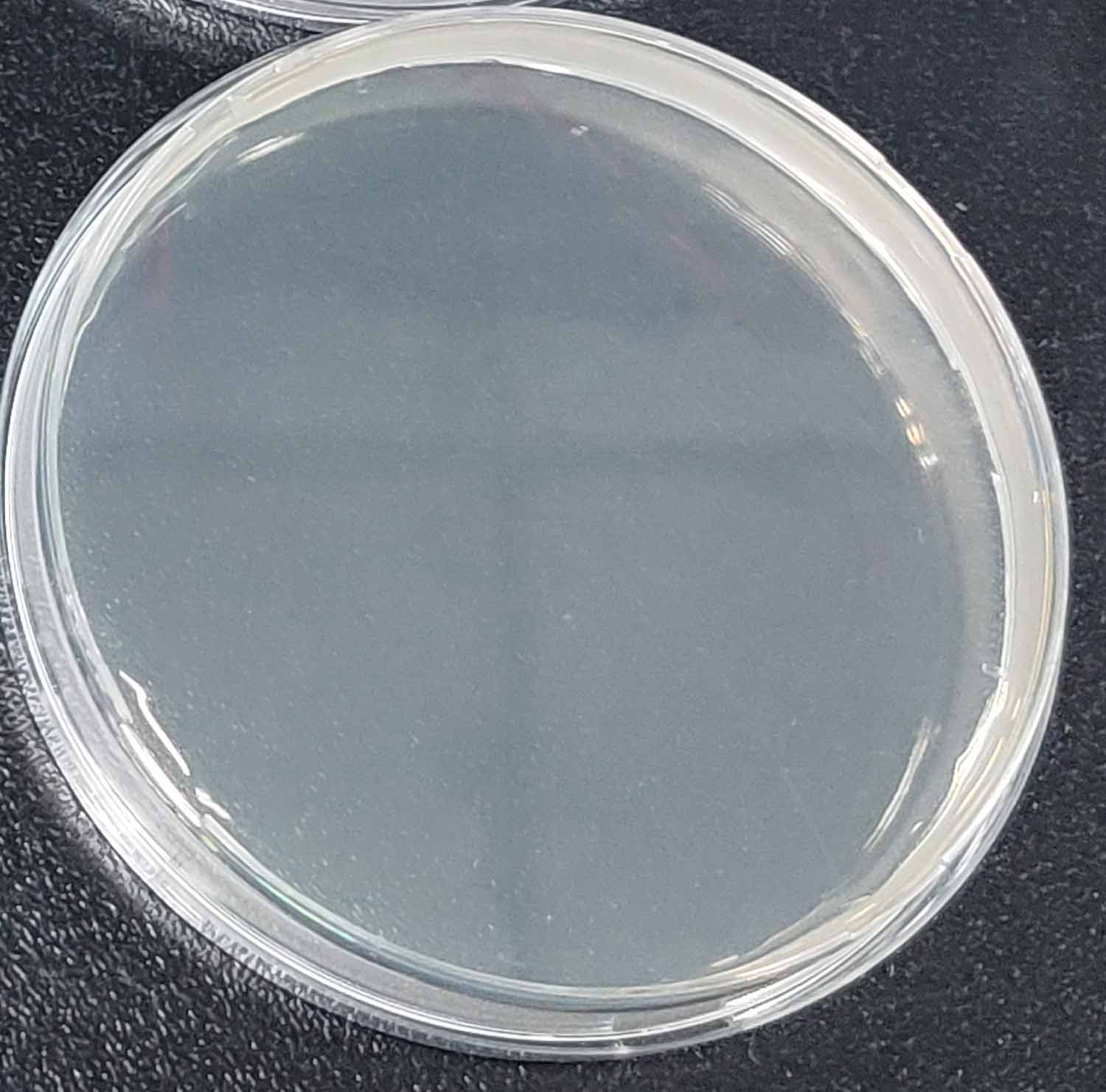

Supplement: Supplementary file 1 [file DataSheet1.zip › Data Sheet 1/Effect of 9 TCMMs on the adhesion ability of XDRAB/adhesion data/Kaempferol/0 (8).jpg]

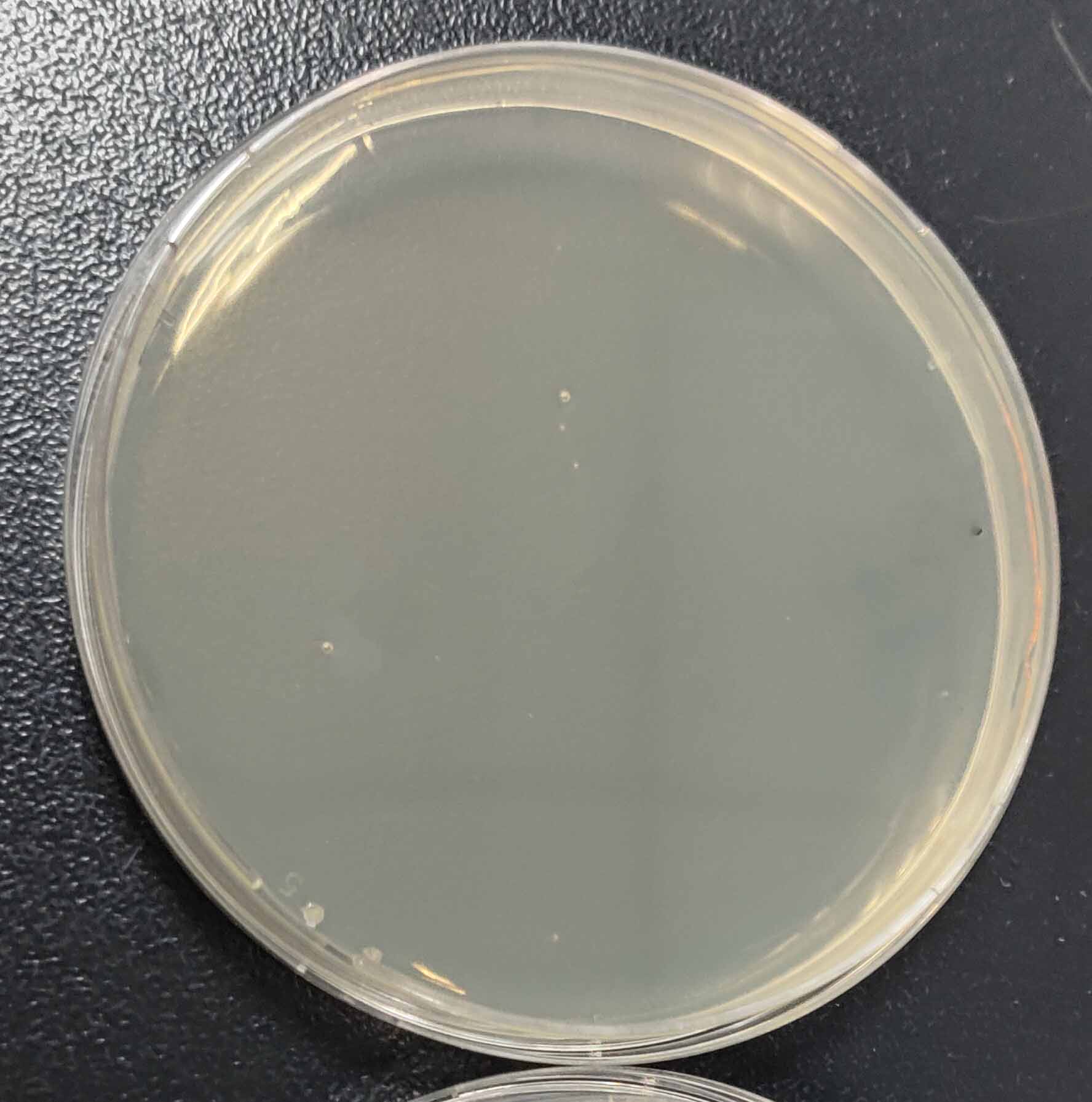

Supplement: Supplementary file 1 [file DataSheet1.zip › Data Sheet 1/Effect of 9 TCMMs on the adhesion ability of XDRAB/adhesion data/Kaempferol/0 (9).jpg]

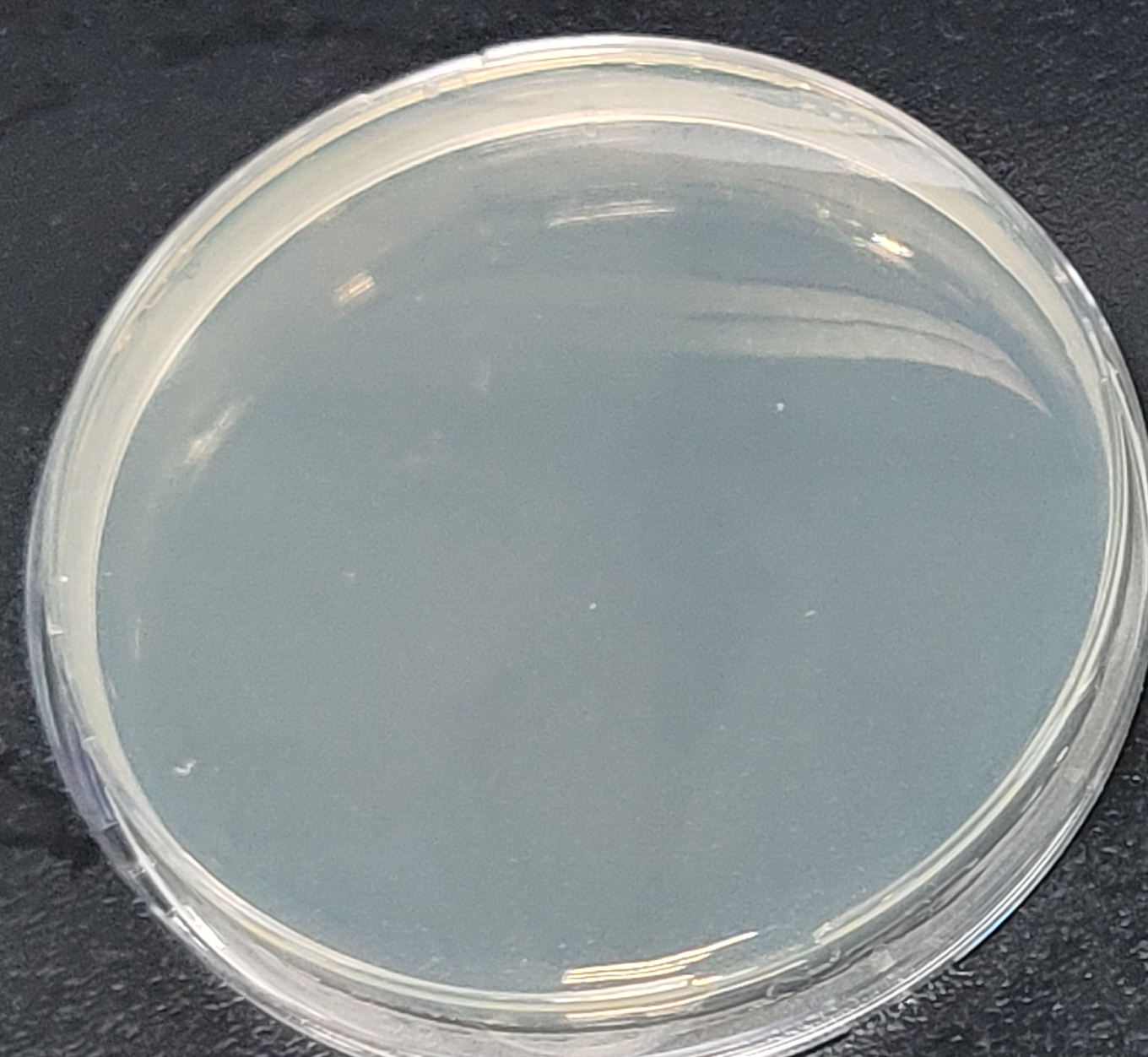

Supplement: Supplementary file 1 [file DataSheet1.zip › Data Sheet 1/Effect of 9 TCMMs on the adhesion ability of XDRAB/adhesion data/Kaempferol/0.jpg]

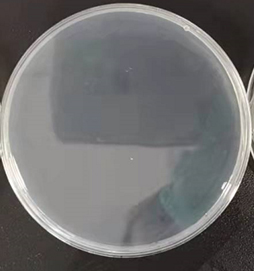

Supplement: Supplementary file 1 [file DataSheet1.zip › Data Sheet 1/Effect of 9 TCMMs on the adhesion ability of XDRAB/adhesion data/Kaempferol/1 (2).jpg]

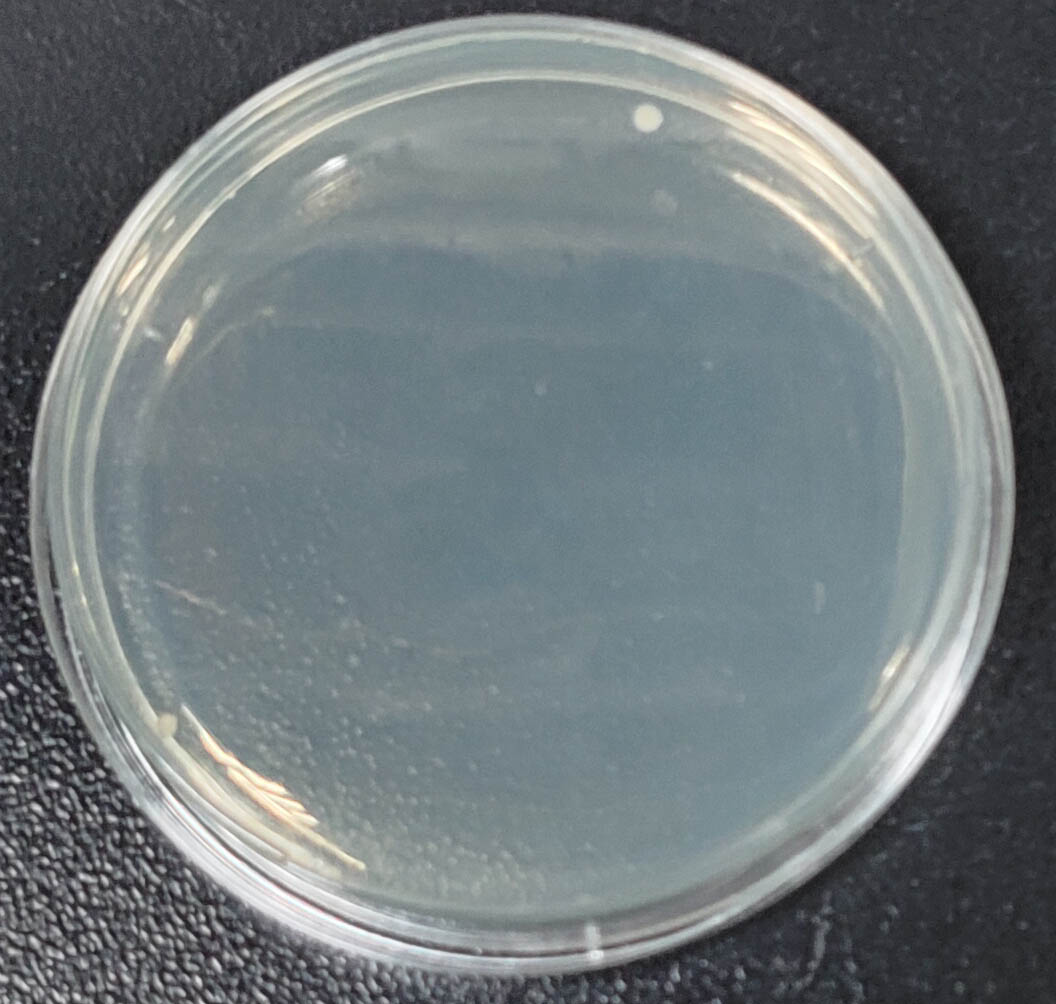

Supplement: Supplementary file 1 [file DataSheet1.zip › Data Sheet 1/Effect of 9 TCMMs on the adhesion ability of XDRAB/adhesion data/Kaempferol/1.jpg]

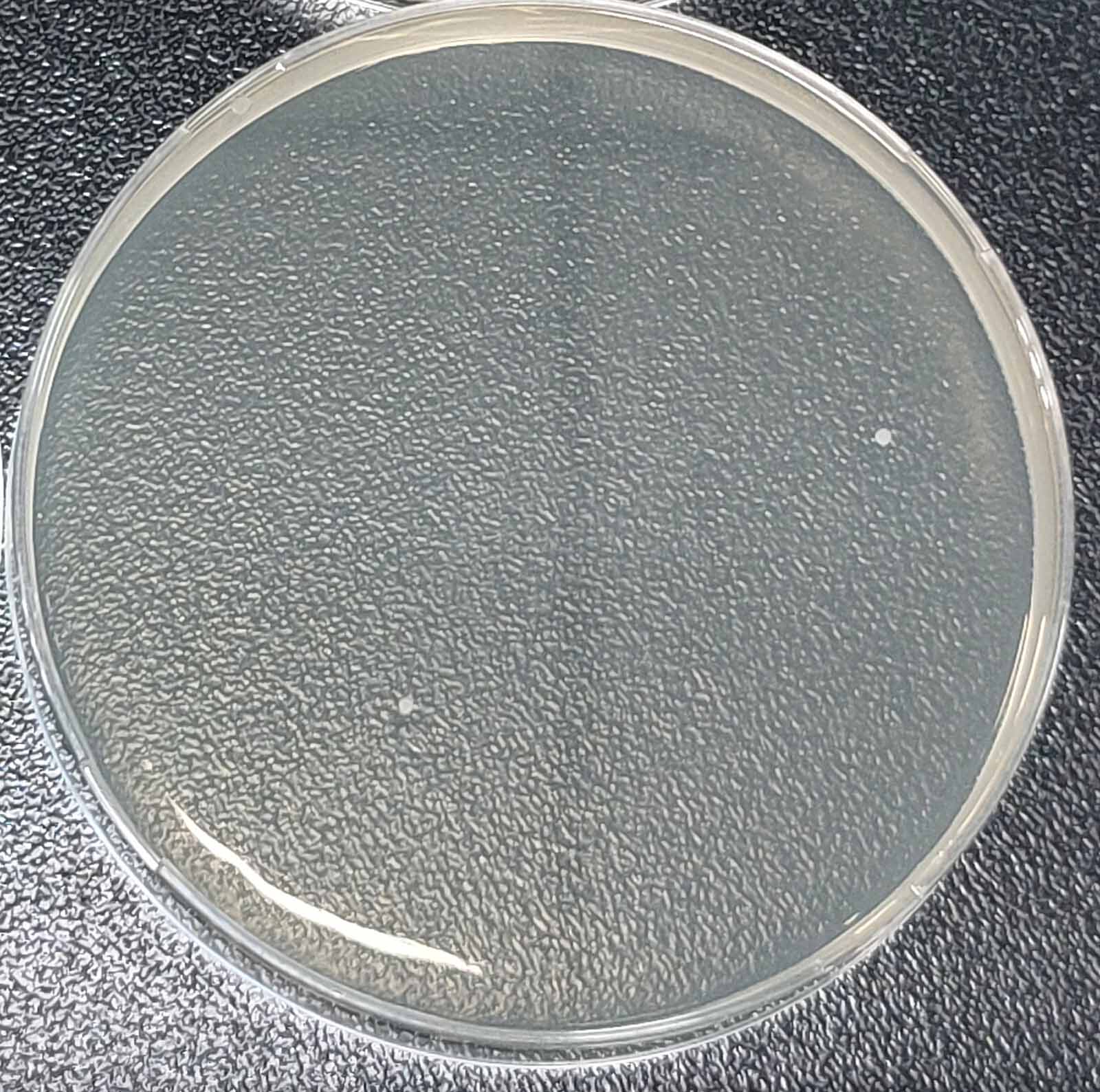

Supplement: Supplementary file 1 [file DataSheet1.zip › Data Sheet 1/Effect of 9 TCMMs on the adhesion ability of XDRAB/adhesion data/Kaempferol/2.jpg]

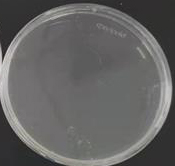

Supplement: Supplementary file 1 [file DataSheet1.zip › Data Sheet 1/Effect of 9 TCMMs on the adhesion ability of XDRAB/adhesion data/Matrine/0 (2).jpg]

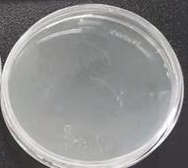

Supplement: Supplementary file 1 [file DataSheet1.zip › Data Sheet 1/Effect of 9 TCMMs on the adhesion ability of XDRAB/adhesion data/Matrine/0 (3).jpg]

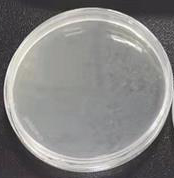

Supplement: Supplementary file 1 [file DataSheet1.zip › Data Sheet 1/Effect of 9 TCMMs on the adhesion ability of XDRAB/adhesion data/Matrine/0 (4).jpg]

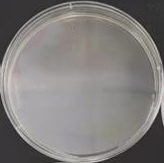

Supplement: Supplementary file 1 [file DataSheet1.zip › Data Sheet 1/Effect of 9 TCMMs on the adhesion ability of XDRAB/adhesion data/Matrine/0.jpg]

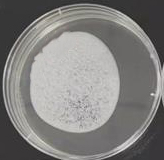

Supplement: Supplementary file 1 [file DataSheet1.zip › Data Sheet 1/Effect of 9 TCMMs on the adhesion ability of XDRAB/adhesion data/Matrine/1 (2).jpg]

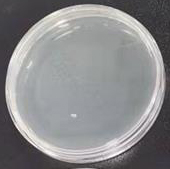

Supplement: Supplementary file 1 [file DataSheet1.zip › Data Sheet 1/Effect of 9 TCMMs on the adhesion ability of XDRAB/adhesion data/Matrine/1 (3).jpg]

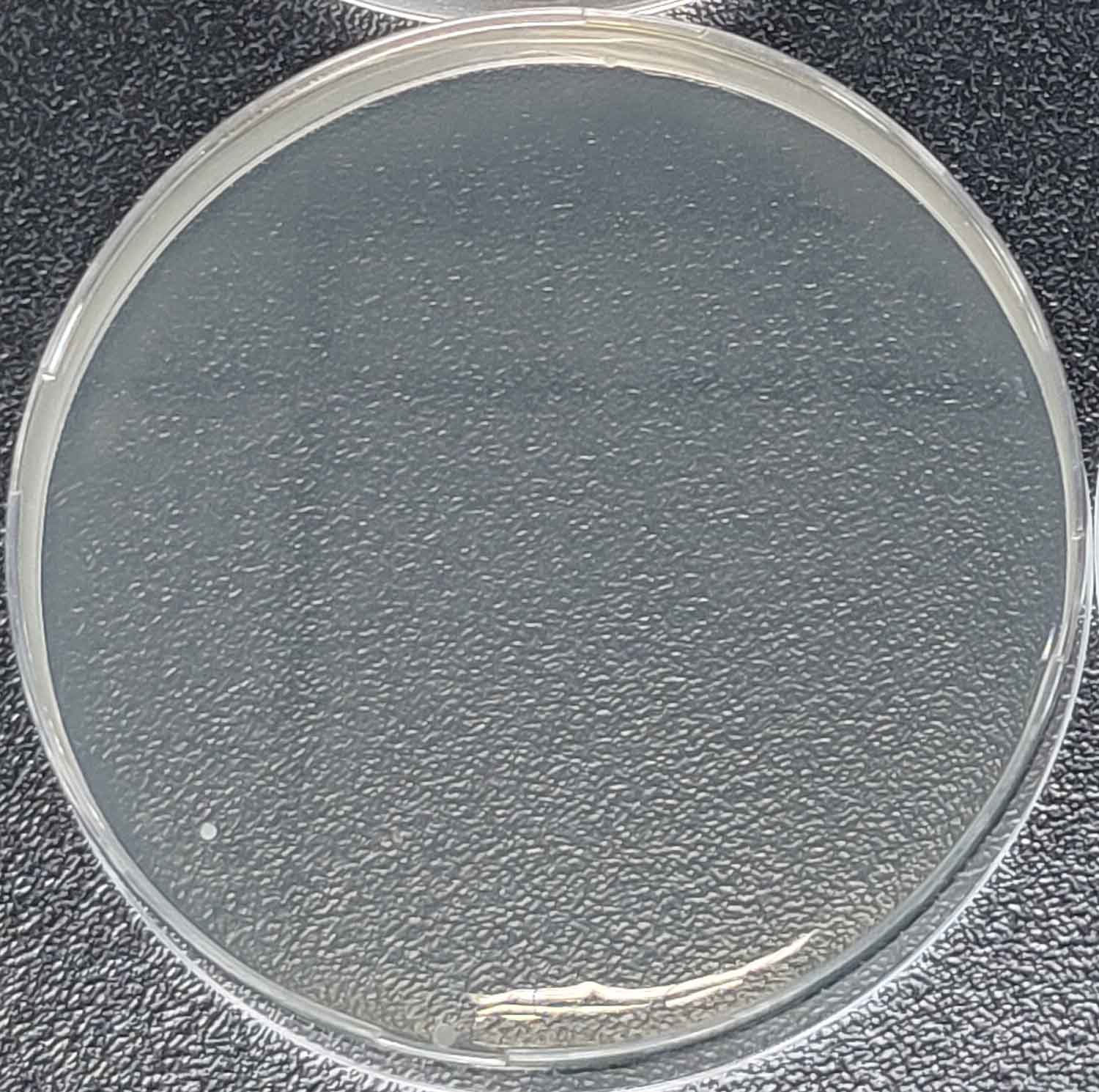

Supplement: Supplementary file 1 [file DataSheet1.zip › Data Sheet 1/Effect of 9 TCMMs on the adhesion ability of XDRAB/adhesion data/Matrine/1.jpg]

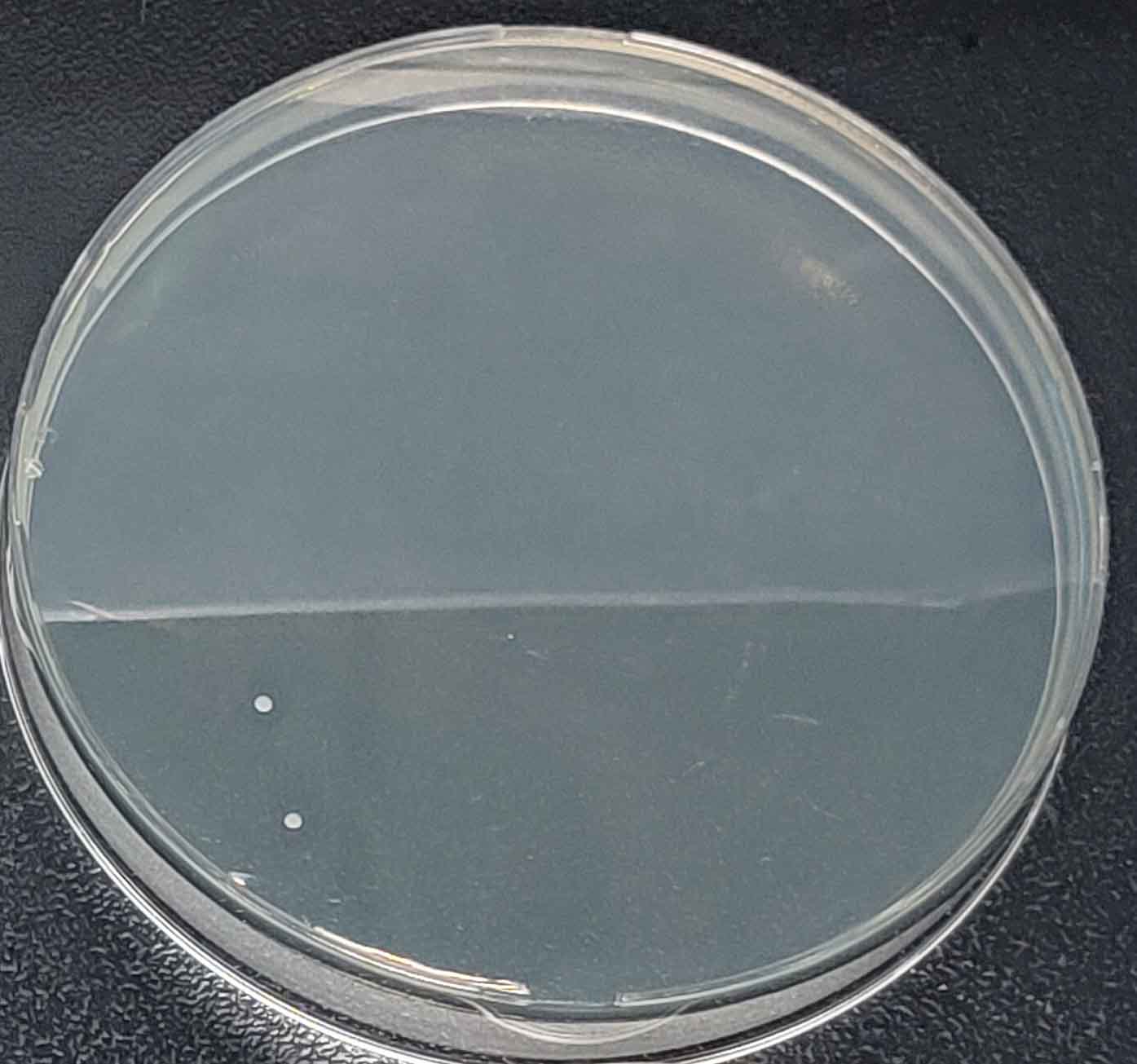

Supplement: Supplementary file 1 [file DataSheet1.zip › Data Sheet 1/Effect of 9 TCMMs on the adhesion ability of XDRAB/adhesion data/Matrine/2 (2).jpg]

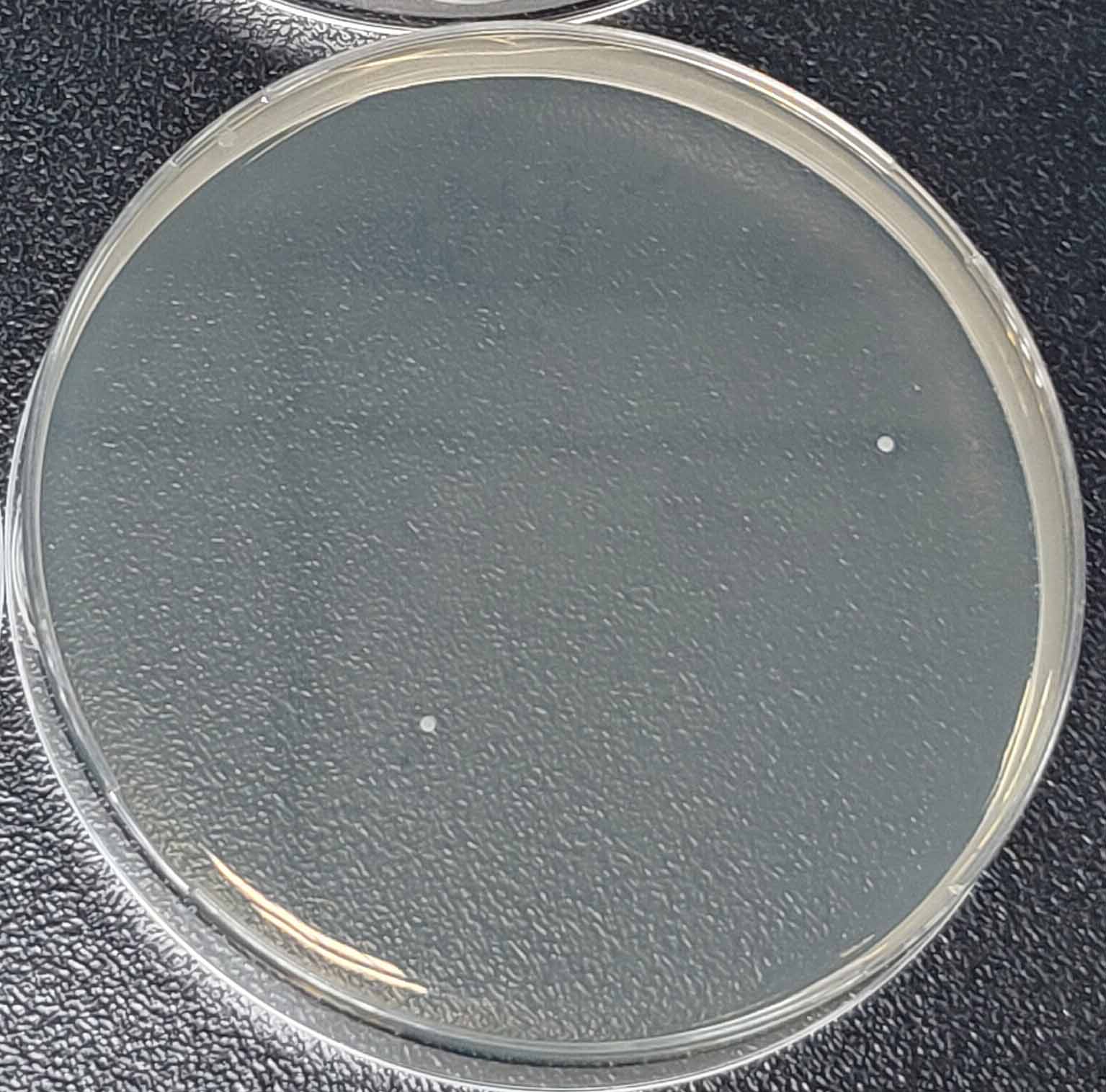

Supplement: Supplementary file 1 [file DataSheet1.zip › Data Sheet 1/Effect of 9 TCMMs on the adhesion ability of XDRAB/adhesion data/Matrine/2 (3).jpg]

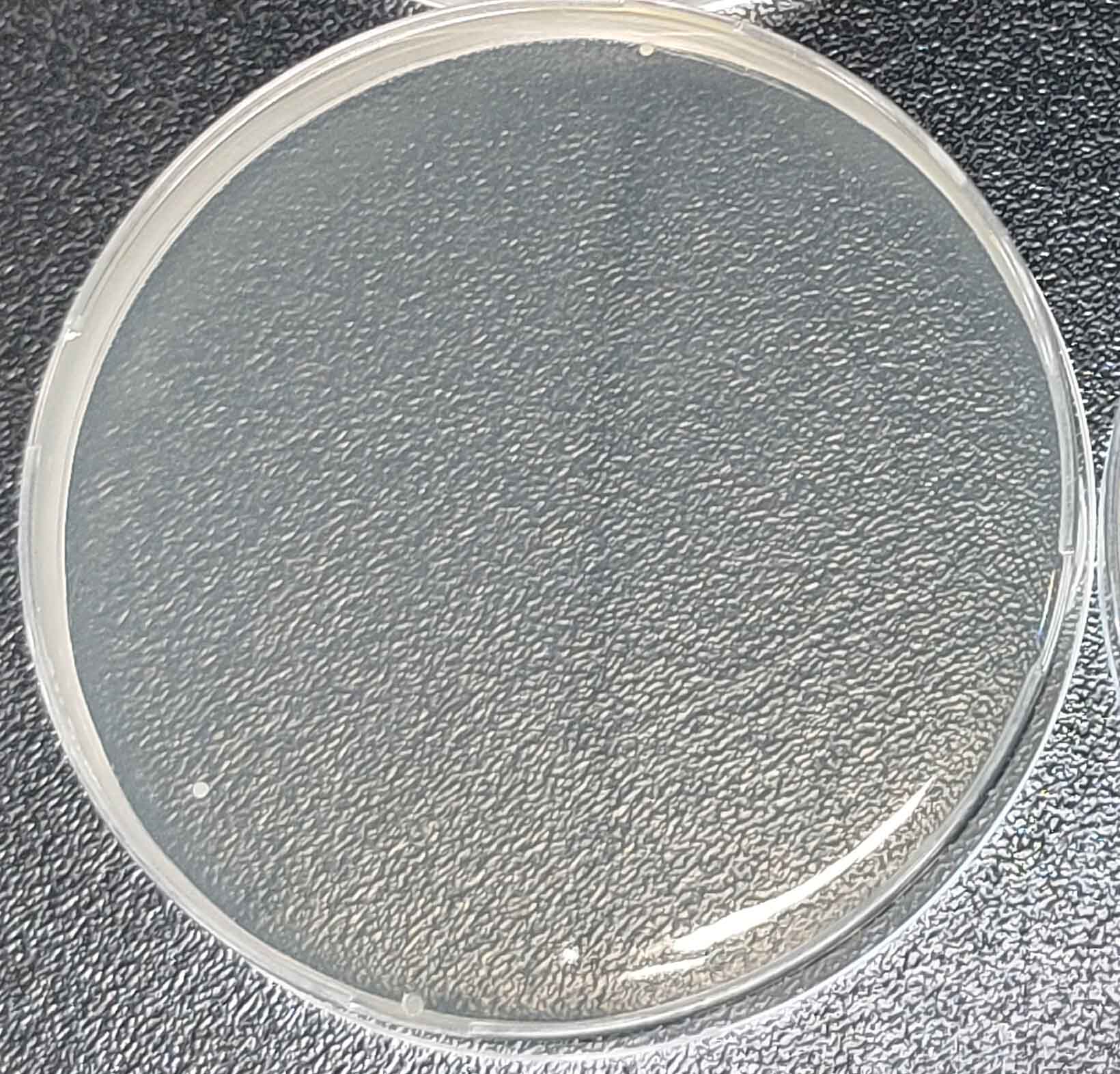

Supplement: Supplementary file 1 [file DataSheet1.zip › Data Sheet 1/Effect of 9 TCMMs on the adhesion ability of XDRAB/adhesion data/Matrine/2.jpg]

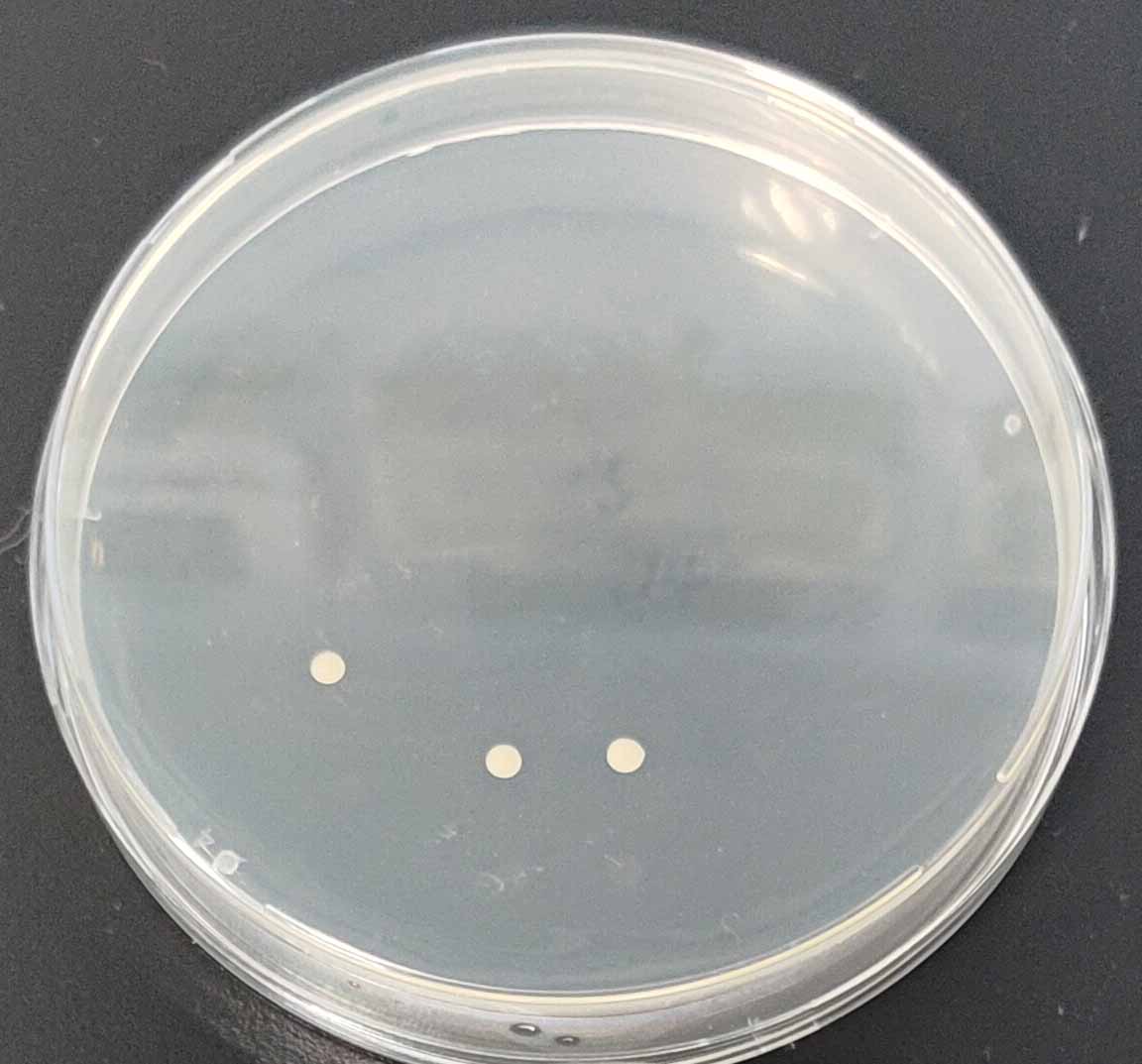

Supplement: Supplementary file 1 [file DataSheet1.zip › Data Sheet 1/Effect of 9 TCMMs on the adhesion ability of XDRAB/adhesion data/Matrine/3.jpg]

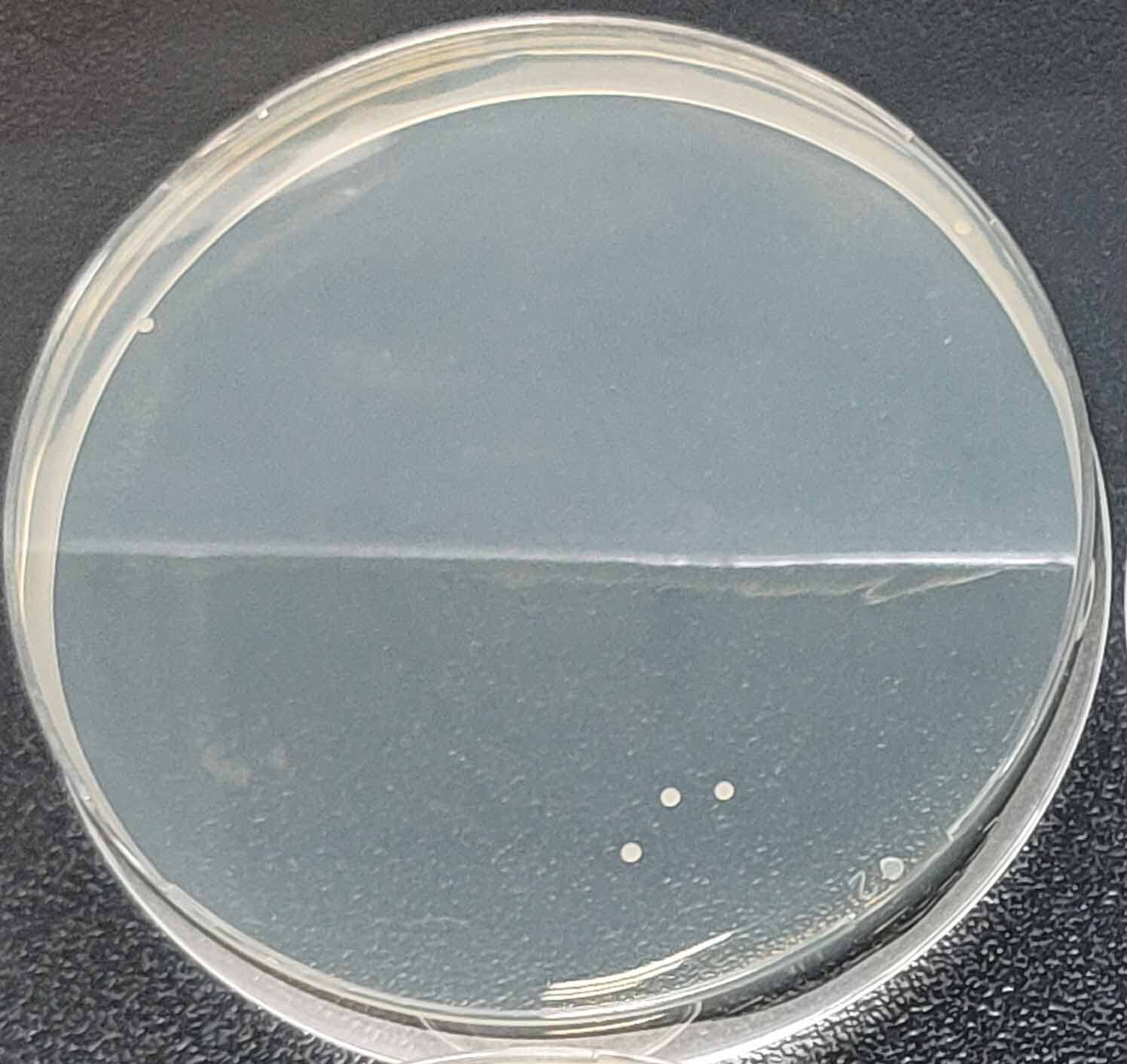

Supplement: Supplementary file 1 [file DataSheet1.zip › Data Sheet 1/Effect of 9 TCMMs on the adhesion ability of XDRAB/adhesion data/Matrine/4.jpg]

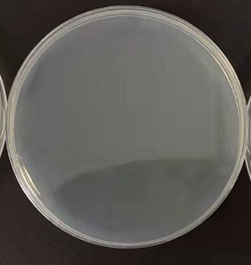

Supplement: Supplementary file 1 [file DataSheet1.zip › Data Sheet 1/Effect of 9 TCMMs on the adhesion ability of XDRAB/adhesion data/Myricetin/0 (2).jpg]

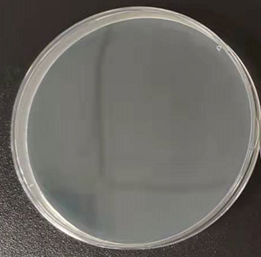

Supplement: Supplementary file 1 [file DataSheet1.zip › Data Sheet 1/Effect of 9 TCMMs on the adhesion ability of XDRAB/adhesion data/Myricetin/0 (3).jpg]

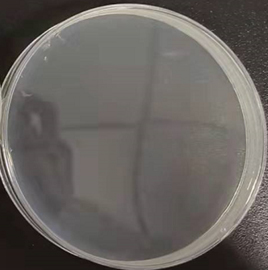

Supplement: Supplementary file 1 [file DataSheet1.zip › Data Sheet 1/Effect of 9 TCMMs on the adhesion ability of XDRAB/adhesion data/Myricetin/0 (4).jpg]

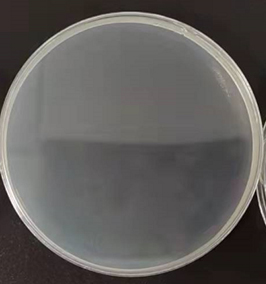

Supplement: Supplementary file 1 [file DataSheet1.zip › Data Sheet 1/Effect of 9 TCMMs on the adhesion ability of XDRAB/adhesion data/Myricetin/0 (5).jpg]

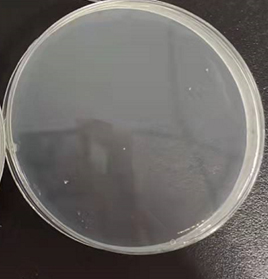

Supplement: Supplementary file 1 [file DataSheet1.zip › Data Sheet 1/Effect of 9 TCMMs on the adhesion ability of XDRAB/adhesion data/Myricetin/0 (6).jpg]

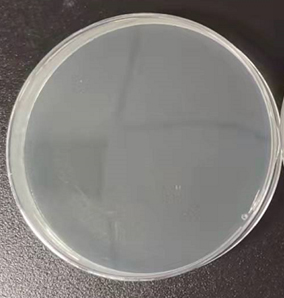

Supplement: Supplementary file 1 [file DataSheet1.zip › Data Sheet 1/Effect of 9 TCMMs on the adhesion ability of XDRAB/adhesion data/Myricetin/0 (7).jpg]

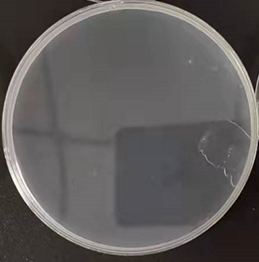

Supplement: Supplementary file 1 [file DataSheet1.zip › Data Sheet 1/Effect of 9 TCMMs on the adhesion ability of XDRAB/adhesion data/Myricetin/0 (8).jpg]

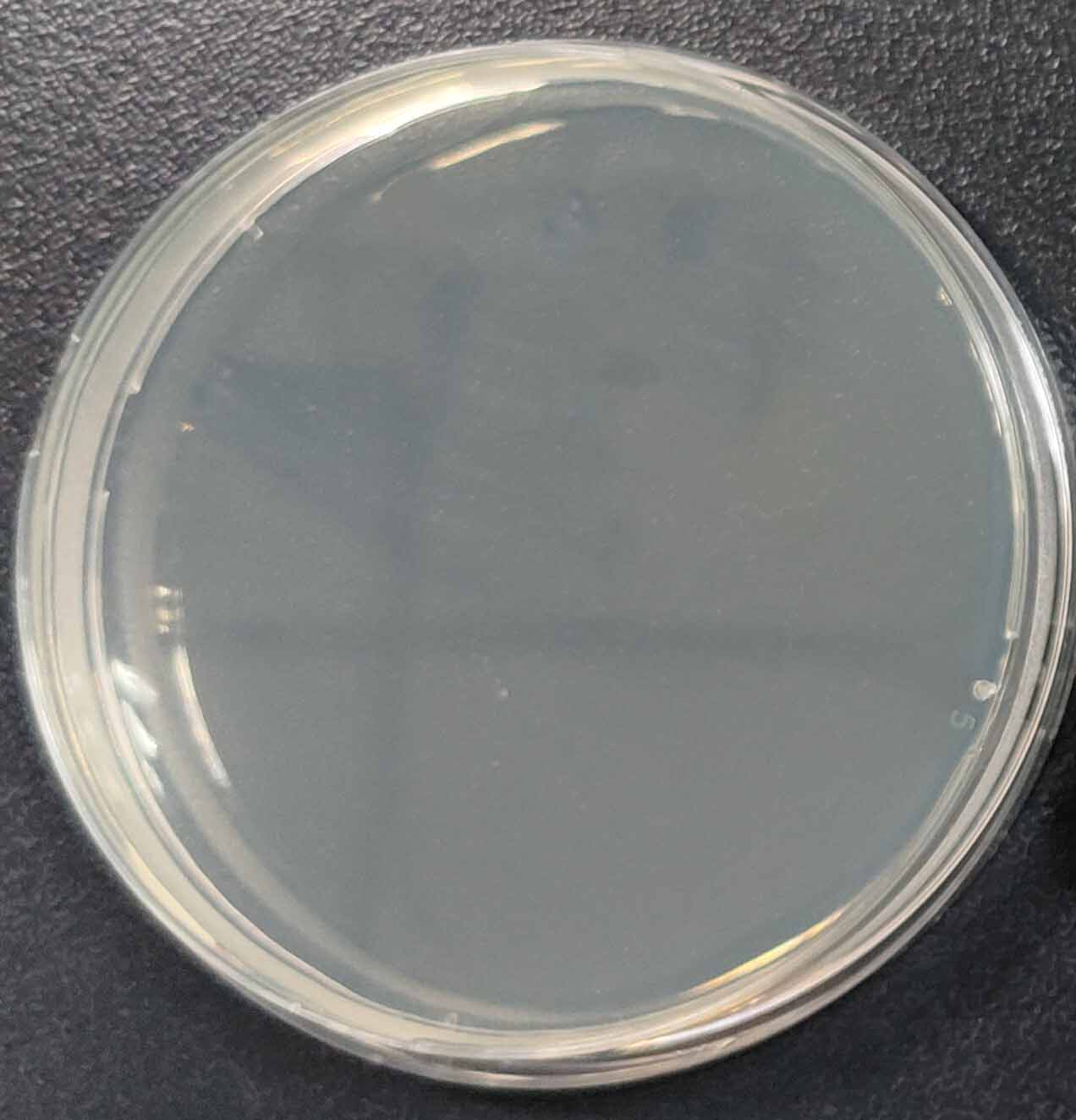

Supplement: Supplementary file 1 [file DataSheet1.zip › Data Sheet 1/Effect of 9 TCMMs on the adhesion ability of XDRAB/adhesion data/Myricetin/0 (9).jpg]

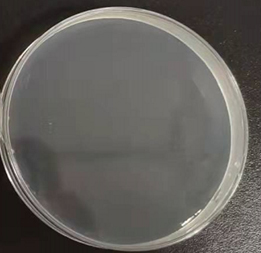

Supplement: Supplementary file 1 [file DataSheet1.zip › Data Sheet 1/Effect of 9 TCMMs on the adhesion ability of XDRAB/adhesion data/Myricetin/0.jpg]

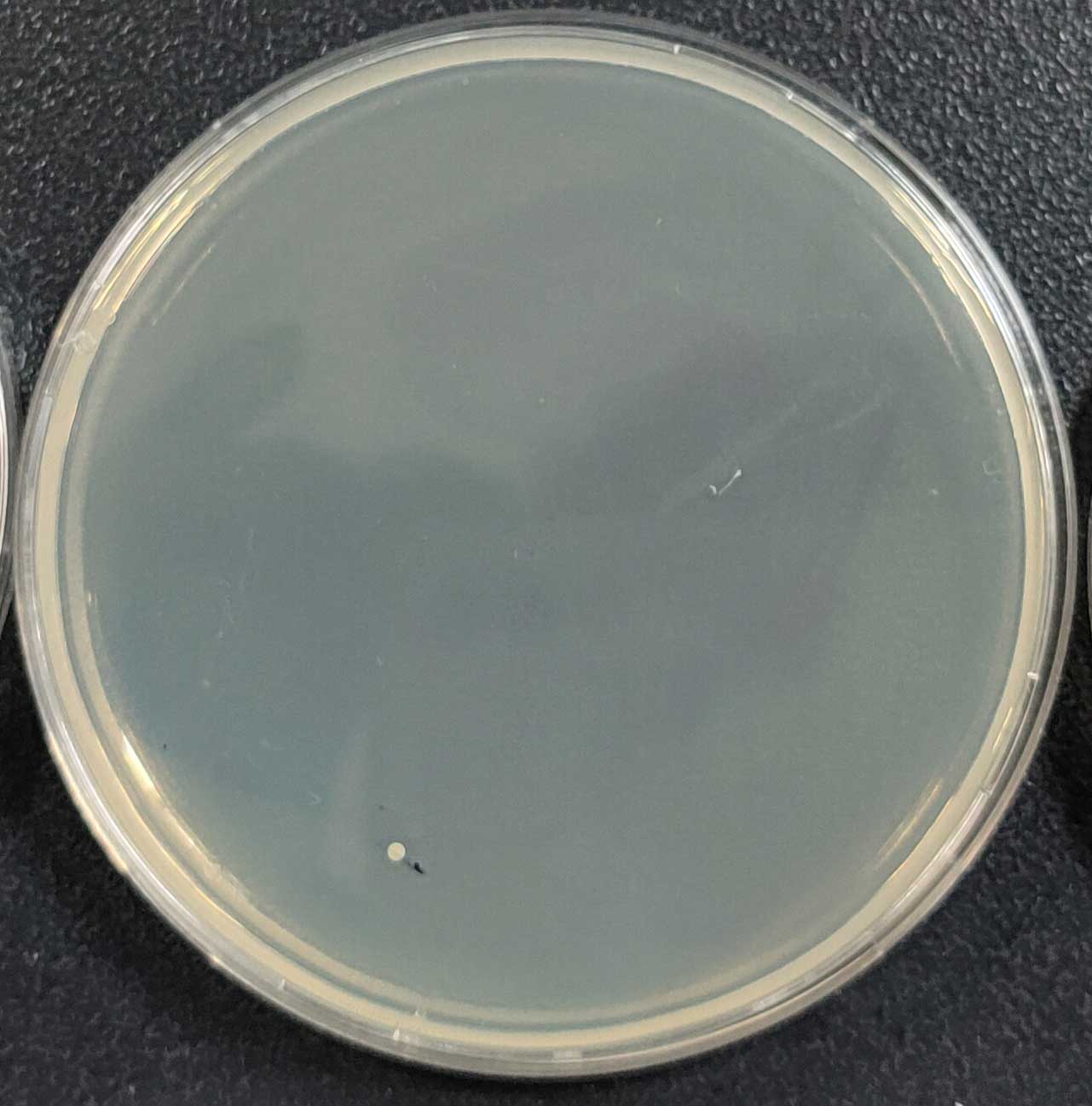

Supplement: Supplementary file 1 [file DataSheet1.zip › Data Sheet 1/Effect of 9 TCMMs on the adhesion ability of XDRAB/adhesion data/Myricetin/1.jpg]

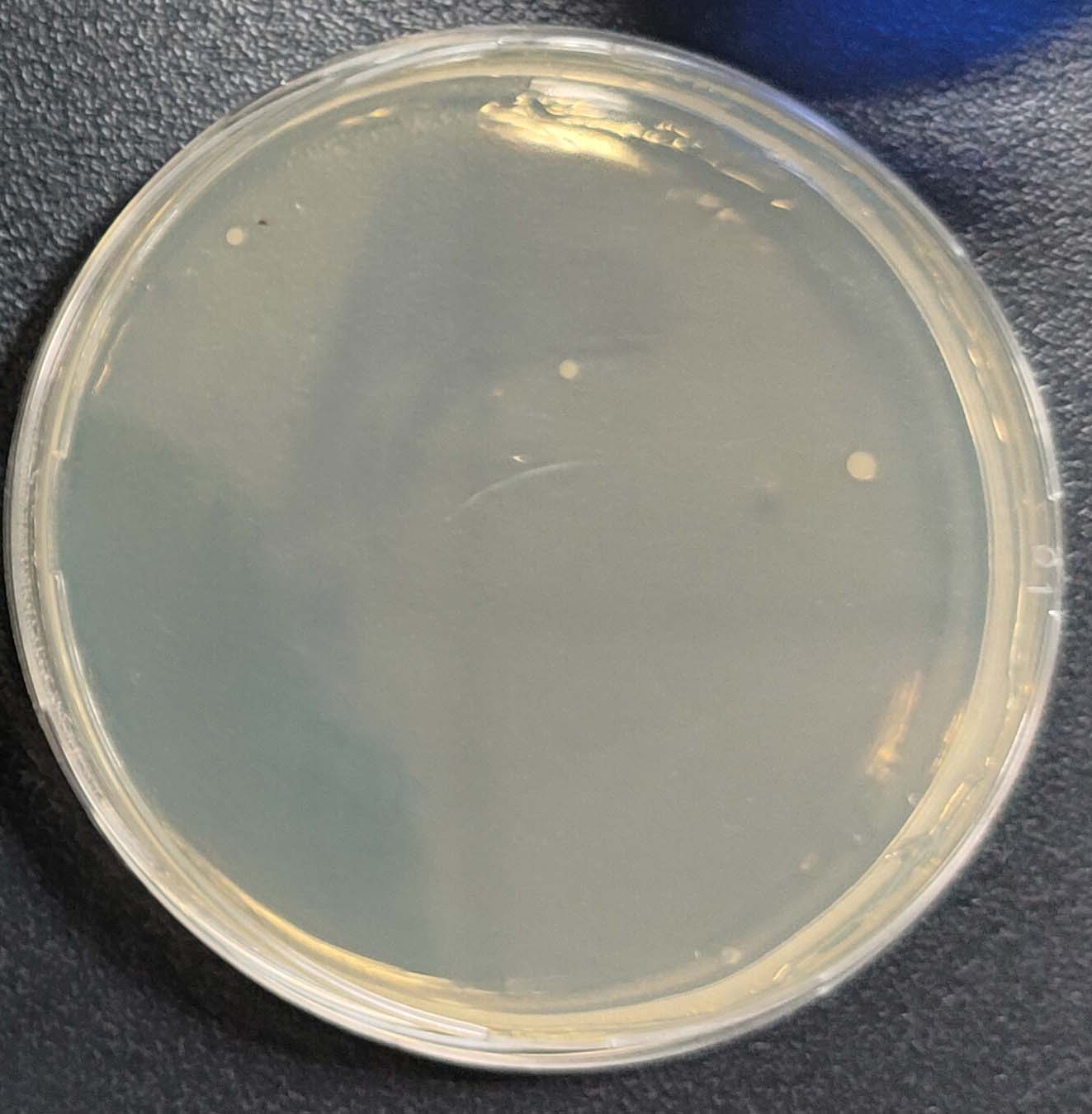

Supplement: Supplementary file 1 [file DataSheet1.zip › Data Sheet 1/Effect of 9 TCMMs on the adhesion ability of XDRAB/adhesion data/Myricetin/3.jpg]

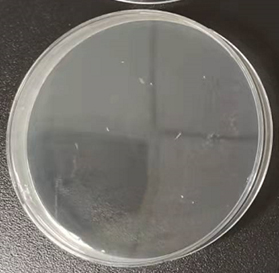

Supplement: Supplementary file 1 [file DataSheet1.zip › Data Sheet 1/Effect of 9 TCMMs on the adhesion ability of XDRAB/adhesion data/Myricetin/5.jpg]

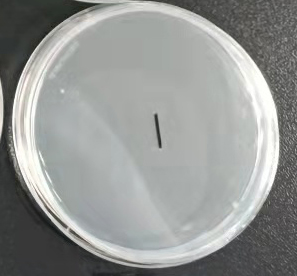

Supplement: Supplementary file 1 [file DataSheet1.zip › Data Sheet 1/Effect of 9 TCMMs on the adhesion ability of XDRAB/adhesion data/Vanillin/1 (2).jpg]

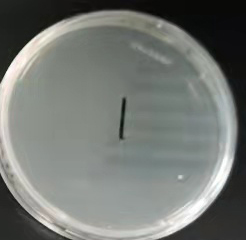

Supplement: Supplementary file 1 [file DataSheet1.zip › Data Sheet 1/Effect of 9 TCMMs on the adhesion ability of XDRAB/adhesion data/Vanillin/1.jpg]

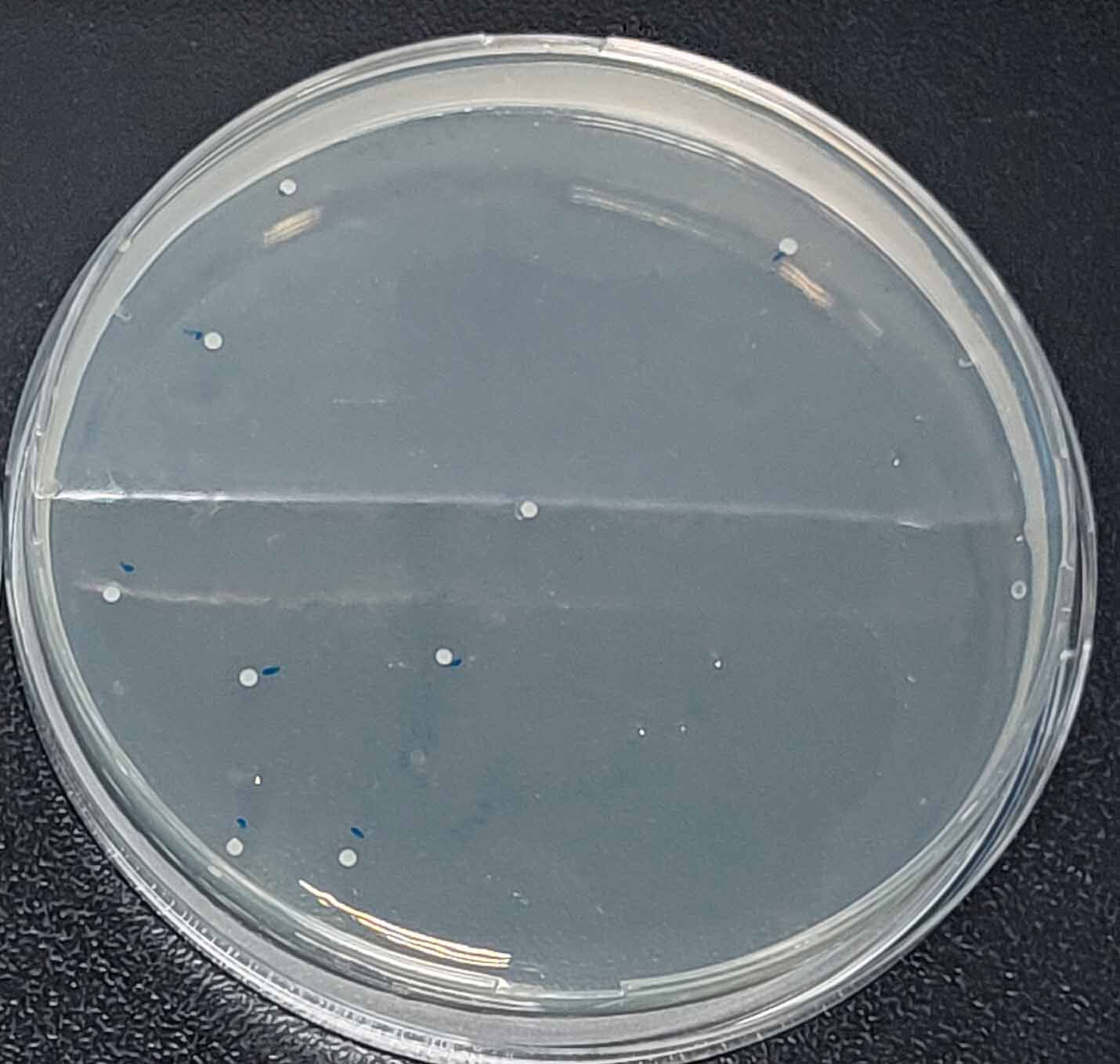

Supplement: Supplementary file 1 [file DataSheet1.zip › Data Sheet 1/Effect of 9 TCMMs on the adhesion ability of XDRAB/adhesion data/Vanillin/10.jpg]

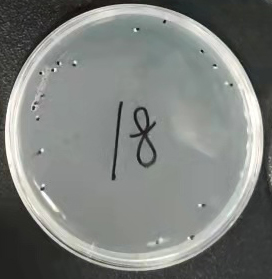

Supplement: Supplementary file 1 [file DataSheet1.zip › Data Sheet 1/Effect of 9 TCMMs on the adhesion ability of XDRAB/adhesion data/Vanillin/18.jpg]

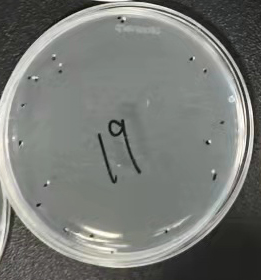

Supplement: Supplementary file 1 [file DataSheet1.zip › Data Sheet 1/Effect of 9 TCMMs on the adhesion ability of XDRAB/adhesion data/Vanillin/19.jpg]

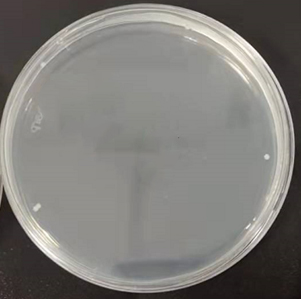

Supplement: Supplementary file 1 [file DataSheet1.zip › Data Sheet 1/Effect of 9 TCMMs on the adhesion ability of XDRAB/adhesion data/Vanillin/2 (2).jpg]

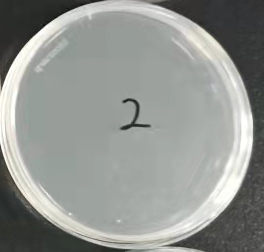

Supplement: Supplementary file 1 [file DataSheet1.zip › Data Sheet 1/Effect of 9 TCMMs on the adhesion ability of XDRAB/adhesion data/Vanillin/2.jpg]

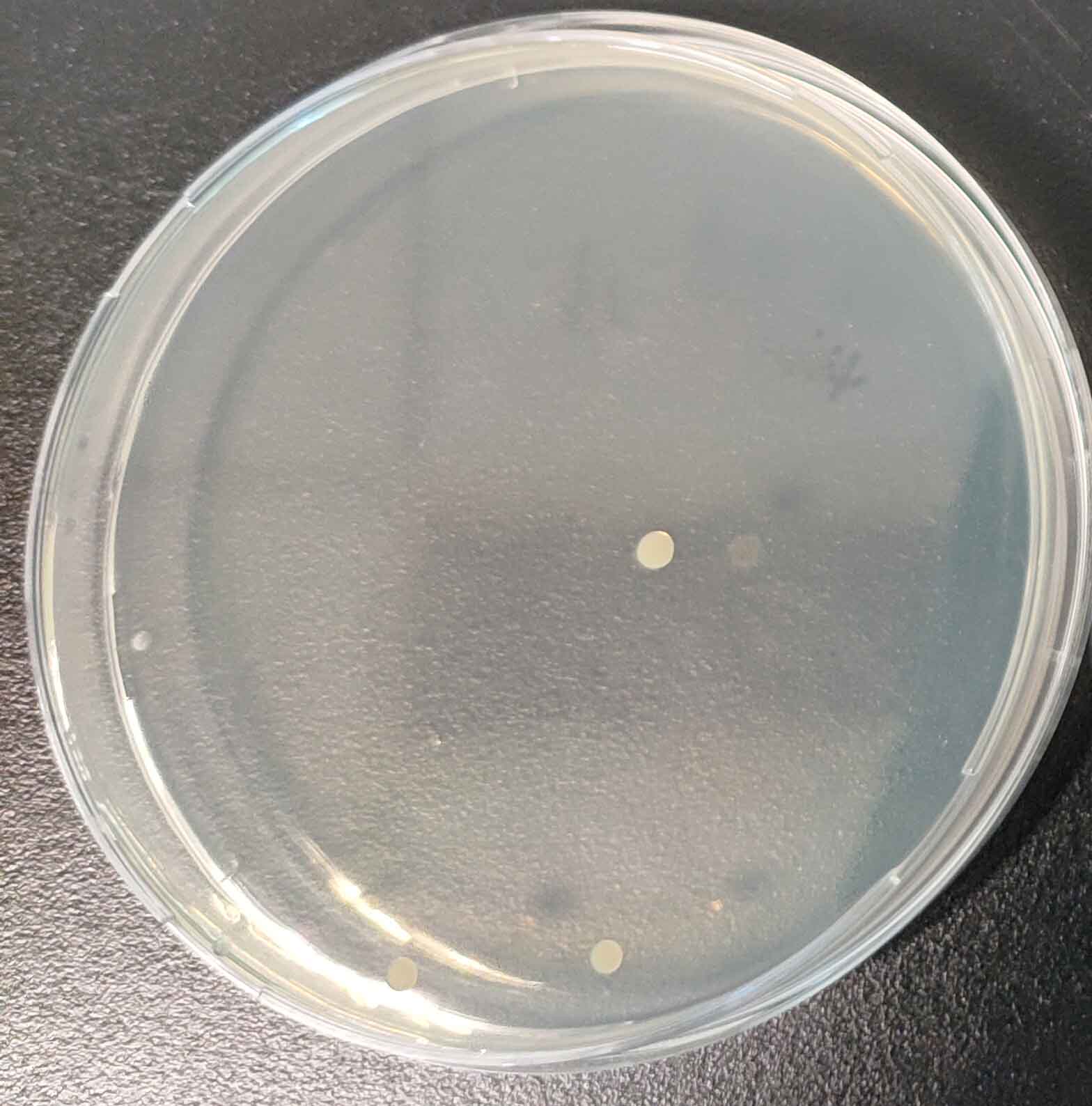

Supplement: Supplementary file 1 [file DataSheet1.zip › Data Sheet 1/Effect of 9 TCMMs on the adhesion ability of XDRAB/adhesion data/Vanillin/3.jpg]

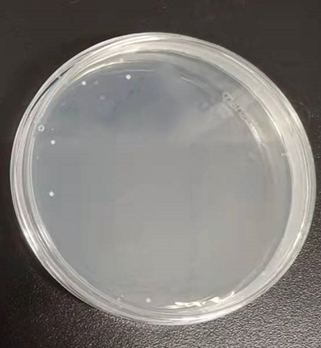

Supplement: Supplementary file 1 [file DataSheet1.zip › Data Sheet 1/Effect of 9 TCMMs on the adhesion ability of XDRAB/adhesion data/Vanillin/5 (2).jpg]

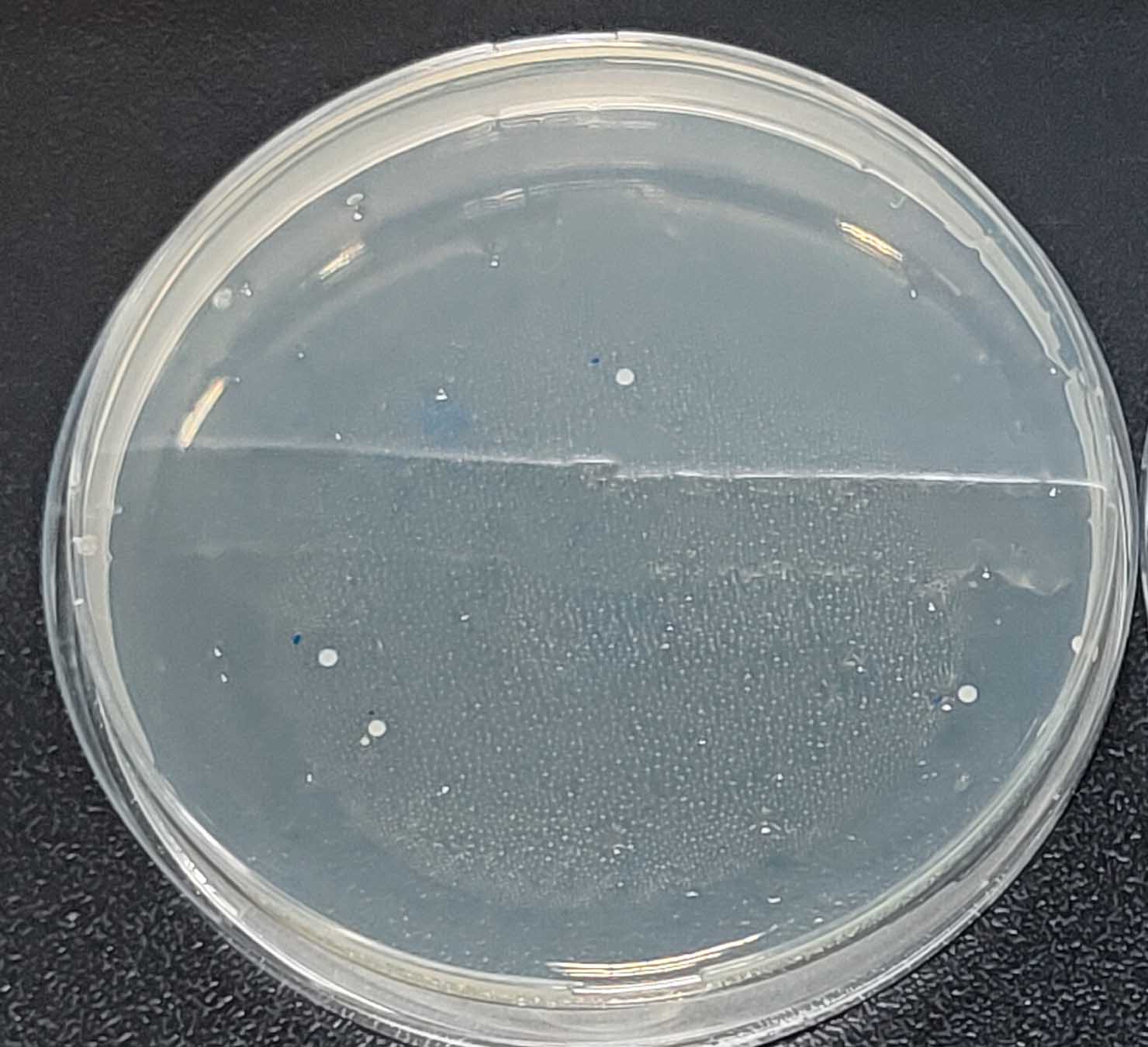

Supplement: Supplementary file 1 [file DataSheet1.zip › Data Sheet 1/Effect of 9 TCMMs on the adhesion ability of XDRAB/adhesion data/Vanillin/5.jpg]

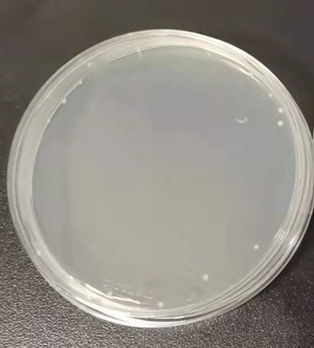

Supplement: Supplementary file 1 [file DataSheet1.zip › Data Sheet 1/Effect of 9 TCMMs on the adhesion ability of XDRAB/adhesion data/Vanillin/6.jpg]

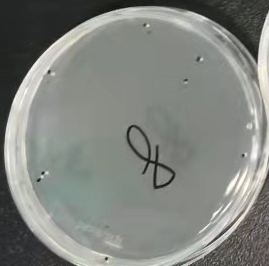

Supplement: Supplementary file 1 [file DataSheet1.zip › Data Sheet 1/Effect of 9 TCMMs on the adhesion ability of XDRAB/adhesion data/Vanillin/8.jpg]

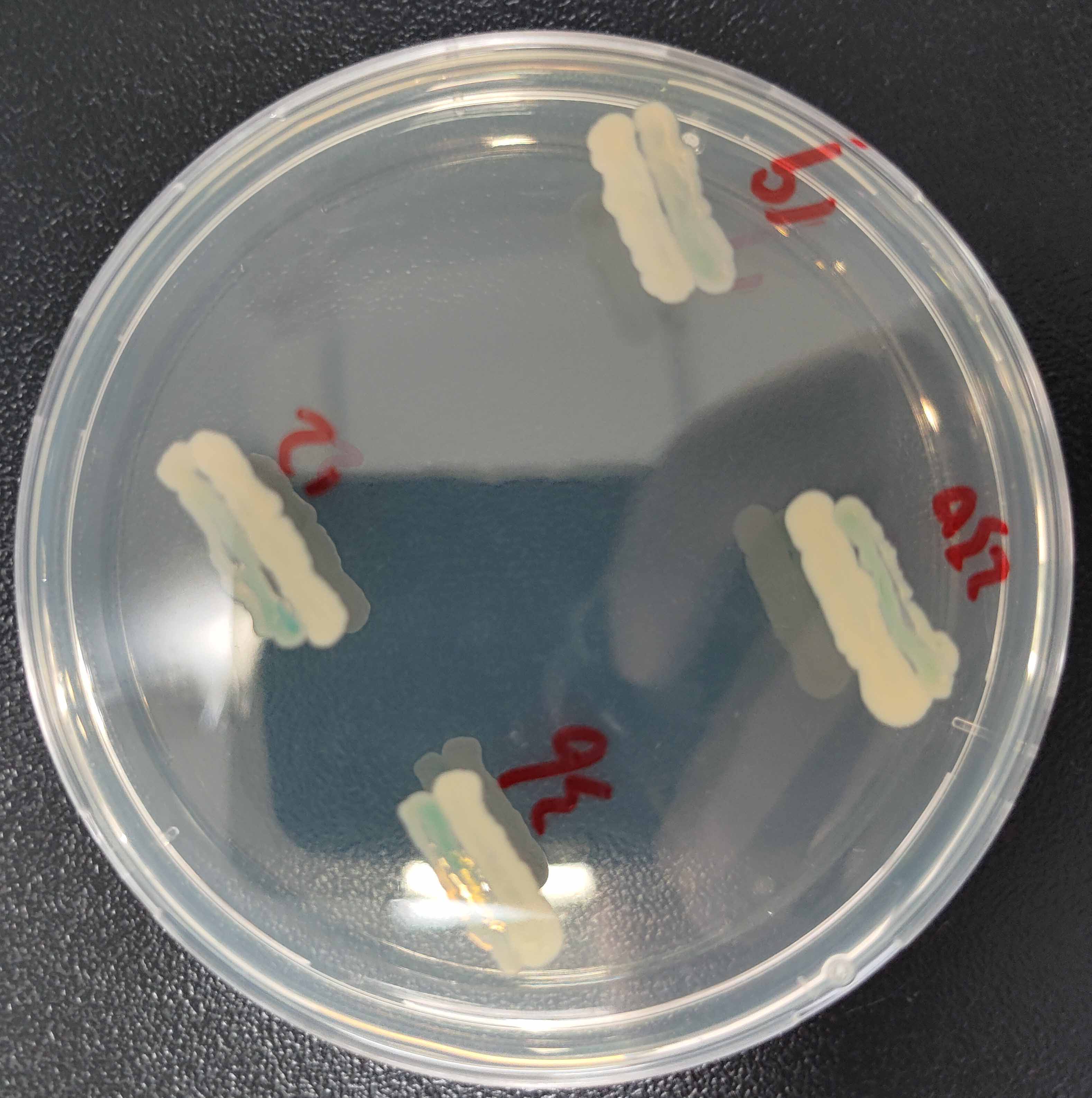

Supplement: Supplementary file 2 [file DataSheet2.ZIP › data/AHLs production of 60 clinical strains of A. baumannii/1.jpg]

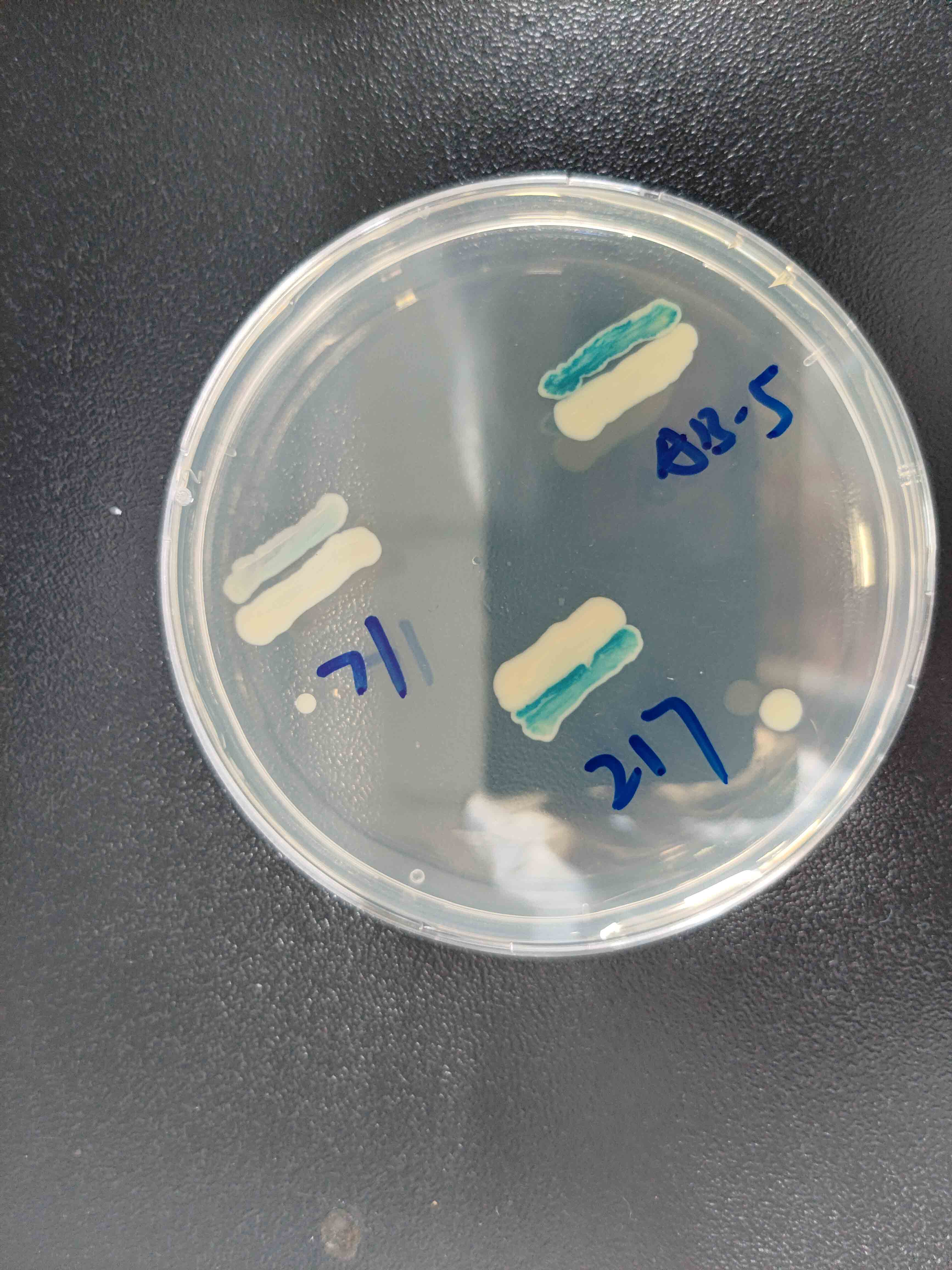

Supplement: Supplementary file 2 [file DataSheet2.ZIP › data/AHLs production of 60 clinical strains of A. baumannii/10.jpg]

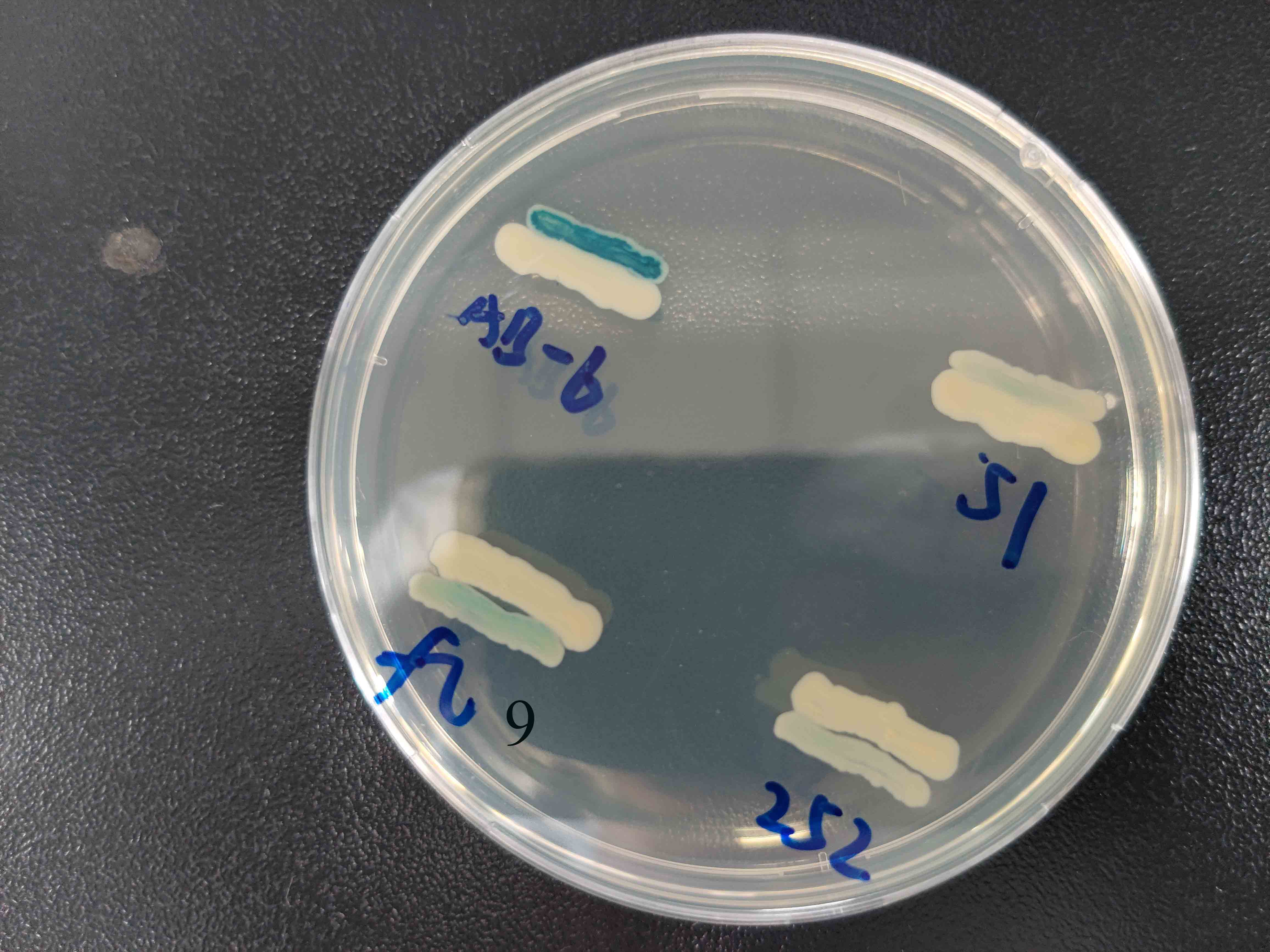

Supplement: Supplementary file 2 [file DataSheet2.ZIP › data/AHLs production of 60 clinical strains of A. baumannii/11.jpg]

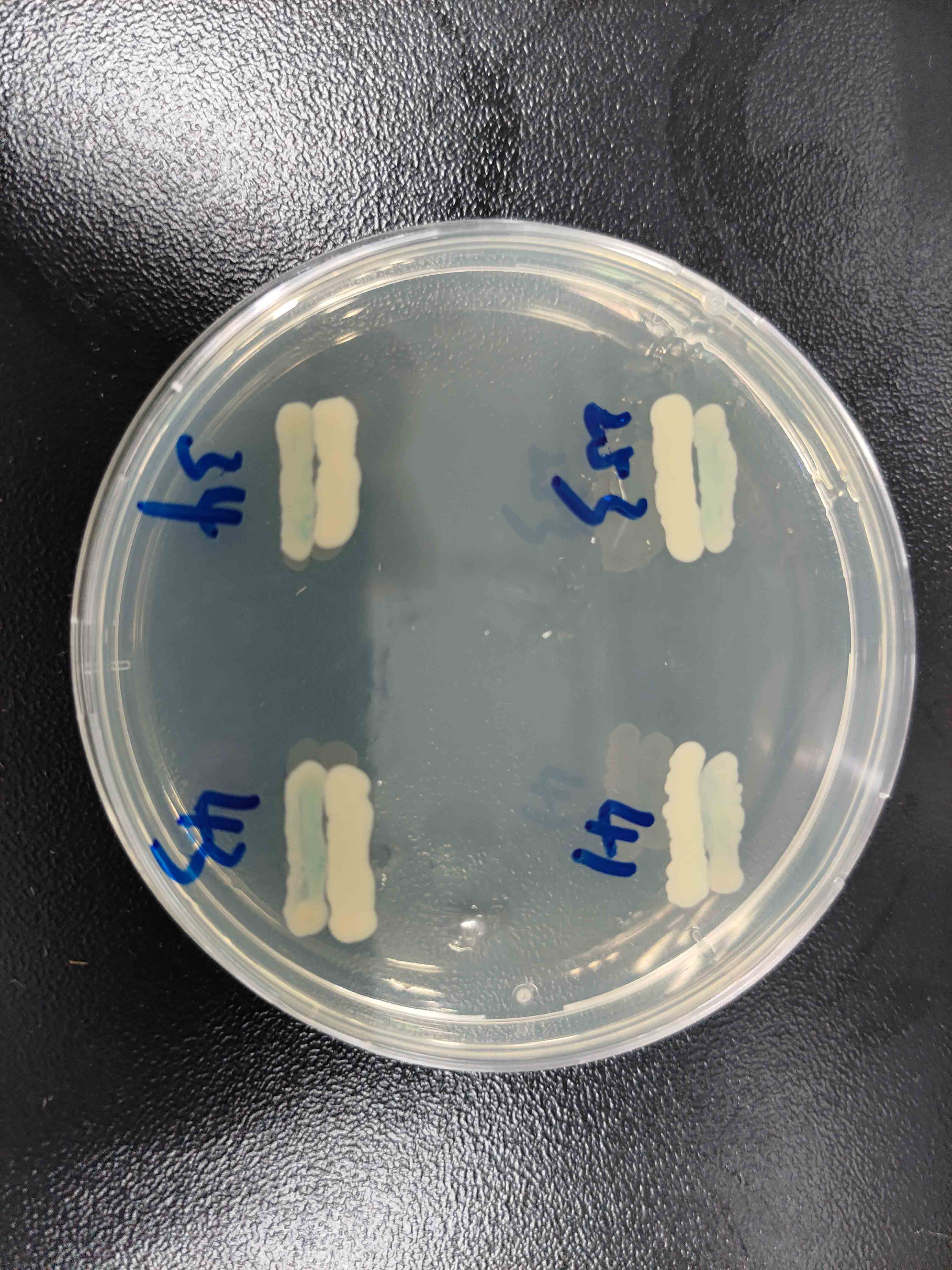

Supplement: Supplementary file 2 [file DataSheet2.ZIP › data/AHLs production of 60 clinical strains of A. baumannii/12.jpg]

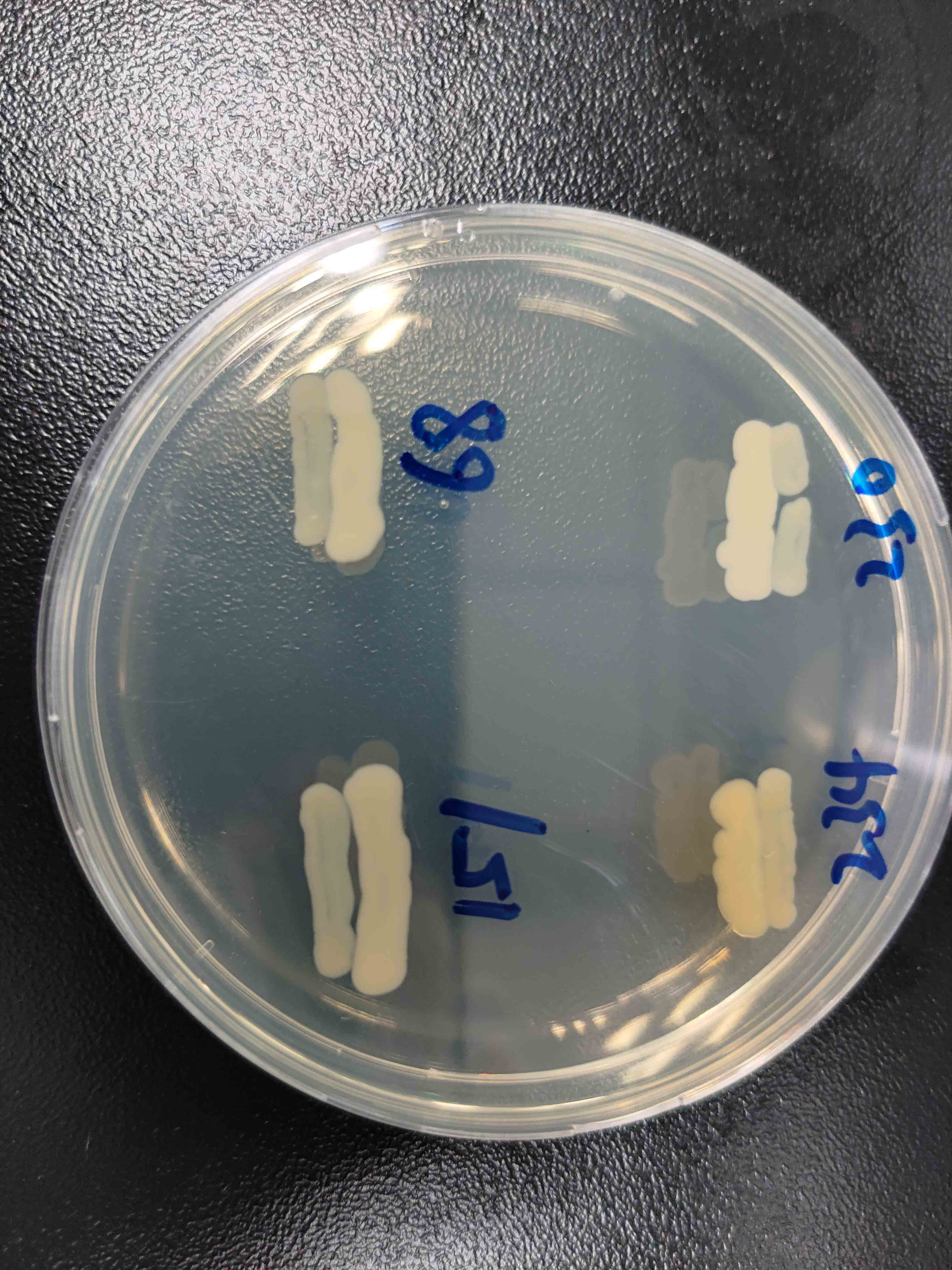

Supplement: Supplementary file 2 [file DataSheet2.ZIP › data/AHLs production of 60 clinical strains of A. baumannii/13.jpg]

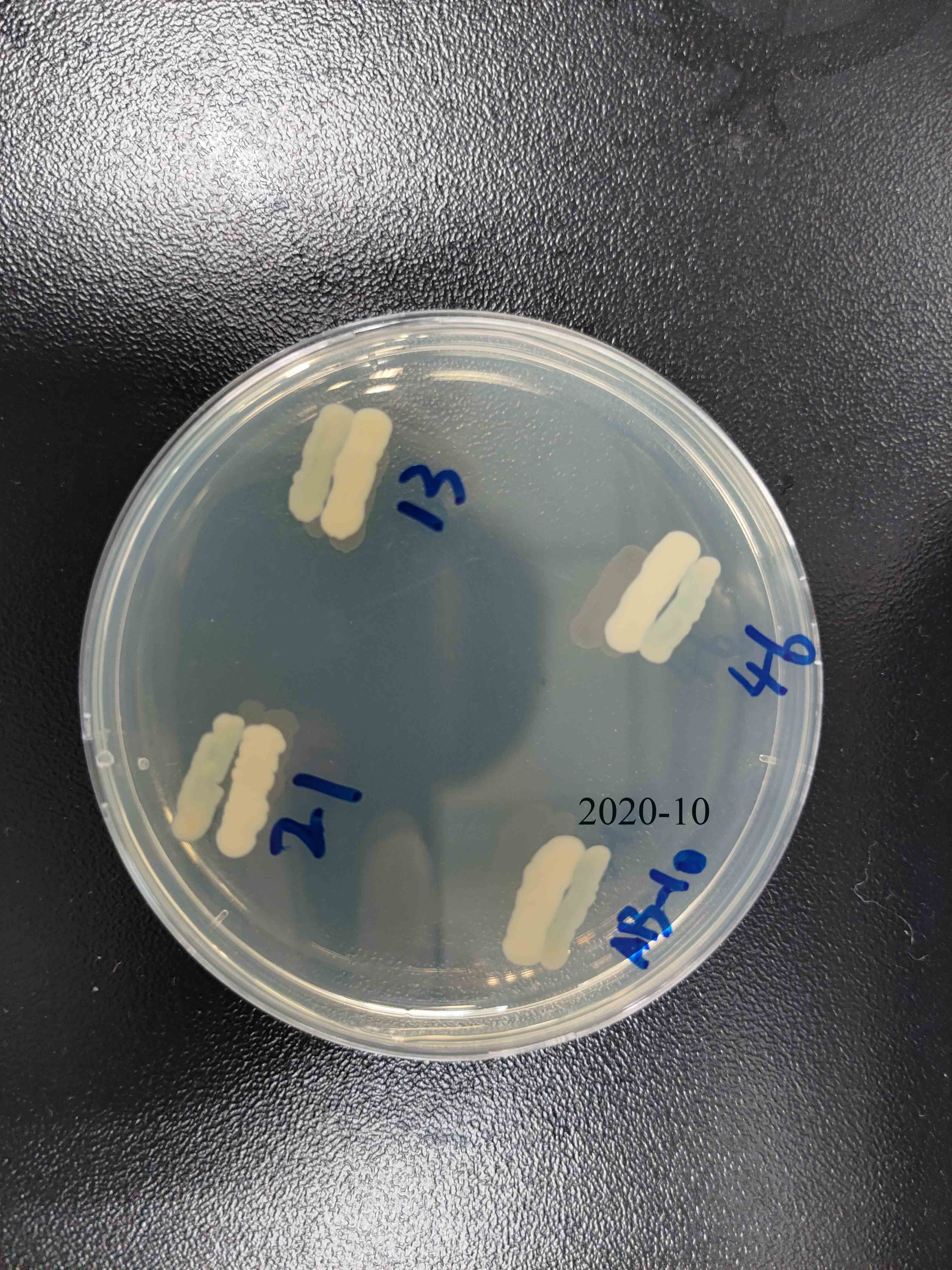

Supplement: Supplementary file 2 [file DataSheet2.ZIP › data/AHLs production of 60 clinical strains of A. baumannii/14.jpg]

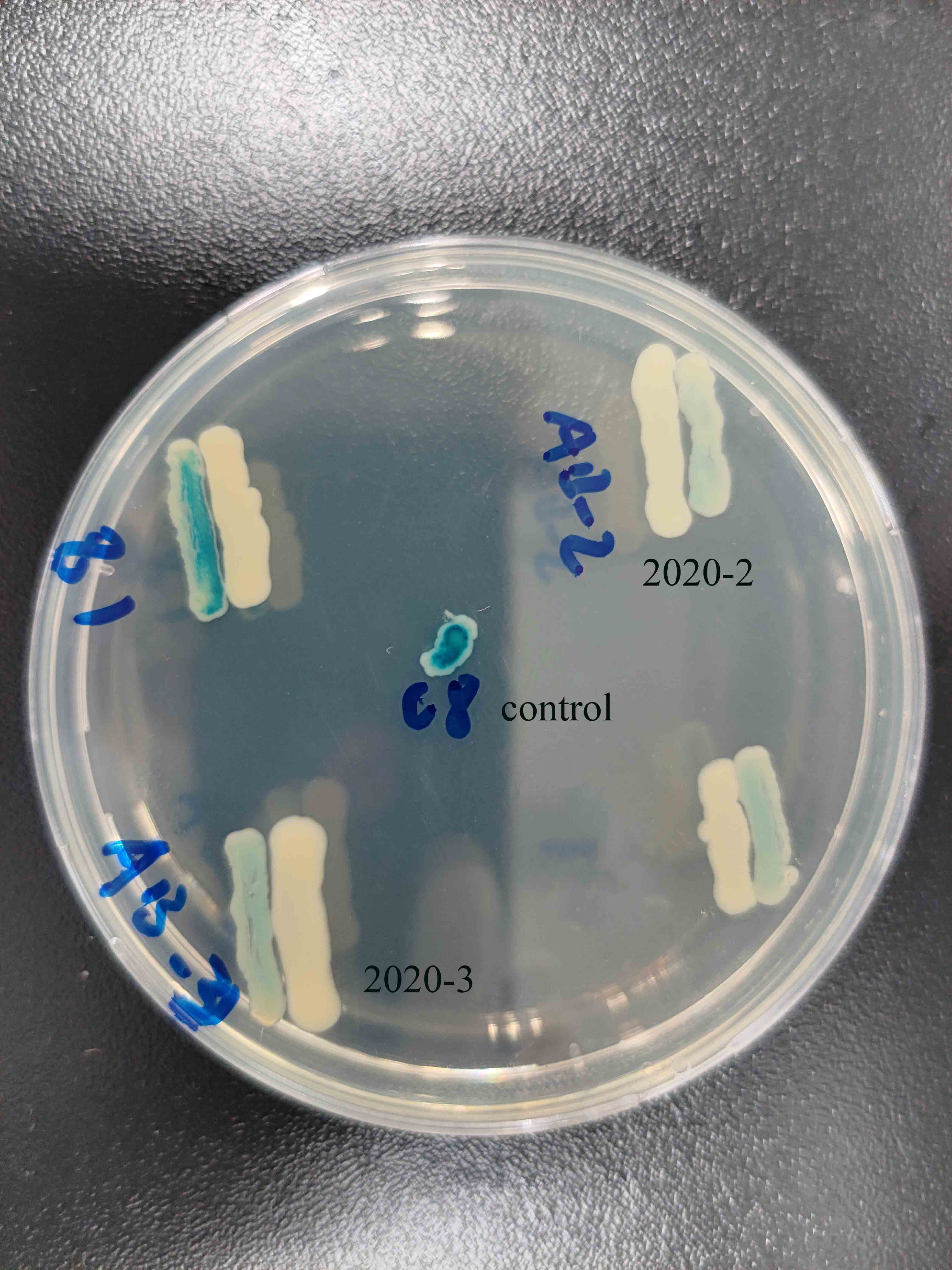

Supplement: Supplementary file 2 [file DataSheet2.ZIP › data/AHLs production of 60 clinical strains of A. baumannii/15.jpg]

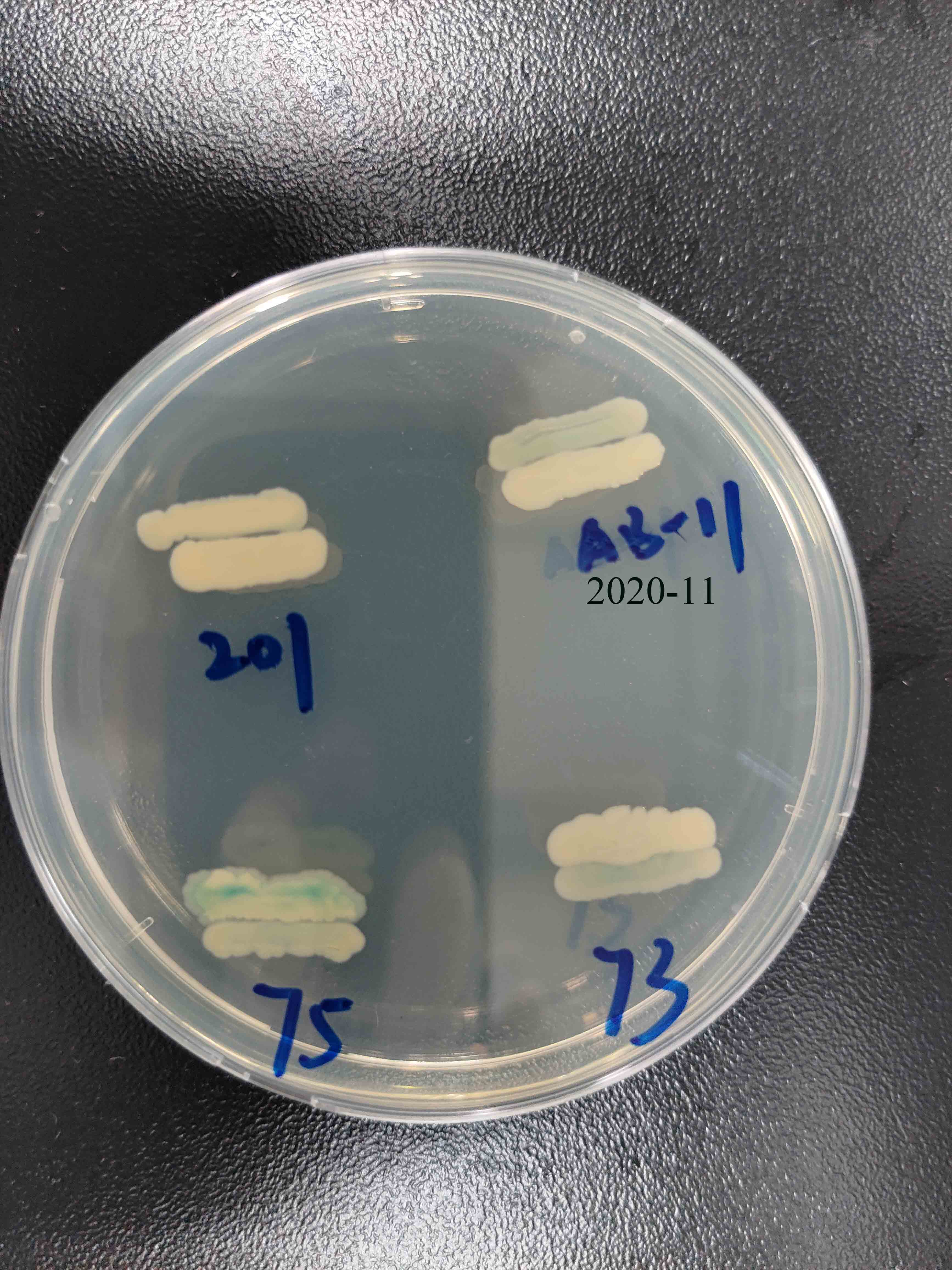

Supplement: Supplementary file 2 [file DataSheet2.ZIP › data/AHLs production of 60 clinical strains of A. baumannii/16.jpg]

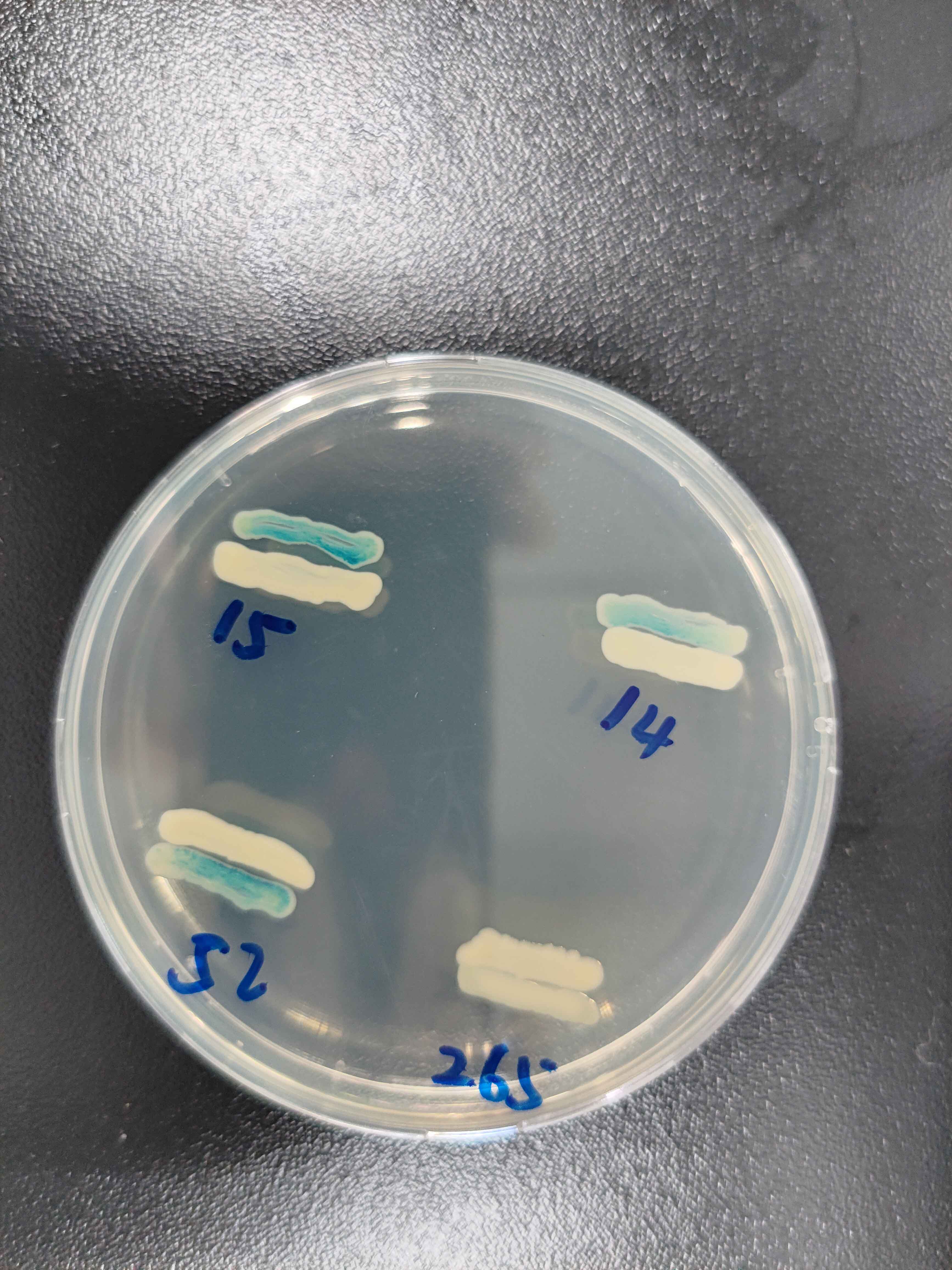

Supplement: Supplementary file 2 [file DataSheet2.ZIP › data/AHLs production of 60 clinical strains of A. baumannii/17.jpg]

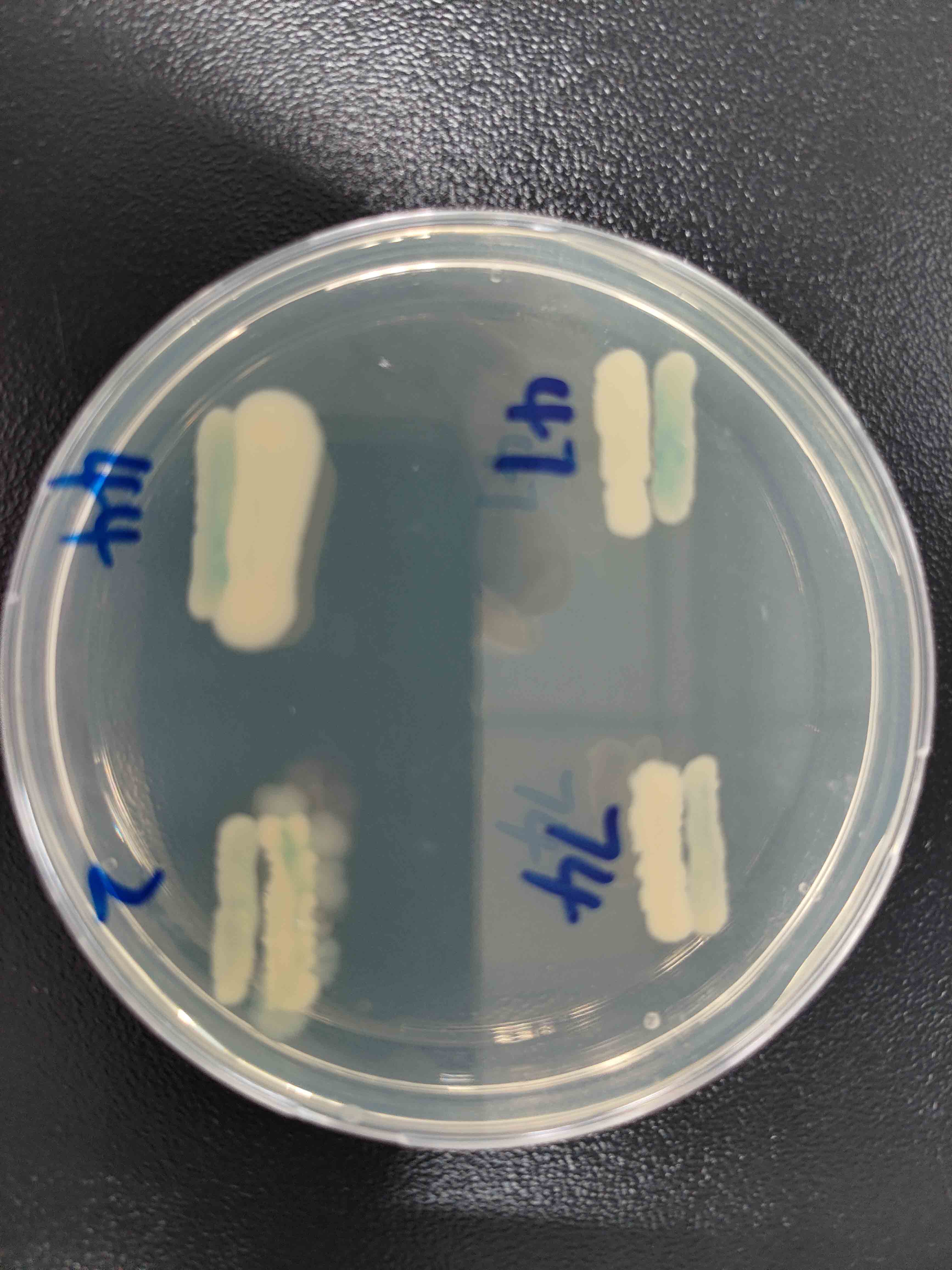

Supplement: Supplementary file 2 [file DataSheet2.ZIP › data/AHLs production of 60 clinical strains of A. baumannii/18.jpg]

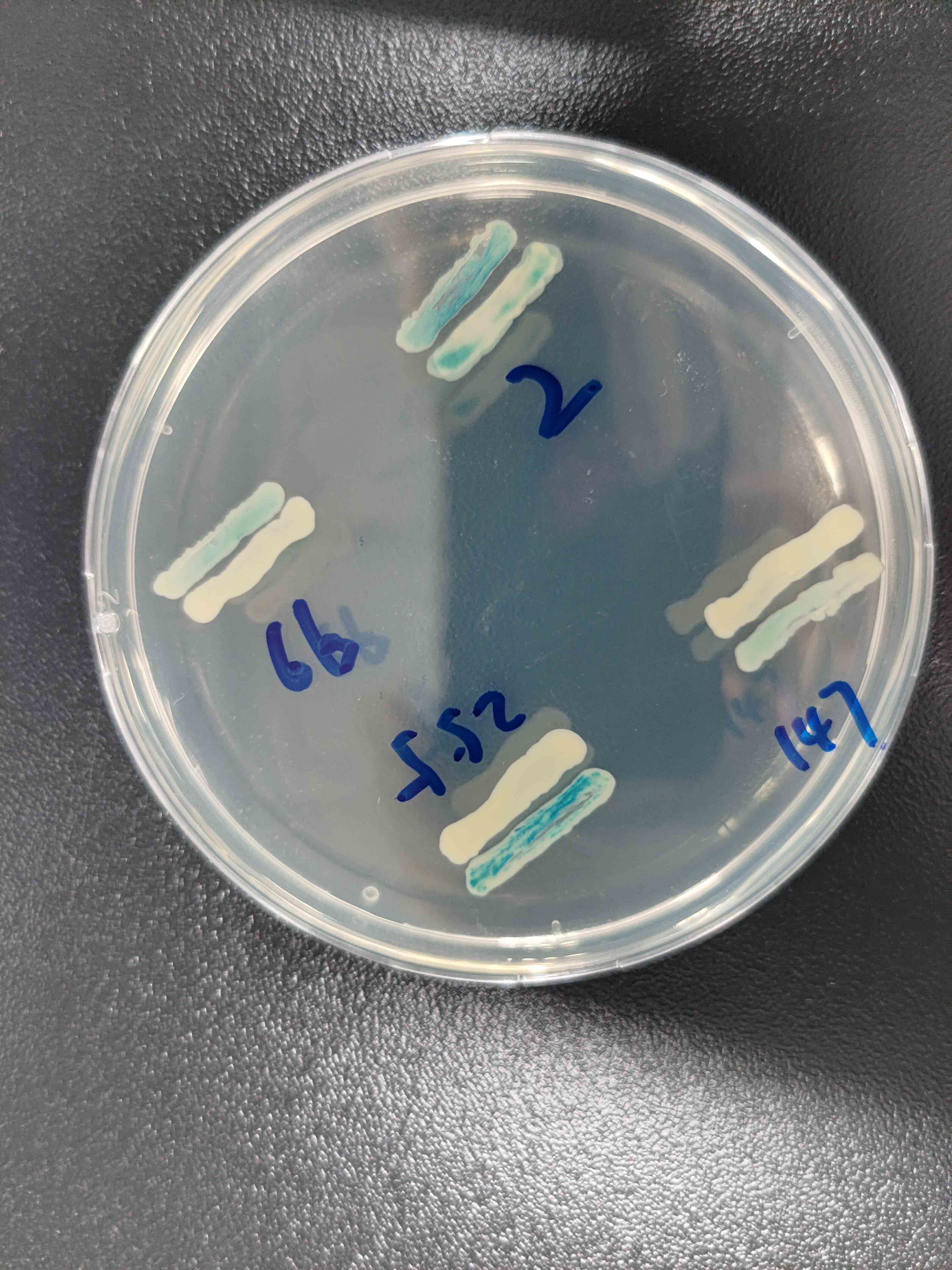

Supplement: Supplementary file 2 [file DataSheet2.ZIP › data/AHLs production of 60 clinical strains of A. baumannii/19.jpg]

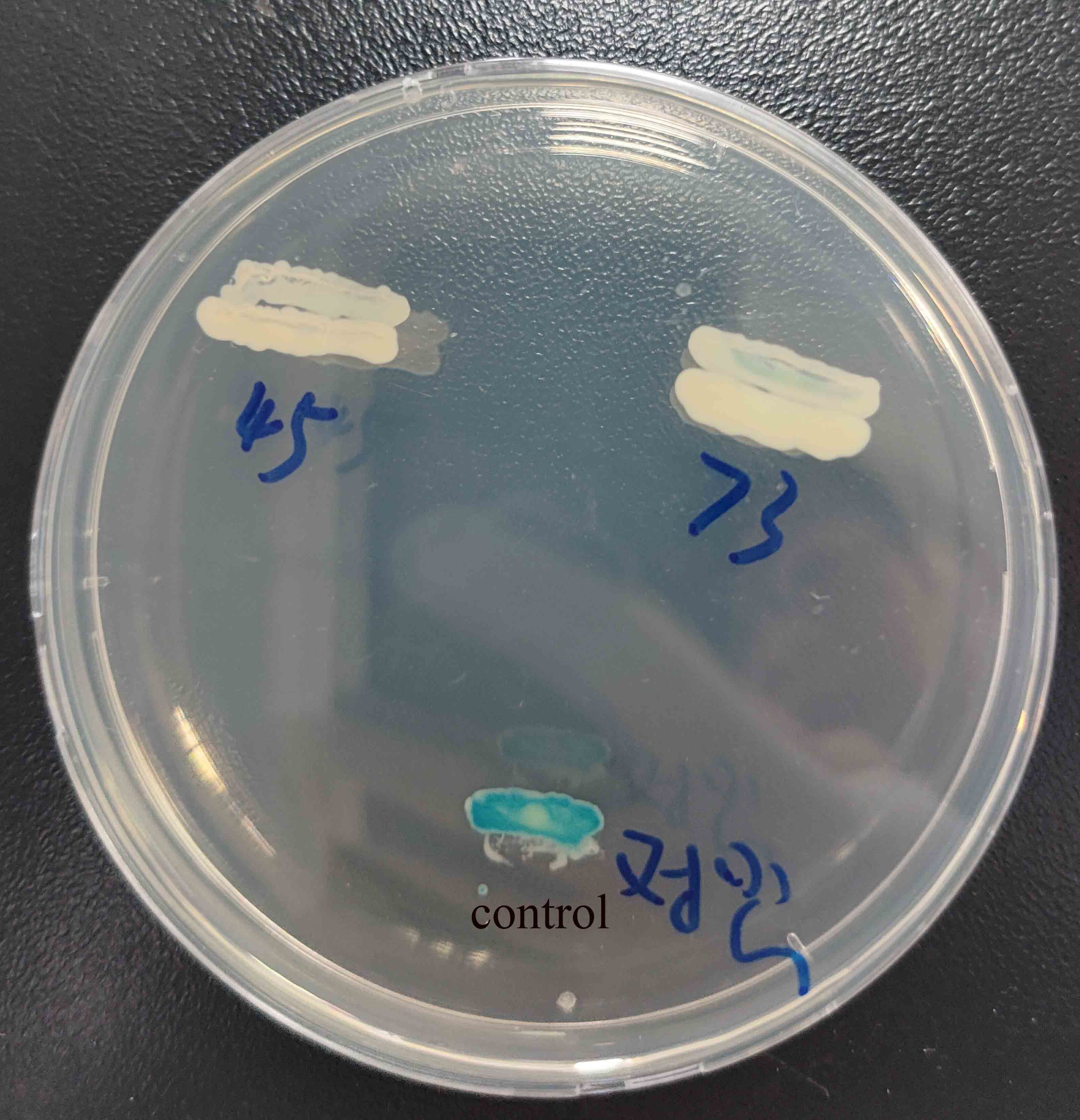

Supplement: Supplementary file 2 [file DataSheet2.ZIP › data/AHLs production of 60 clinical strains of A. baumannii/2.jpg]

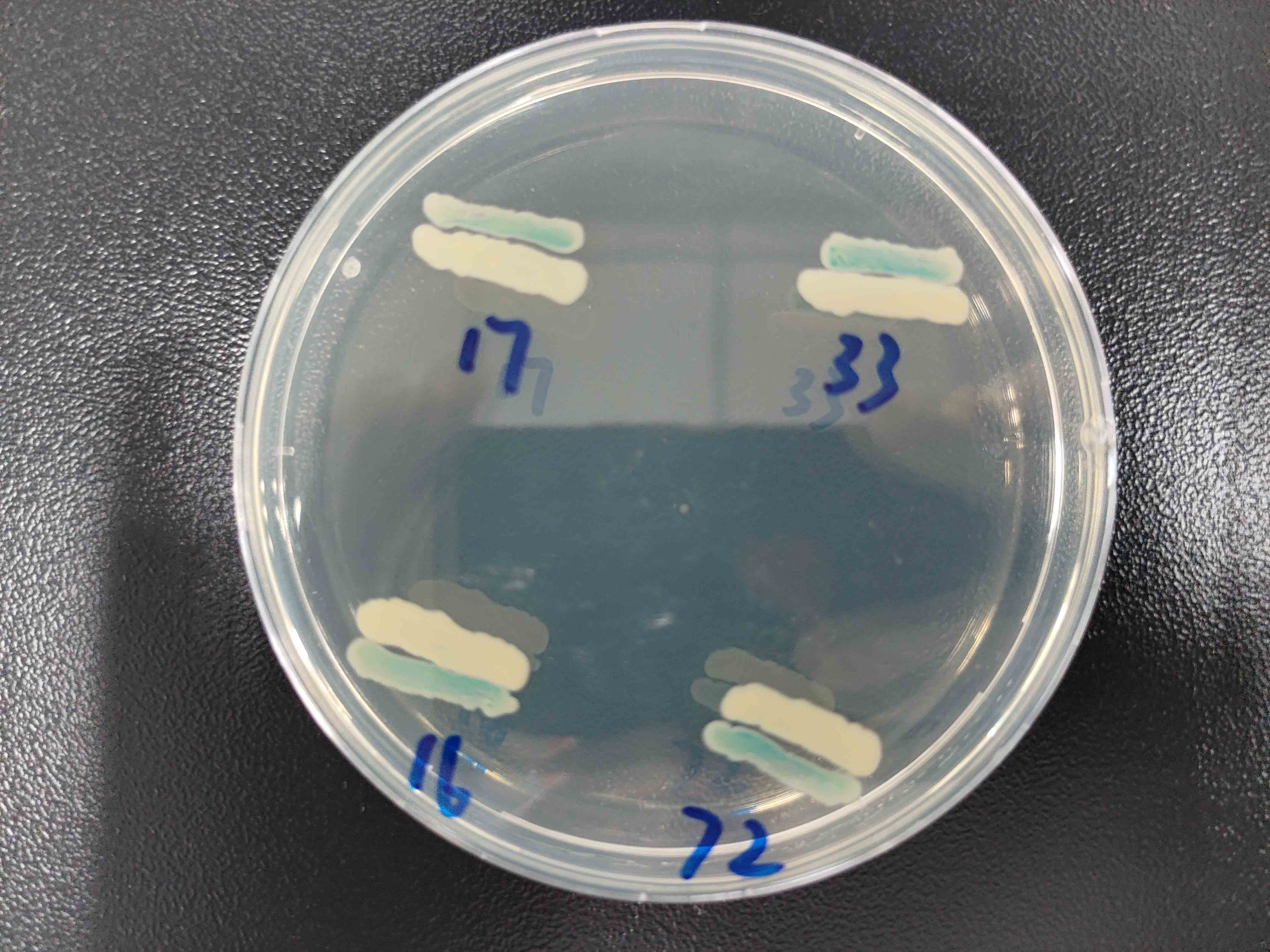

Supplement: Supplementary file 2 [file DataSheet2.ZIP › data/AHLs production of 60 clinical strains of A. baumannii/20.jpg]

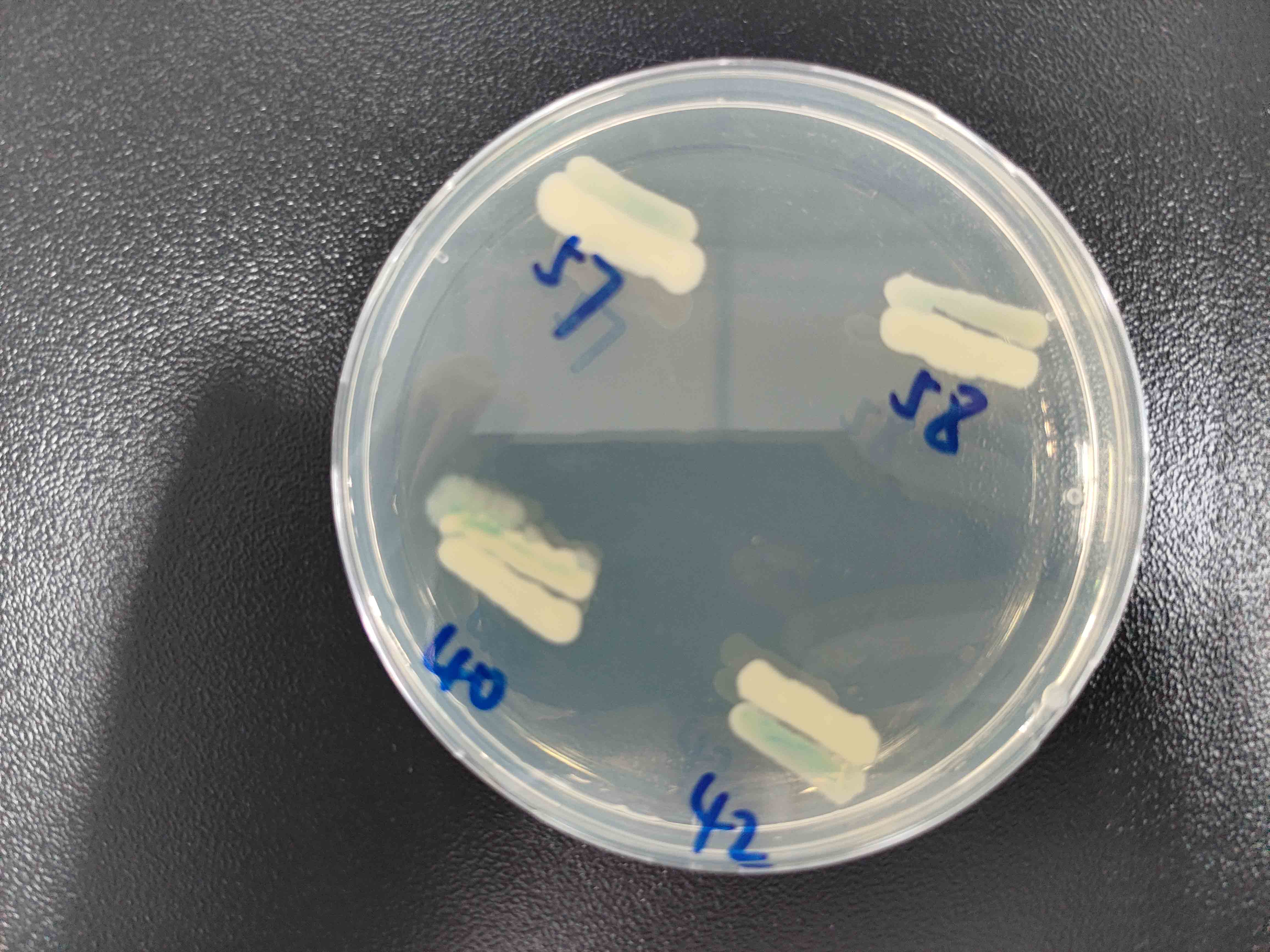

Supplement: Supplementary file 2 [file DataSheet2.ZIP › data/AHLs production of 60 clinical strains of A. baumannii/21.jpg]

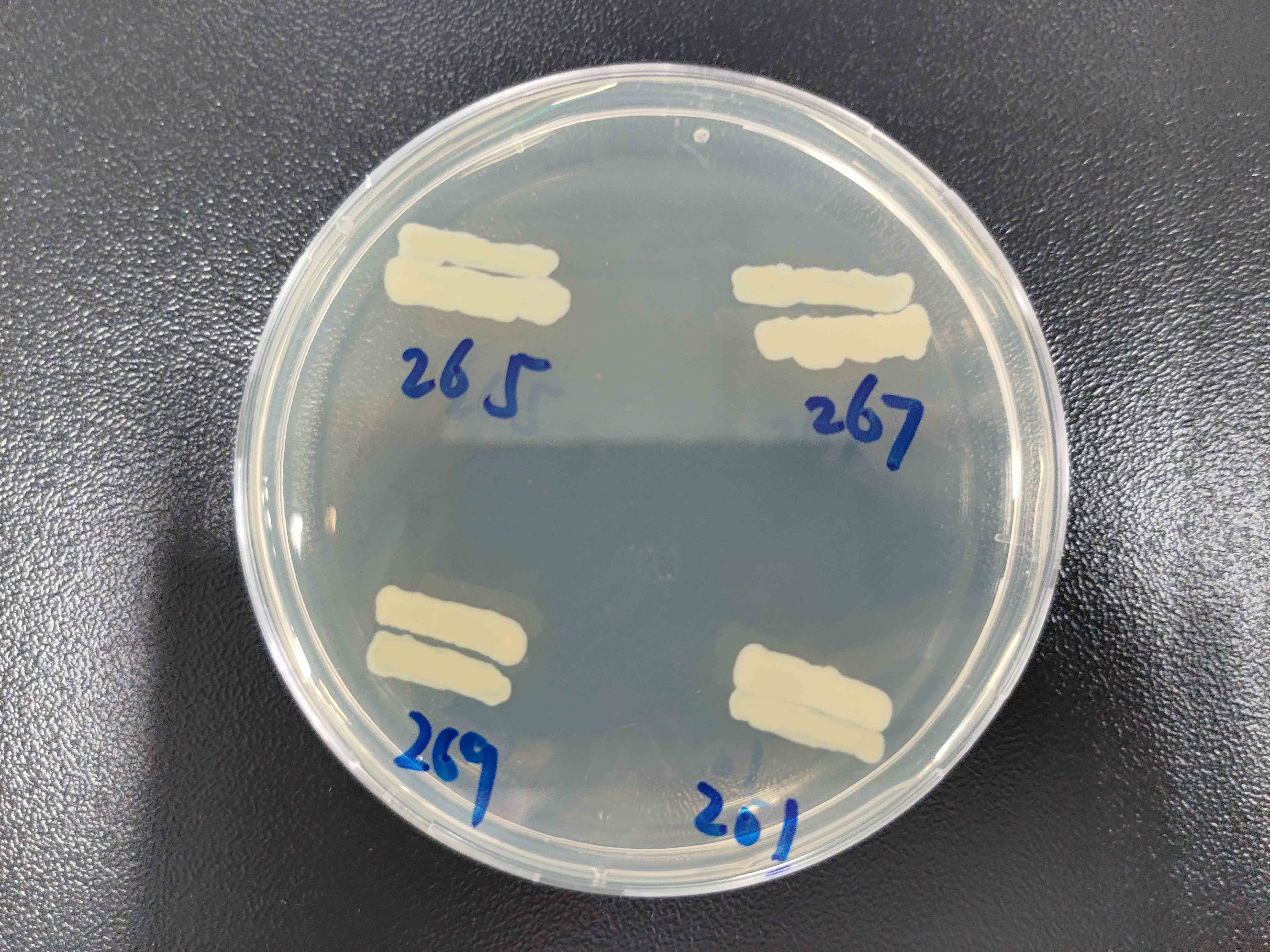

Supplement: Supplementary file 2 [file DataSheet2.ZIP › data/AHLs production of 60 clinical strains of A. baumannii/22.jpg]

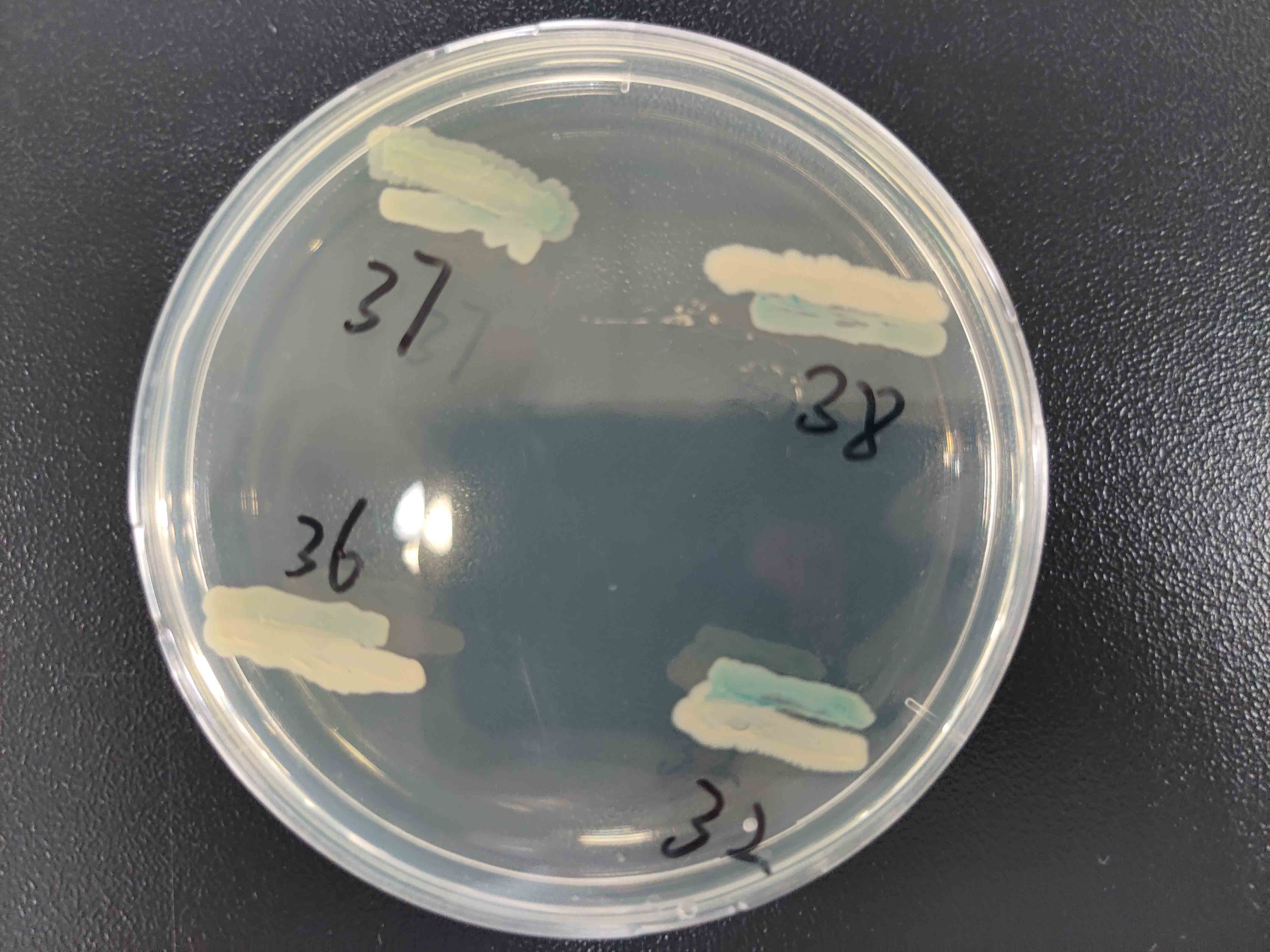

Supplement: Supplementary file 2 [file DataSheet2.ZIP › data/AHLs production of 60 clinical strains of A. baumannii/23.jpg]

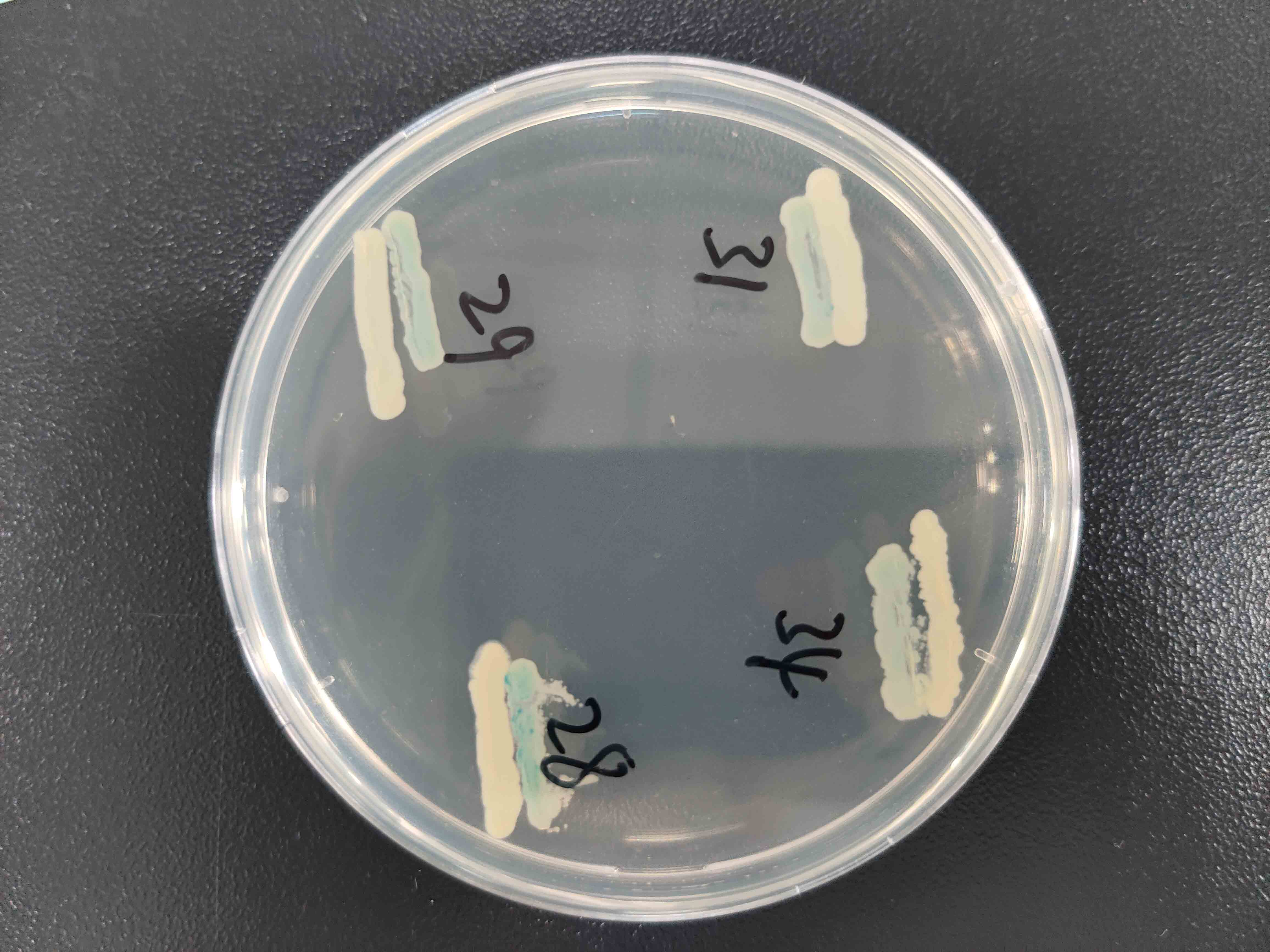

Supplement: Supplementary file 2 [file DataSheet2.ZIP › data/AHLs production of 60 clinical strains of A. baumannii/24.jpg]

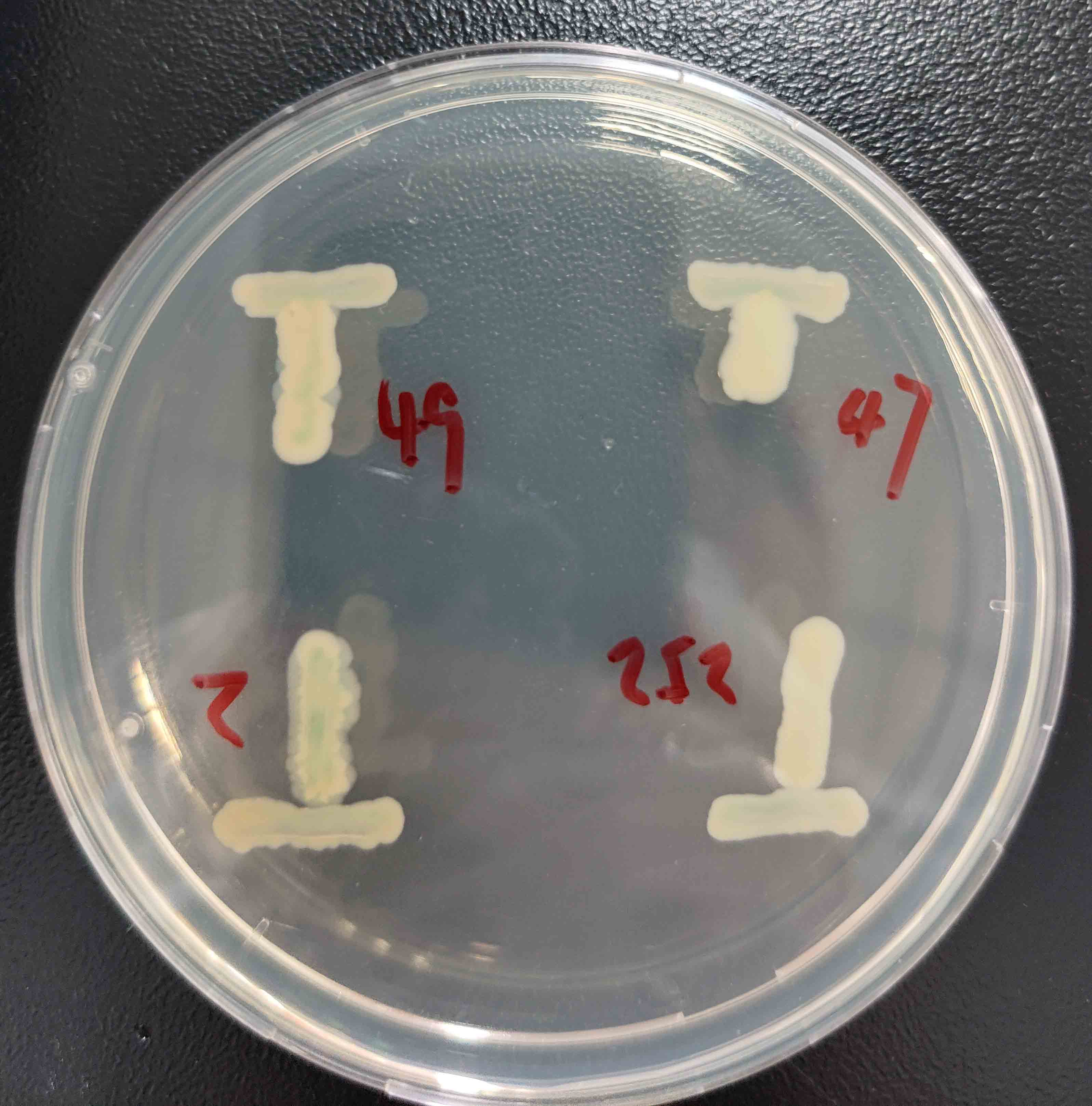

Supplement: Supplementary file 2 [file DataSheet2.ZIP › data/AHLs production of 60 clinical strains of A. baumannii/3.jpg]

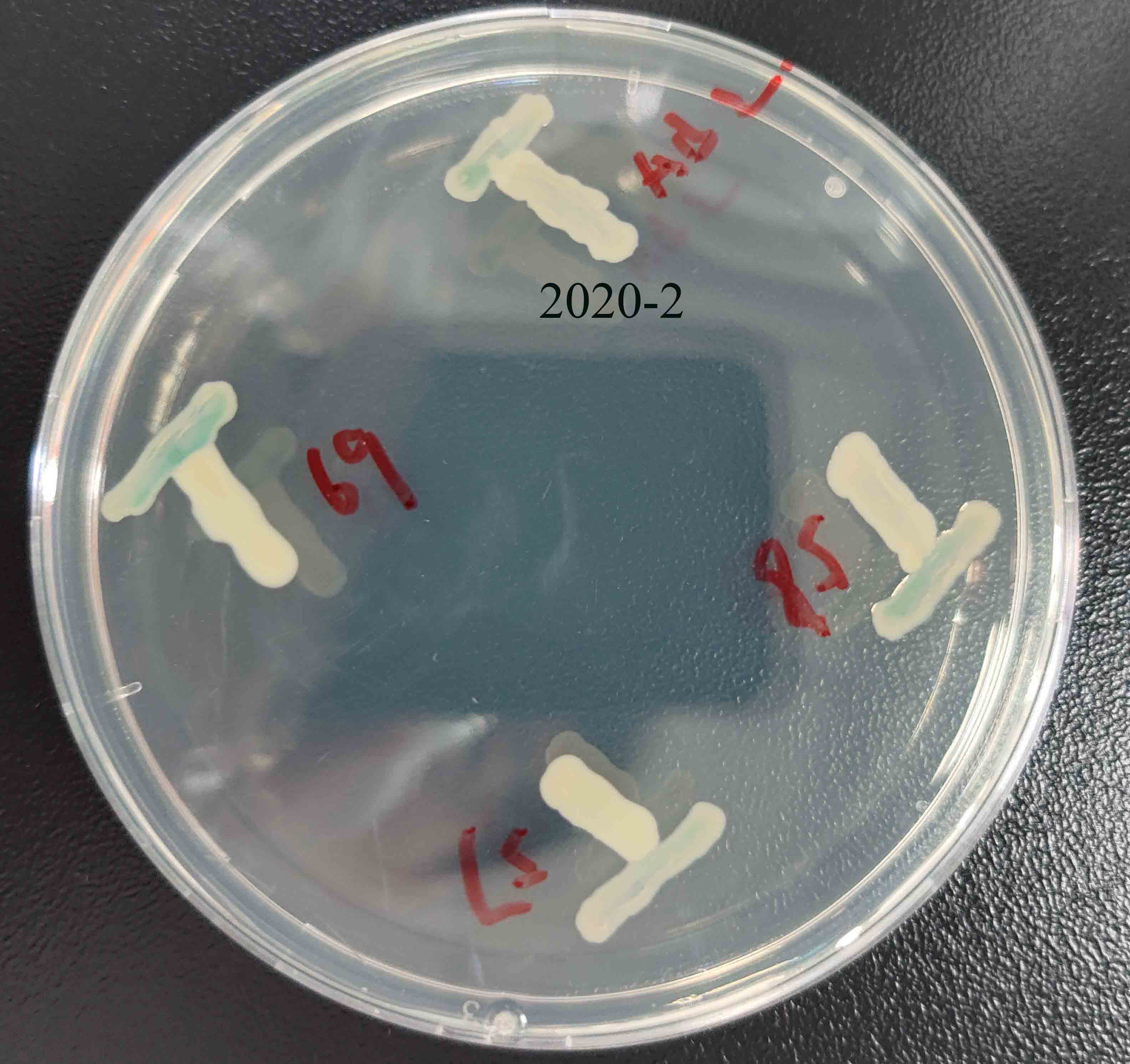

Supplement: Supplementary file 2 [file DataSheet2.ZIP › data/AHLs production of 60 clinical strains of A. baumannii/4.jpg]

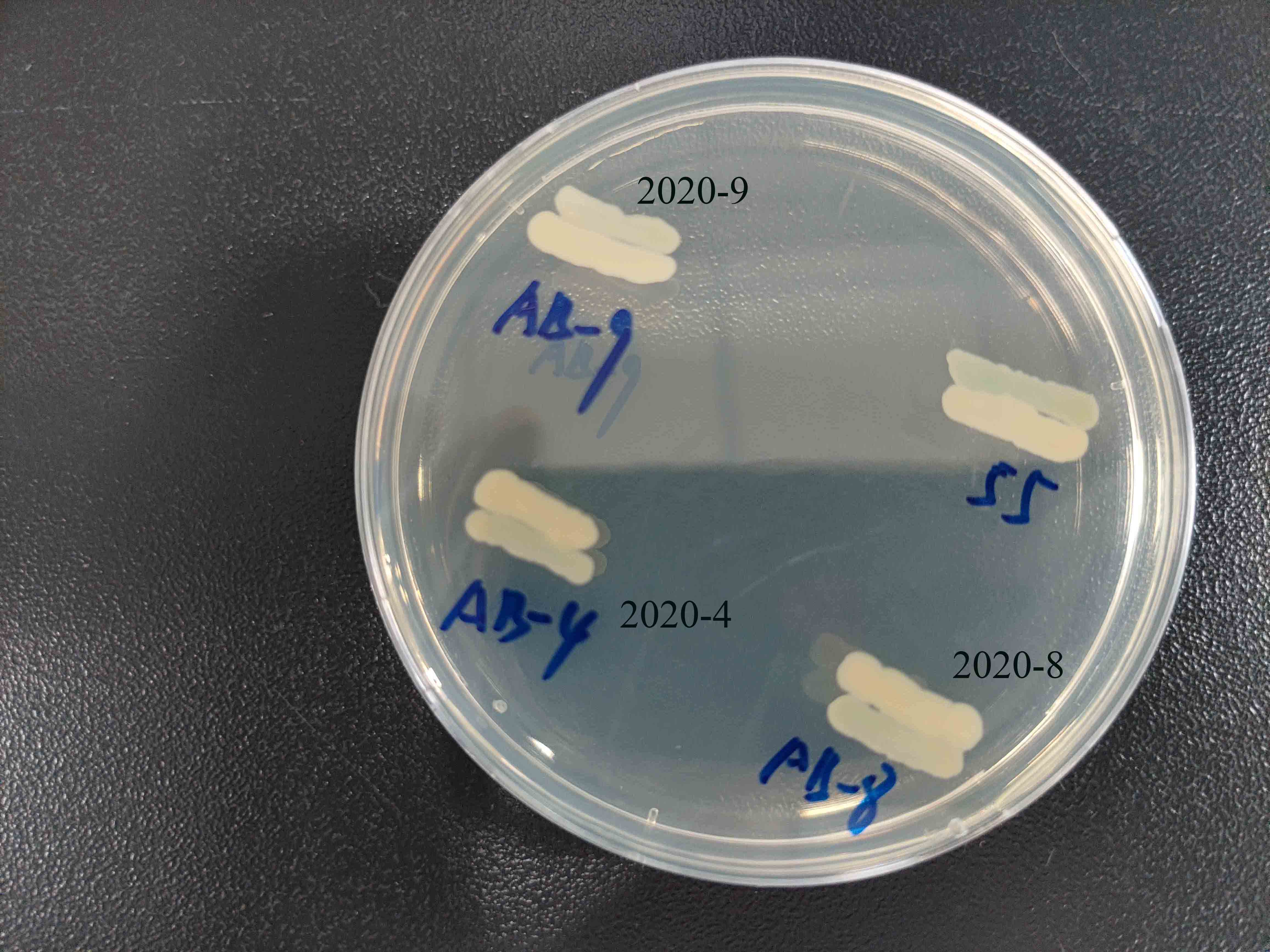

Supplement: Supplementary file 2 [file DataSheet2.ZIP › data/AHLs production of 60 clinical strains of A. baumannii/5.jpg]

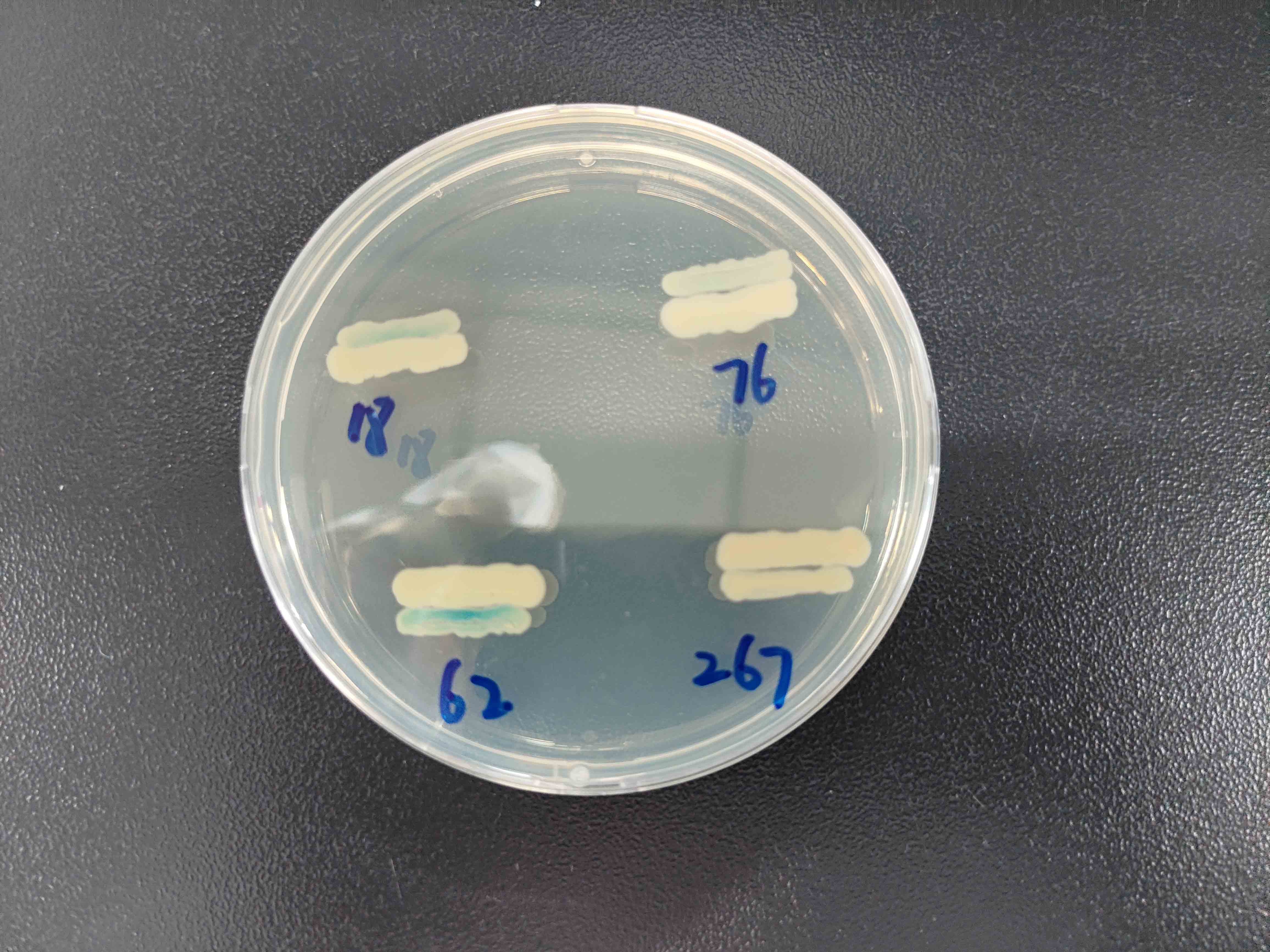

Supplement: Supplementary file 2 [file DataSheet2.ZIP › data/AHLs production of 60 clinical strains of A. baumannii/6.jpg]

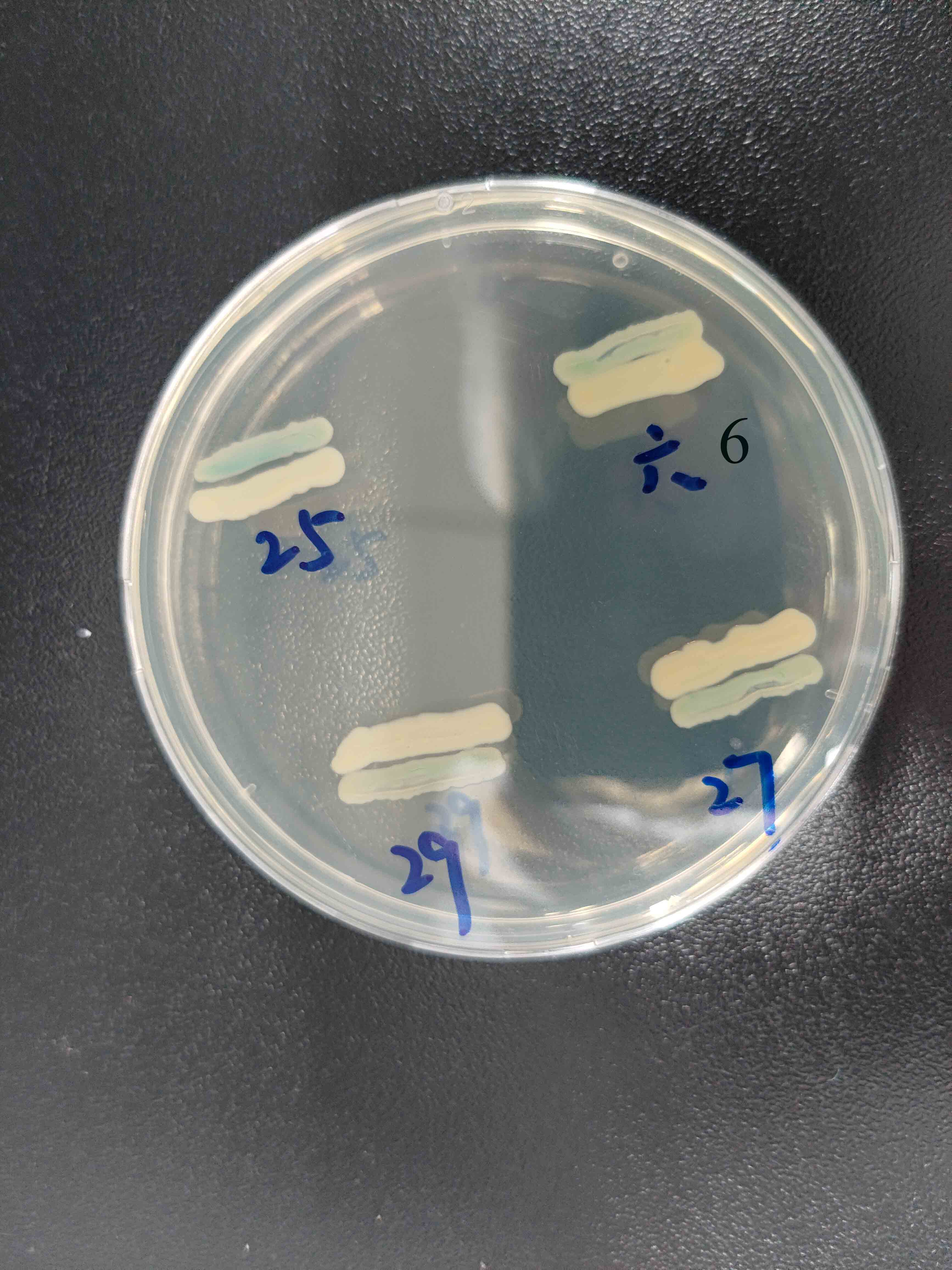

Supplement: Supplementary file 2 [file DataSheet2.ZIP › data/AHLs production of 60 clinical strains of A. baumannii/7.jpg]

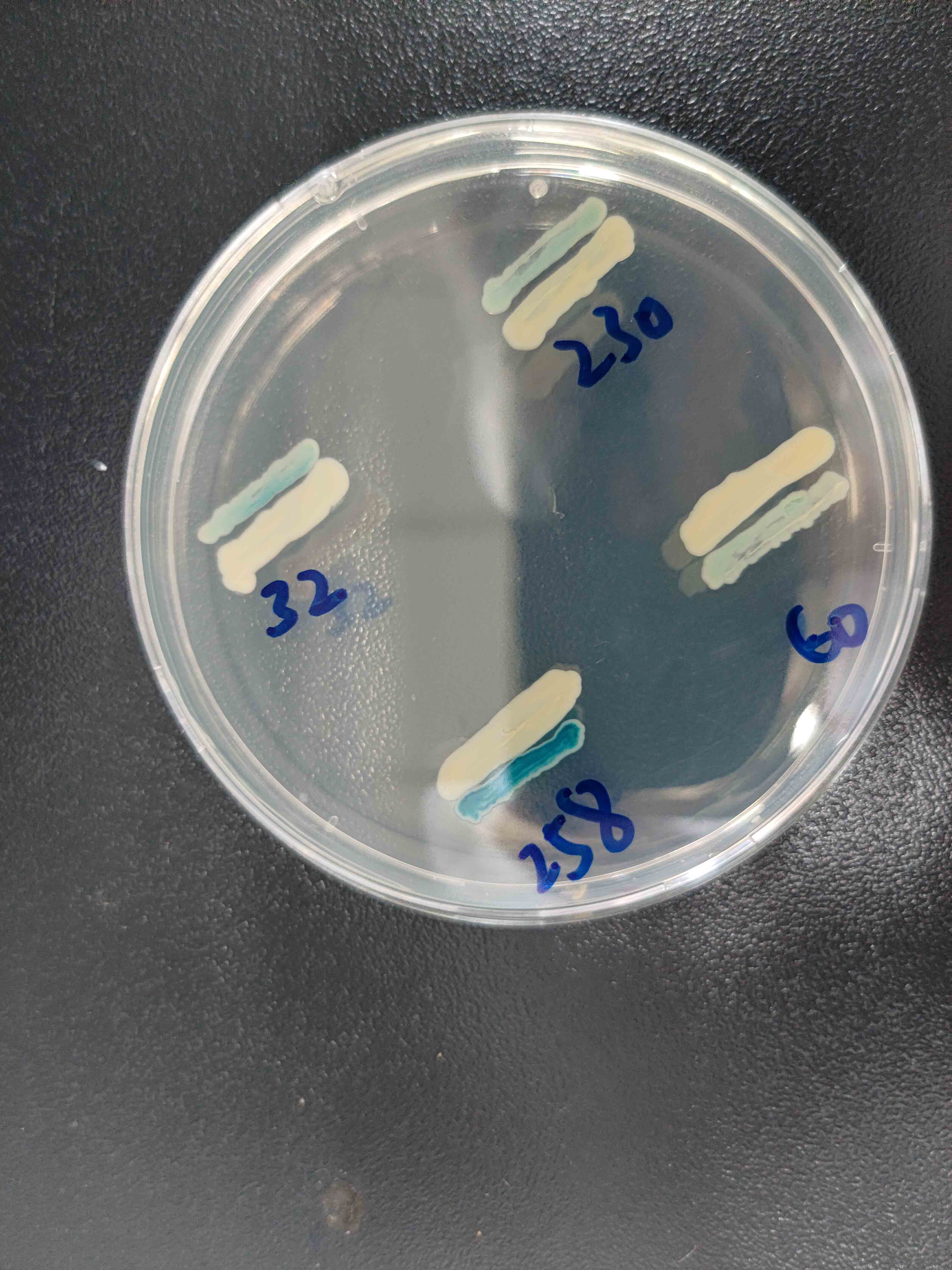

Supplement: Supplementary file 2 [file DataSheet2.ZIP › data/AHLs production of 60 clinical strains of A. baumannii/8.jpg]

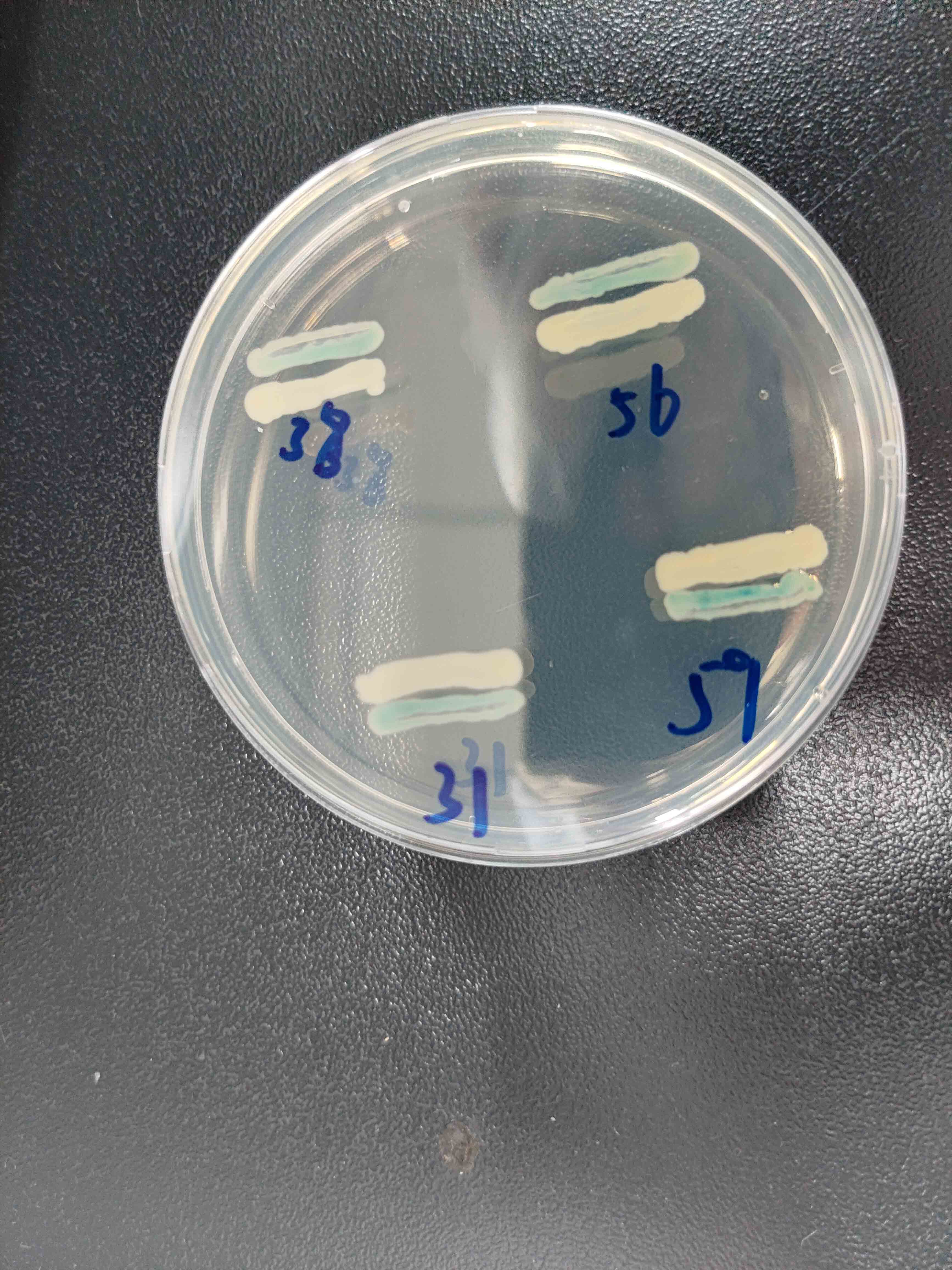

Supplement: Supplementary file 2 [file DataSheet2.ZIP › data/AHLs production of 60 clinical strains of A. baumannii/9.jpg]

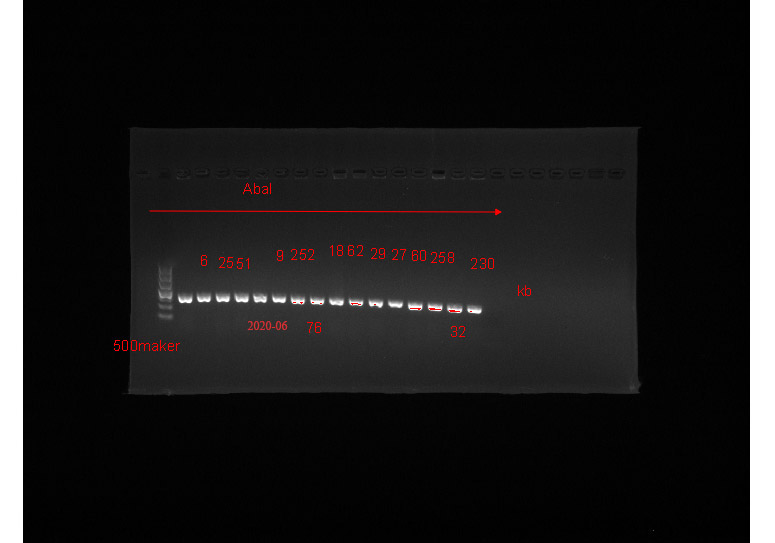

Supplement: Supplementary file 2 [file DataSheet2.ZIP › data/Expression of the gene abaIíóabaR in 60 clinical isolates of Acinetobacter baumannii/abaI(1).jpg]

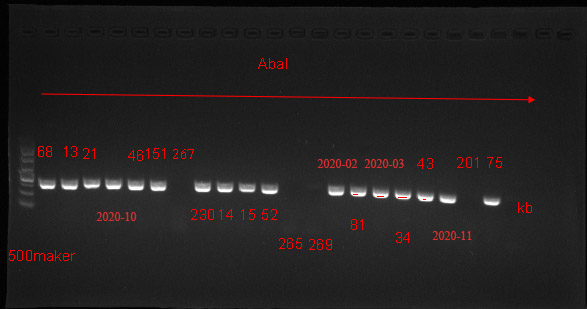

Supplement: Supplementary file 2 [file DataSheet2.ZIP › data/Expression of the gene abaIíóabaR in 60 clinical isolates of Acinetobacter baumannii/abaI(2).jpg]

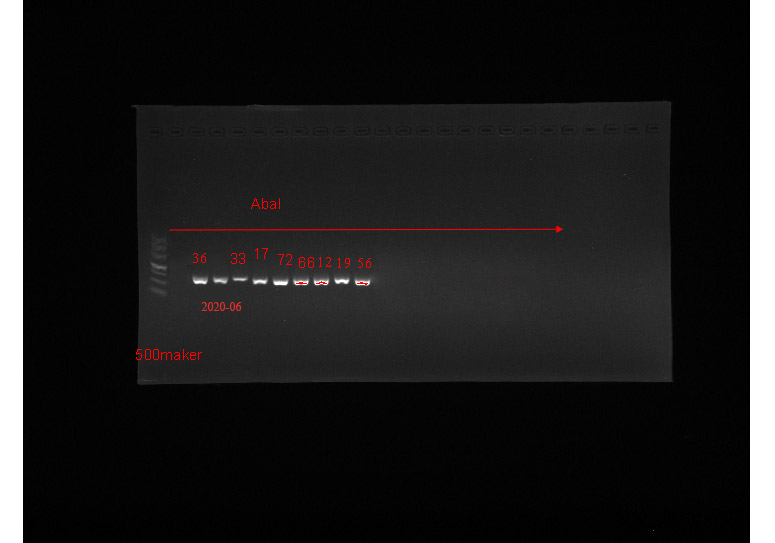

Supplement: Supplementary file 2 [file DataSheet2.ZIP › data/Expression of the gene abaIíóabaR in 60 clinical isolates of Acinetobacter baumannii/abaI(3).tif]

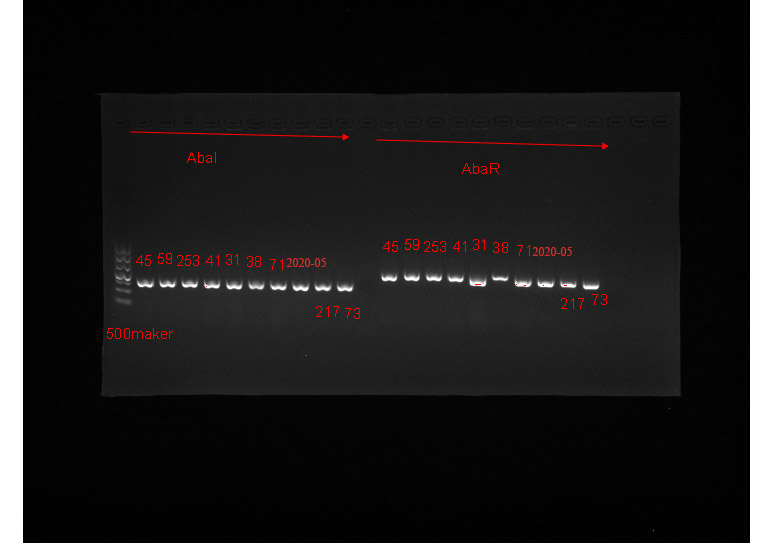

Supplement: Supplementary file 2 [file DataSheet2.ZIP › data/Expression of the gene abaIíóabaR in 60 clinical isolates of Acinetobacter baumannii/abaI,abaR.jpg]

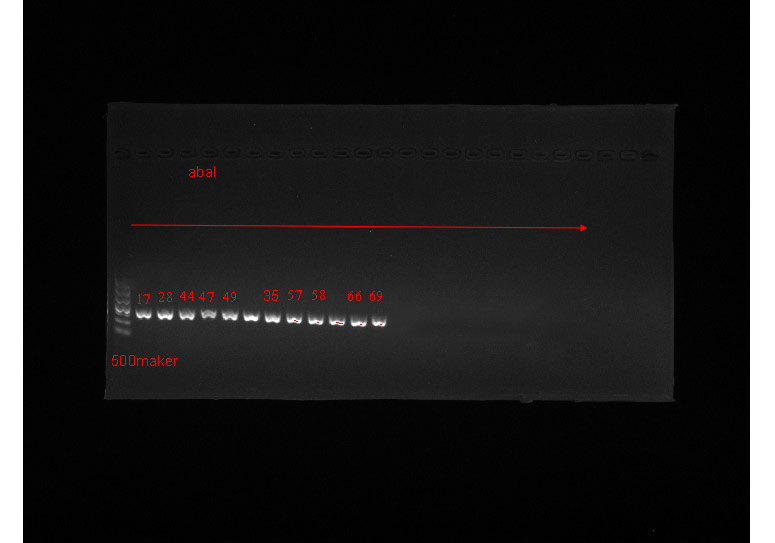

Supplement: Supplementary file 2 [file DataSheet2.ZIP › data/Expression of the gene abaIíóabaR in 60 clinical isolates of Acinetobacter baumannii/abaI.jpg]

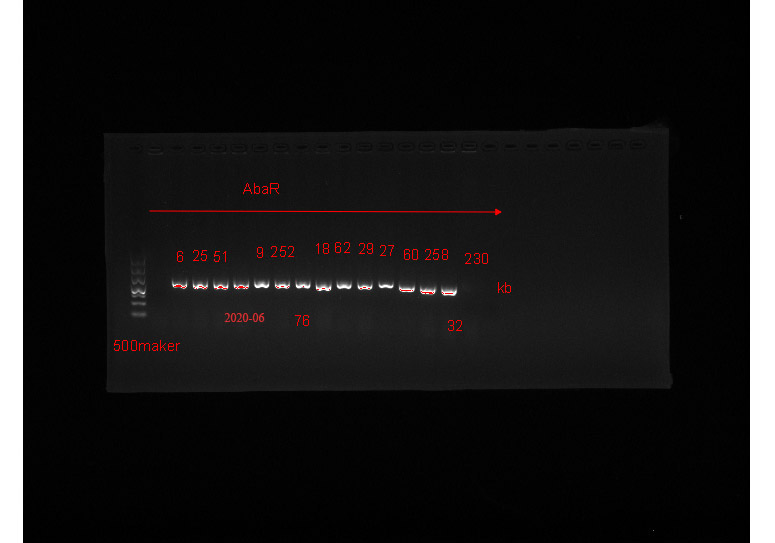

Supplement: Supplementary file 2 [file DataSheet2.ZIP › data/Expression of the gene abaIíóabaR in 60 clinical isolates of Acinetobacter baumannii/abaR(1).jpg]

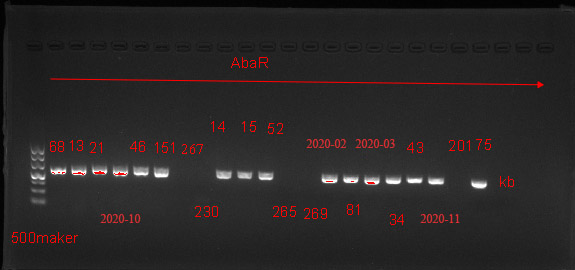

Supplement: Supplementary file 2 [file DataSheet2.ZIP › data/Expression of the gene abaIíóabaR in 60 clinical isolates of Acinetobacter baumannii/abaR(2).jpg]

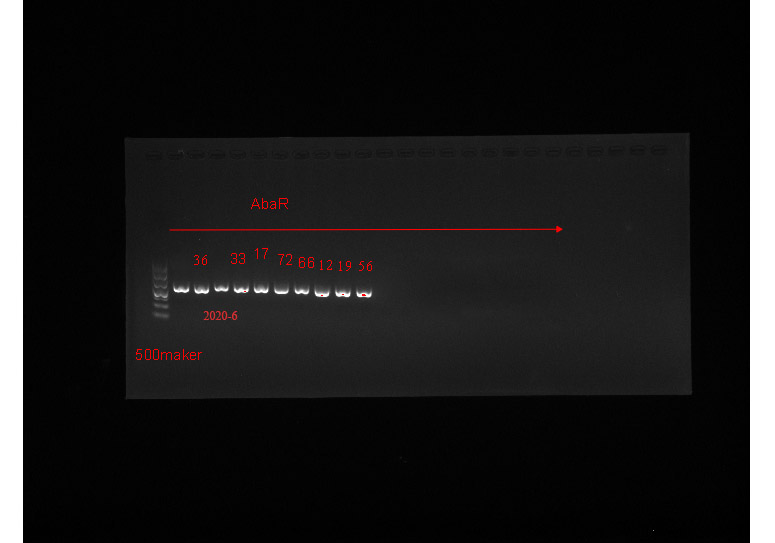

Supplement: Supplementary file 2 [file DataSheet2.ZIP › data/Expression of the gene abaIíóabaR in 60 clinical isolates of Acinetobacter baumannii/abaR(3).jpg]

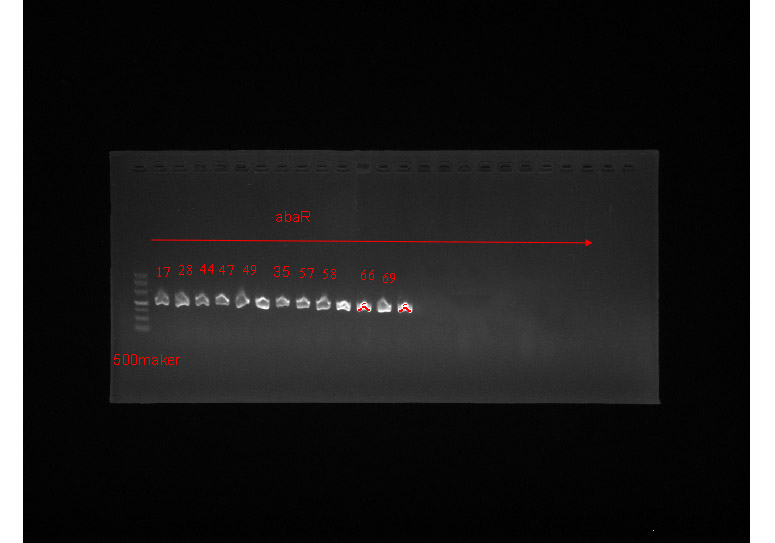

Supplement: Supplementary file 2 [file DataSheet2.ZIP › data/Expression of the gene abaIíóabaR in 60 clinical isolates of Acinetobacter baumannii/abaR.jpg]
