# Supplementary figures and images for: Effect of traditional Chinese medicine monomers interfering with quorum-sensing on virulence factors of extensively drug-resistant Acinetobacter baumannii (part 1 of 2)
Source: Front Pharmacol. 2023 Mar 30;14:1135180. doi: 10.3389/fphar.2023.1135180 (PMC10097947; doi:10.3389/fphar.2023.1135180)

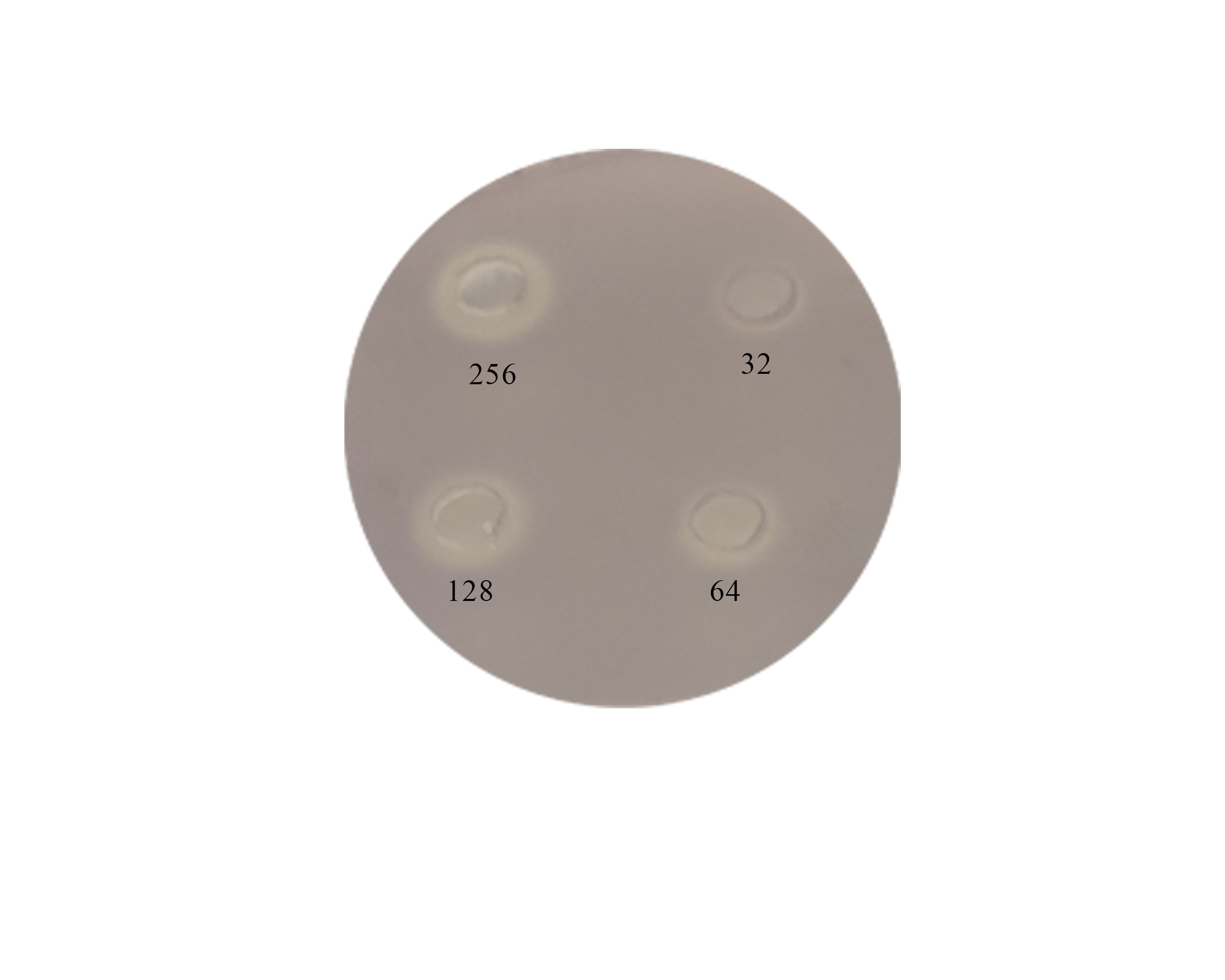

Supplement: Supplementary file 1 [file DataSheet1.zip › Data Sheet 1/Effect of 21TCMMs on QS activity of CV026/Effect of 21TCMMs on QS activity of CV026/4-Terpineol.jpg]

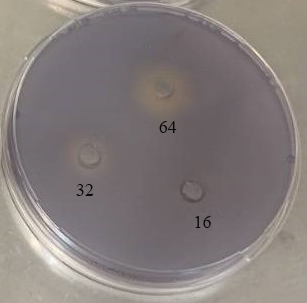

Supplement: Supplementary file 1 [file DataSheet1.zip › Data Sheet 1/Effect of 21TCMMs on QS activity of CV026/Effect of 21TCMMs on QS activity of CV026/Baicalein.tif]

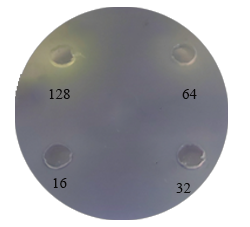

Supplement: Supplementary file 1 [file DataSheet1.zip › Data Sheet 1/Effect of 21TCMMs on QS activity of CV026/Effect of 21TCMMs on QS activity of CV026/Berberine.tif]

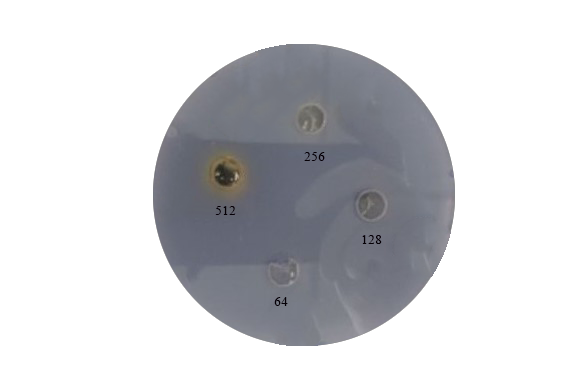

Supplement: Supplementary file 1 [file DataSheet1.zip › Data Sheet 1/Effect of 21TCMMs on QS activity of CV026/Effect of 21TCMMs on QS activity of CV026/Caffeic acid.tif]

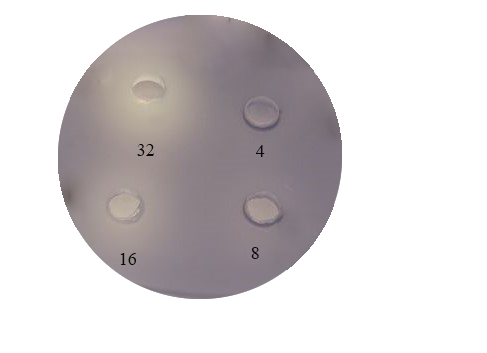

Supplement: Supplementary file 1 [file DataSheet1.zip › Data Sheet 1/Effect of 21TCMMs on QS activity of CV026/Effect of 21TCMMs on QS activity of CV026/Carvacrol.tif]

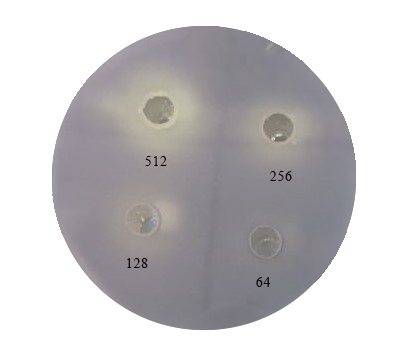

Supplement: Supplementary file 1 [file DataSheet1.zip › Data Sheet 1/Effect of 21TCMMs on QS activity of CV026/Effect of 21TCMMs on QS activity of CV026/Cinnamic acid.tif]

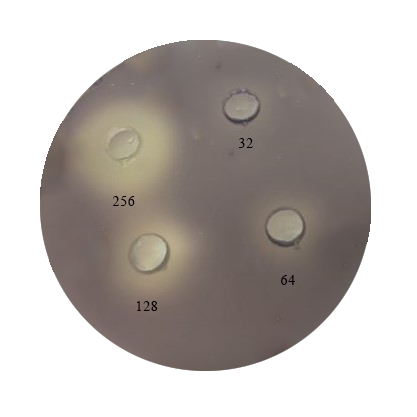

Supplement: Supplementary file 1 [file DataSheet1.zip › Data Sheet 1/Effect of 21TCMMs on QS activity of CV026/Effect of 21TCMMs on QS activity of CV026/Coumarin.tif]

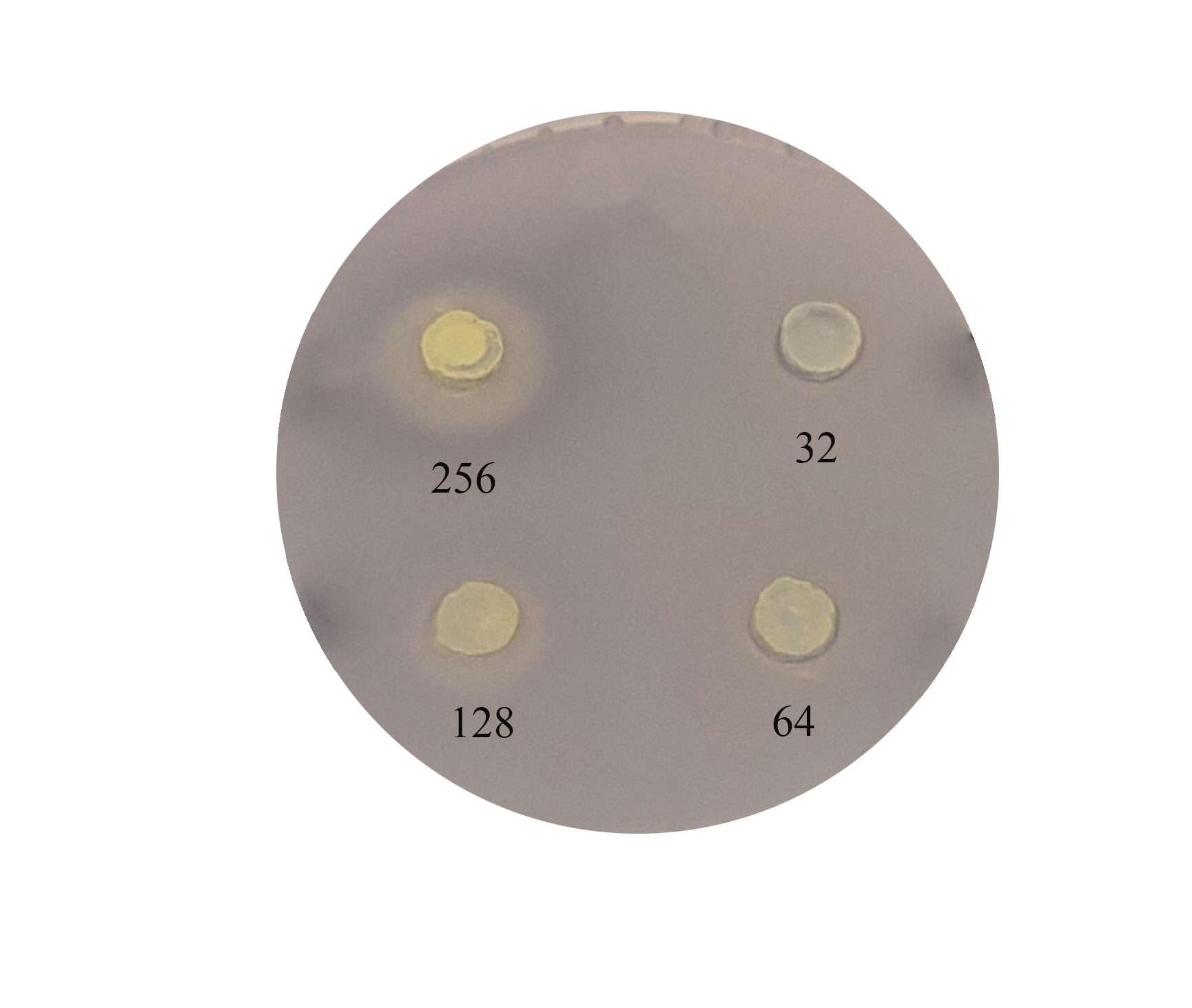

Supplement: Supplementary file 1 [file DataSheet1.zip › Data Sheet 1/Effect of 21TCMMs on QS activity of CV026/Effect of 21TCMMs on QS activity of CV026/Ellagic acid.jpg]

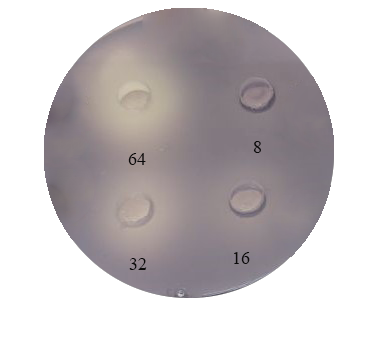

Supplement: Supplementary file 1 [file DataSheet1.zip › Data Sheet 1/Effect of 21TCMMs on QS activity of CV026/Effect of 21TCMMs on QS activity of CV026/Eugenol.tif]

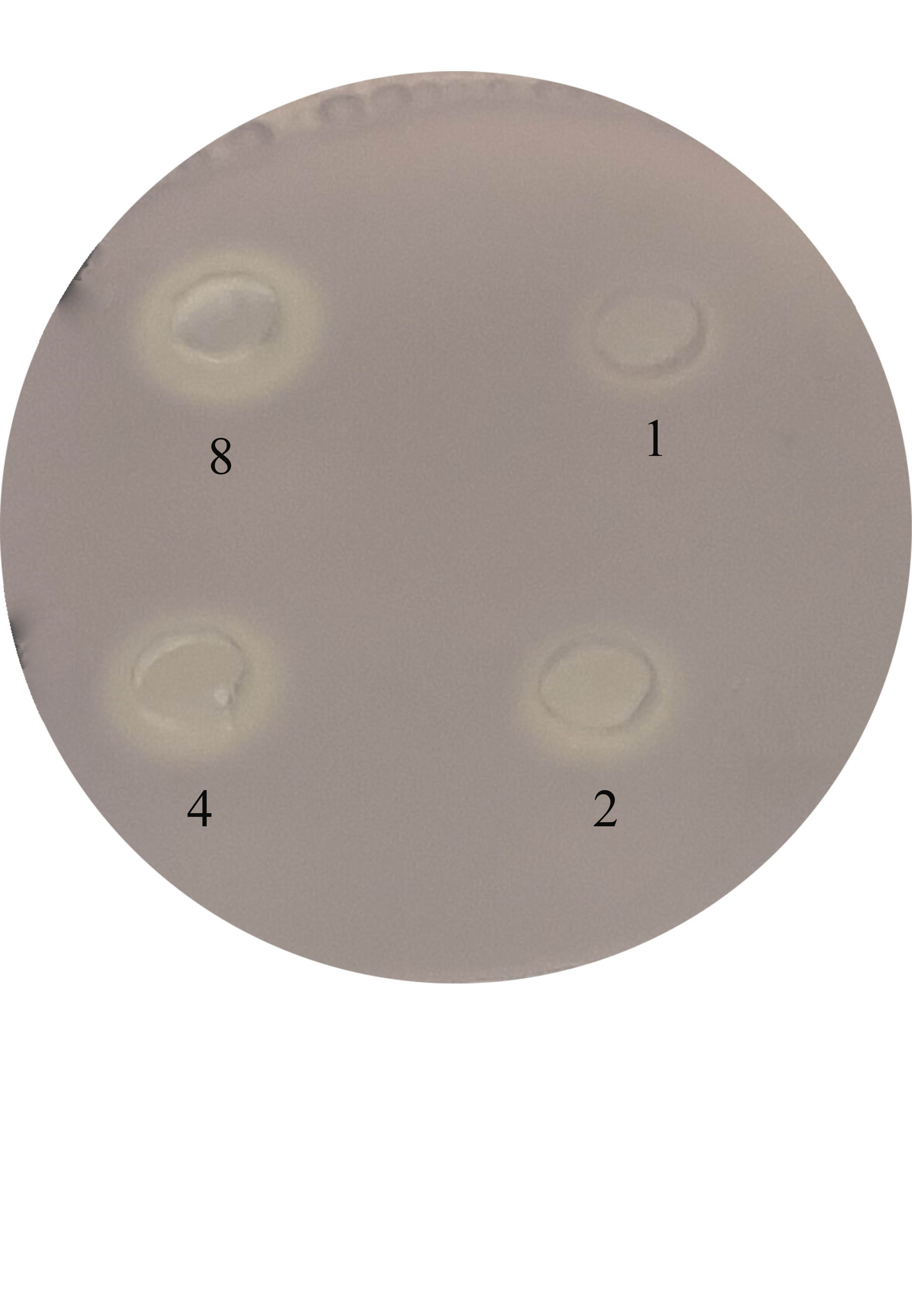

Supplement: Supplementary file 1 [file DataSheet1.zip › Data Sheet 1/Effect of 21TCMMs on QS activity of CV026/Effect of 21TCMMs on QS activity of CV026/Furanone C30.jpg]

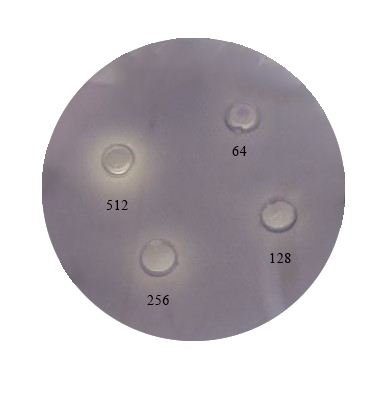

Supplement: Supplementary file 1 [file DataSheet1.zip › Data Sheet 1/Effect of 21TCMMs on QS activity of CV026/Effect of 21TCMMs on QS activity of CV026/Hordenine.tif]

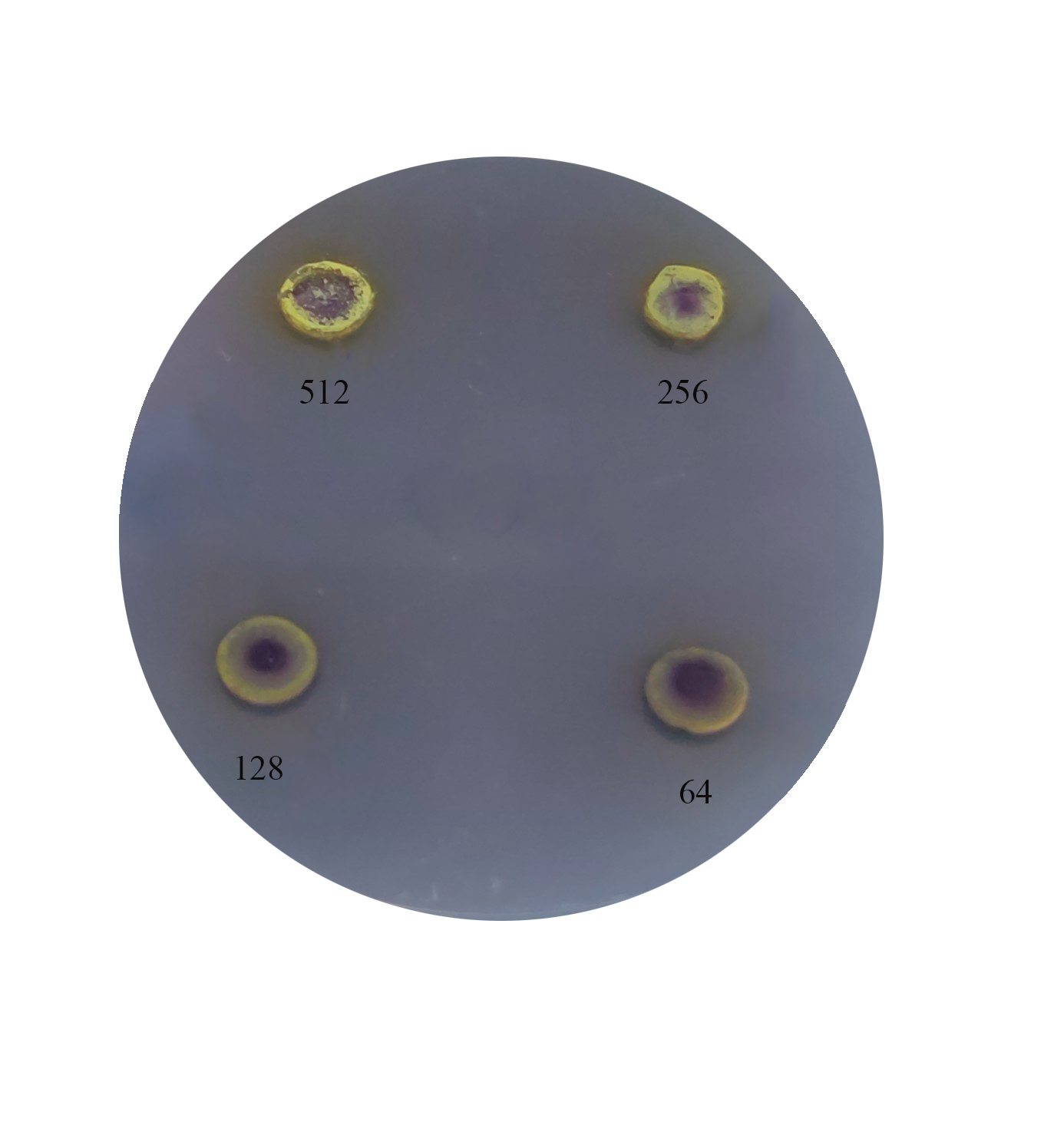

Supplement: Supplementary file 1 [file DataSheet1.zip › Data Sheet 1/Effect of 21TCMMs on QS activity of CV026/Effect of 21TCMMs on QS activity of CV026/Kaempferol.jpg]

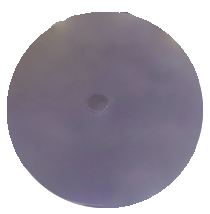

Supplement: Supplementary file 1 [file DataSheet1.zip › Data Sheet 1/Effect of 21TCMMs on QS activity of CV026/Effect of 21TCMMs on QS activity of CV026/kb.tif]

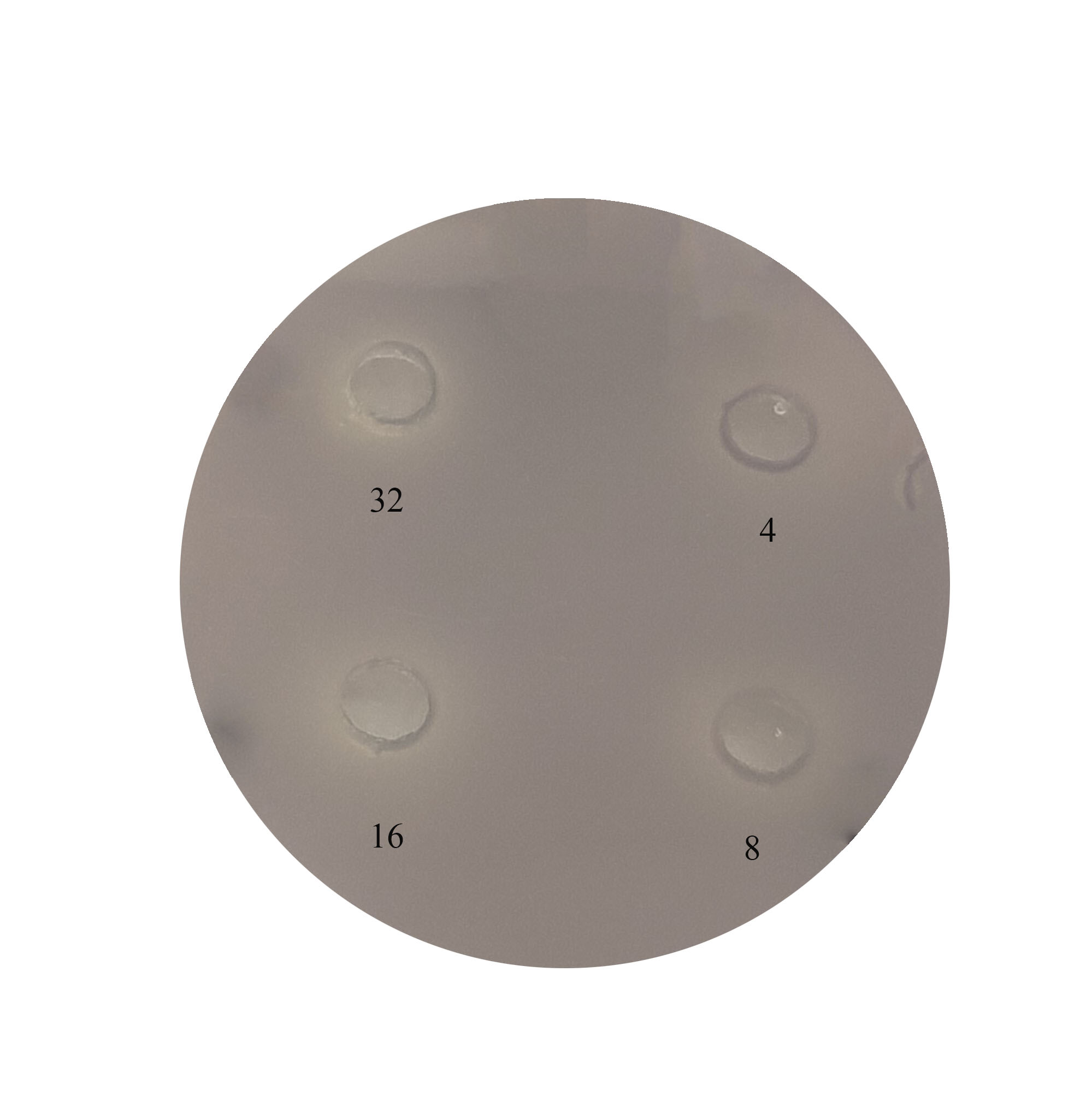

Supplement: Supplementary file 1 [file DataSheet1.zip › Data Sheet 1/Effect of 21TCMMs on QS activity of CV026/Effect of 21TCMMs on QS activity of CV026/Magnolol.jpg]

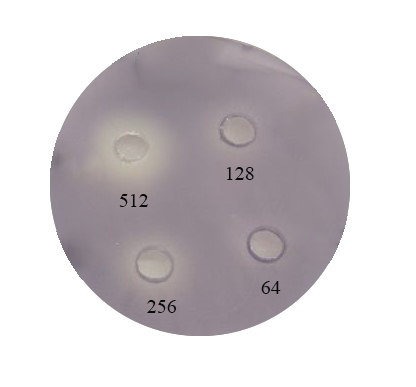

Supplement: Supplementary file 1 [file DataSheet1.zip › Data Sheet 1/Effect of 21TCMMs on QS activity of CV026/Effect of 21TCMMs on QS activity of CV026/Matrine.tif]

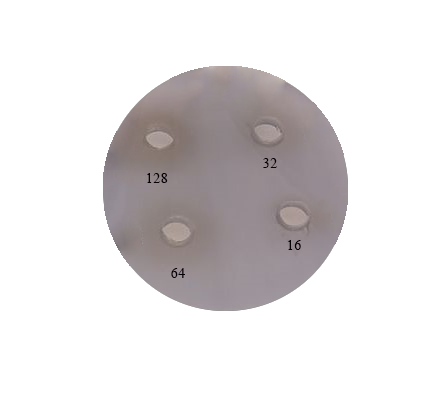

Supplement: Supplementary file 1 [file DataSheet1.zip › Data Sheet 1/Effect of 21TCMMs on QS activity of CV026/Effect of 21TCMMs on QS activity of CV026/Myricetin.tif]

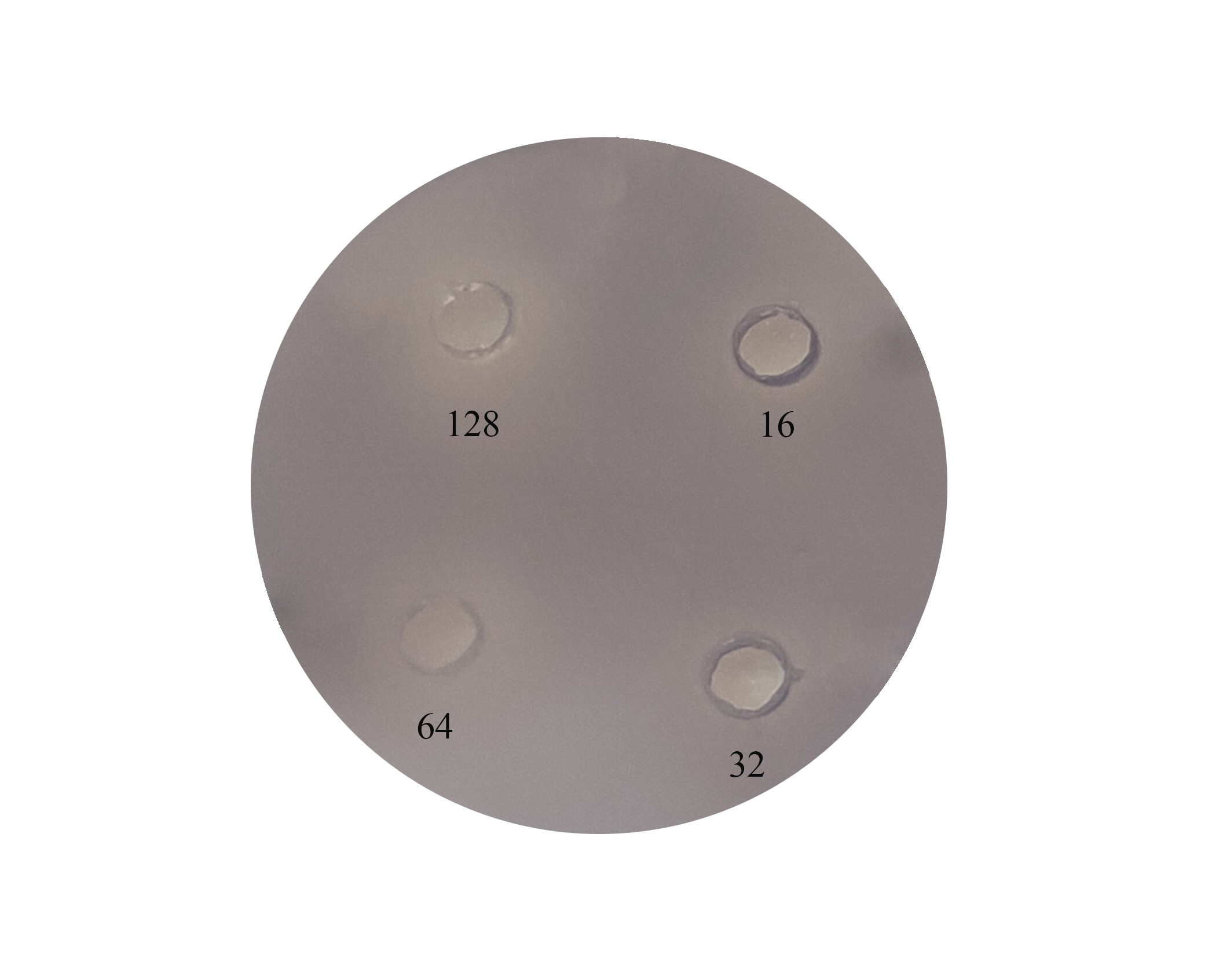

Supplement: Supplementary file 1 [file DataSheet1.zip › Data Sheet 1/Effect of 21TCMMs on QS activity of CV026/Effect of 21TCMMs on QS activity of CV026/Naringenin.jpg]

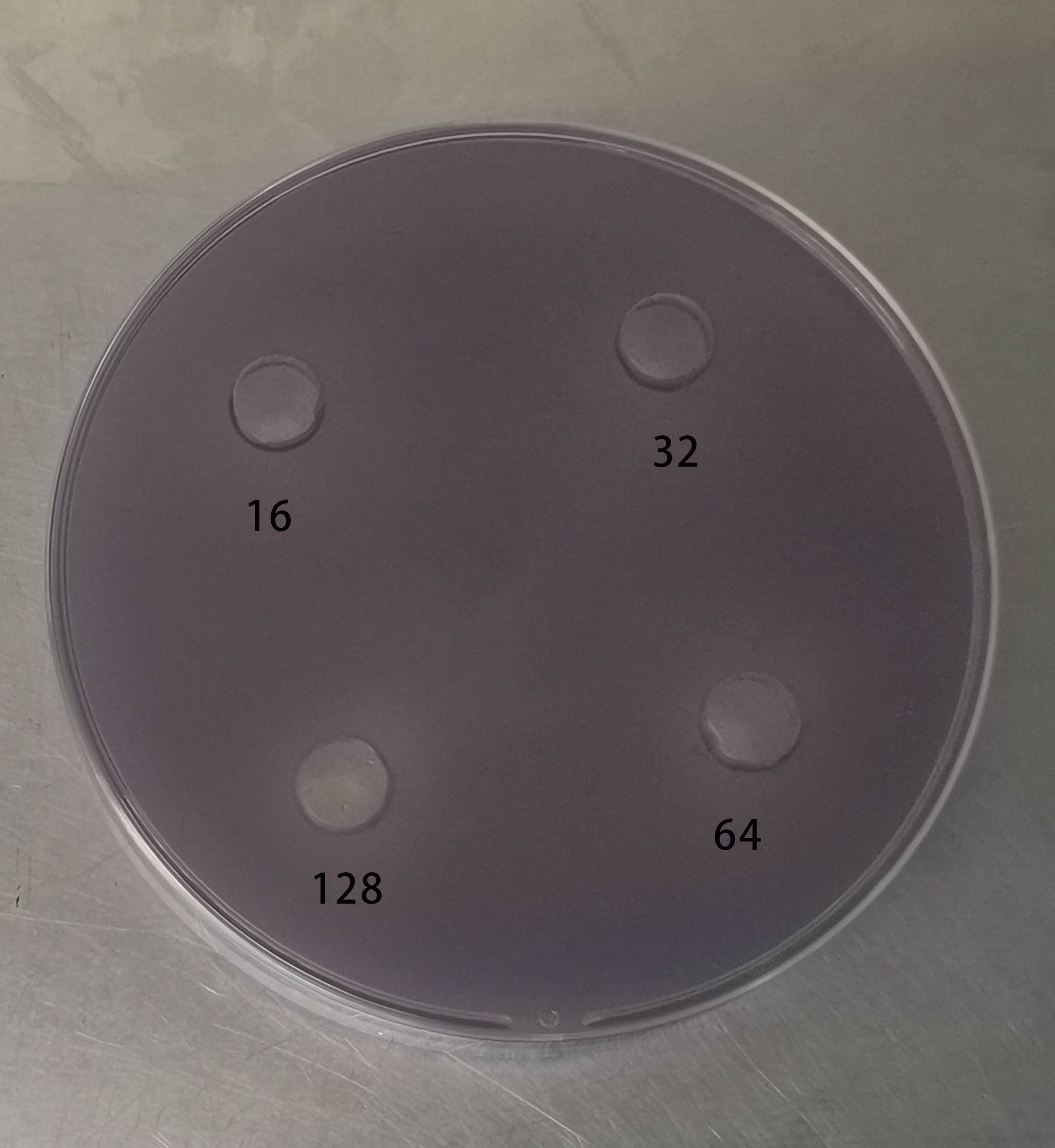

Supplement: Supplementary file 1 [file DataSheet1.zip › Data Sheet 1/Effect of 21TCMMs on QS activity of CV026/Effect of 21TCMMs on QS activity of CV026/Nootkatone.jpg]

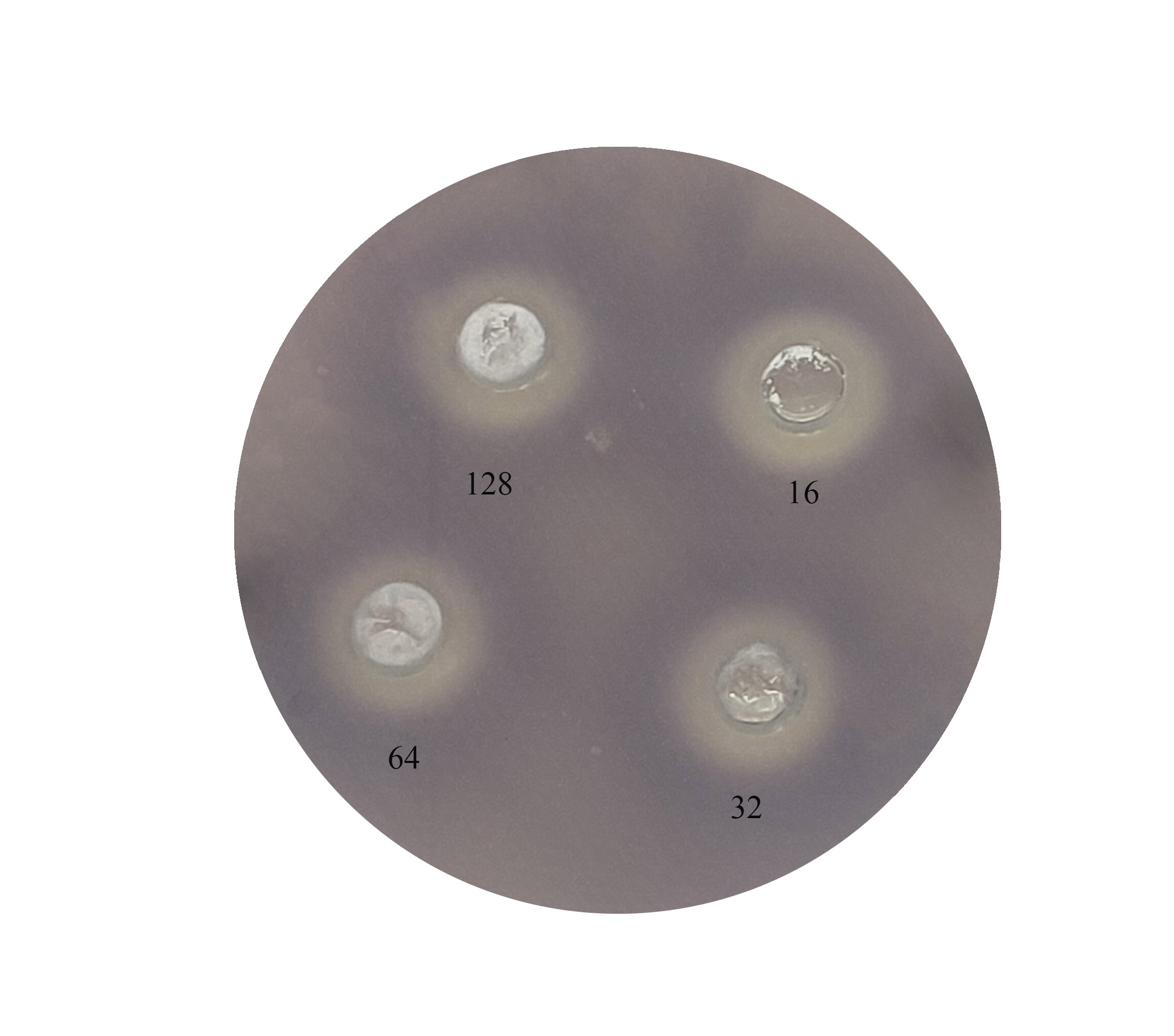

Supplement: Supplementary file 1 [file DataSheet1.zip › Data Sheet 1/Effect of 21TCMMs on QS activity of CV026/Effect of 21TCMMs on QS activity of CV026/Osthole.jpg]

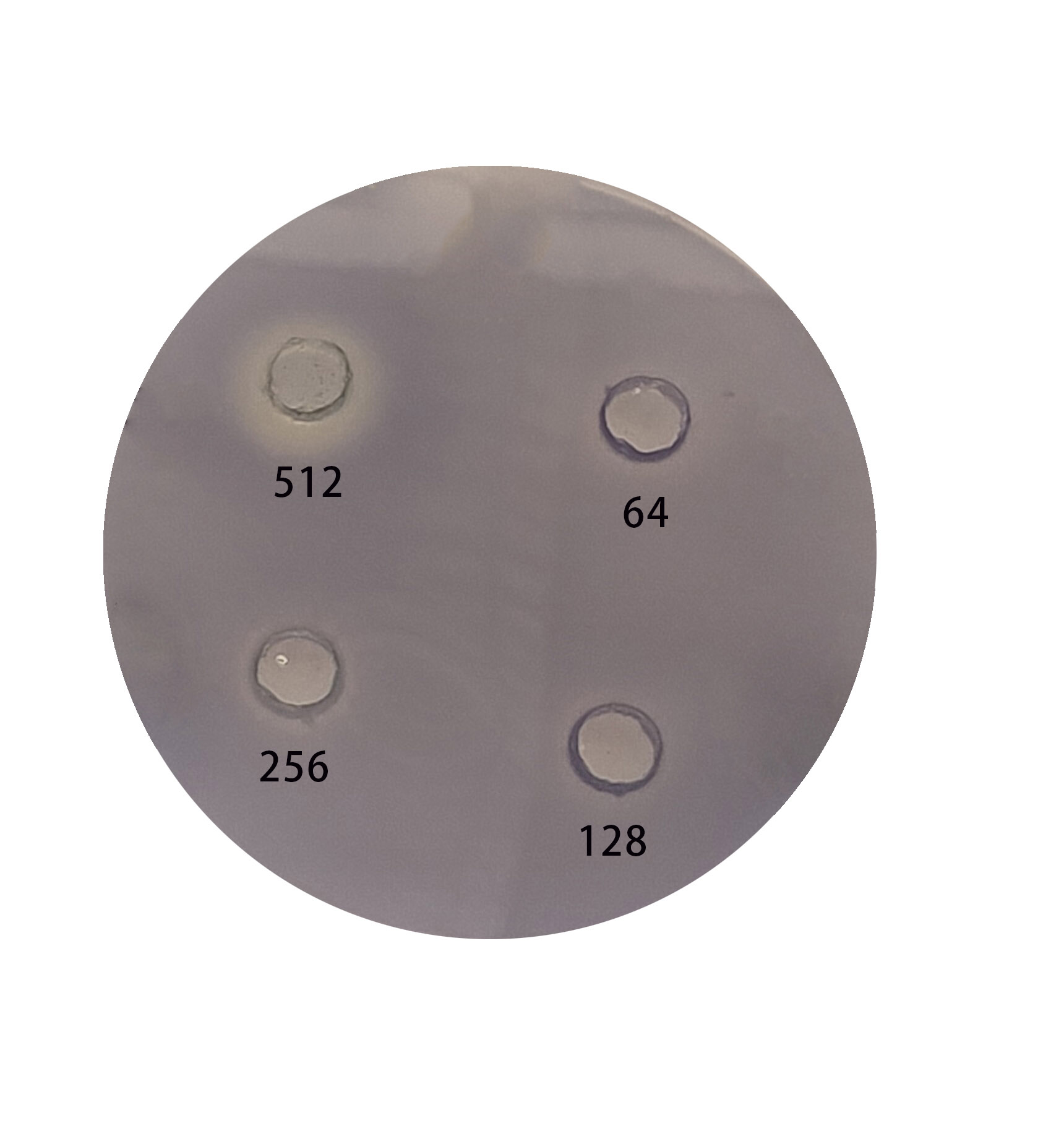

Supplement: Supplementary file 1 [file DataSheet1.zip › Data Sheet 1/Effect of 21TCMMs on QS activity of CV026/Effect of 21TCMMs on QS activity of CV026/Parthenolide.jpg]

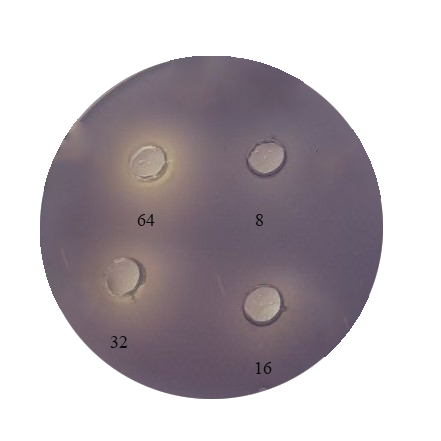

Supplement: Supplementary file 1 [file DataSheet1.zip › Data Sheet 1/Effect of 21TCMMs on QS activity of CV026/Effect of 21TCMMs on QS activity of CV026/Phloretin.tif]

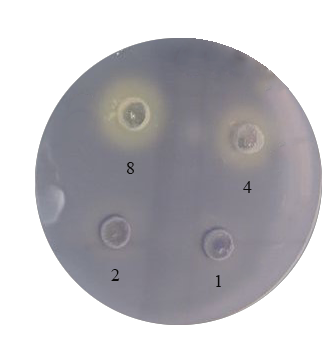

Supplement: Supplementary file 1 [file DataSheet1.zip › Data Sheet 1/Effect of 21TCMMs on QS activity of CV026/Effect of 21TCMMs on QS activity of CV026/Resveratrol.tif]

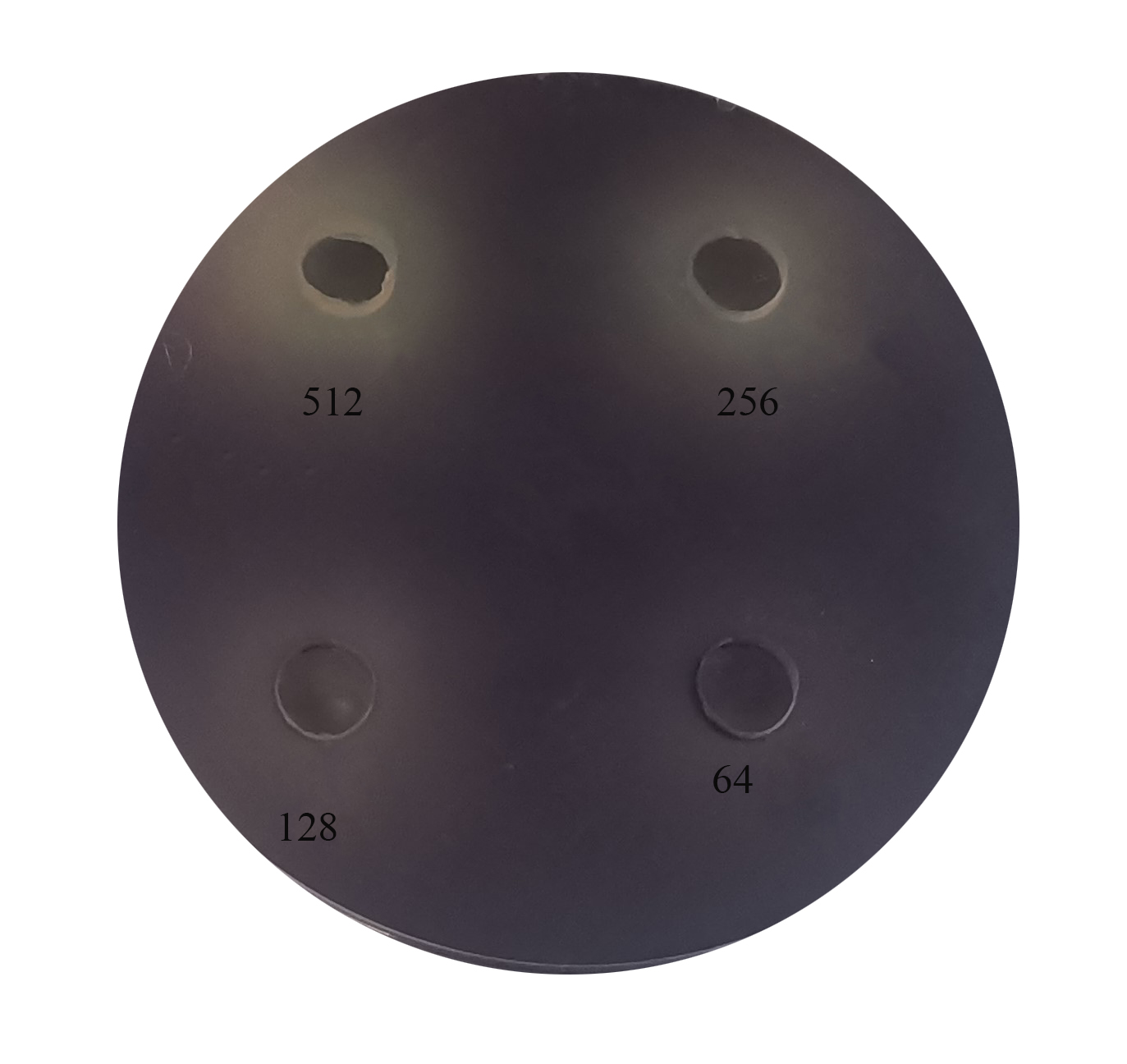

Supplement: Supplementary file 1 [file DataSheet1.zip › Data Sheet 1/Effect of 21TCMMs on QS activity of CV026/Effect of 21TCMMs on QS activity of CV026/Vanillin.jpg]

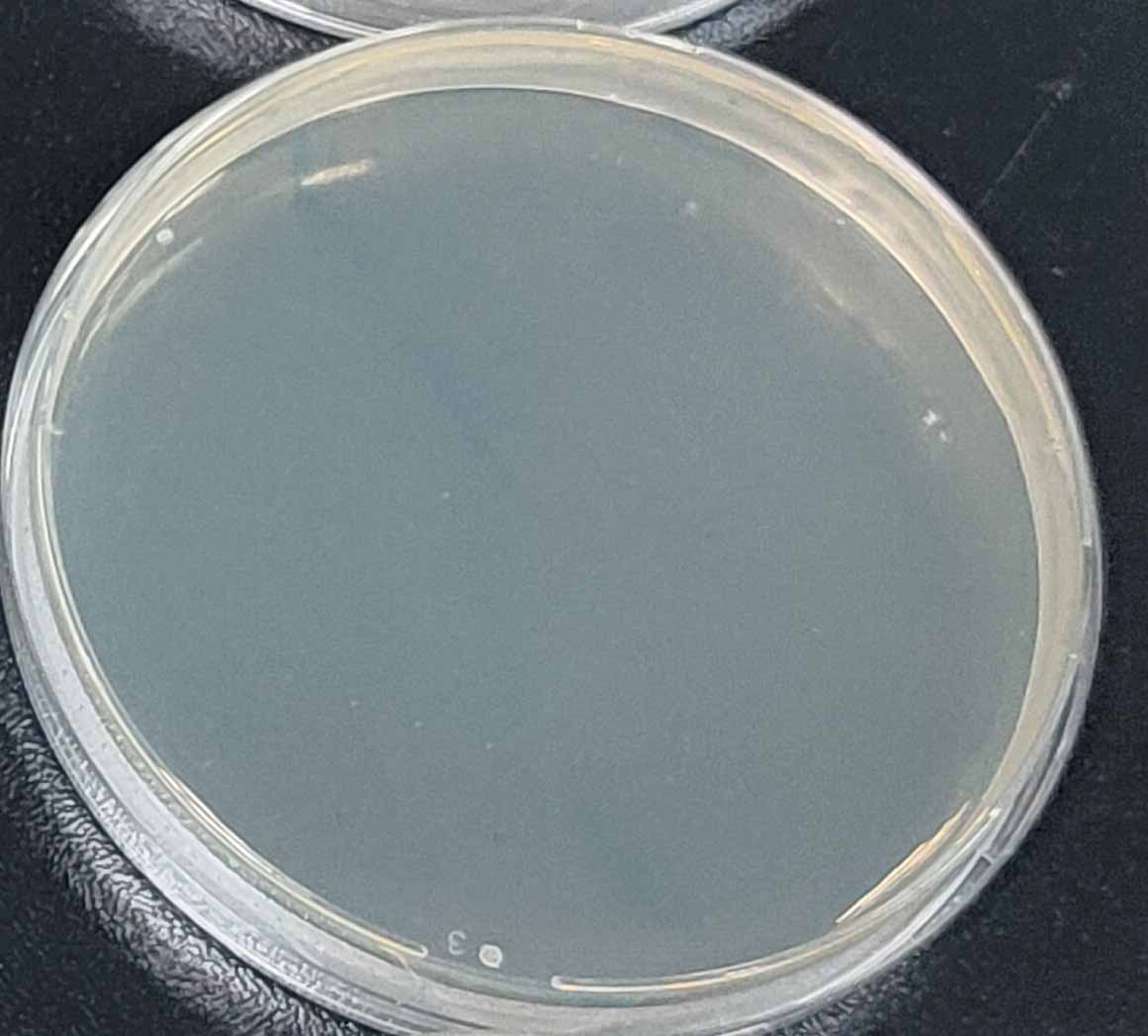

Supplement: Supplementary file 1 [file DataSheet1.zip › Data Sheet 1/Effect of 9 TCMMs on the adhesion ability of XDRAB/adhesion data/4-Terpineol/0 (2).jpg]

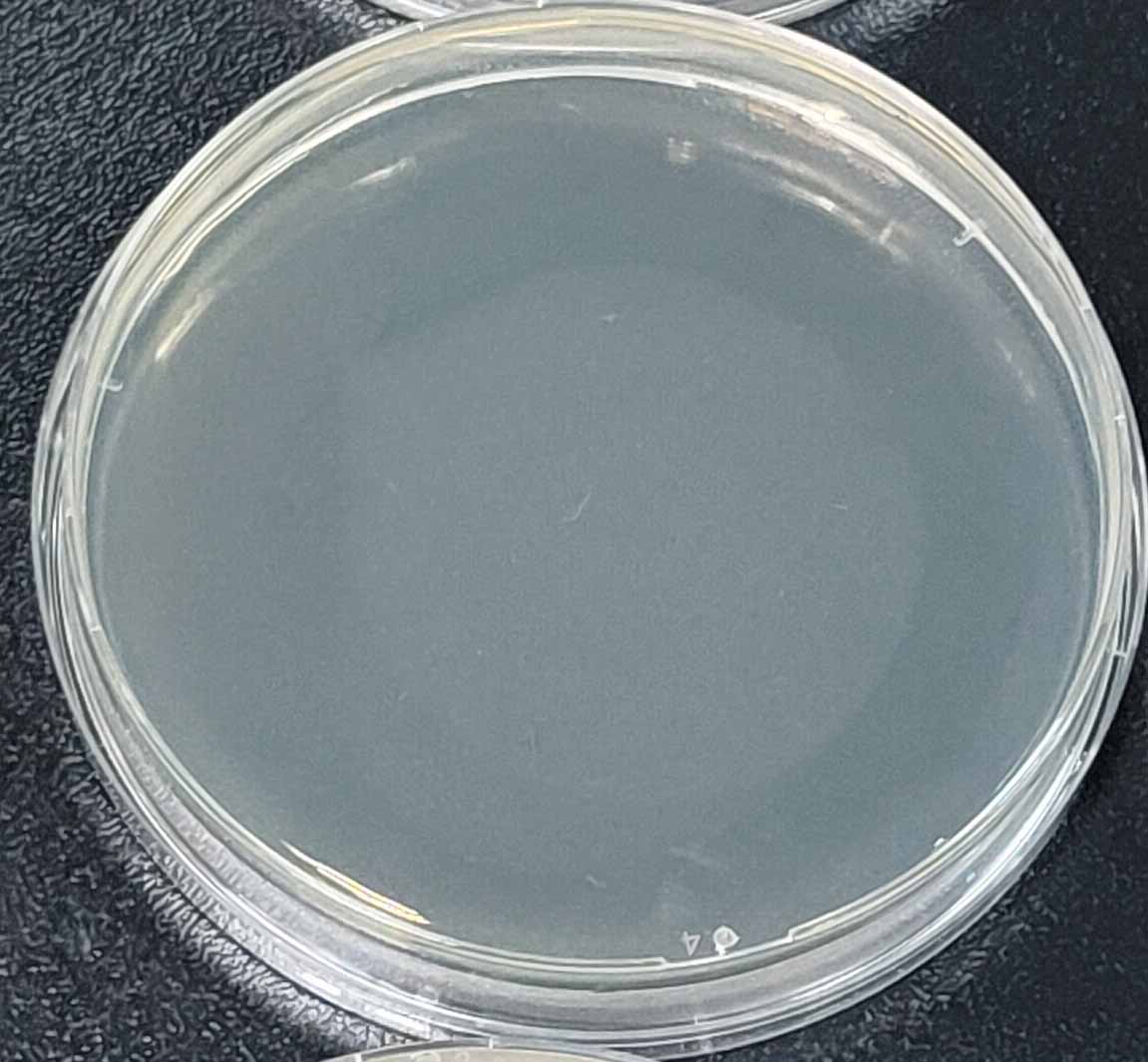

Supplement: Supplementary file 1 [file DataSheet1.zip › Data Sheet 1/Effect of 9 TCMMs on the adhesion ability of XDRAB/adhesion data/4-Terpineol/0 (3).jpg]

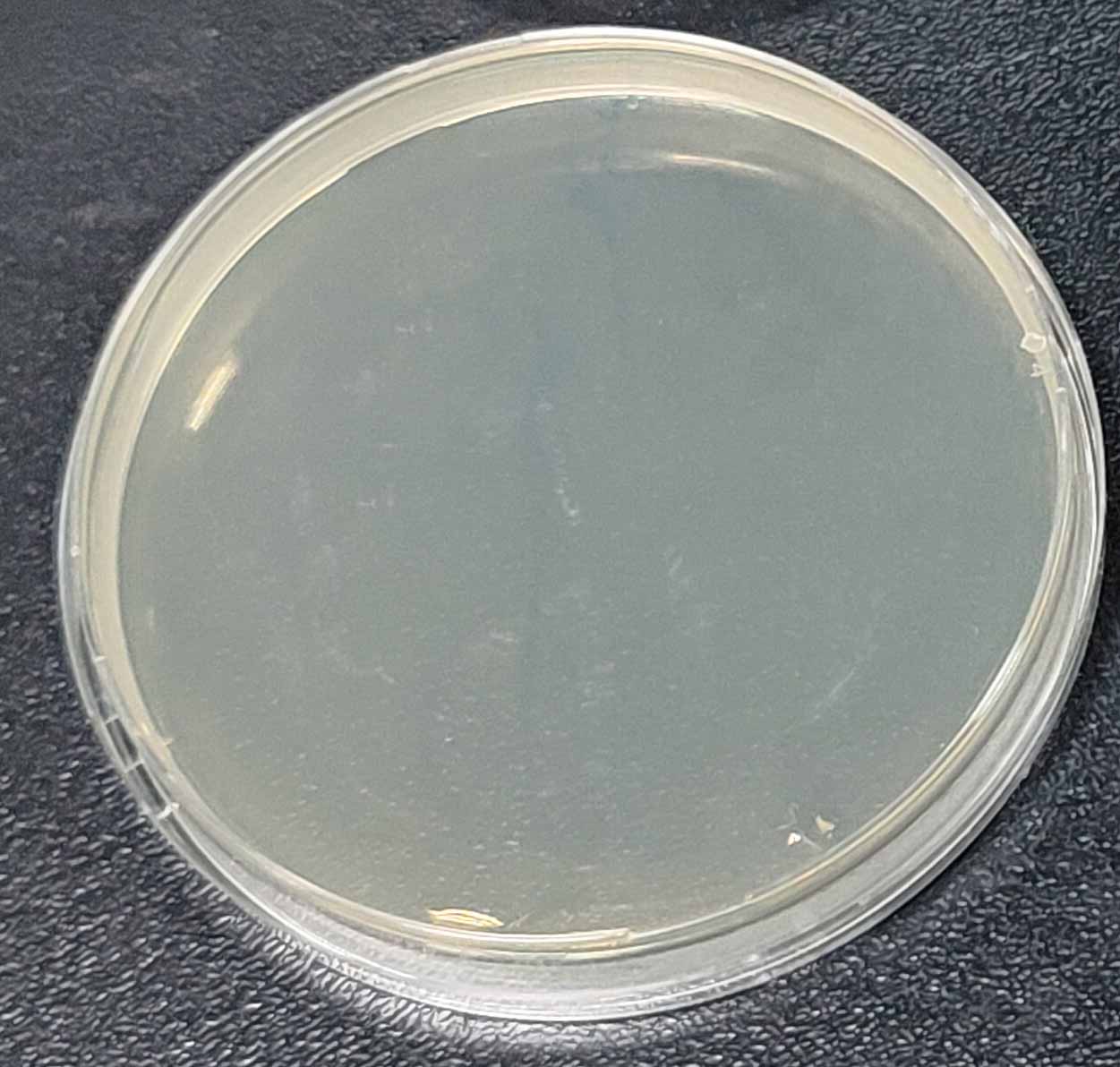

Supplement: Supplementary file 1 [file DataSheet1.zip › Data Sheet 1/Effect of 9 TCMMs on the adhesion ability of XDRAB/adhesion data/4-Terpineol/0 (4).jpg]

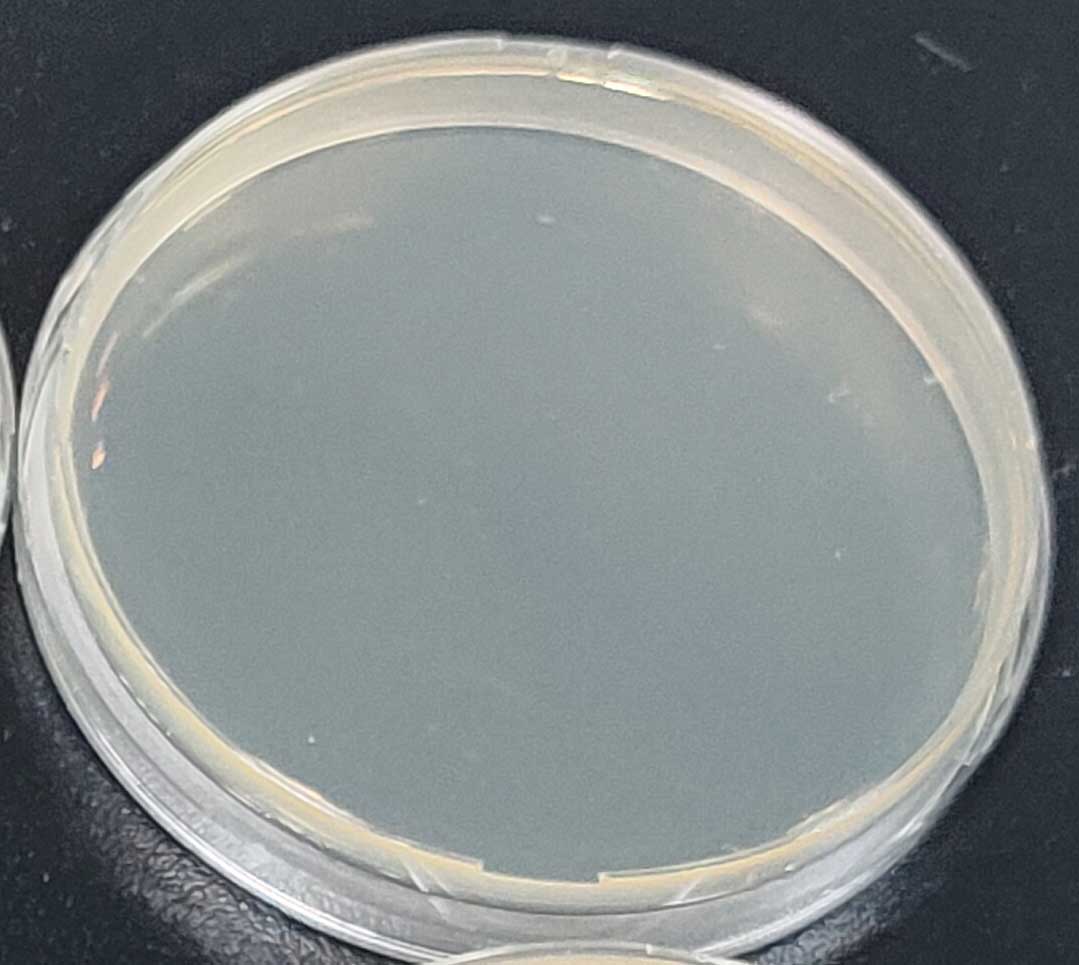

Supplement: Supplementary file 1 [file DataSheet1.zip › Data Sheet 1/Effect of 9 TCMMs on the adhesion ability of XDRAB/adhesion data/4-Terpineol/0 (5).jpg]

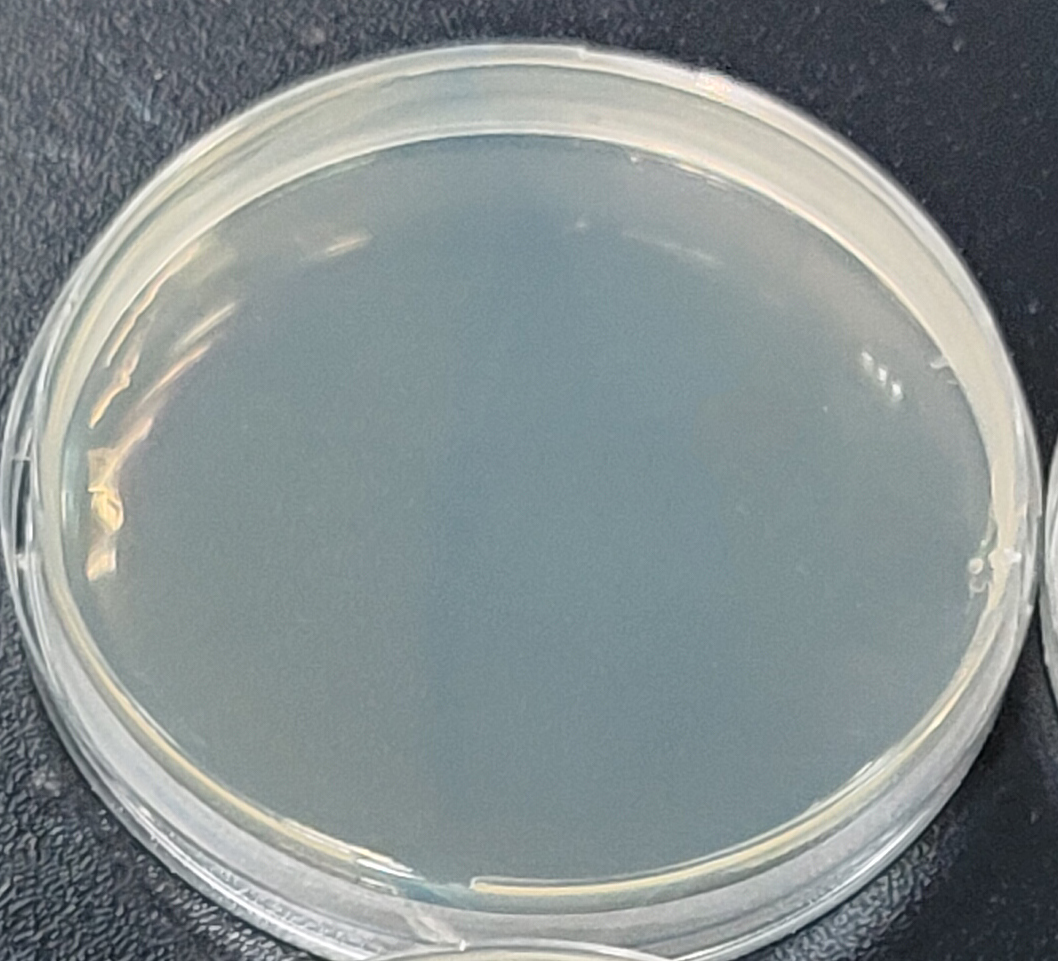

Supplement: Supplementary file 1 [file DataSheet1.zip › Data Sheet 1/Effect of 9 TCMMs on the adhesion ability of XDRAB/adhesion data/4-Terpineol/0 (6).jpg]

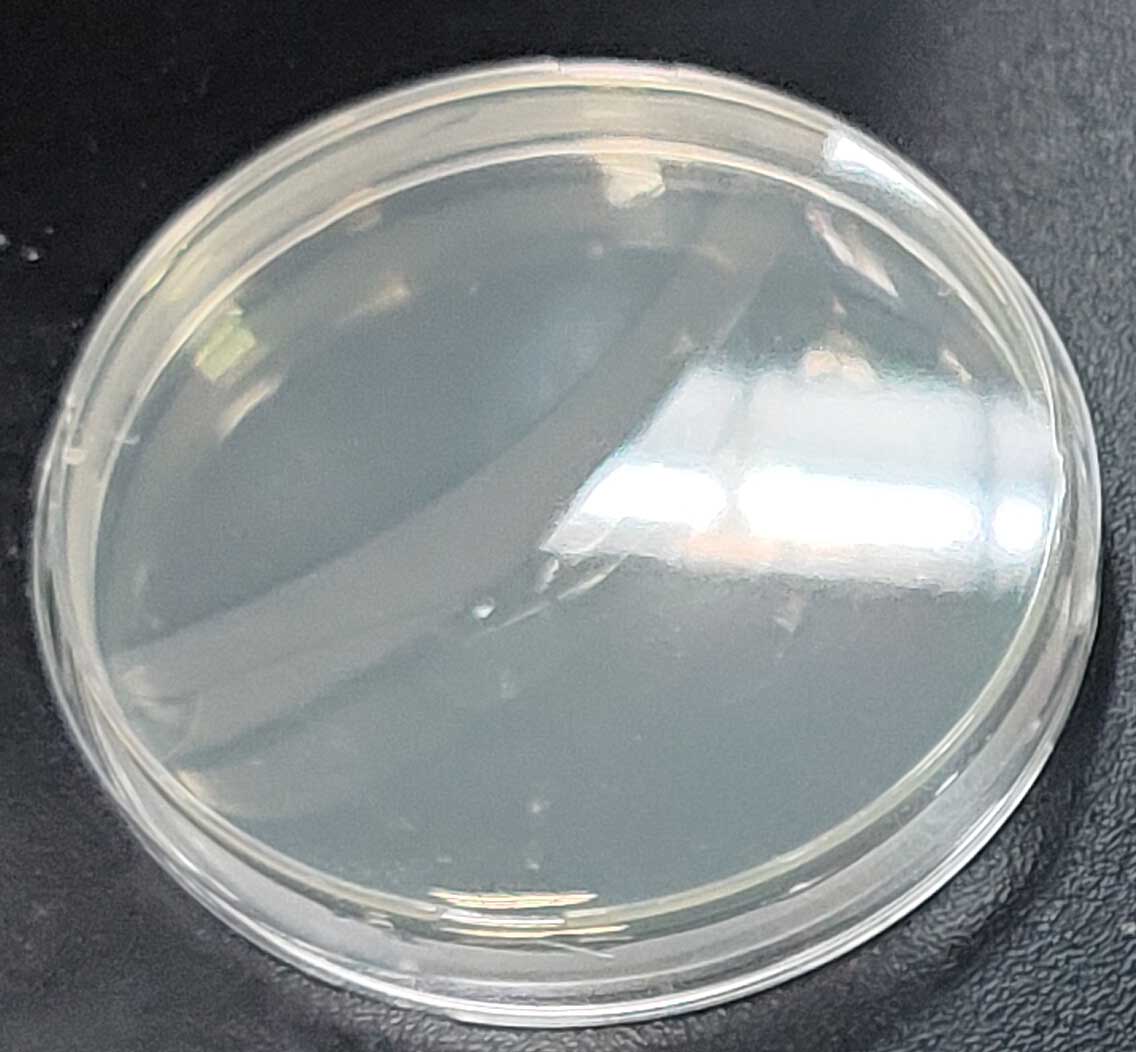

Supplement: Supplementary file 1 [file DataSheet1.zip › Data Sheet 1/Effect of 9 TCMMs on the adhesion ability of XDRAB/adhesion data/4-Terpineol/0 (7).jpg]

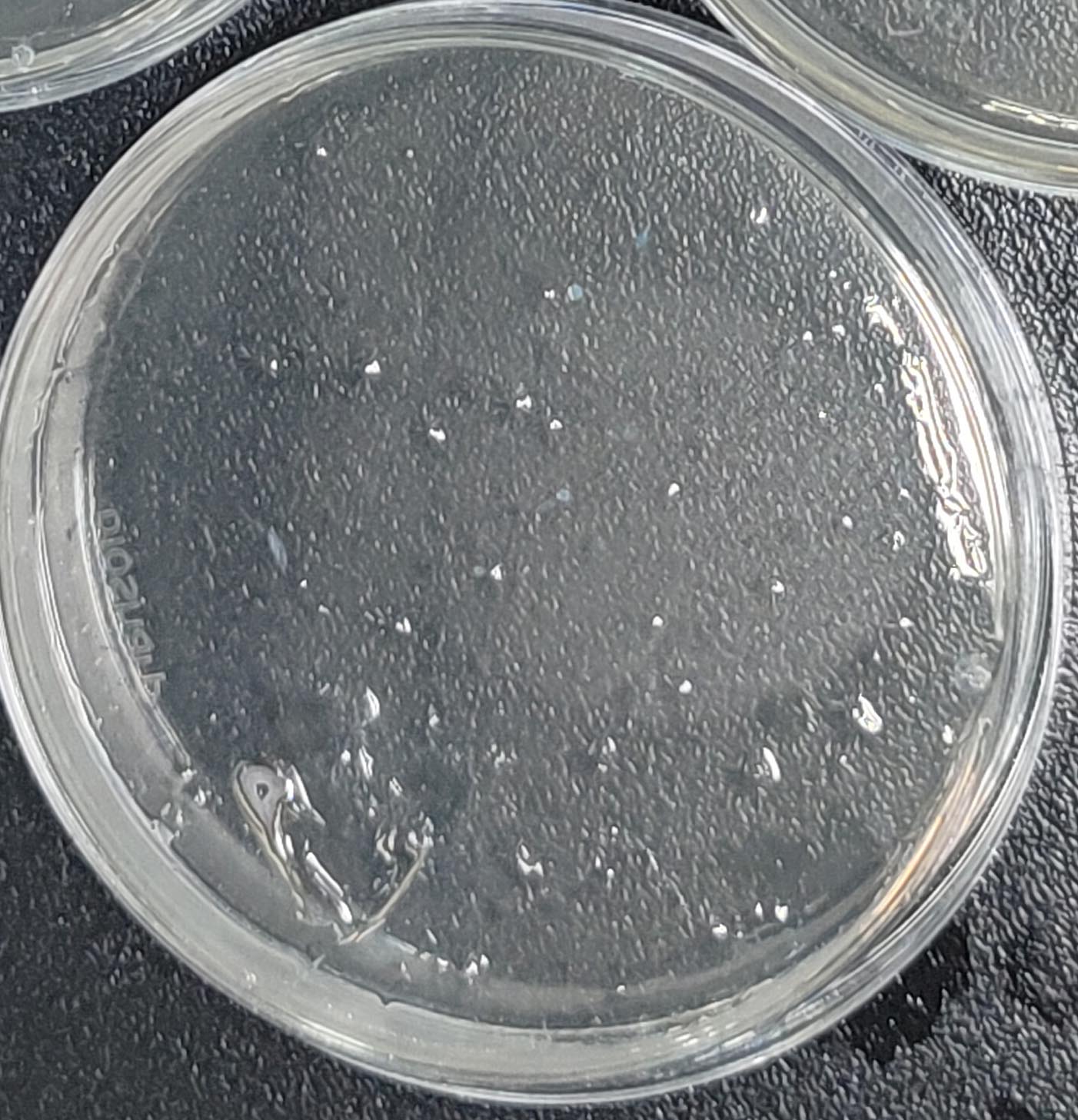

Supplement: Supplementary file 1 [file DataSheet1.zip › Data Sheet 1/Effect of 9 TCMMs on the adhesion ability of XDRAB/adhesion data/4-Terpineol/0 (8).jpg]

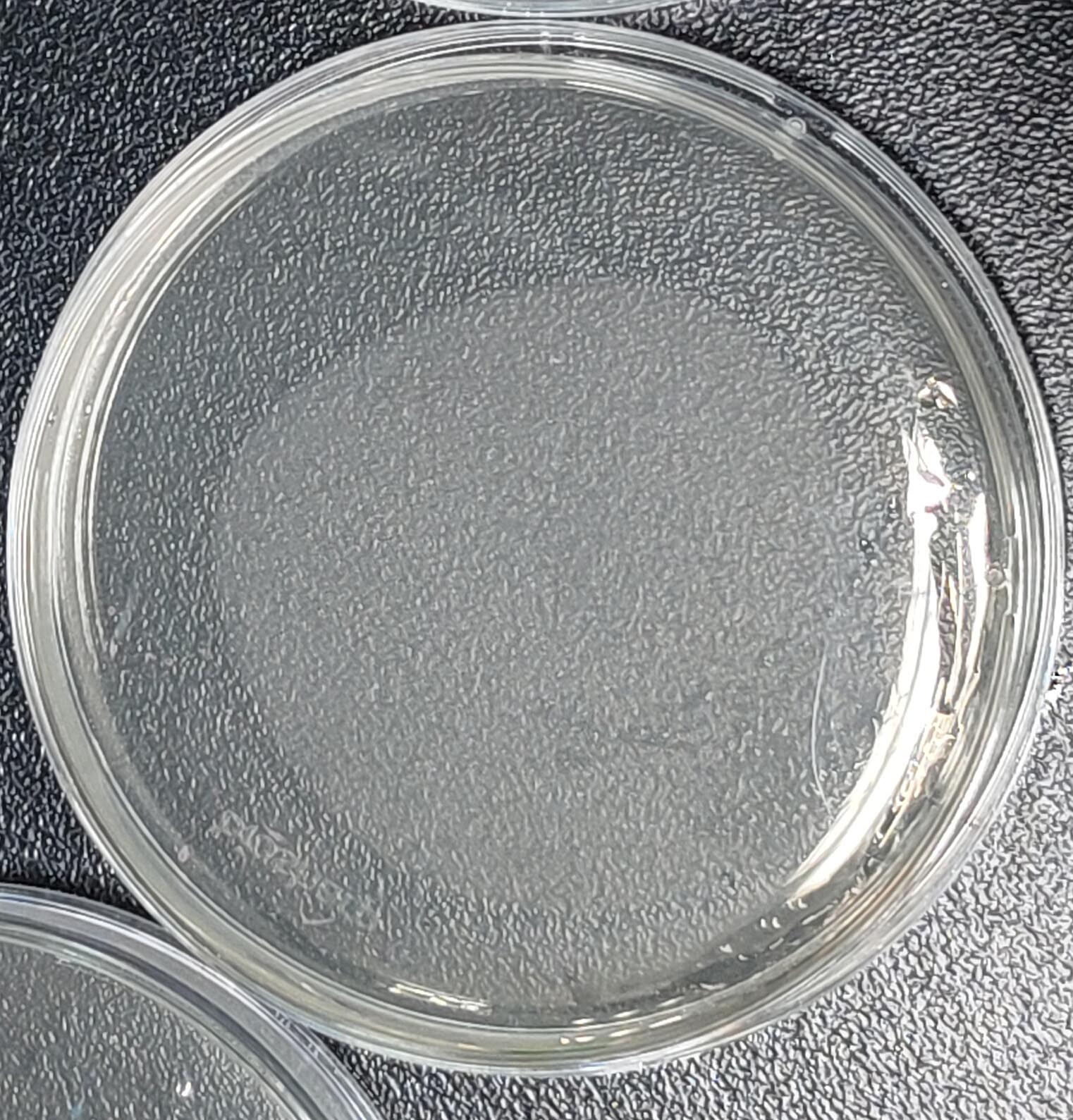

Supplement: Supplementary file 1 [file DataSheet1.zip › Data Sheet 1/Effect of 9 TCMMs on the adhesion ability of XDRAB/adhesion data/4-Terpineol/0 (9).jpg]

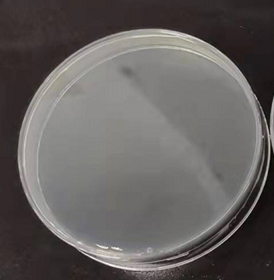

Supplement: Supplementary file 1 [file DataSheet1.zip › Data Sheet 1/Effect of 9 TCMMs on the adhesion ability of XDRAB/adhesion data/4-Terpineol/0.jpg]

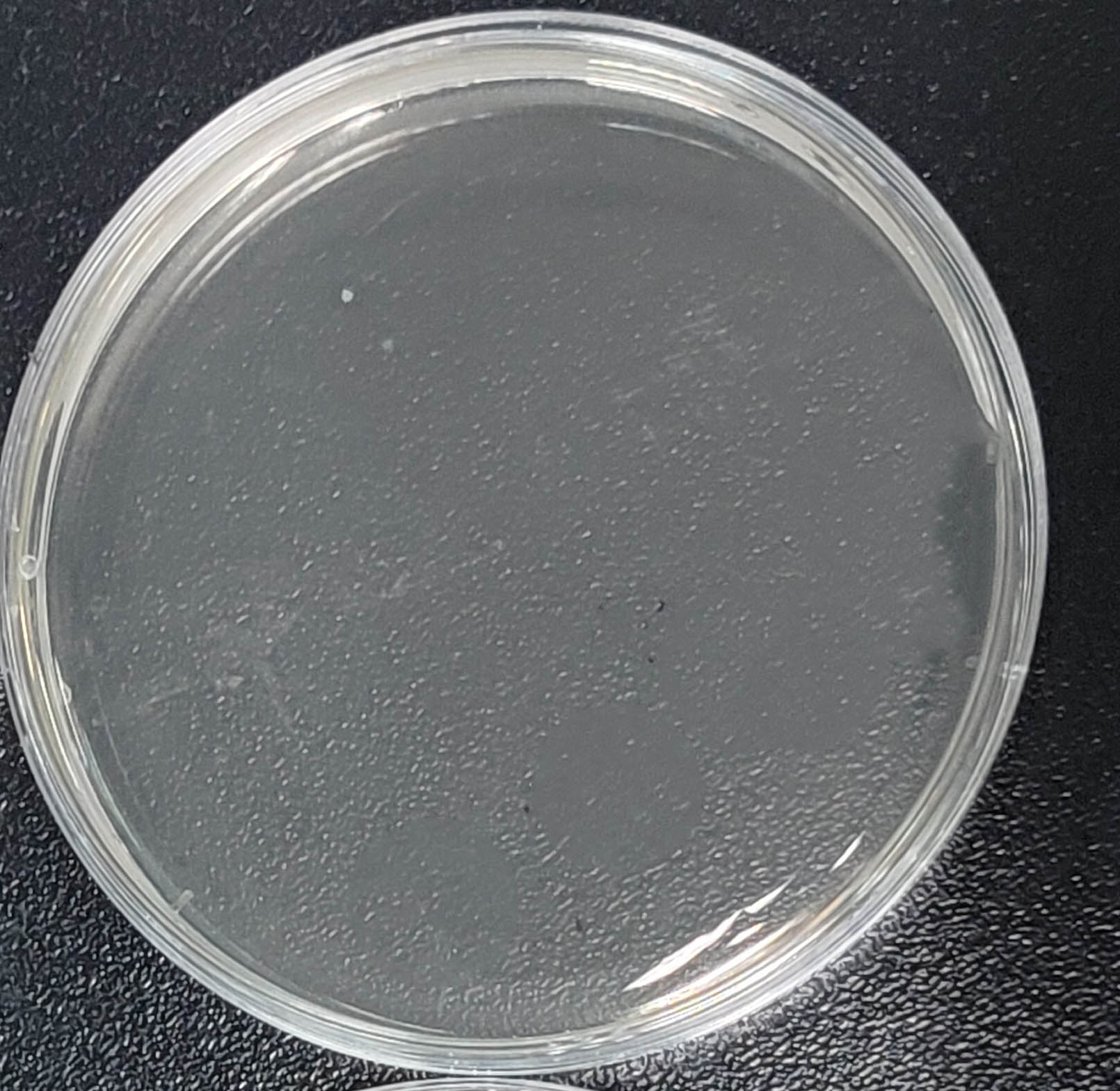

Supplement: Supplementary file 1 [file DataSheet1.zip › Data Sheet 1/Effect of 9 TCMMs on the adhesion ability of XDRAB/adhesion data/4-Terpineol/1 (2).jpg]

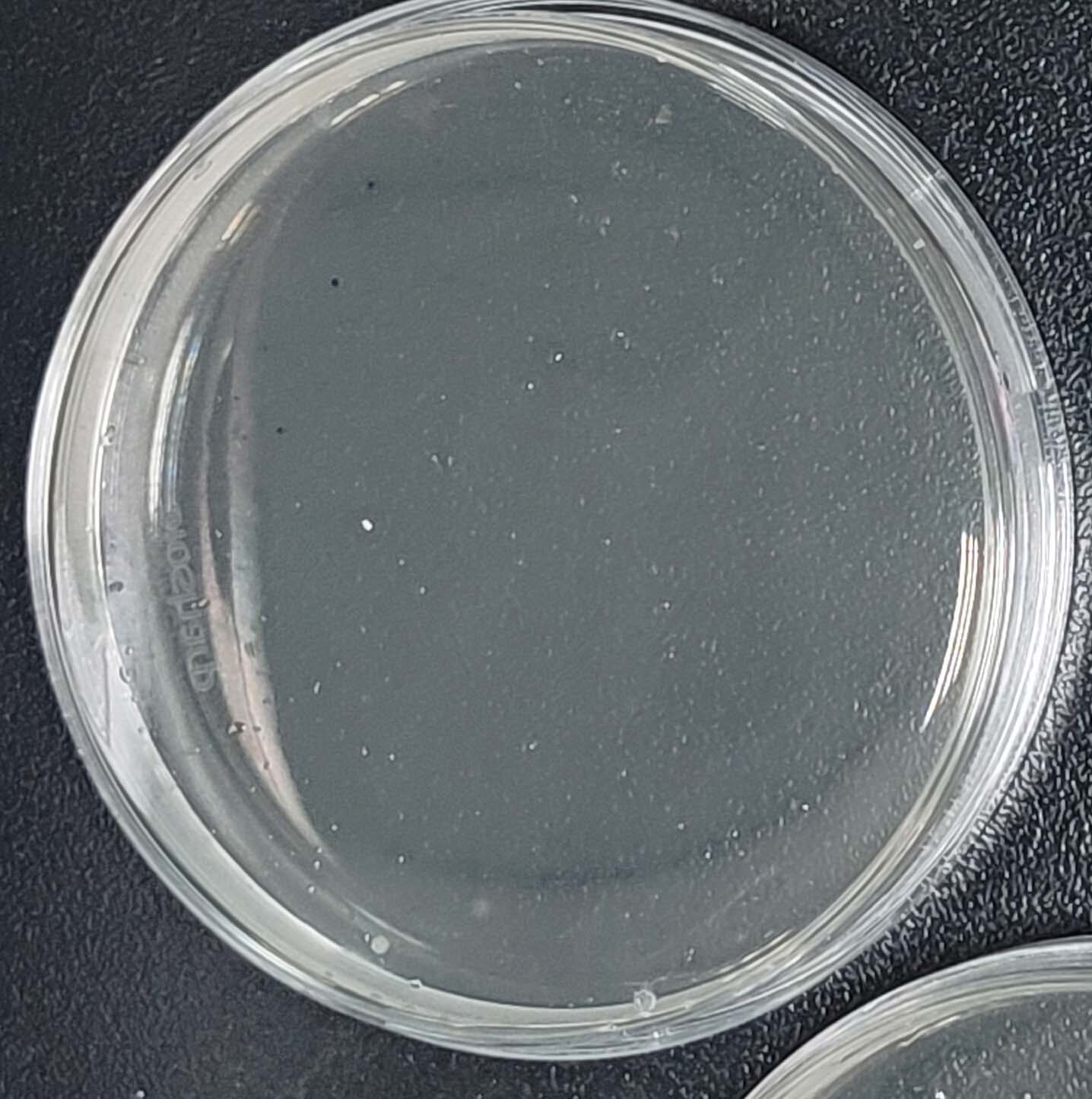

Supplement: Supplementary file 1 [file DataSheet1.zip › Data Sheet 1/Effect of 9 TCMMs on the adhesion ability of XDRAB/adhesion data/4-Terpineol/1.jpg]

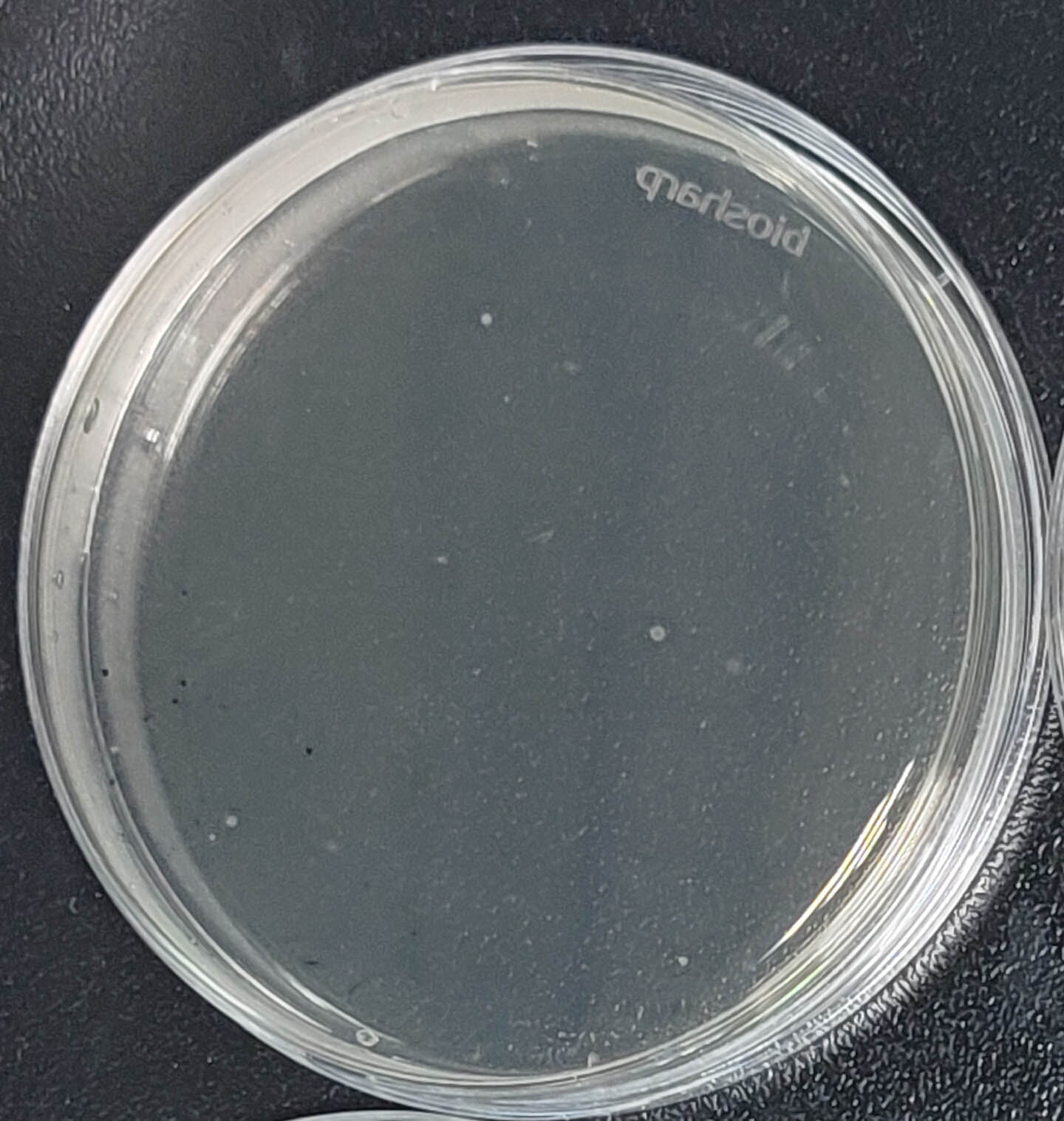

Supplement: Supplementary file 1 [file DataSheet1.zip › Data Sheet 1/Effect of 9 TCMMs on the adhesion ability of XDRAB/adhesion data/4-Terpineol/4.jpg]

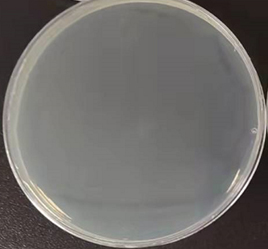

Supplement: Supplementary file 1 [file DataSheet1.zip › Data Sheet 1/Effect of 9 TCMMs on the adhesion ability of XDRAB/adhesion data/Caffeic acid/0 (2).jpg]

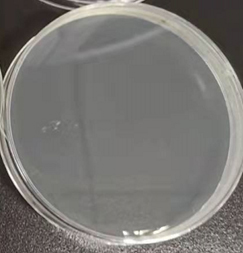

Supplement: Supplementary file 1 [file DataSheet1.zip › Data Sheet 1/Effect of 9 TCMMs on the adhesion ability of XDRAB/adhesion data/Caffeic acid/0 (3).jpg]

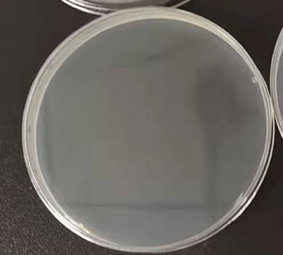

Supplement: Supplementary file 1 [file DataSheet1.zip › Data Sheet 1/Effect of 9 TCMMs on the adhesion ability of XDRAB/adhesion data/Caffeic acid/0 (4).jpg]

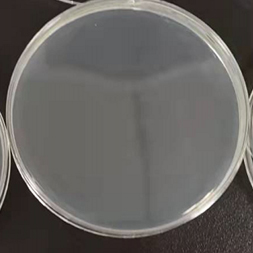

Supplement: Supplementary file 1 [file DataSheet1.zip › Data Sheet 1/Effect of 9 TCMMs on the adhesion ability of XDRAB/adhesion data/Caffeic acid/0 (5).jpg]

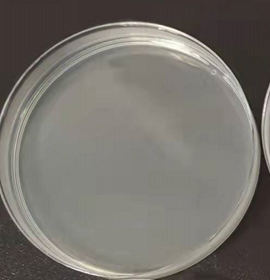

Supplement: Supplementary file 1 [file DataSheet1.zip › Data Sheet 1/Effect of 9 TCMMs on the adhesion ability of XDRAB/adhesion data/Caffeic acid/0 (6).jpg]

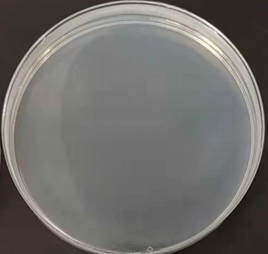

Supplement: Supplementary file 1 [file DataSheet1.zip › Data Sheet 1/Effect of 9 TCMMs on the adhesion ability of XDRAB/adhesion data/Caffeic acid/0 (7).jpg]

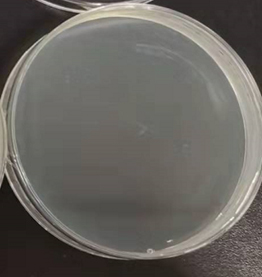

Supplement: Supplementary file 1 [file DataSheet1.zip › Data Sheet 1/Effect of 9 TCMMs on the adhesion ability of XDRAB/adhesion data/Caffeic acid/0 (8).jpg]

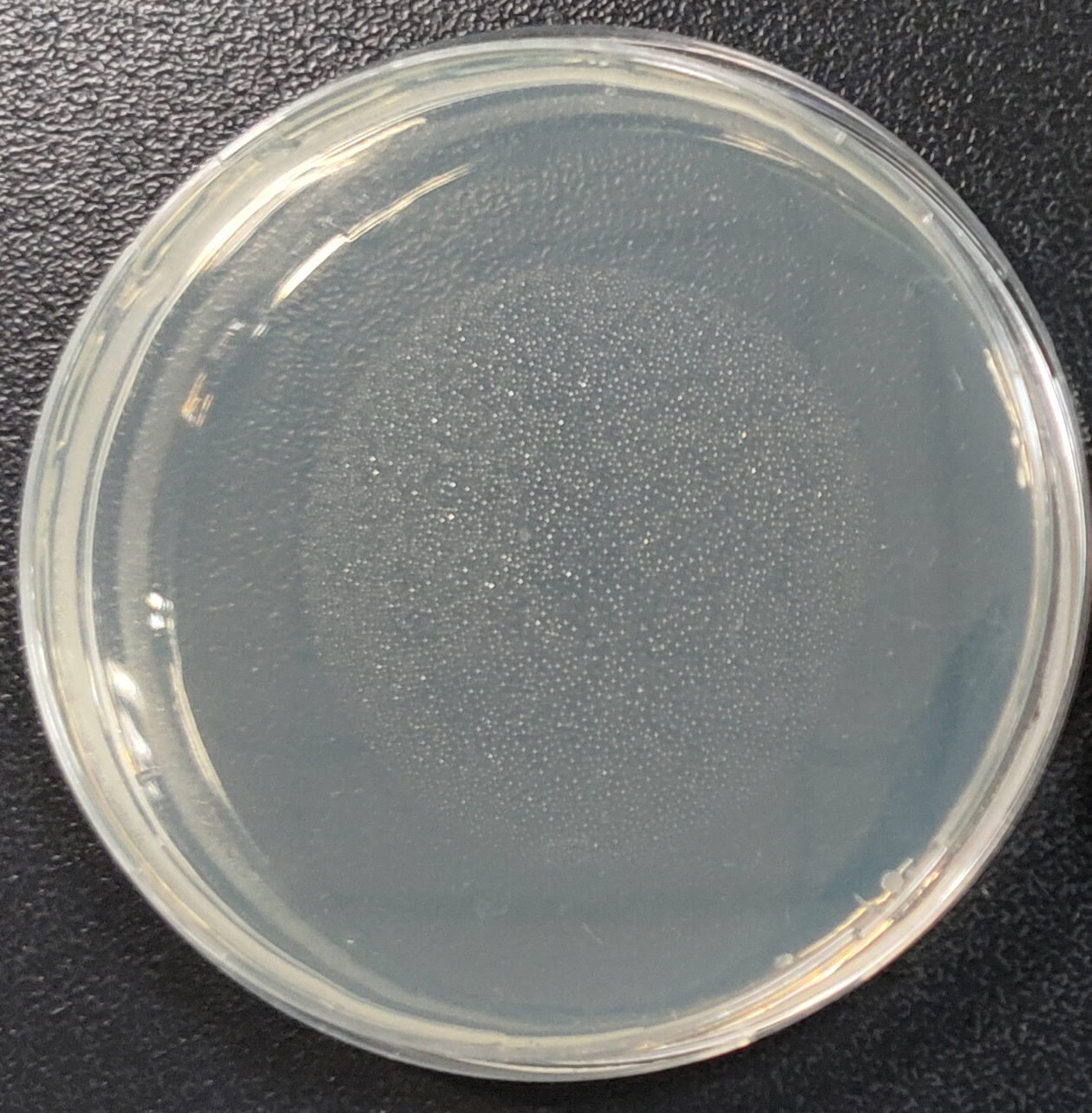

Supplement: Supplementary file 1 [file DataSheet1.zip › Data Sheet 1/Effect of 9 TCMMs on the adhesion ability of XDRAB/adhesion data/Caffeic acid/0 (9).jpg]

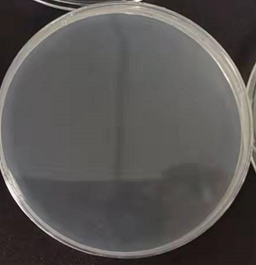

Supplement: Supplementary file 1 [file DataSheet1.zip › Data Sheet 1/Effect of 9 TCMMs on the adhesion ability of XDRAB/adhesion data/Caffeic acid/0.jpg]

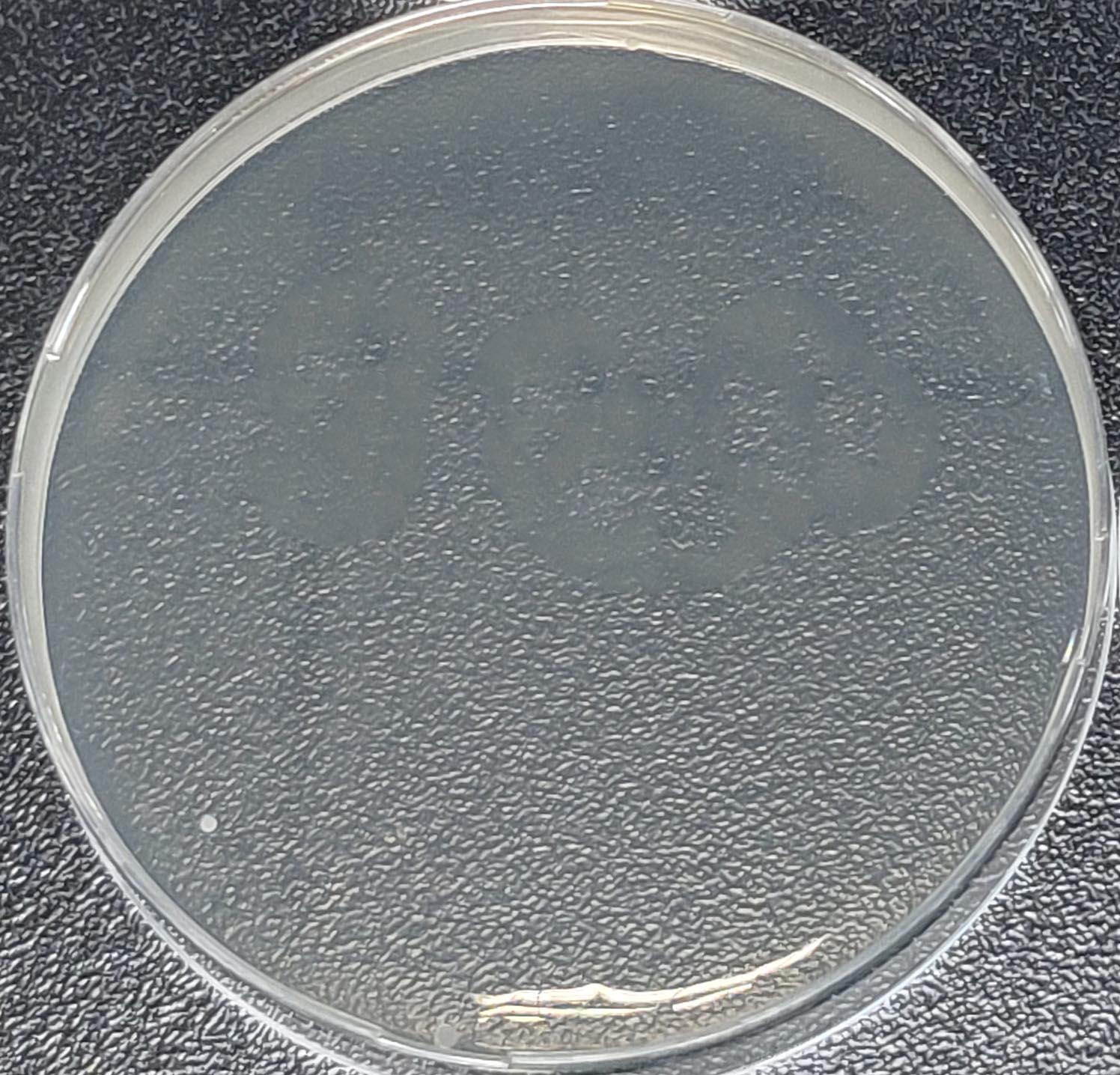

Supplement: Supplementary file 1 [file DataSheet1.zip › Data Sheet 1/Effect of 9 TCMMs on the adhesion ability of XDRAB/adhesion data/Caffeic acid/1.jpg]

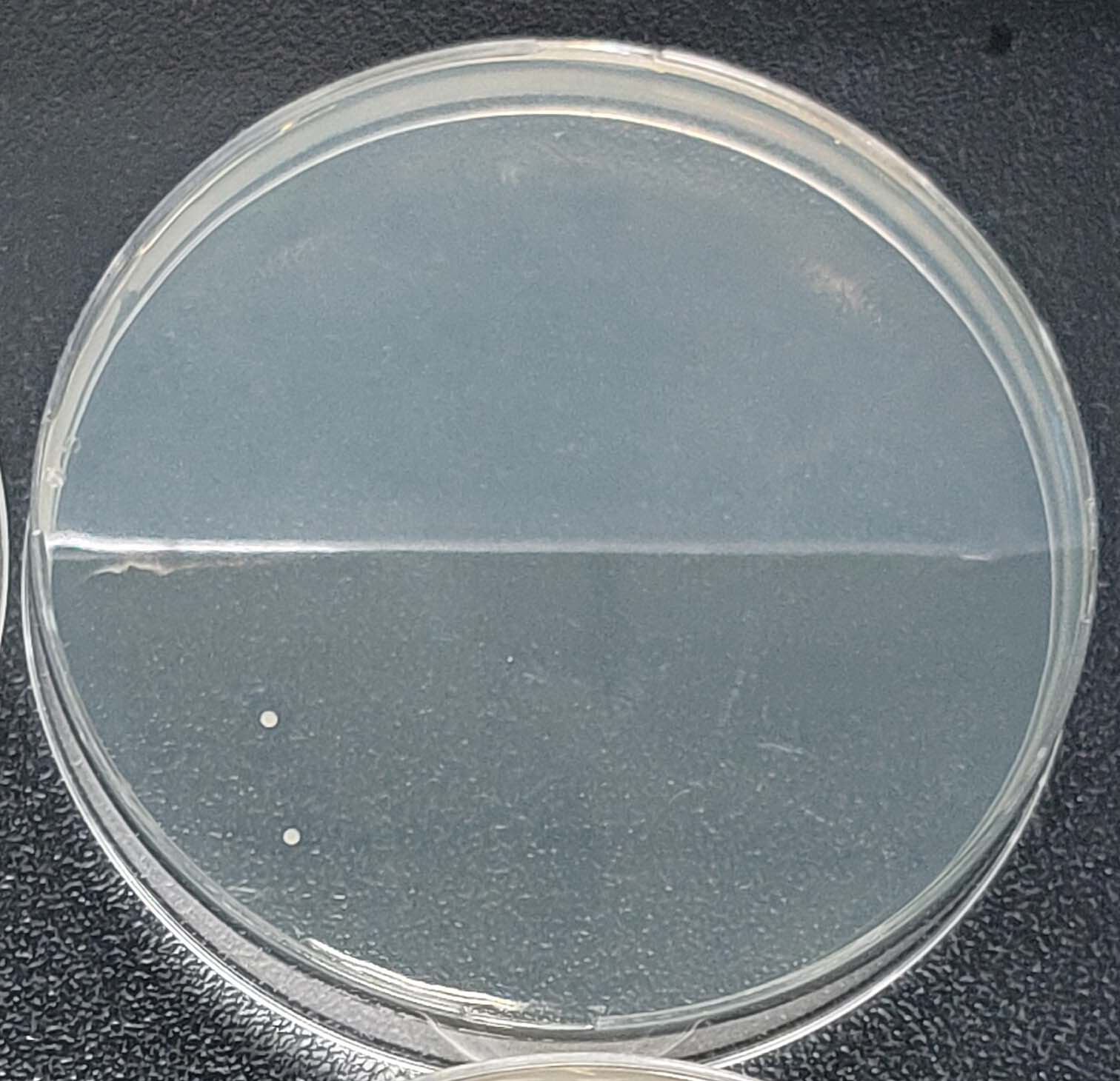

Supplement: Supplementary file 1 [file DataSheet1.zip › Data Sheet 1/Effect of 9 TCMMs on the adhesion ability of XDRAB/adhesion data/Caffeic acid/2.jpg]

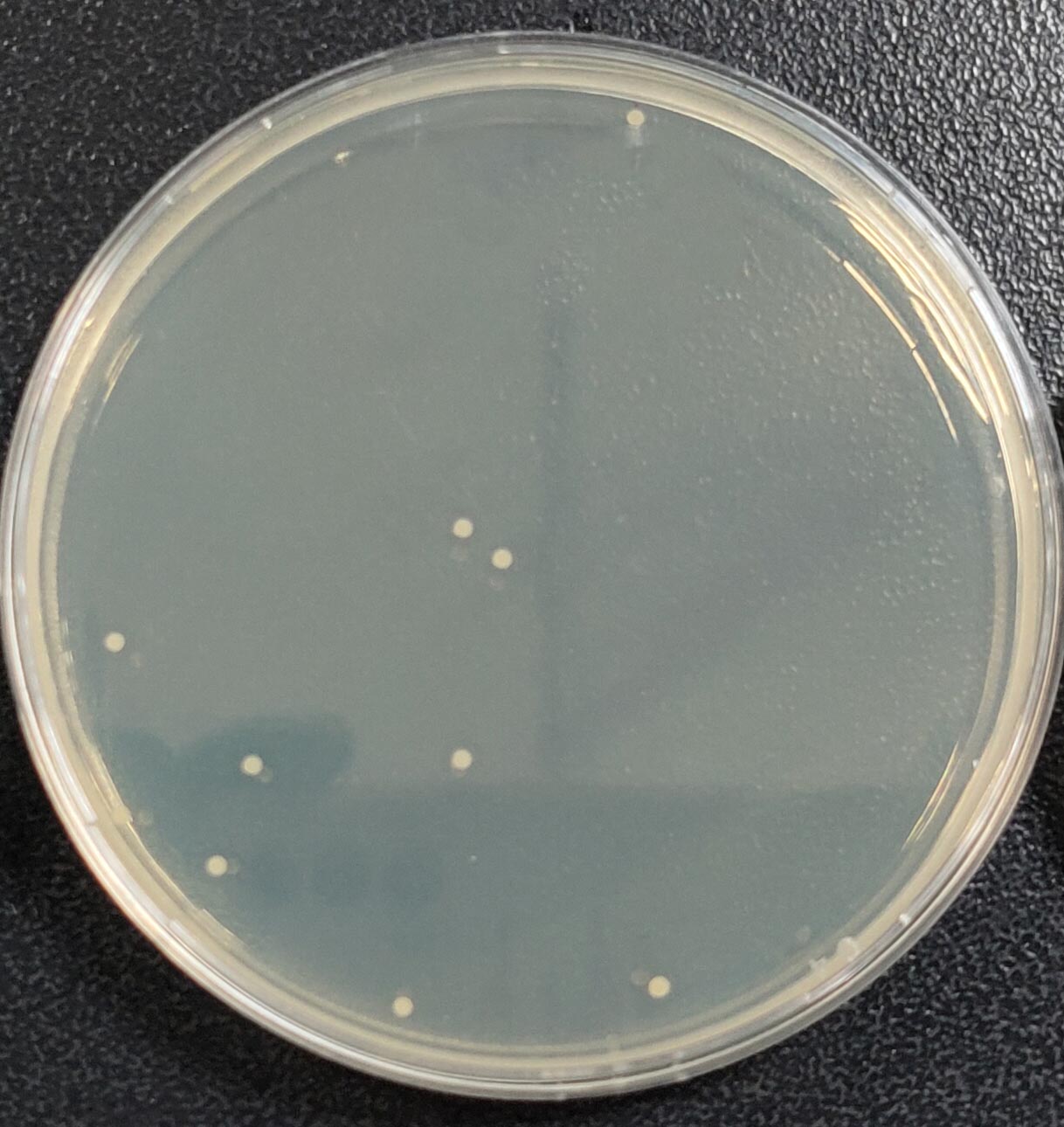

Supplement: Supplementary file 1 [file DataSheet1.zip › Data Sheet 1/Effect of 9 TCMMs on the adhesion ability of XDRAB/adhesion data/Caffeic acid/9.jpg]

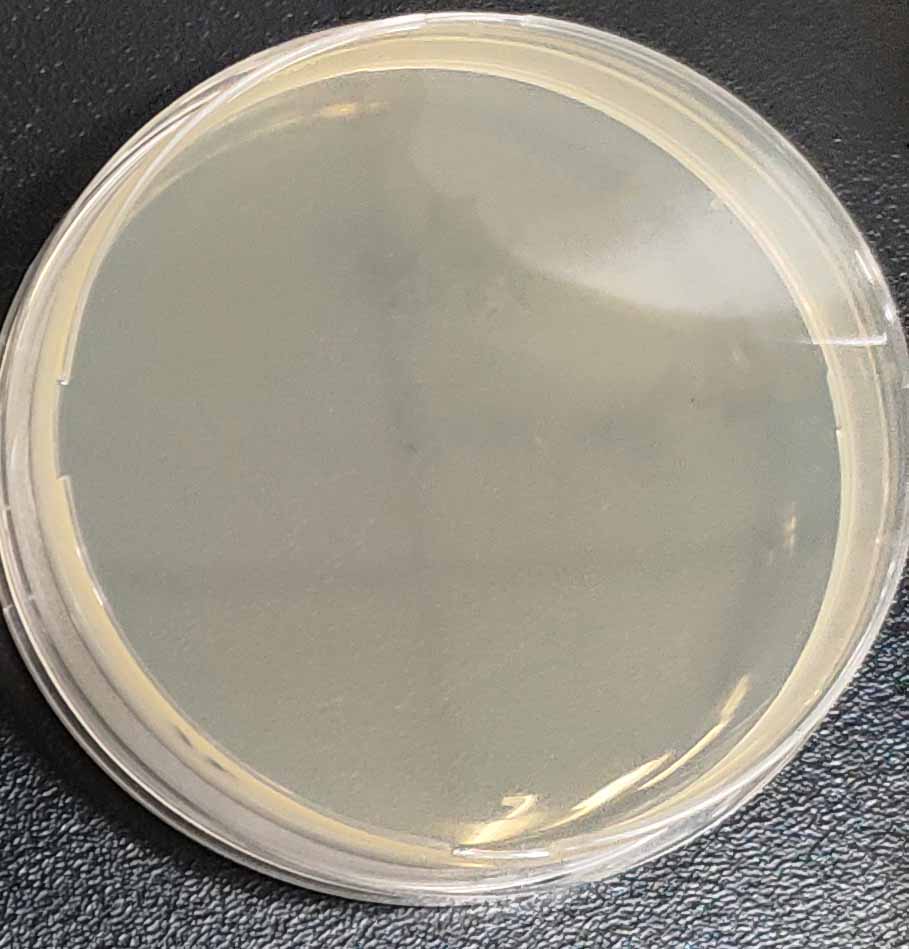

Supplement: Supplementary file 1 [file DataSheet1.zip › Data Sheet 1/Effect of 9 TCMMs on the adhesion ability of XDRAB/adhesion data/Cinnamic acid/0 (10).jpg]

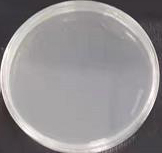

Supplement: Supplementary file 1 [file DataSheet1.zip › Data Sheet 1/Effect of 9 TCMMs on the adhesion ability of XDRAB/adhesion data/Cinnamic acid/0 (2).jpg]

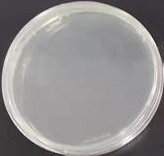

Supplement: Supplementary file 1 [file DataSheet1.zip › Data Sheet 1/Effect of 9 TCMMs on the adhesion ability of XDRAB/adhesion data/Cinnamic acid/0 (3).jpg]

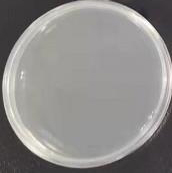

Supplement: Supplementary file 1 [file DataSheet1.zip › Data Sheet 1/Effect of 9 TCMMs on the adhesion ability of XDRAB/adhesion data/Cinnamic acid/0 (4).jpg]

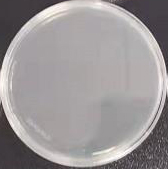

Supplement: Supplementary file 1 [file DataSheet1.zip › Data Sheet 1/Effect of 9 TCMMs on the adhesion ability of XDRAB/adhesion data/Cinnamic acid/0 (5).jpg]

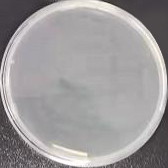

Supplement: Supplementary file 1 [file DataSheet1.zip › Data Sheet 1/Effect of 9 TCMMs on the adhesion ability of XDRAB/adhesion data/Cinnamic acid/0 (6).jpg]

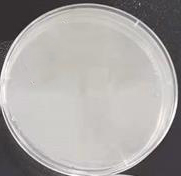

Supplement: Supplementary file 1 [file DataSheet1.zip › Data Sheet 1/Effect of 9 TCMMs on the adhesion ability of XDRAB/adhesion data/Cinnamic acid/0 (7).jpg]

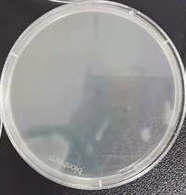

Supplement: Supplementary file 1 [file DataSheet1.zip › Data Sheet 1/Effect of 9 TCMMs on the adhesion ability of XDRAB/adhesion data/Cinnamic acid/0 (8).jpg]

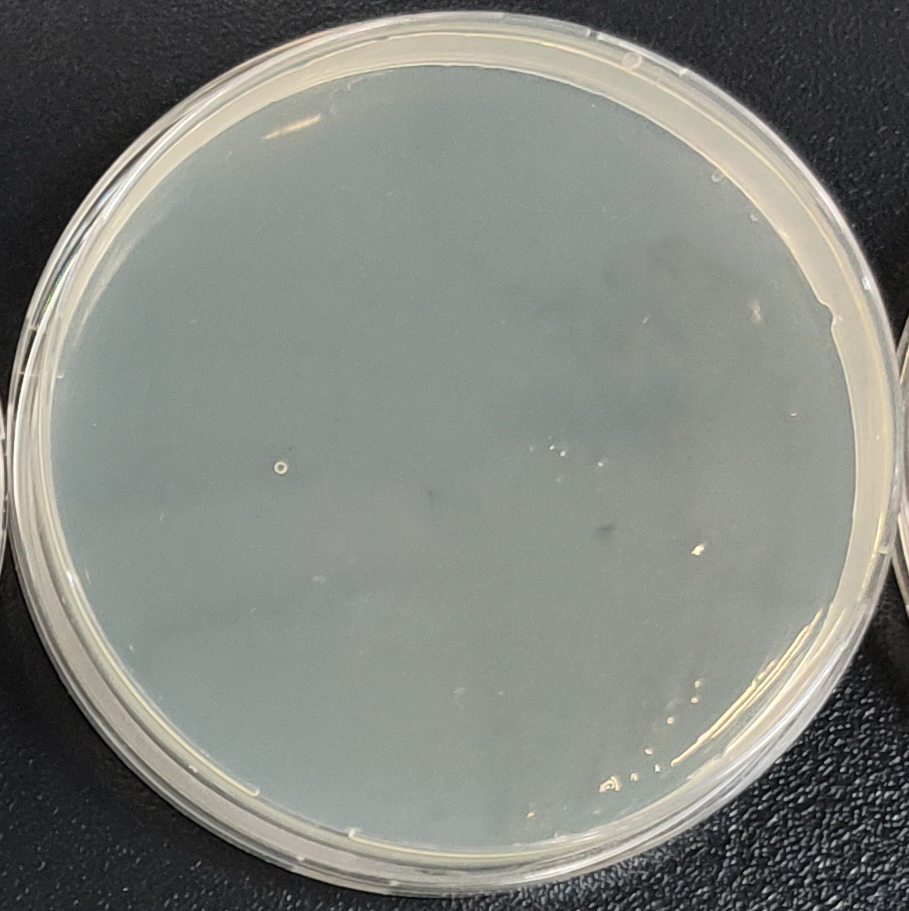

Supplement: Supplementary file 1 [file DataSheet1.zip › Data Sheet 1/Effect of 9 TCMMs on the adhesion ability of XDRAB/adhesion data/Cinnamic acid/0 (9).jpg]

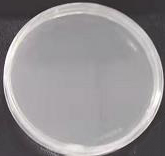

Supplement: Supplementary file 1 [file DataSheet1.zip › Data Sheet 1/Effect of 9 TCMMs on the adhesion ability of XDRAB/adhesion data/Cinnamic acid/0.jpg]

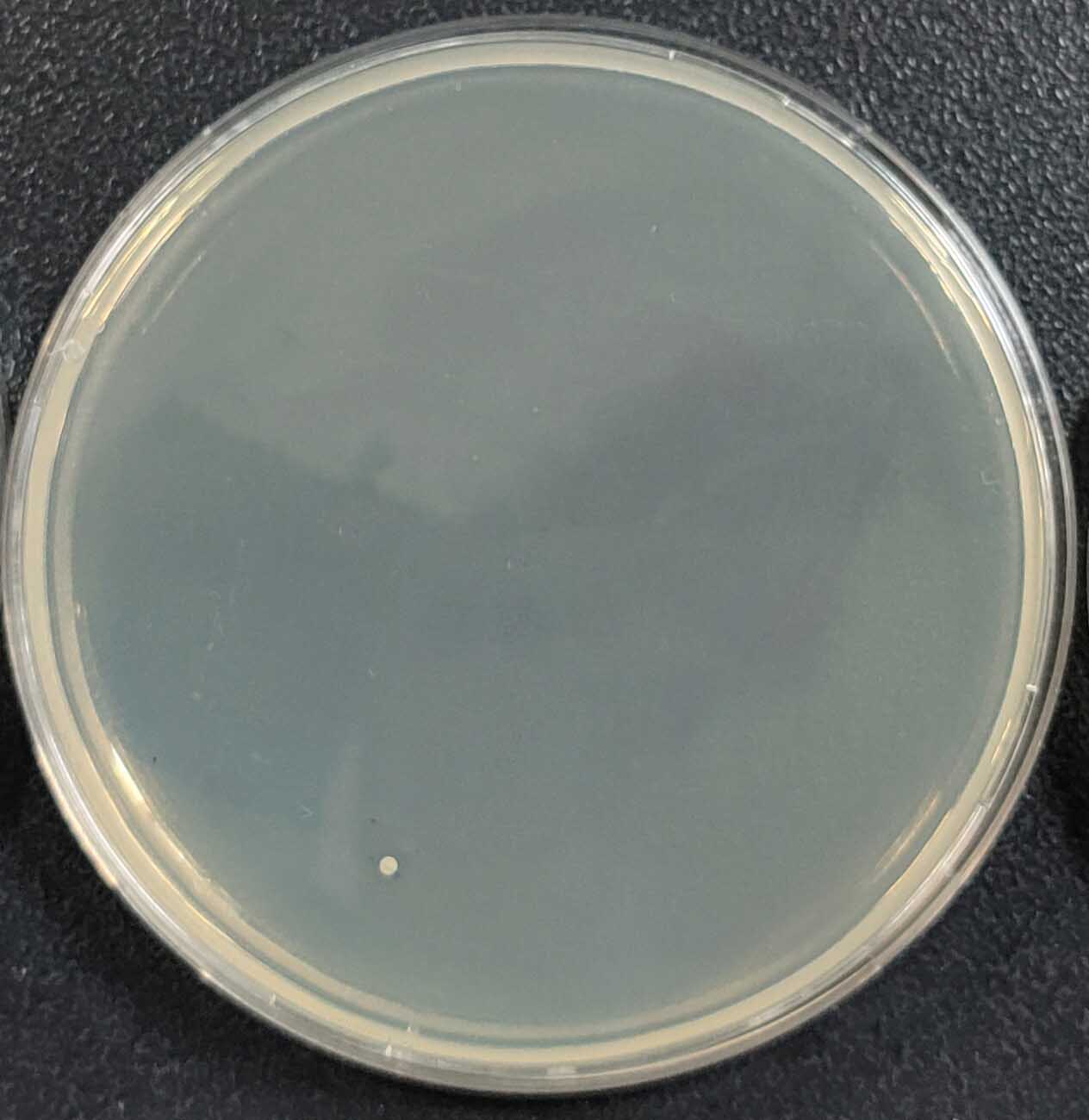

Supplement: Supplementary file 1 [file DataSheet1.zip › Data Sheet 1/Effect of 9 TCMMs on the adhesion ability of XDRAB/adhesion data/Cinnamic acid/1 (2).jpg]

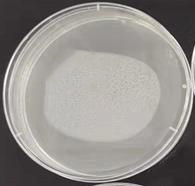

Supplement: Supplementary file 1 [file DataSheet1.zip › Data Sheet 1/Effect of 9 TCMMs on the adhesion ability of XDRAB/adhesion data/Cinnamic acid/1.jpg]

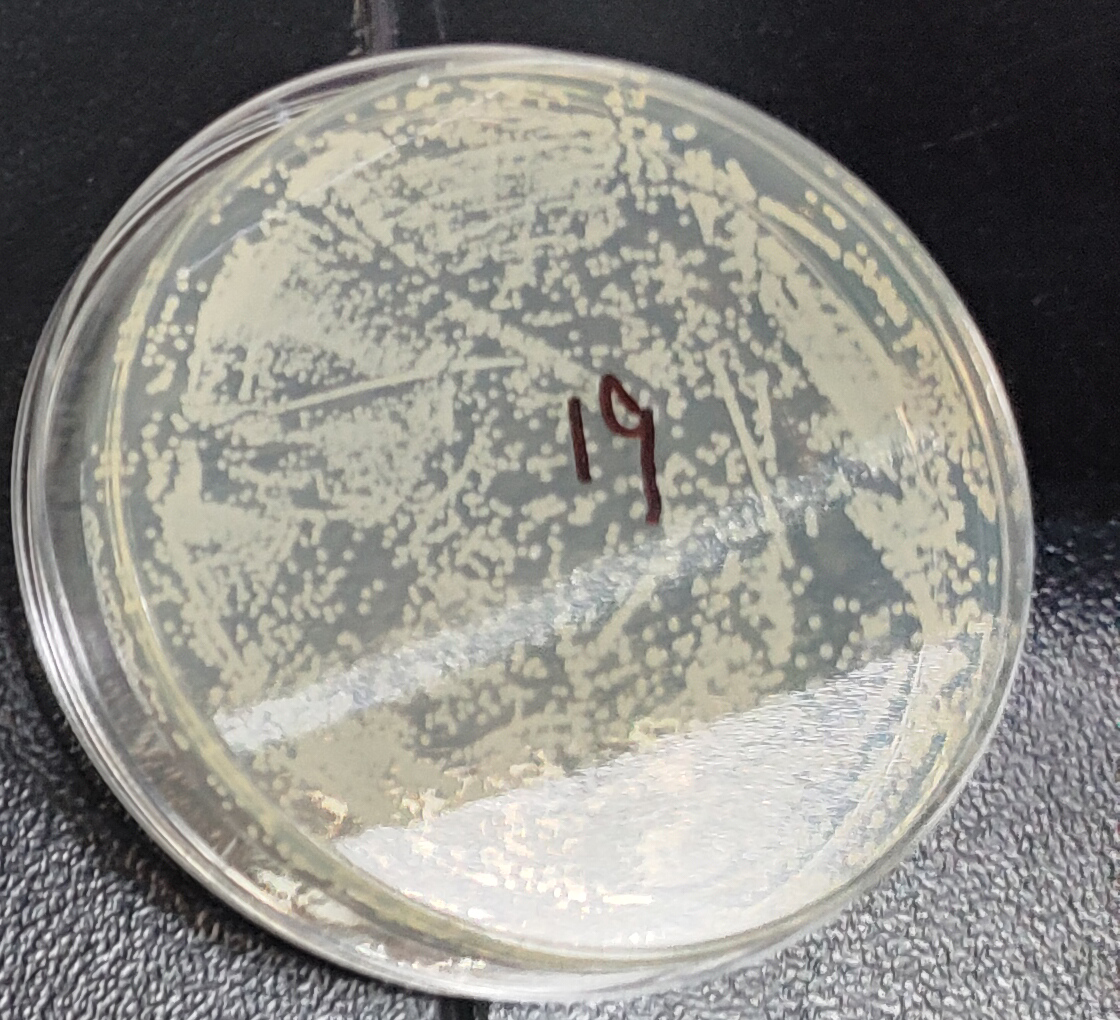

Supplement: Supplementary file 1 [file DataSheet1.zip › Data Sheet 1/Effect of 9 TCMMs on the adhesion ability of XDRAB/adhesion data/control/P11004-154412.jpg]

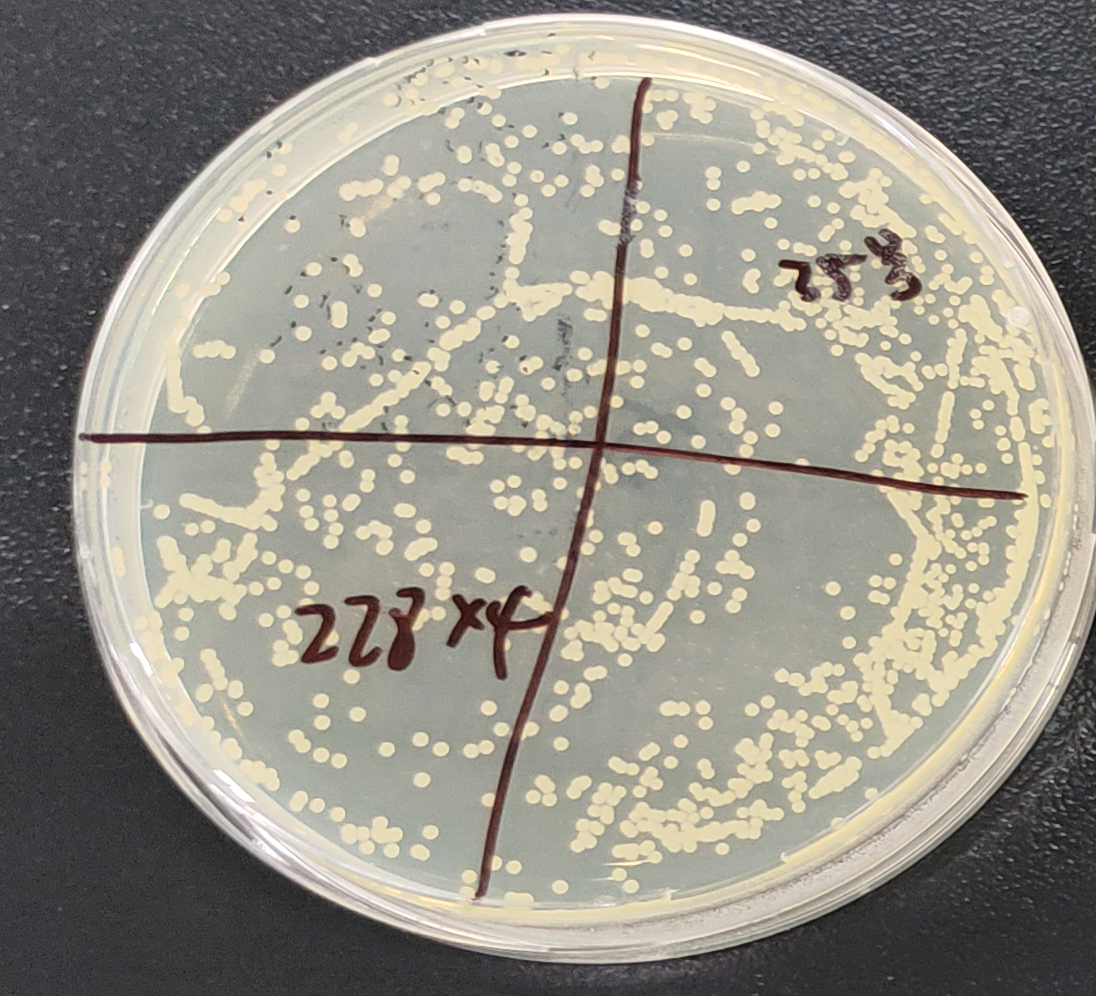

Supplement: Supplementary file 1 [file DataSheet1.zip › Data Sheet 1/Effect of 9 TCMMs on the adhesion ability of XDRAB/adhesion data/control/P11026-144341.jpg]

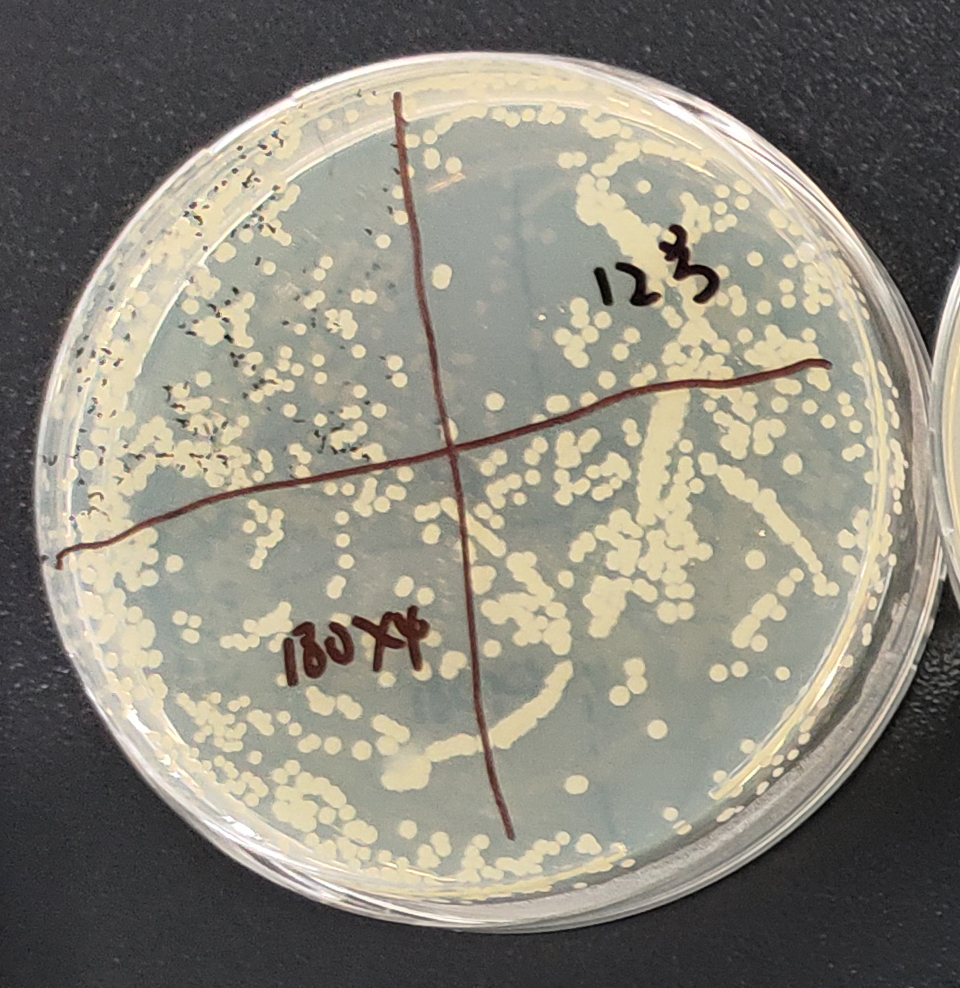

Supplement: Supplementary file 1 [file DataSheet1.zip › Data Sheet 1/Effect of 9 TCMMs on the adhesion ability of XDRAB/adhesion data/control/P11026-144454.jpg]

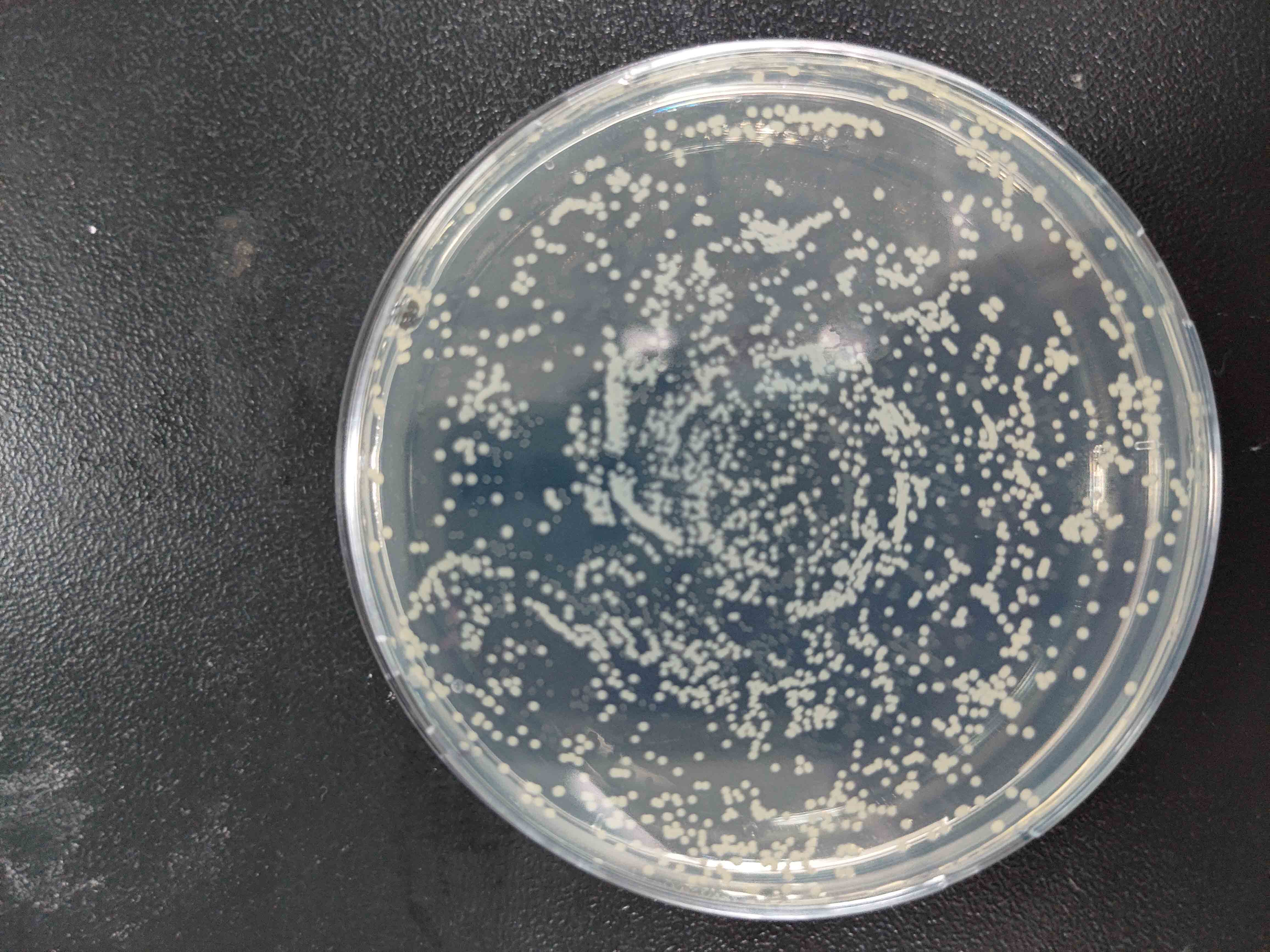

Supplement: Supplementary file 1 [file DataSheet1.zip › Data Sheet 1/Effect of 9 TCMMs on the adhesion ability of XDRAB/adhesion data/control/P11031-204145.jpg]

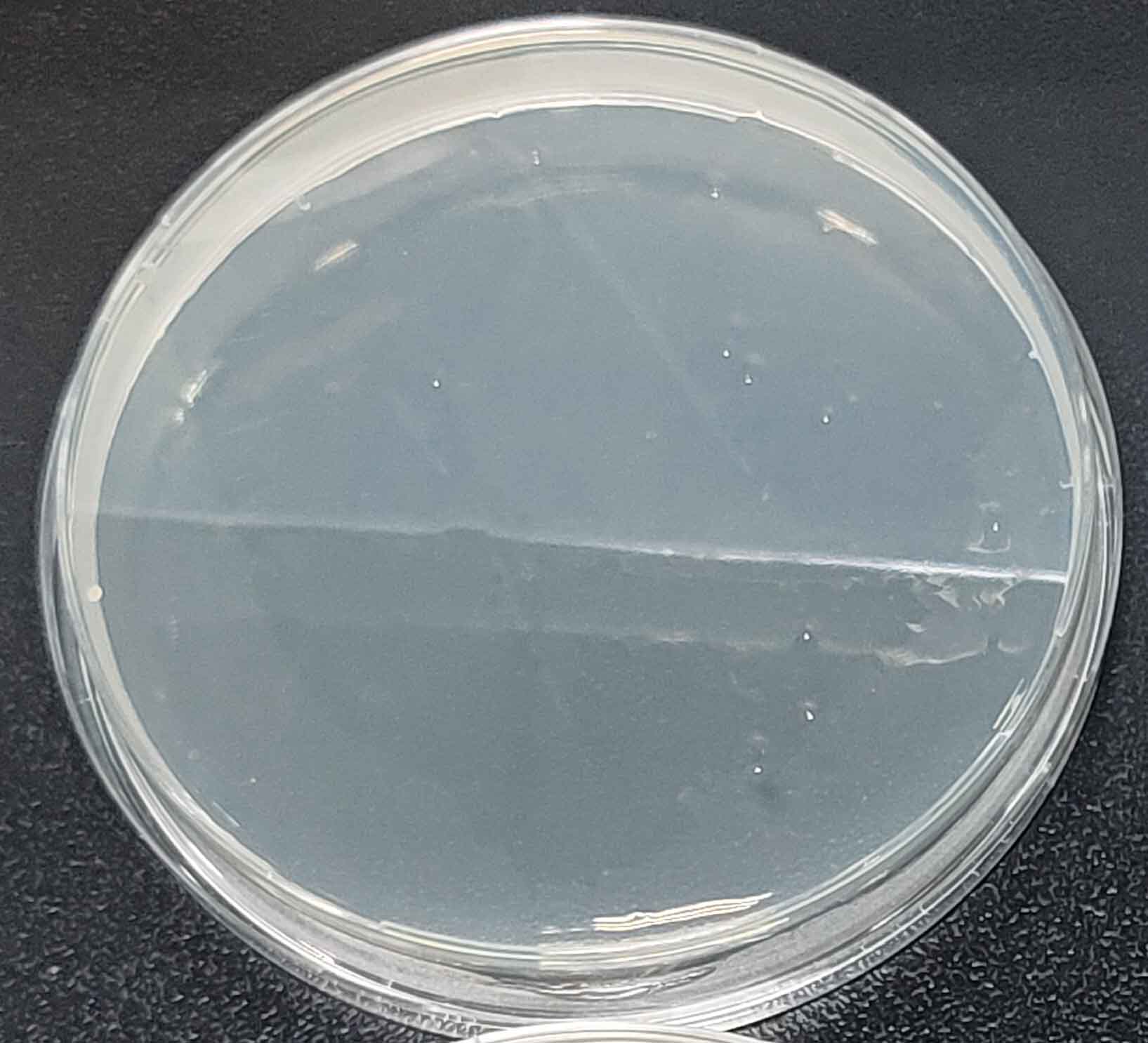

Supplement: Supplementary file 1 [file DataSheet1.zip › Data Sheet 1/Effect of 9 TCMMs on the adhesion ability of XDRAB/adhesion data/Coumarin/1.jpg]

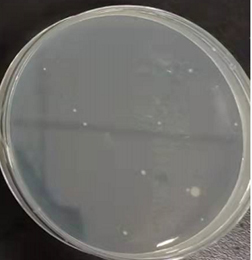

Supplement: Supplementary file 1 [file DataSheet1.zip › Data Sheet 1/Effect of 9 TCMMs on the adhesion ability of XDRAB/adhesion data/Coumarin/10.jpg]

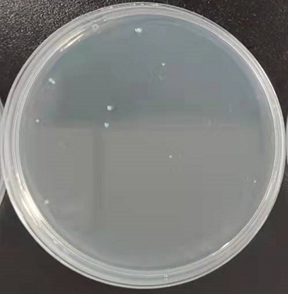

Supplement: Supplementary file 1 [file DataSheet1.zip › Data Sheet 1/Effect of 9 TCMMs on the adhesion ability of XDRAB/adhesion data/Coumarin/11.jpg]

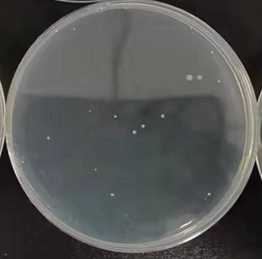

Supplement: Supplementary file 1 [file DataSheet1.zip › Data Sheet 1/Effect of 9 TCMMs on the adhesion ability of XDRAB/adhesion data/Coumarin/16.jpg]

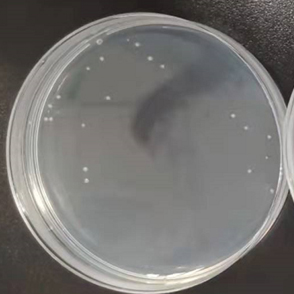

Supplement: Supplementary file 1 [file DataSheet1.zip › Data Sheet 1/Effect of 9 TCMMs on the adhesion ability of XDRAB/adhesion data/Coumarin/20.jpg]

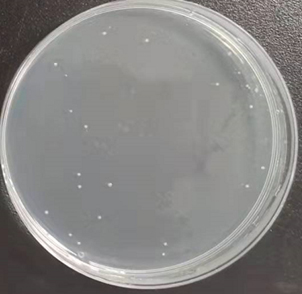

Supplement: Supplementary file 1 [file DataSheet1.zip › Data Sheet 1/Effect of 9 TCMMs on the adhesion ability of XDRAB/adhesion data/Coumarin/24 (2).jpg]

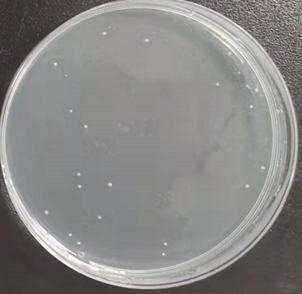

Supplement: Supplementary file 1 [file DataSheet1.zip › Data Sheet 1/Effect of 9 TCMMs on the adhesion ability of XDRAB/adhesion data/Coumarin/24.jpg]

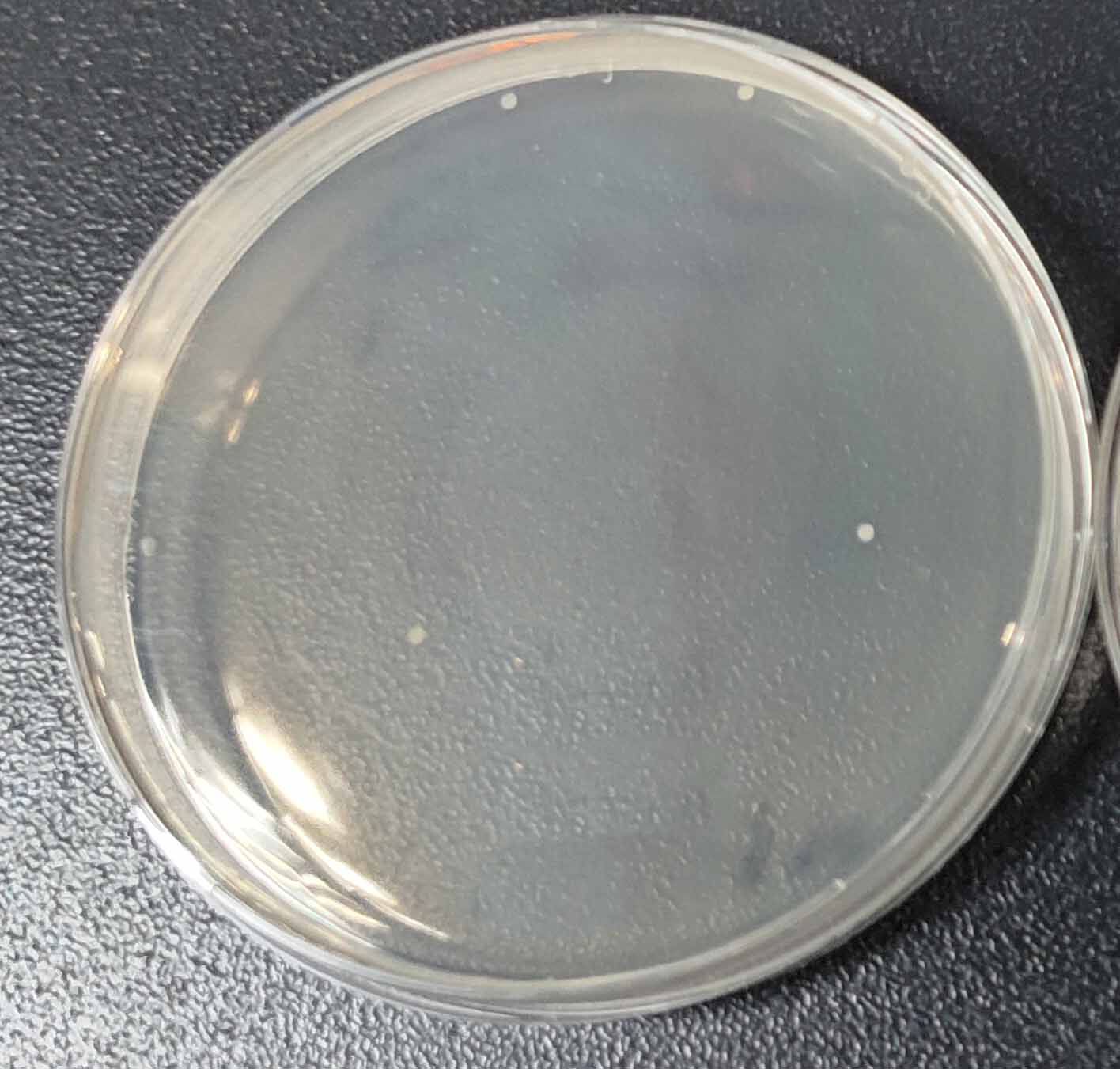

Supplement: Supplementary file 1 [file DataSheet1.zip › Data Sheet 1/Effect of 9 TCMMs on the adhesion ability of XDRAB/adhesion data/Coumarin/4.jpg]

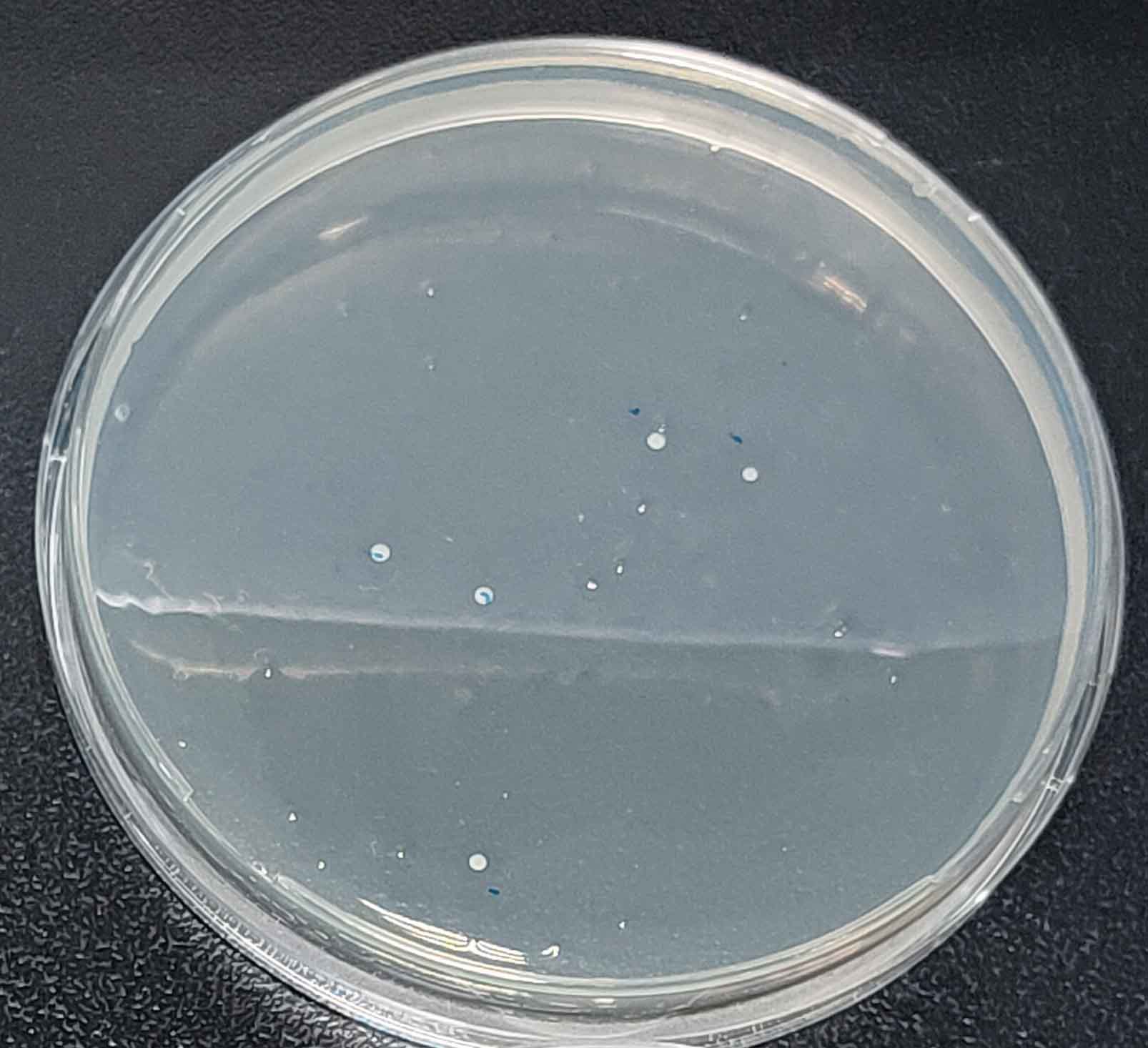

Supplement: Supplementary file 1 [file DataSheet1.zip › Data Sheet 1/Effect of 9 TCMMs on the adhesion ability of XDRAB/adhesion data/Coumarin/5 (2).jpg]

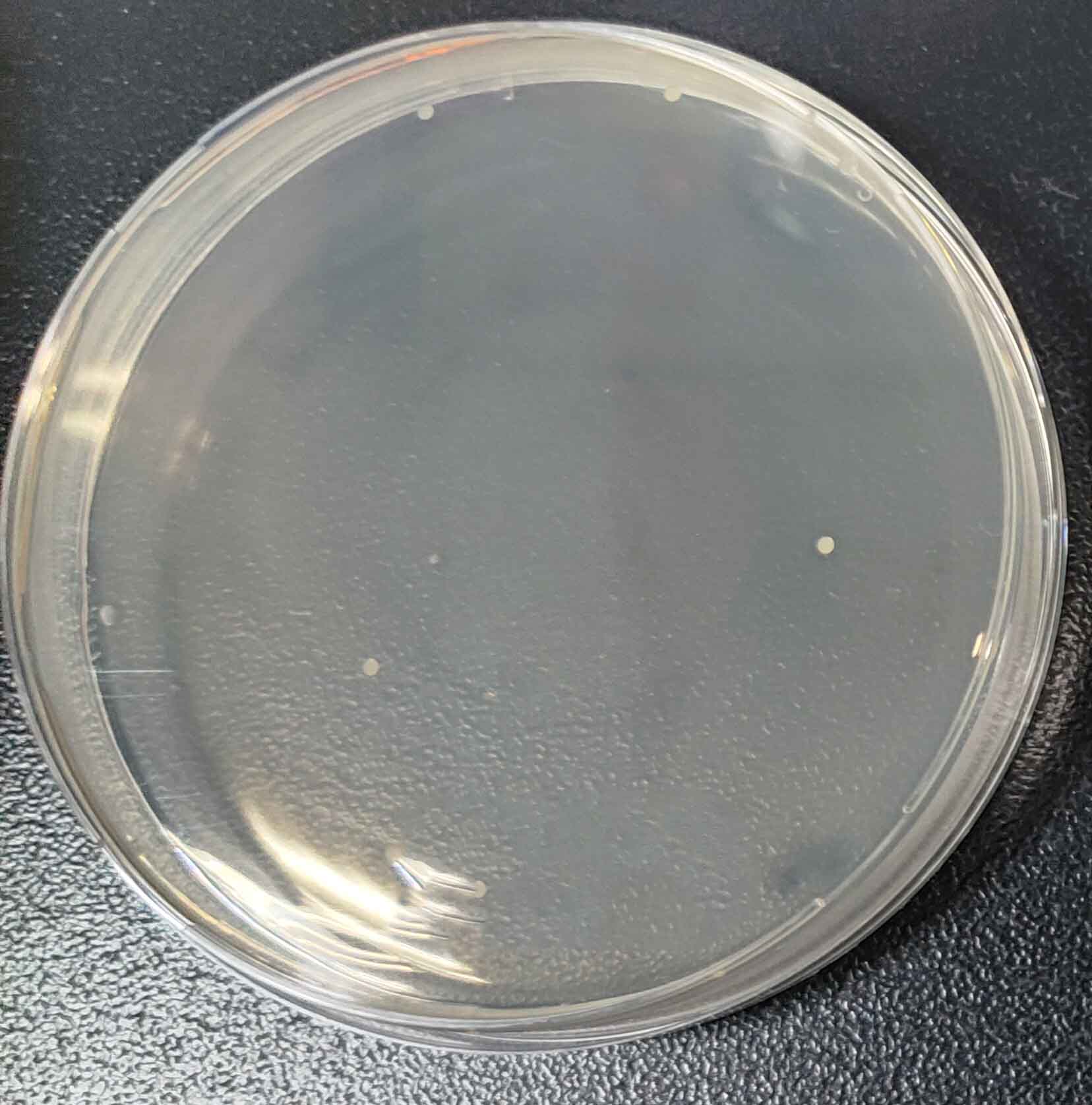

Supplement: Supplementary file 1 [file DataSheet1.zip › Data Sheet 1/Effect of 9 TCMMs on the adhesion ability of XDRAB/adhesion data/Coumarin/5.jpg]

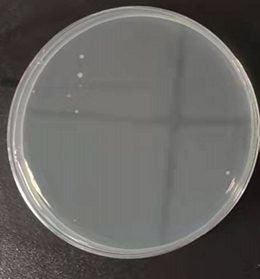

Supplement: Supplementary file 1 [file DataSheet1.zip › Data Sheet 1/Effect of 9 TCMMs on the adhesion ability of XDRAB/adhesion data/Coumarin/6.jpg]

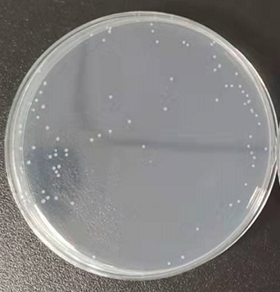

Supplement: Supplementary file 1 [file DataSheet1.zip › Data Sheet 1/Effect of 9 TCMMs on the adhesion ability of XDRAB/adhesion data/Coumarin/76.jpg]

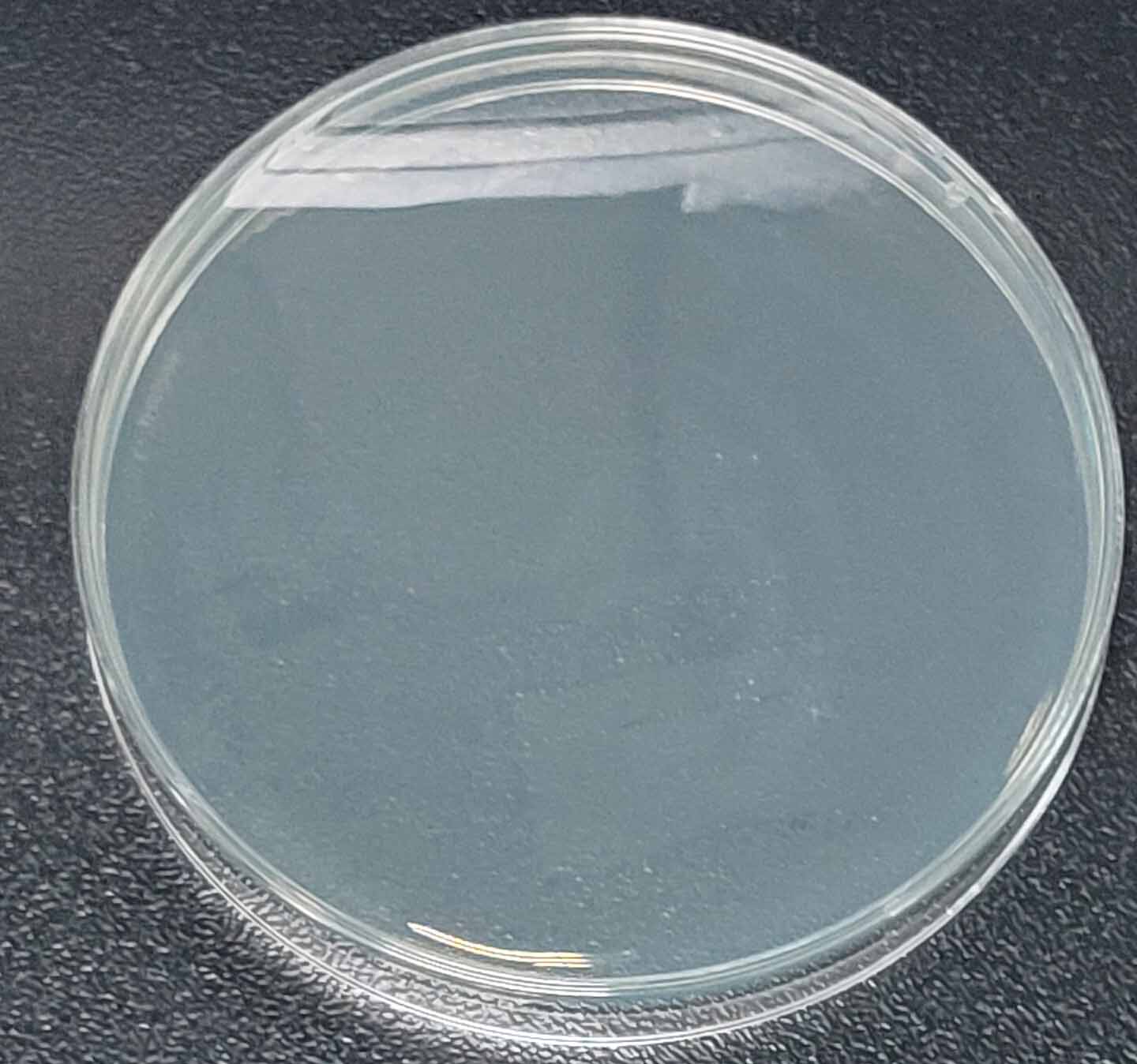

Supplement: Supplementary file 1 [file DataSheet1.zip › Data Sheet 1/Effect of 9 TCMMs on the adhesion ability of XDRAB/adhesion data/Furanone C30/0 (10).jpg]

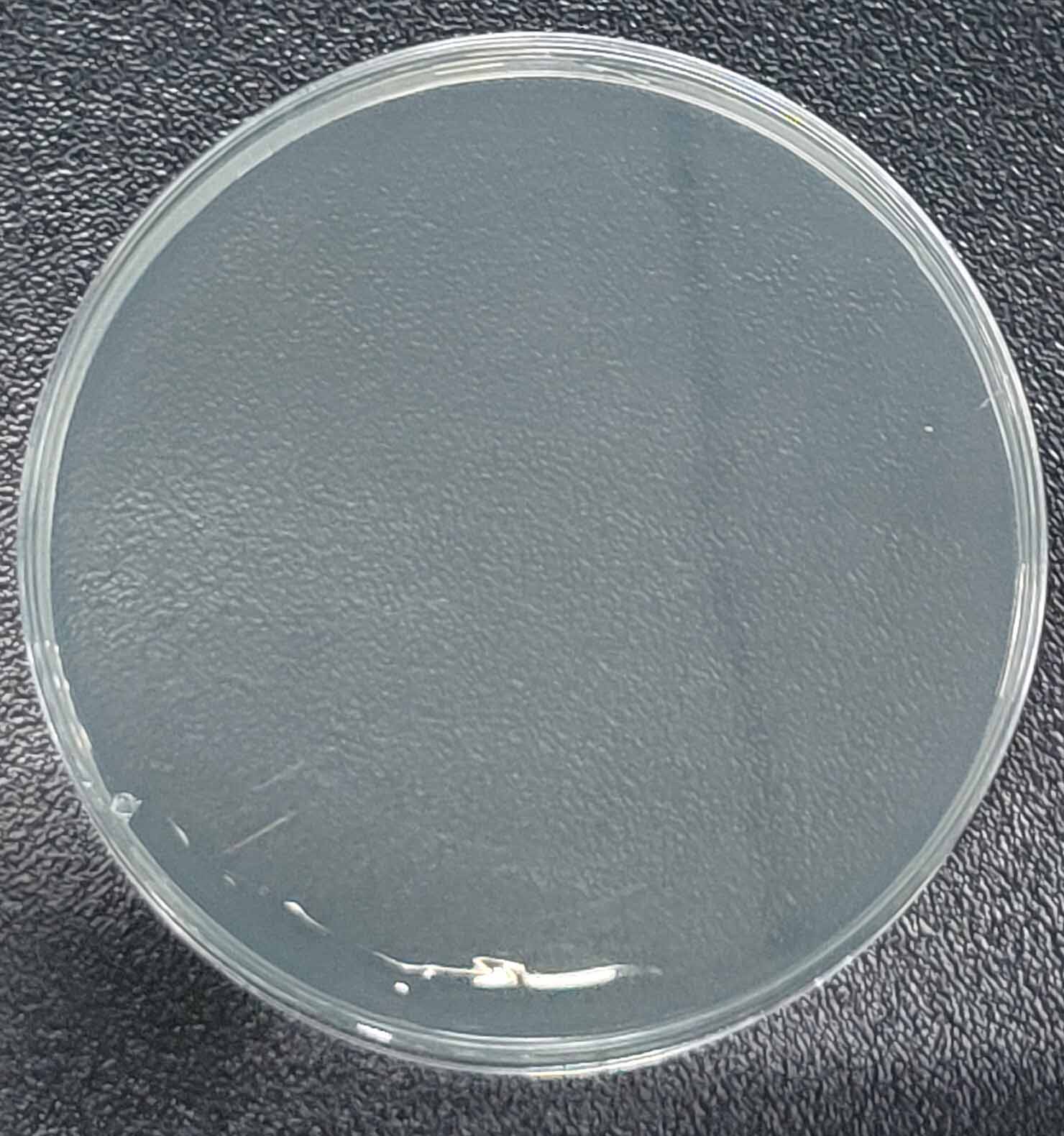

Supplement: Supplementary file 1 [file DataSheet1.zip › Data Sheet 1/Effect of 9 TCMMs on the adhesion ability of XDRAB/adhesion data/Furanone C30/0 (2).jpg]

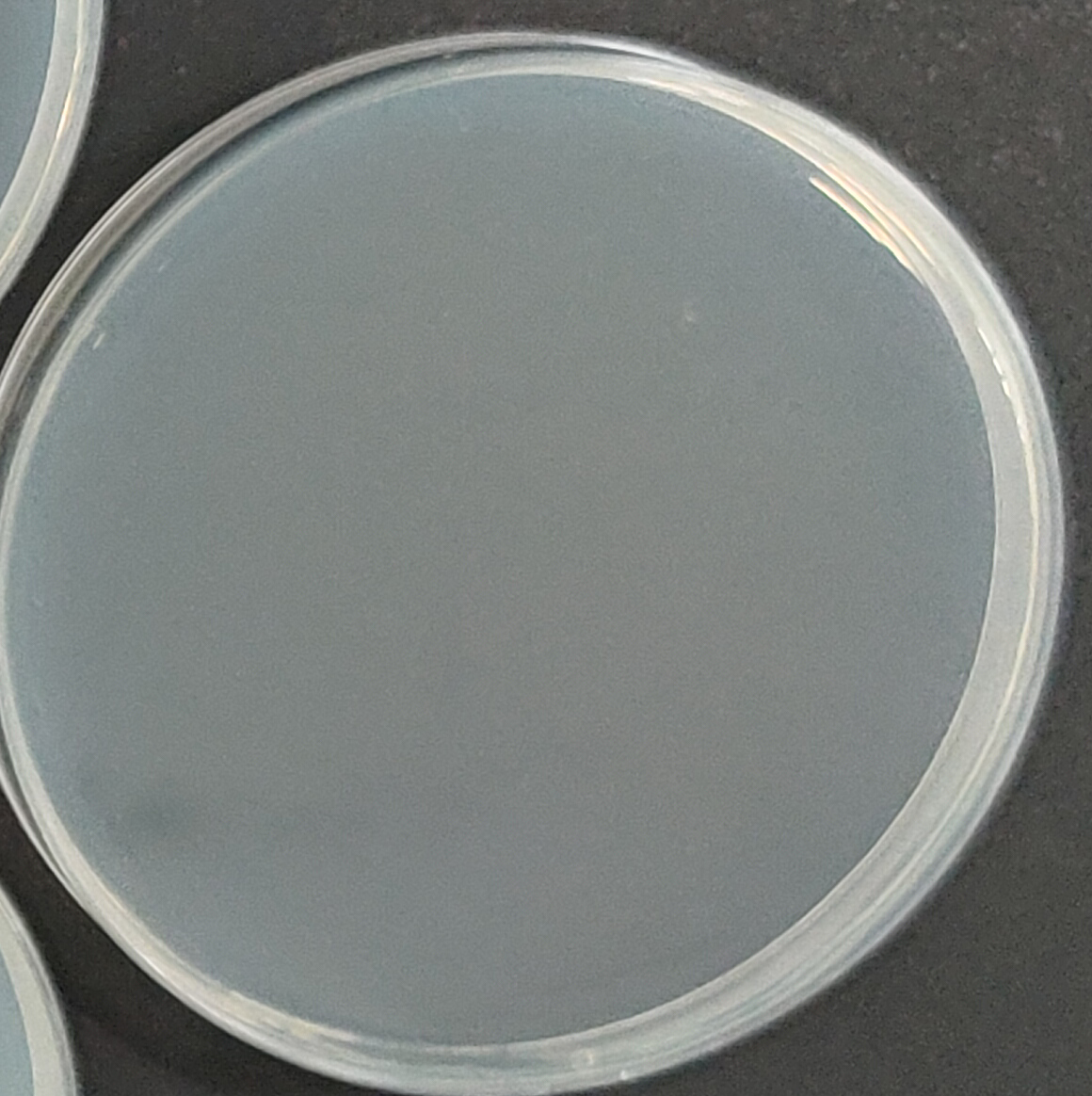

Supplement: Supplementary file 1 [file DataSheet1.zip › Data Sheet 1/Effect of 9 TCMMs on the adhesion ability of XDRAB/adhesion data/Furanone C30/0 (3).jpg]

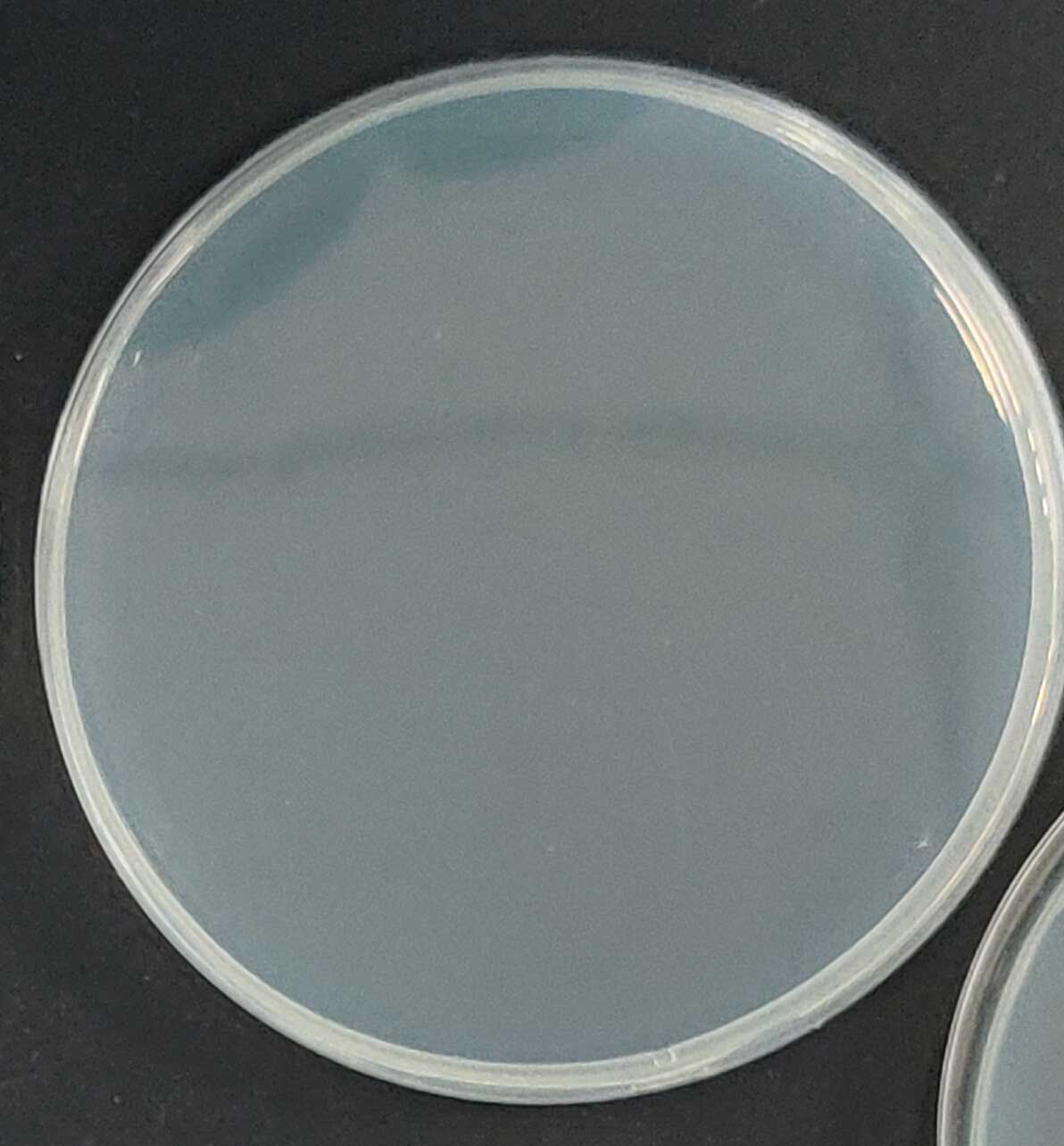

Supplement: Supplementary file 1 [file DataSheet1.zip › Data Sheet 1/Effect of 9 TCMMs on the adhesion ability of XDRAB/adhesion data/Furanone C30/0 (4).jpg]

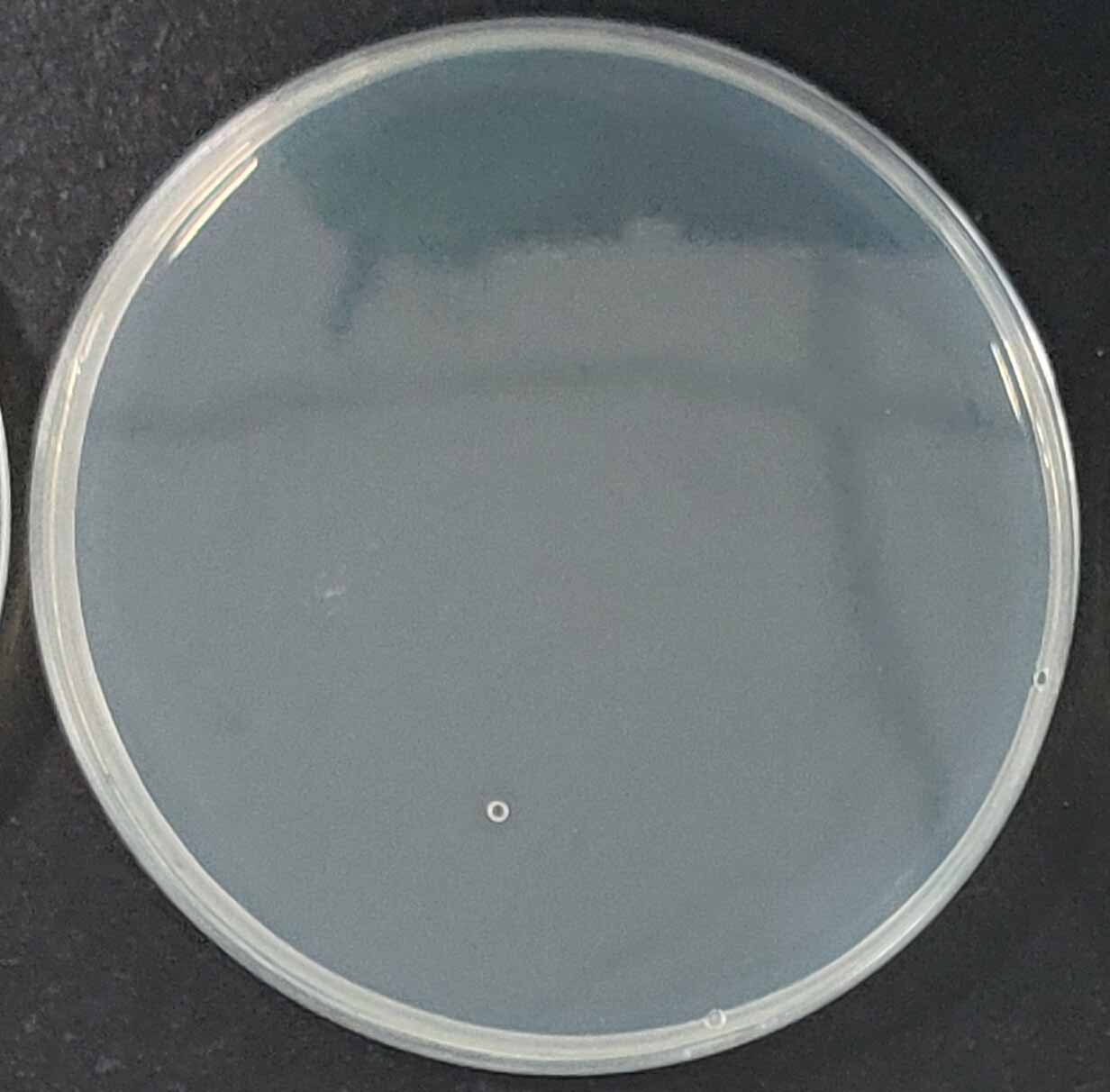

Supplement: Supplementary file 1 [file DataSheet1.zip › Data Sheet 1/Effect of 9 TCMMs on the adhesion ability of XDRAB/adhesion data/Furanone C30/0 (5).jpg]

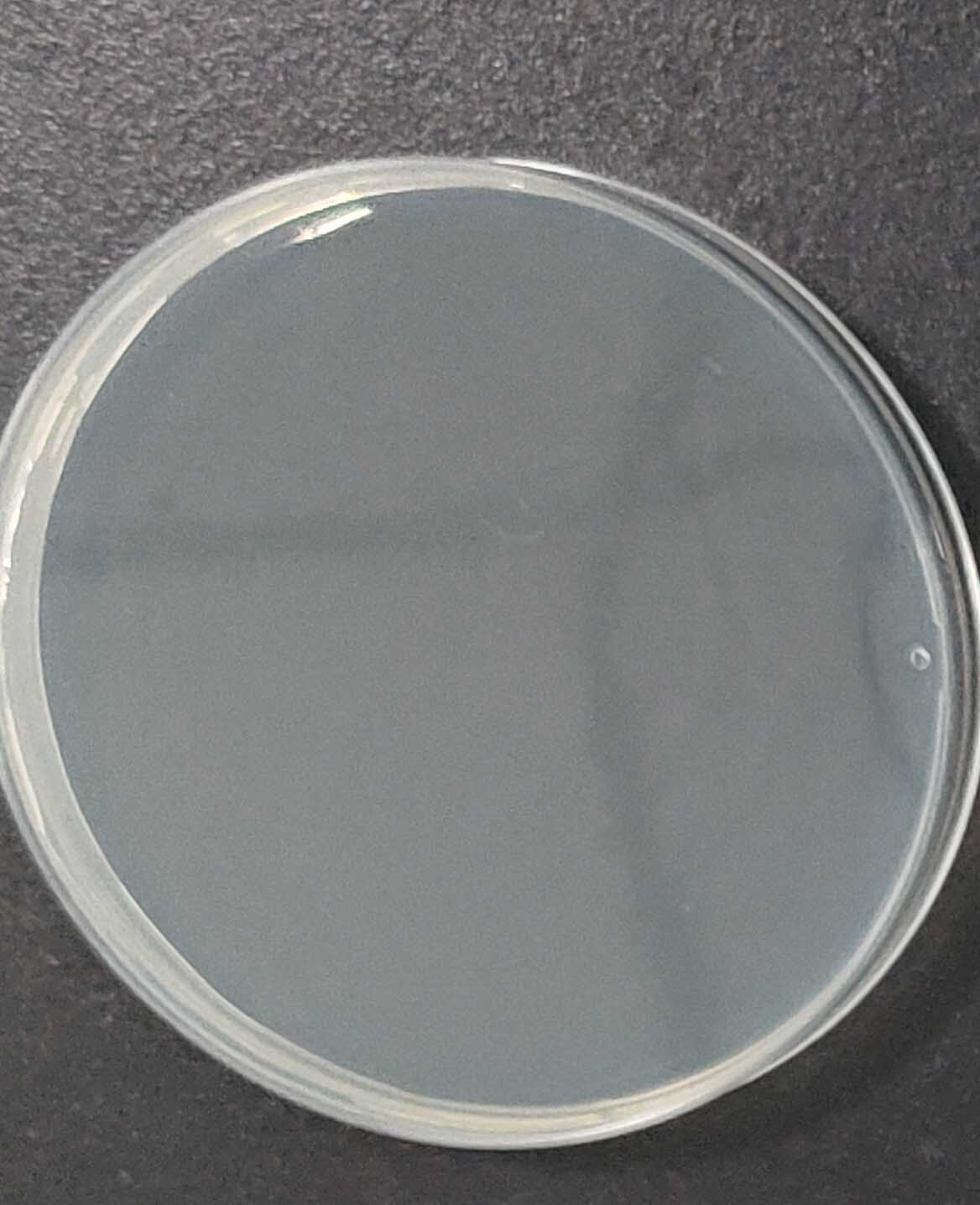

Supplement: Supplementary file 1 [file DataSheet1.zip › Data Sheet 1/Effect of 9 TCMMs on the adhesion ability of XDRAB/adhesion data/Furanone C30/0 (6).jpg]

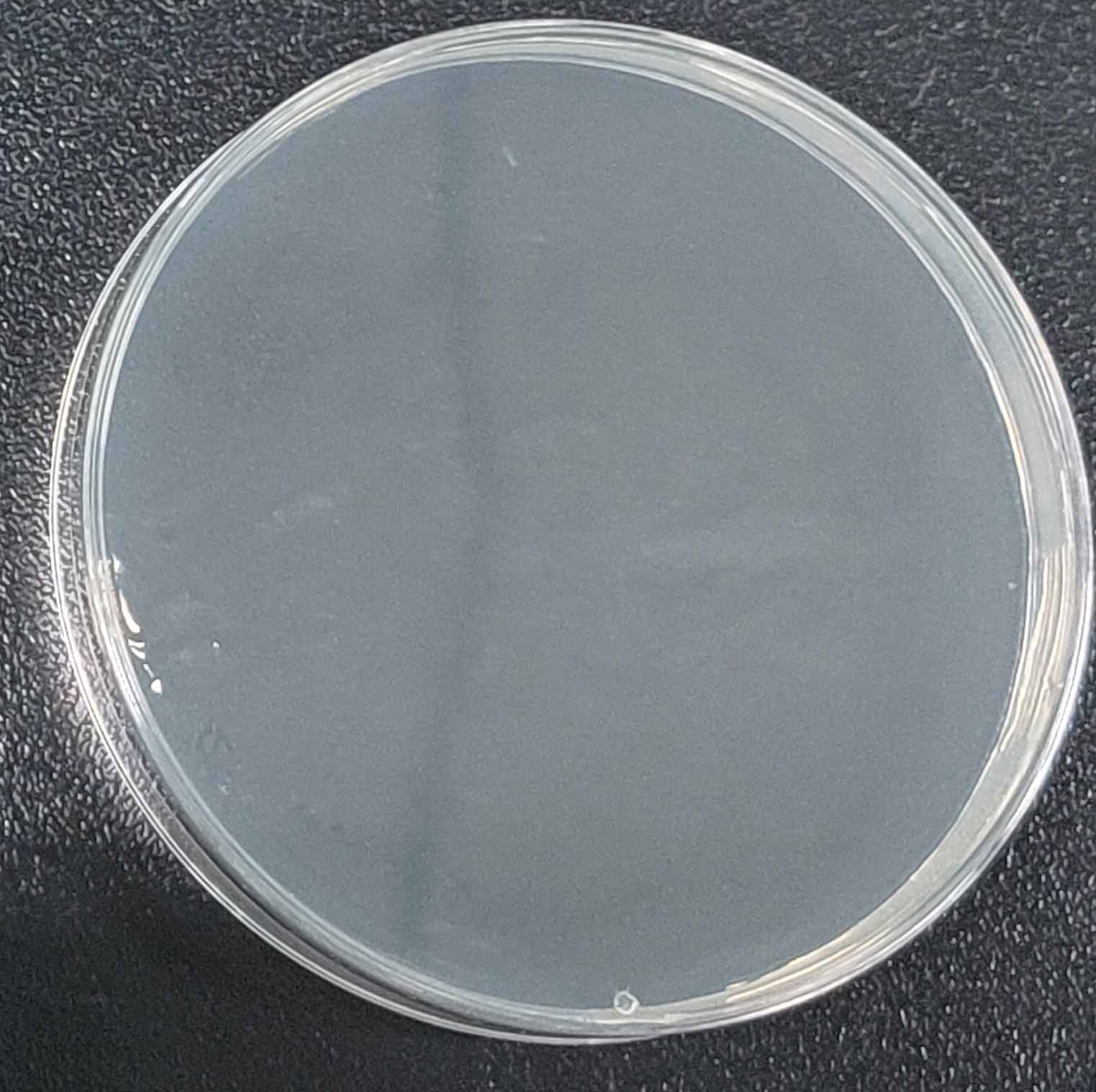

Supplement: Supplementary file 1 [file DataSheet1.zip › Data Sheet 1/Effect of 9 TCMMs on the adhesion ability of XDRAB/adhesion data/Furanone C30/0 (7).jpg]

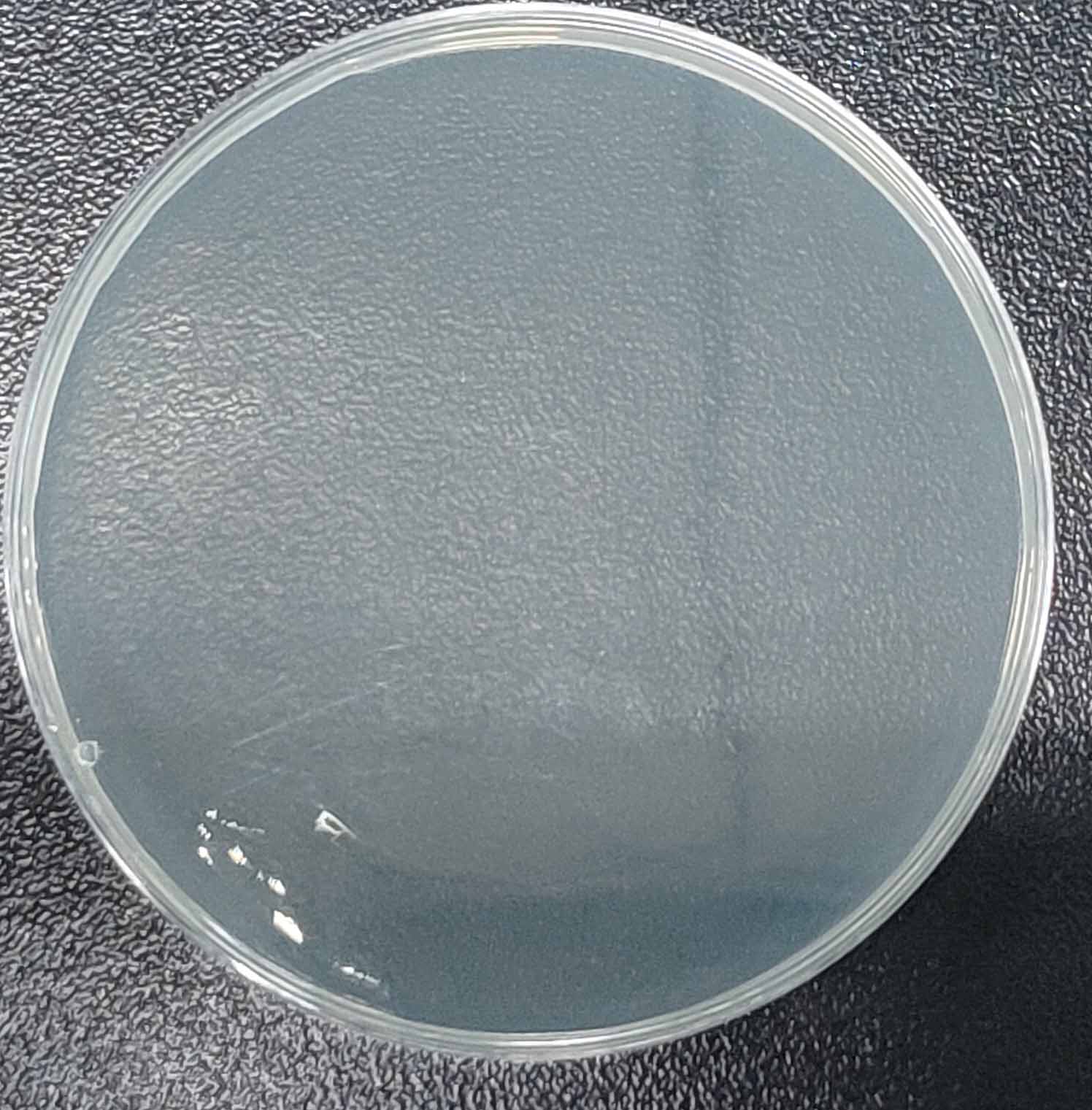

Supplement: Supplementary file 1 [file DataSheet1.zip › Data Sheet 1/Effect of 9 TCMMs on the adhesion ability of XDRAB/adhesion data/Furanone C30/0 (8).jpg]

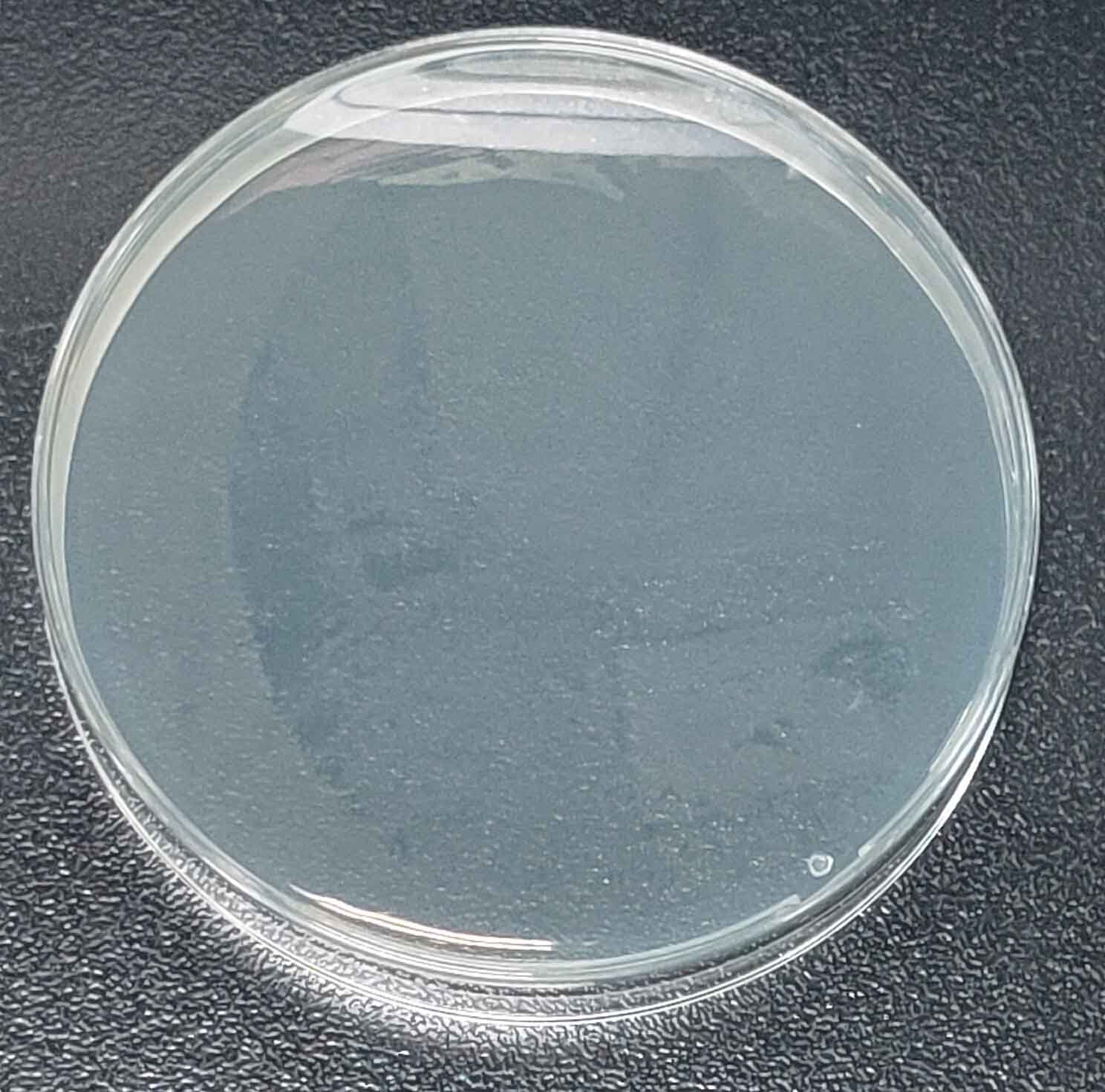

Supplement: Supplementary file 1 [file DataSheet1.zip › Data Sheet 1/Effect of 9 TCMMs on the adhesion ability of XDRAB/adhesion data/Furanone C30/0 (9).jpg]

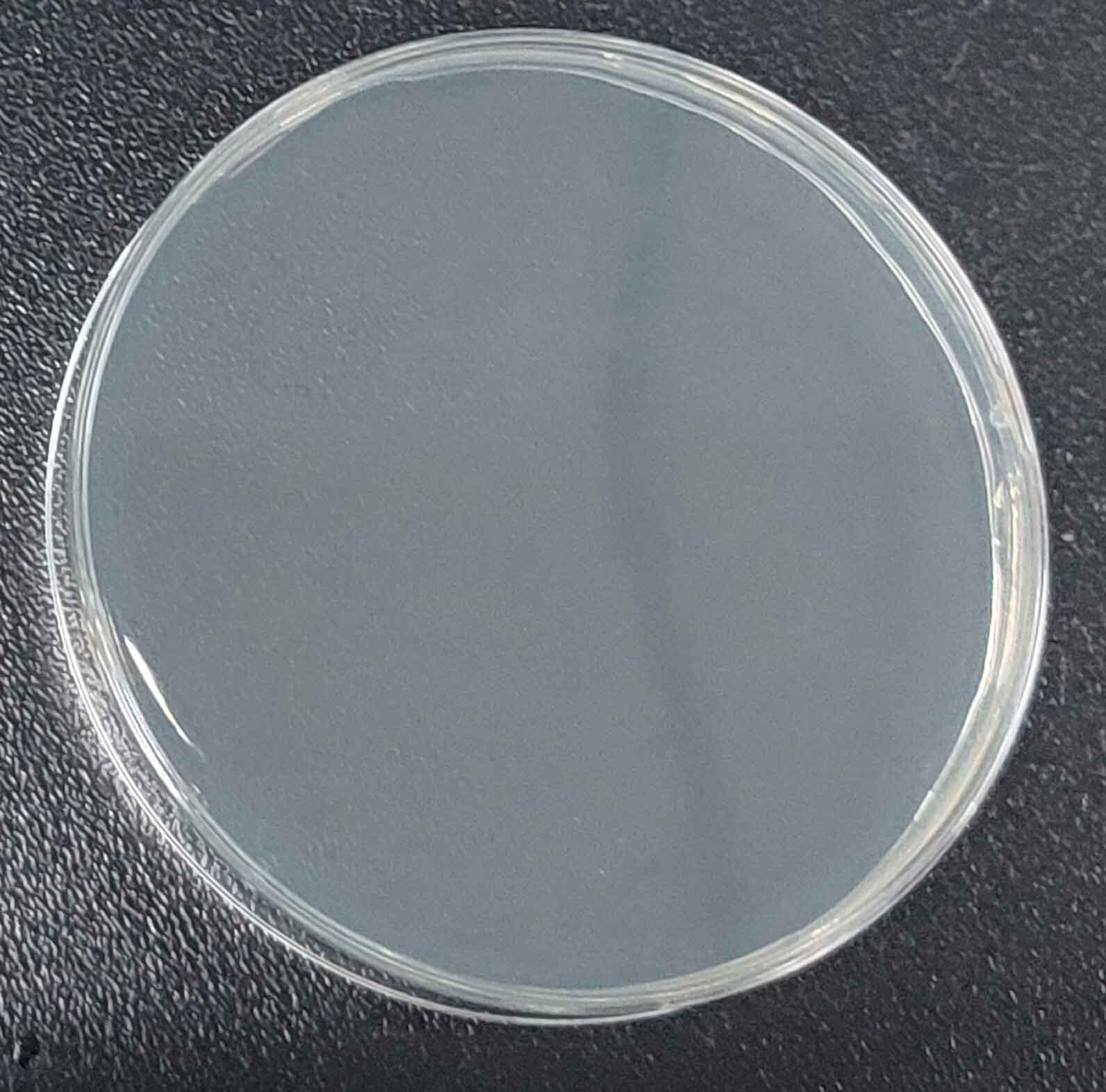

Supplement: Supplementary file 1 [file DataSheet1.zip › Data Sheet 1/Effect of 9 TCMMs on the adhesion ability of XDRAB/adhesion data/Furanone C30/0.jpg]

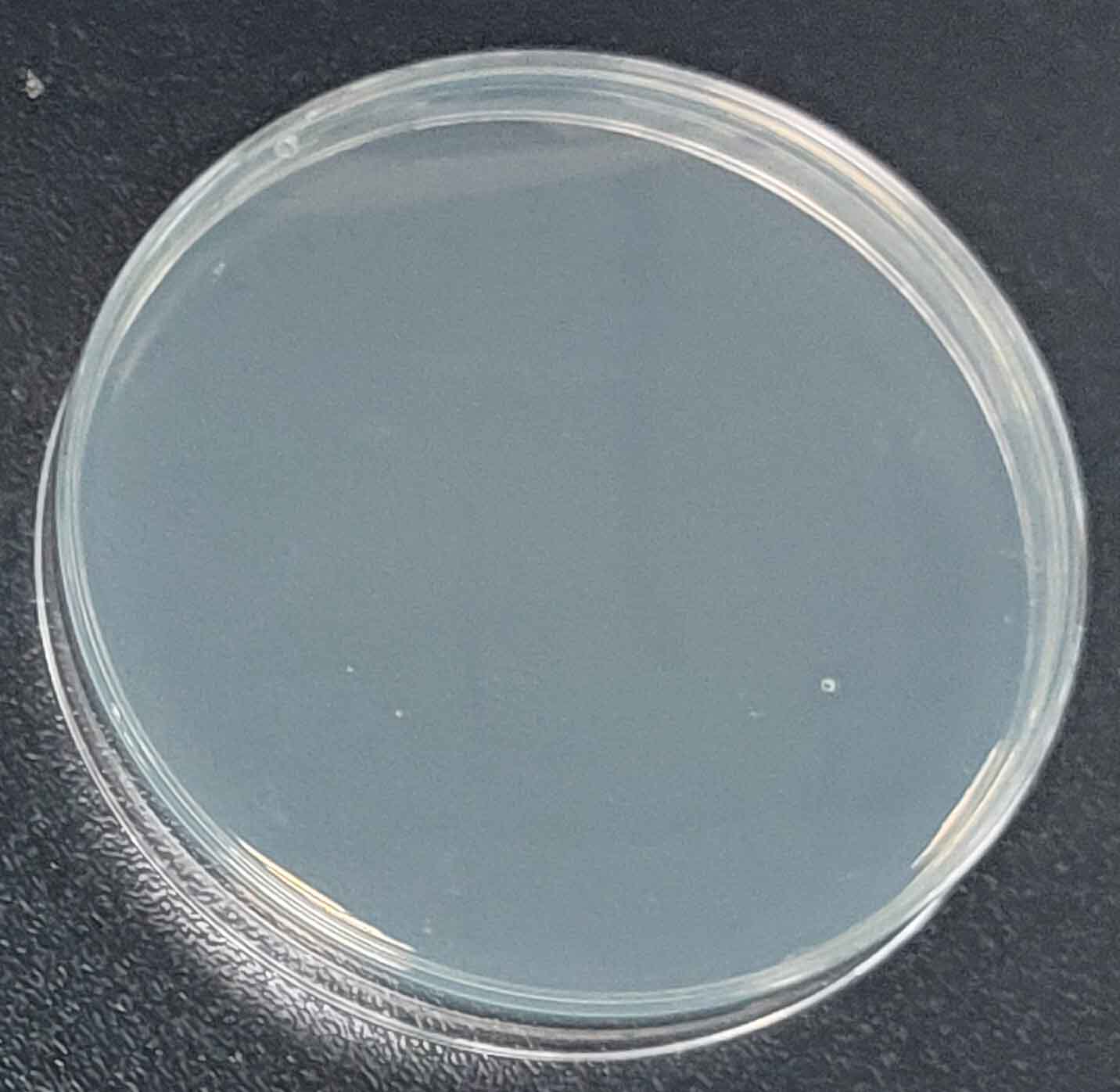

Supplement: Supplementary file 1 [file DataSheet1.zip › Data Sheet 1/Effect of 9 TCMMs on the adhesion ability of XDRAB/adhesion data/Furanone C30/1.jpg]

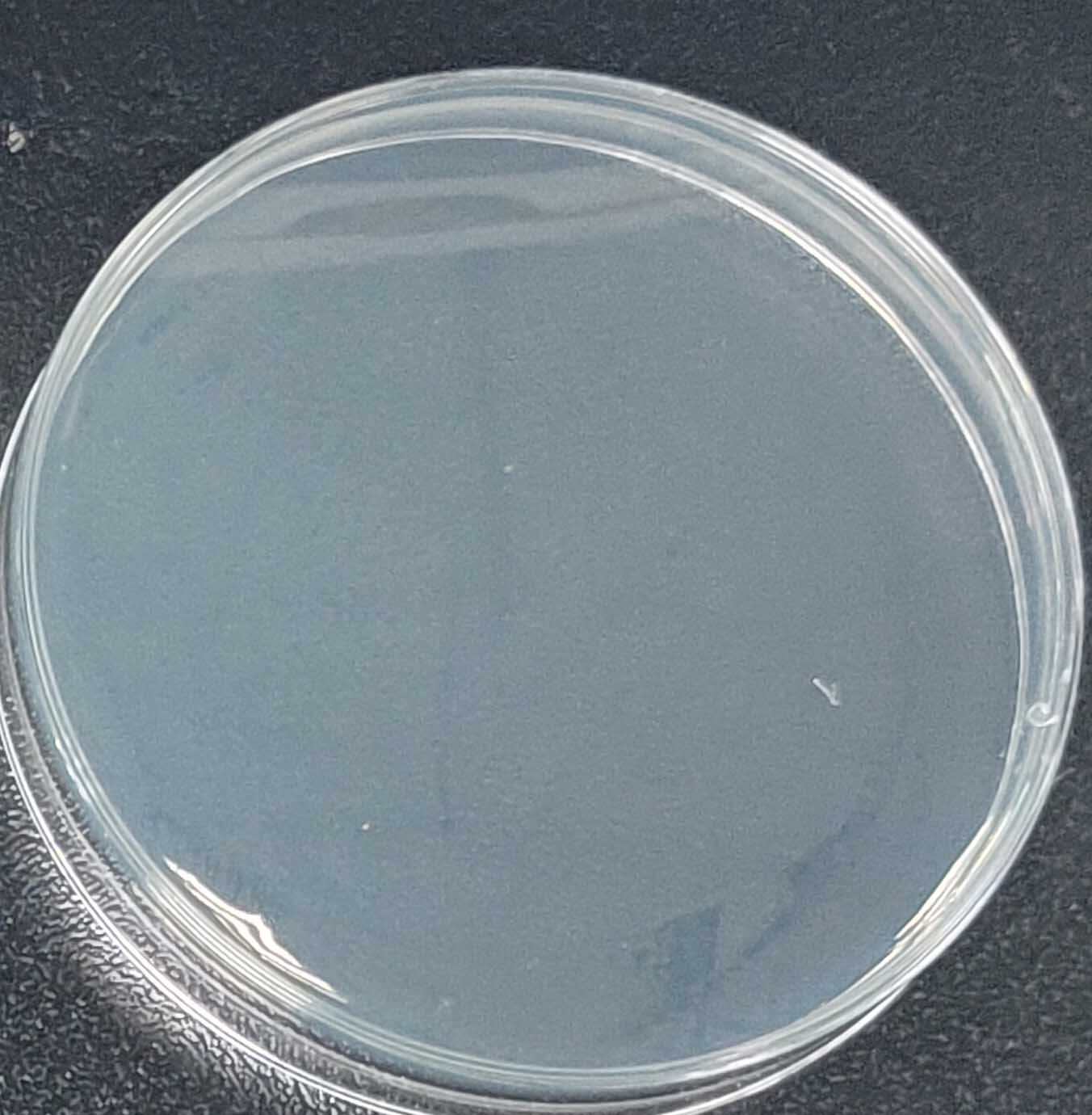

Supplement: Supplementary file 1 [file DataSheet1.zip › Data Sheet 1/Effect of 9 TCMMs on the adhesion ability of XDRAB/adhesion data/Furanone C30/2.jpg]

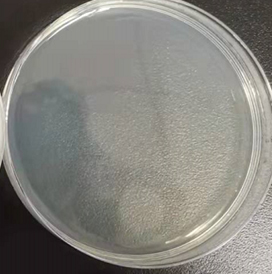

Supplement: Supplementary file 1 [file DataSheet1.zip › Data Sheet 1/Effect of 9 TCMMs on the adhesion ability of XDRAB/adhesion data/Hordenine/0 (2).jpg]

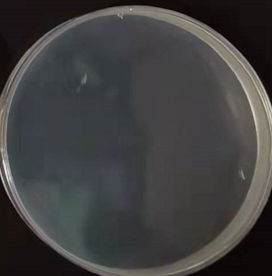

Supplement: Supplementary file 1 [file DataSheet1.zip › Data Sheet 1/Effect of 9 TCMMs on the adhesion ability of XDRAB/adhesion data/Hordenine/0 (3).jpg]

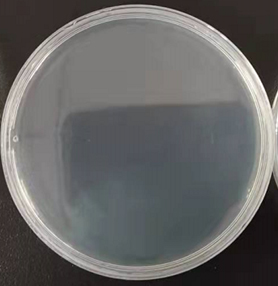

Supplement: Supplementary file 1 [file DataSheet1.zip › Data Sheet 1/Effect of 9 TCMMs on the adhesion ability of XDRAB/adhesion data/Hordenine/0 (4).jpg]

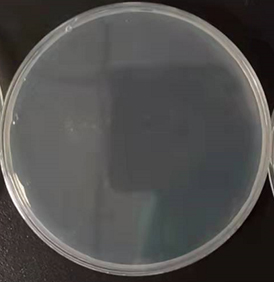

Supplement: Supplementary file 1 [file DataSheet1.zip › Data Sheet 1/Effect of 9 TCMMs on the adhesion ability of XDRAB/adhesion data/Hordenine/0 (5).jpg]

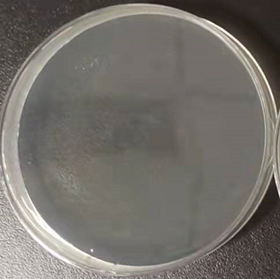

Supplement: Supplementary file 1 [file DataSheet1.zip › Data Sheet 1/Effect of 9 TCMMs on the adhesion ability of XDRAB/adhesion data/Hordenine/0 (6).jpg]

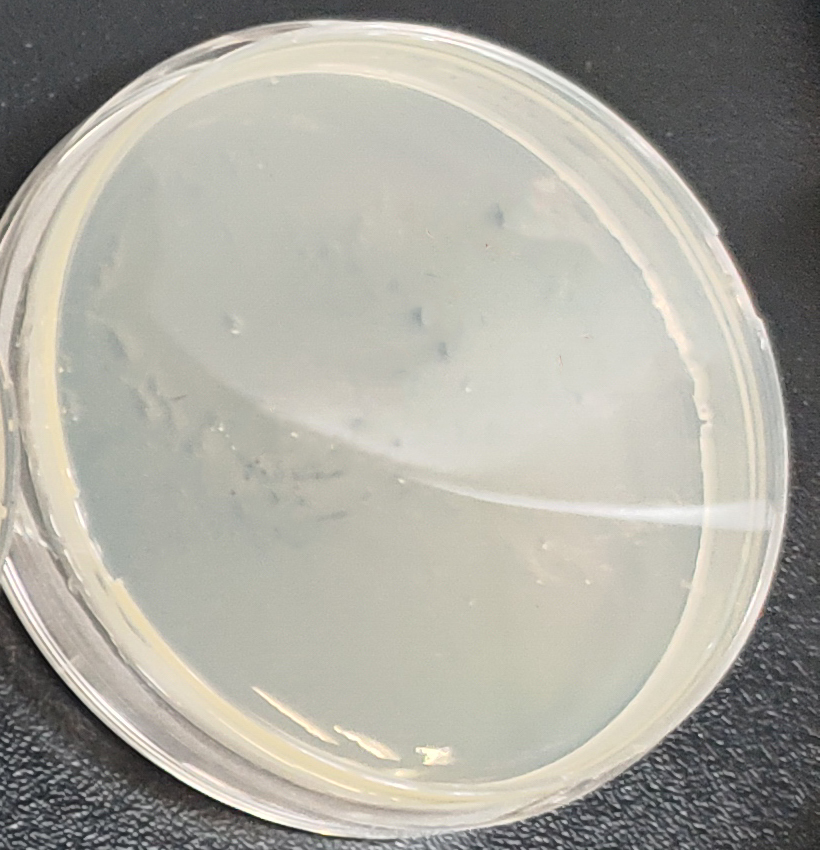

Supplement: Supplementary file 1 [file DataSheet1.zip › Data Sheet 1/Effect of 9 TCMMs on the adhesion ability of XDRAB/adhesion data/Hordenine/0 (7).jpg]

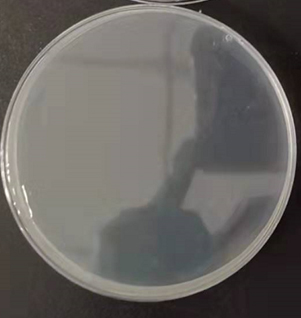

Supplement: Supplementary file 1 [file DataSheet1.zip › Data Sheet 1/Effect of 9 TCMMs on the adhesion ability of XDRAB/adhesion data/Hordenine/0 (8).jpg]

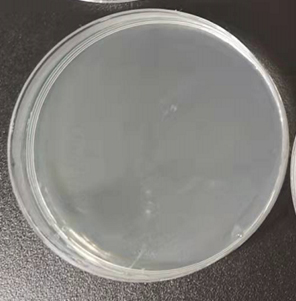

Supplement: Supplementary file 1 [file DataSheet1.zip › Data Sheet 1/Effect of 9 TCMMs on the adhesion ability of XDRAB/adhesion data/Hordenine/0 (9).jpg]

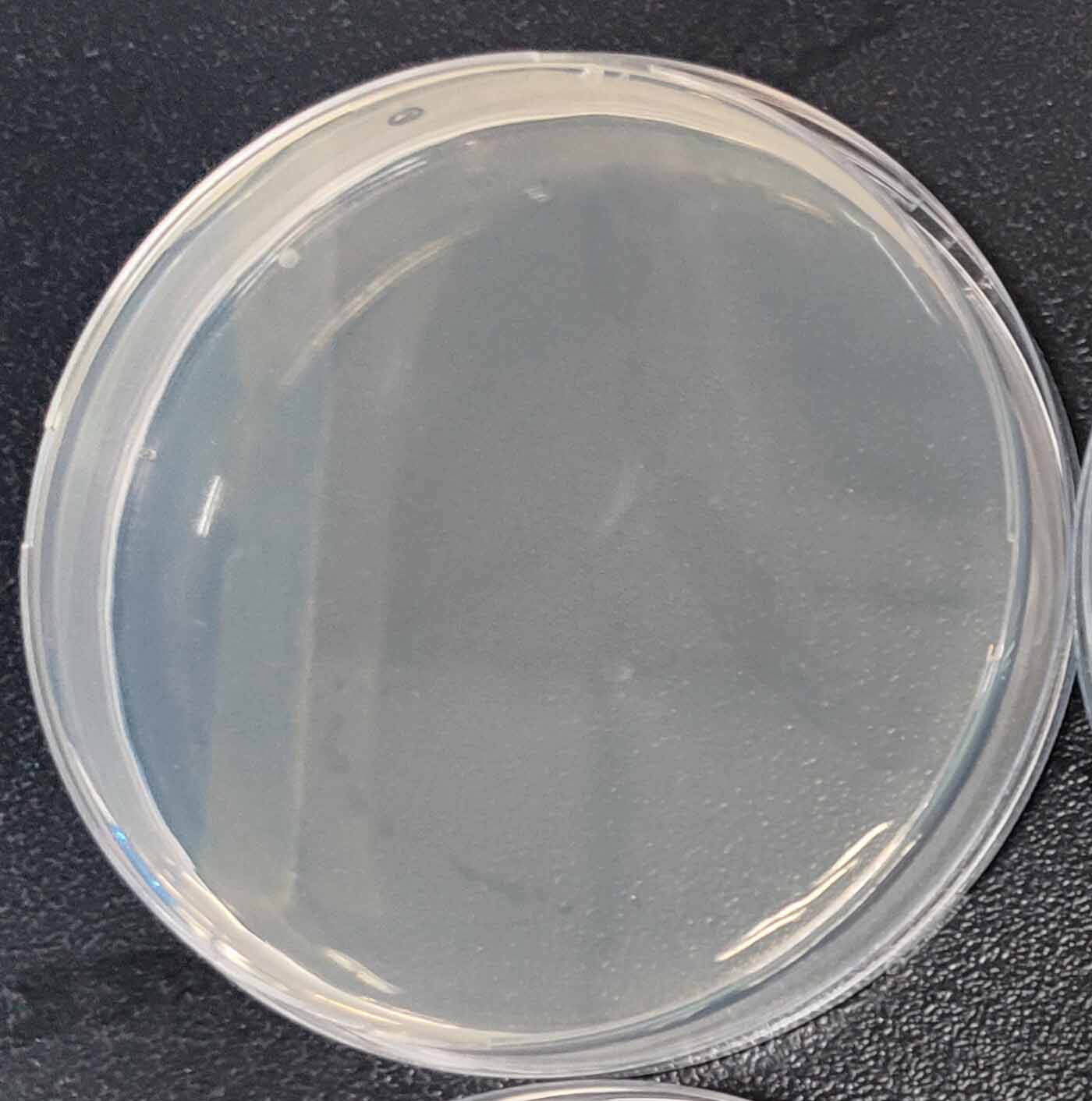

Supplement: Supplementary file 1 [file DataSheet1.zip › Data Sheet 1/Effect of 9 TCMMs on the adhesion ability of XDRAB/adhesion data/Hordenine/0.jpg]

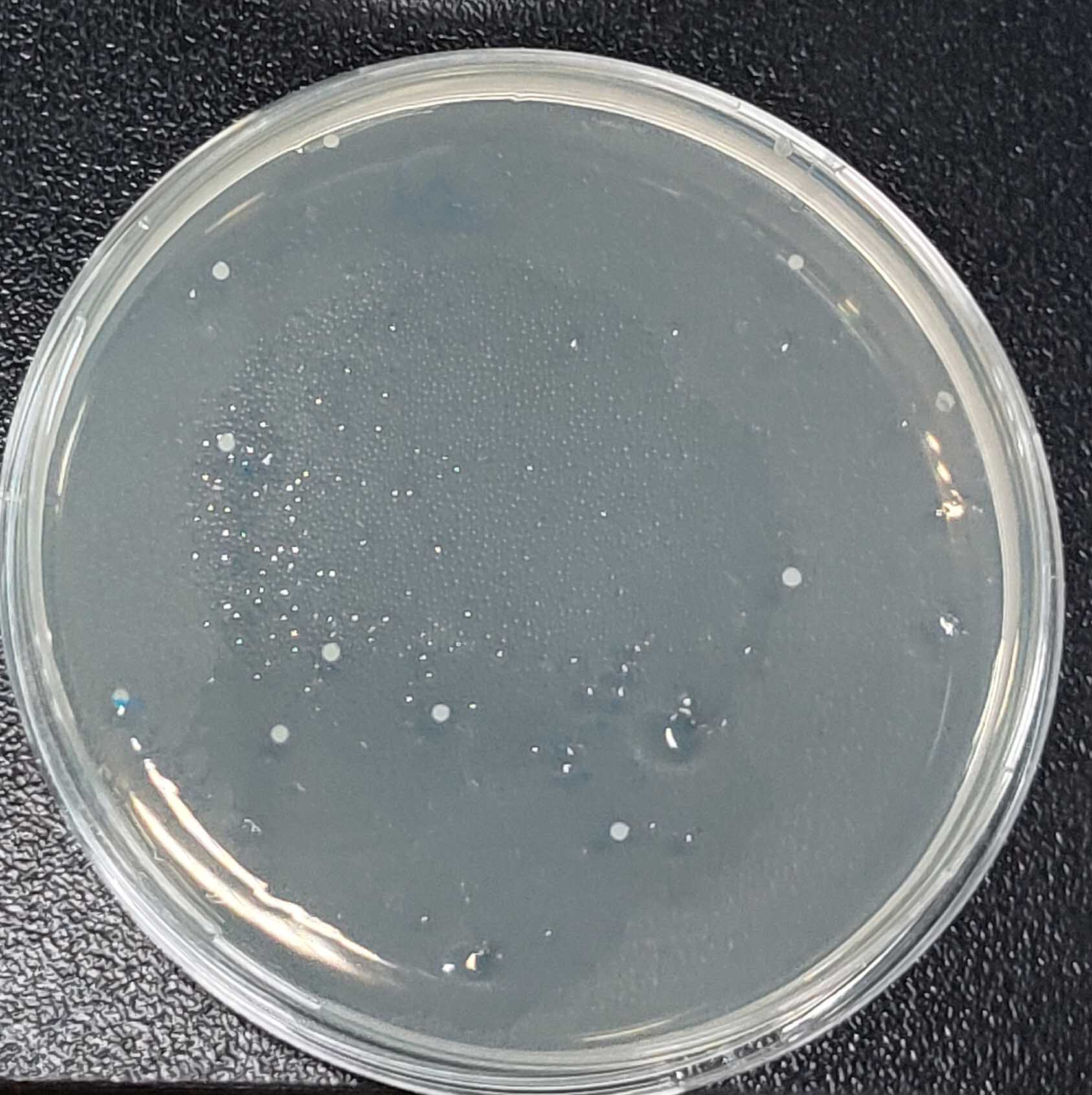

Supplement: Supplementary file 1 [file DataSheet1.zip › Data Sheet 1/Effect of 9 TCMMs on the adhesion ability of XDRAB/adhesion data/Hordenine/10.jpg]

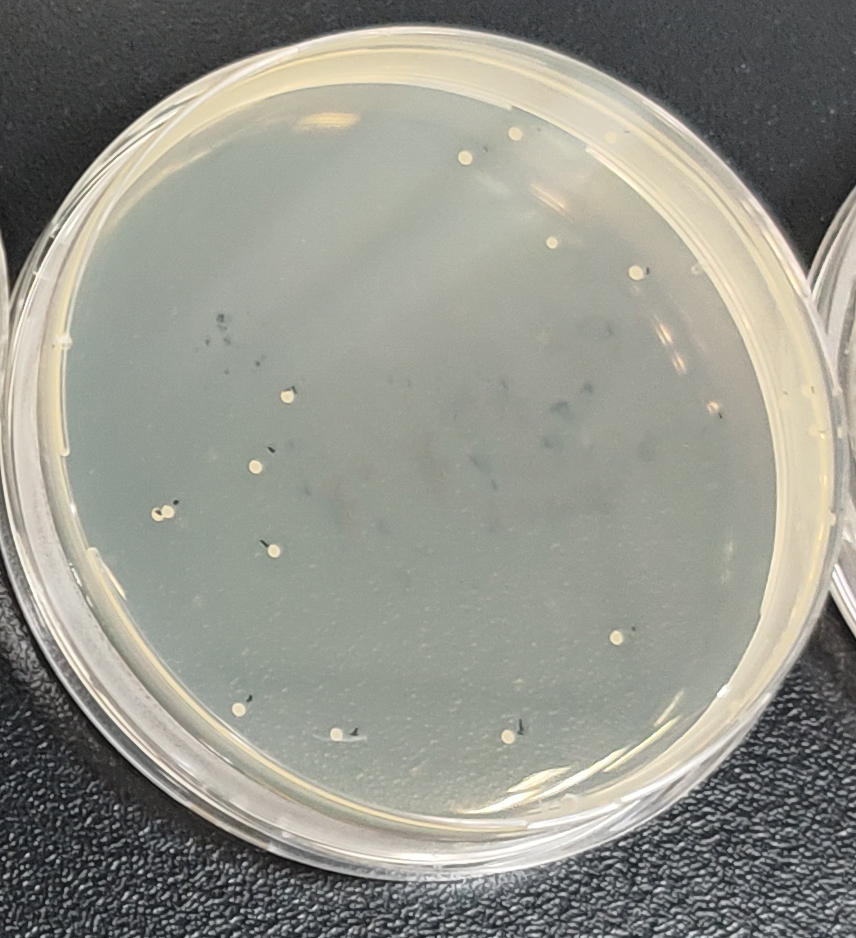

Supplement: Supplementary file 1 [file DataSheet1.zip › Data Sheet 1/Effect of 9 TCMMs on the adhesion ability of XDRAB/adhesion data/Hordenine/13.jpg]

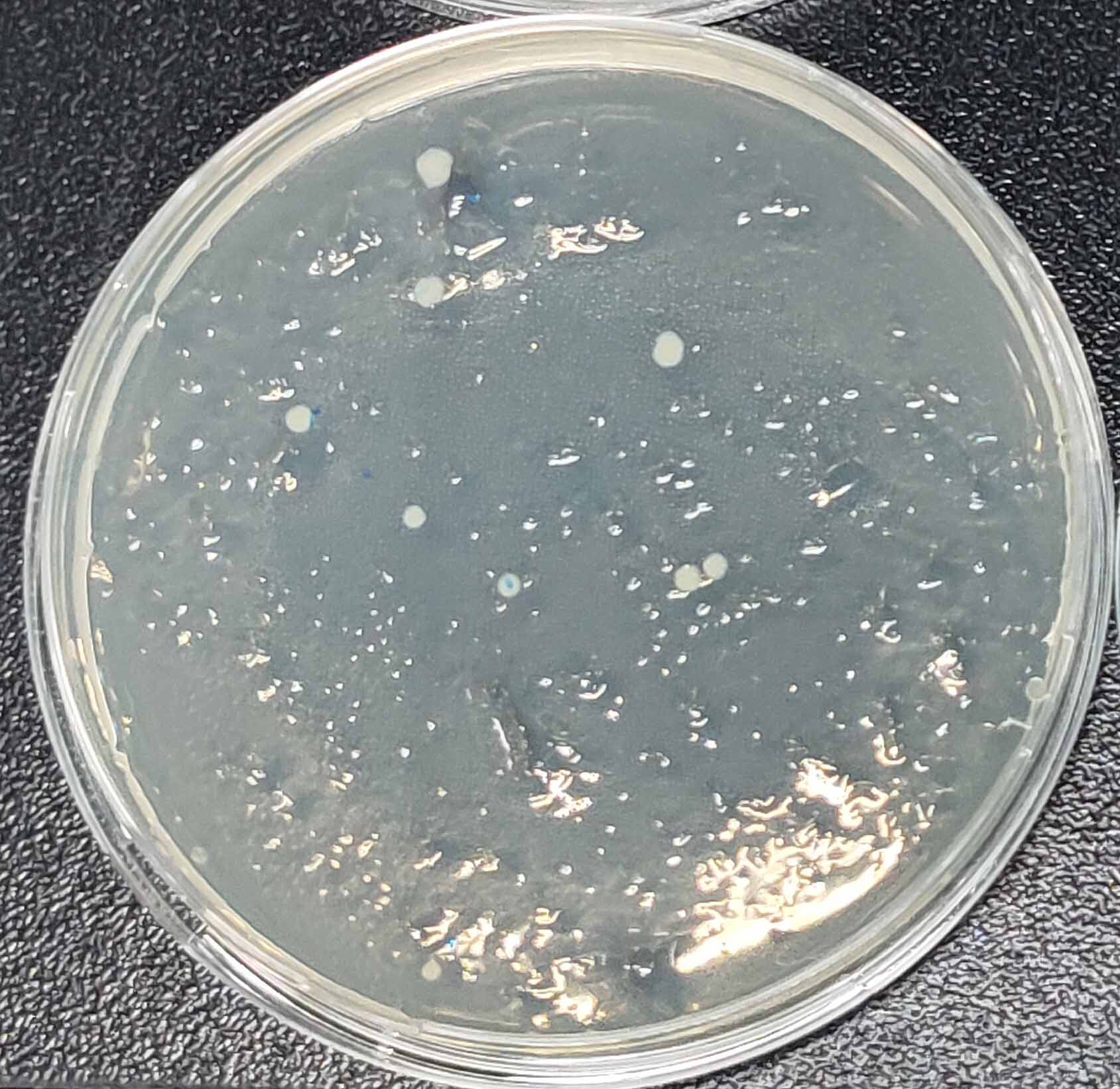

Supplement: Supplementary file 1 [file DataSheet1.zip › Data Sheet 1/Effect of 9 TCMMs on the adhesion ability of XDRAB/adhesion data/Hordenine/9.jpg]

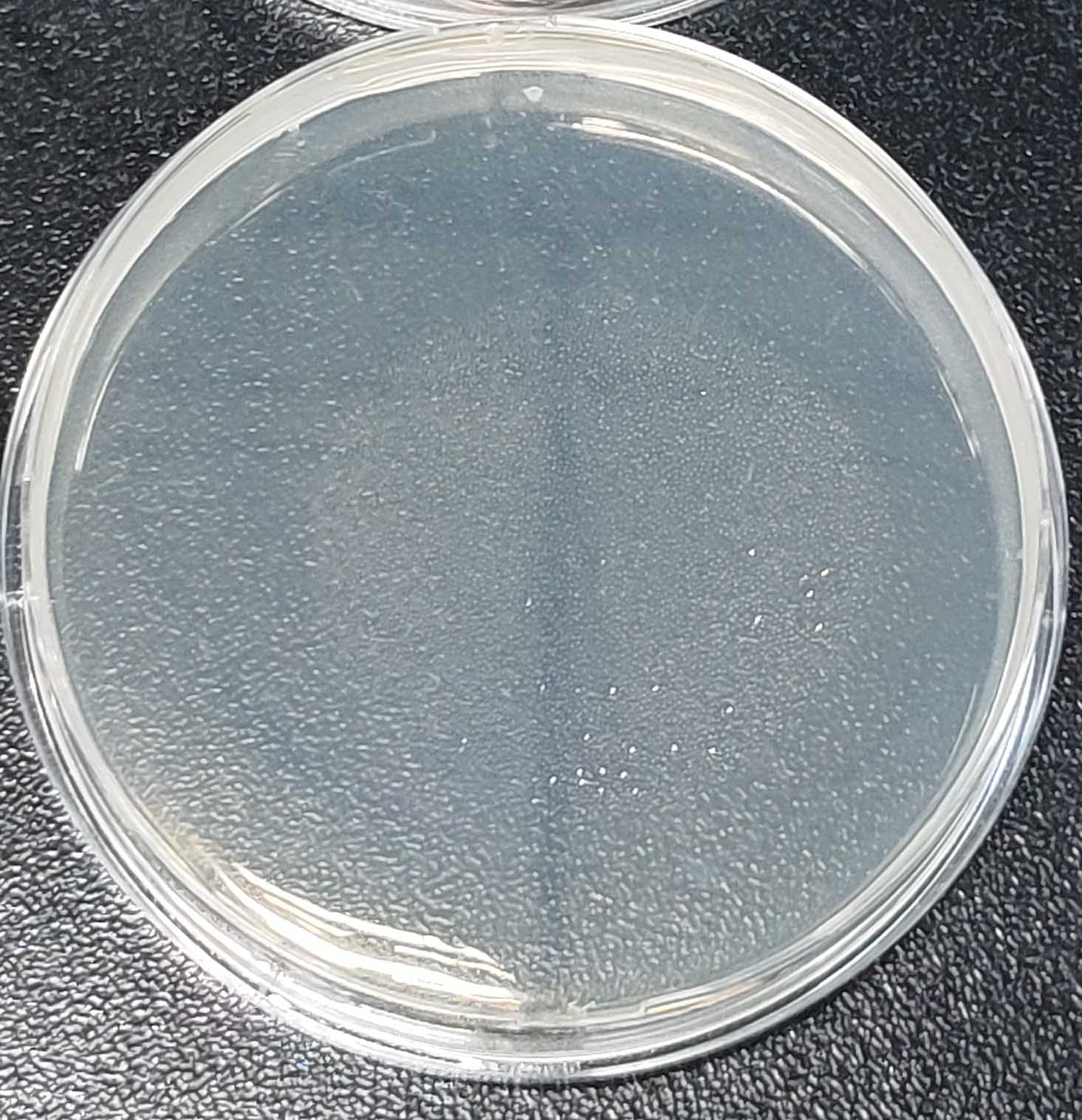

Supplement: Supplementary file 1 [file DataSheet1.zip › Data Sheet 1/Effect of 9 TCMMs on the adhesion ability of XDRAB/adhesion data/Kaempferol/0 (2).jpg]
